# Synthesis and Spectroscopic Characterization of Bis(thiadiazolo)benzoporphyrinoids: Insights into the Properties of Porphyrin-Type Systems with Strongly Electron-Withdrawing β,β’-Fused Rings

**DOI:** 10.3390/molecules30081822

**Published:** 2025-04-18

**Authors:** Timothy D. Lash, Catherine M. Cillo, Deyaa I. AbuSalim

**Affiliations:** 1 Department of Chemistry, Illinois State University, Normal, IL 61790, USA; cmcill2@yahoo.com (C.M.C.);; 2 Department of Chemistry, Rowan University, Glassboro, NJ 08028, USA; 3 STEM Department, Rowan College of South Jersey, Vineland, NJ 08360, USA

**Keywords:** annulated porphyrinoids, aromaticity, carbaporphyrins, electron-deficient heterocycles

## Abstract

A series of porphyrinoids fused to highly electron-withdrawing bis(thiadiazolo)benzene units have been prepared and spectroscopically characterized. These structures have modified chromophores and exhibit large bathochromic shifts. The nickel(II), copper(II) and zinc complexes of a bis(thiadiazolo)benzoporphyrin were prepared, and these showed strong absorptions above 600 nm that shifted to longer wavelengths with increasing atomic number for the coordinated metal cations. Although the investigated porphyrinoids were poorly soluble, proton NMR data could be obtained, and these demonstrated that the structures possess global aromatic character. This was confirmed with nucleus-independent chemical shift (NICS) calculations and anisotropy of induced current density (AICD) plots. The AICD plots also demonstrate that the fused heterocyclic unit is disconnected from the porphyrinoid *π*-system, and in this respect, it differs from phenanthroline-fused porphyrinoids as it shows the presence of extended conjugation pathways.

## 1. Introduction

**Figure 1:**
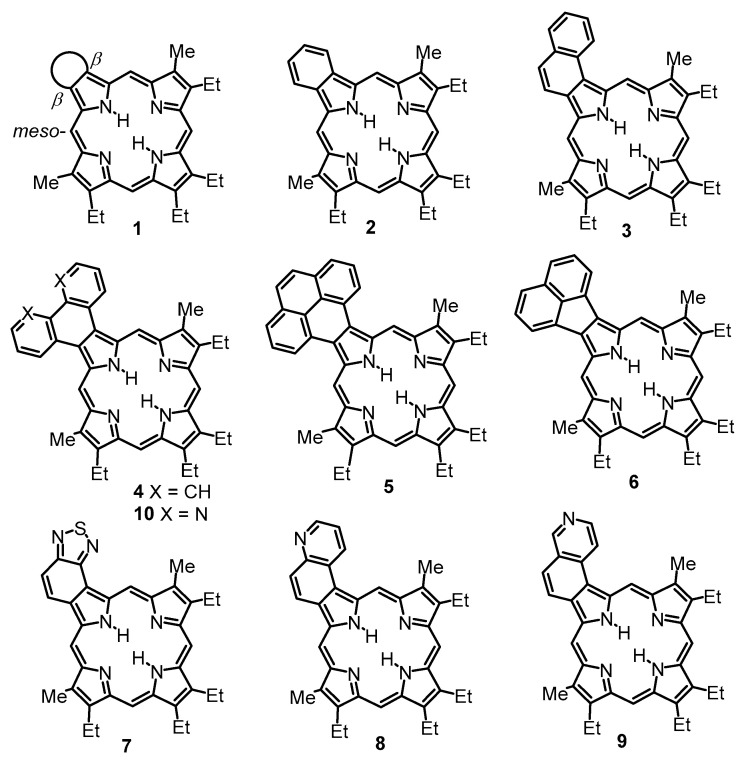
Selected structures of annelated porphyrins.

Porphyrins with β,β’-fused aromatic rings **1** have been widely explored due to their modified reactivity and the presence of extended chromophores [1,2,3]. Although the UV-vis spectra for monobenzoporphyrins **2** [4,5], naphtho[1,2-*b*]porphyrins **3** [6,7], phenanthro[5,6-*b*]porphyrins **4** [8,9] and pyrenoporphyrins **5** [10] and related systems [11] only show minor bathochromic shifts, acenapthoporphyrins **6** [12,13] and thiadiazolobenzoporphyrins **7** [14] exhibit highly modified spectra [15] (Figure 1). These effects are magnified when two or more fused rings are present [1,2,3,15]. Evidence showing that the diatropic ring currents for structures of this type can extend through the fused arene units have also been presented [16]. Porphyrins with fused heterocyclic rings [17], including quinoporphyrin **8**, isoquinoporphyrin **9** [18], and phenanthrolinoporphyrins **10** [19,20], are also known and show a similar range of spectroscopic properties.

**Figure 2:**
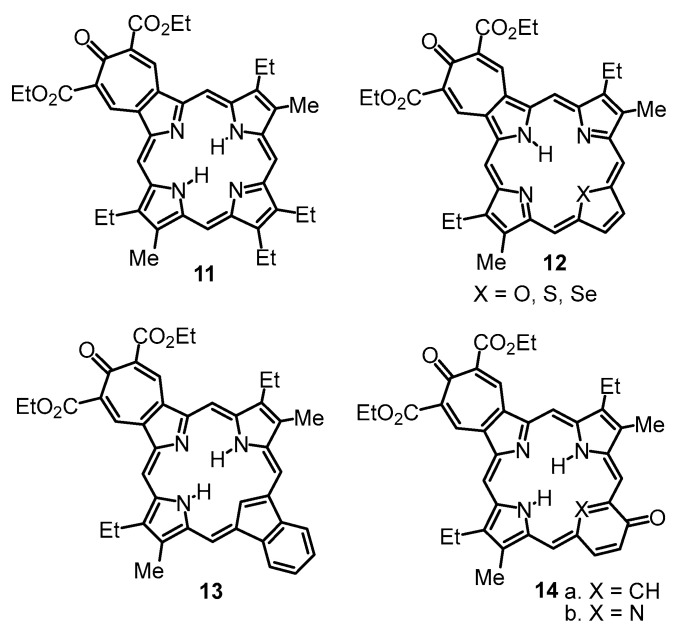
Tropone-fused porphyrinoids.

Studies of this type have been extended to annulated porphyrin analogs. For instance, a series of structures with fused tropone units were prepared [21], specifically porphyrin **11**, heteroporphyrins **12**, carbaporphyrin **13**, oxybenziporphyrin **14a**, and oxypyriporphyrin **14b** (Figure 2). These porphyrinoids showed intriguing spectroscopic properties. In addition to giving red-shifted UV-vis spectra, the proton NMR spectra were also unusual. The external *meso*-proton resonances were shifted upfield compared to structures lacking the fused tropone units, indicating that the macrocyclic ring currents were reduced [21]. This interpretation was supported by the resonances for the methyl substituents as these were also shifted comparatively upfield from the expected value of 3.6 ppm. For instance, carbaporphyrin **13** gave two 2H resonances for the *meso*-protons at 9.04 and 8.50 ppm, results that are 1–1.5 ppm upfield from the expected values. However, the internal protons are shifted upfield as well, indicating that they have enhanced diamagnetic ring currents instead. For **13**, the NHs appeared at −6.99 ppm, while the 21-H was located at −9.29 ppm, chemical shifts that are over 2 ppm upfield from the expected values [21]. The origin of these contradictory results cannot be easily explained.

**Figure 3:**
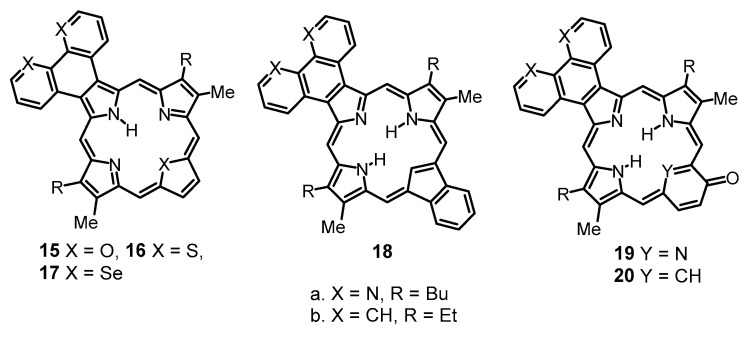
1,10-Phenanthroline-fused porphyrinoids.

**Figure 4:**
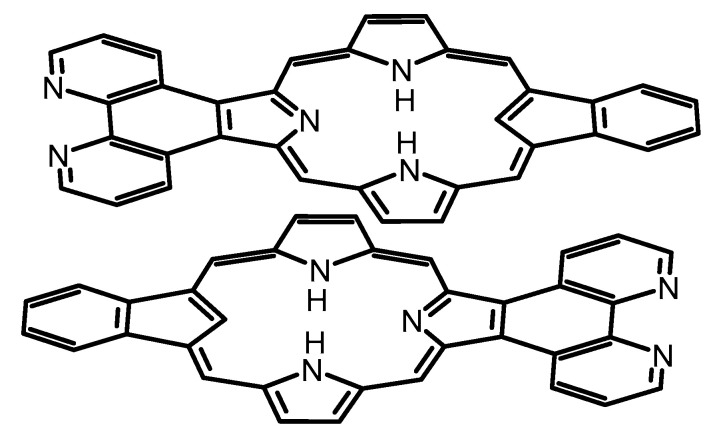
A possible head-to-tail face-to-face intramolecular interaction for a phenanthroline-fused carbaporphyrin.

As the tropone unit is strongly electron-withdrawing, it was anticipated that porphyrinoids directly fused to 1,10-phenanthroline might exhibit the same phenomenon. A series of phenanthroline-fused porphyrinoids (series a) were prepared [22] and contrasted with equivalent structures fused to phenanthrene (series b) (Figure 3) [9,23,24]. Solubility was a limiting factor, but the results demonstrated that similar upfield shifts to the internal and external proton resonances were found in series a but not for series b (Table 1, Table 2 and Table 3). Phenanthrolinoporphyrin **10** (series a) shows significant upfield shifts to the *meso*-protons compared to its structural analog **4** (series b), although the values for the inner NH protons are less clear-cut [22] (Table 1). Similar comparisons could be made for thiaporphyrins **16a**/**b** and selenaporphyrins **17a**/**b**. A reduction in diatropic character in porphyrinoids due to the presence of electron-withdrawing substituents has been noted by others [23,24], and the results given in Table 1 might be considered to be a result of this factor. Nevertheless, the NH resonances for series a are mostly further upfield than the results for series b, which is the opposite to expectations. However, the data for carbaporphyrins **18a** and **18b** provide more clarity [22] (Table 2). The *meso*-protons are significantly shielded, as were the methyl substituents, for phenanthrolinocarbaporphyrin **18a** compared to the analogous phenanthrene-fused structure **18b** [25]. Some variations were noted due to temperature changes, but the NH and 21-H protons were shifted upfield by ca. 2 ppm, thereby showing similar contradictory results to tropone-fused porphyrinoid **13**. Similar contrasting results are seen for oxypyriporphyrins **19a** and **19b**, as well as for oxybenziporphyrins **20a** and **20b** [26] (Table 3). Nucleus-independent chemical shift (NICS) calculations showed that the phenanthrolinoporphyrinoids **10** and **15a**–**20a** retained fully aromatic properties, adding further confusion [22]. Both the tropone- and phenanthroline-fused series showed similar upfield shifts to both the internal and external protons resonances, where, on balance, the macrocycles appear to have reduced diatropic character, but the NICS calculations contradict this conclusion. One possibility is that the shifts result from selective intermolecular aggregation in a face-to-face head-to-tail orientation (Figure 4), but the limited solubility of these structures makes this hypothesis difficult to investigate.

**Scheme 1:**
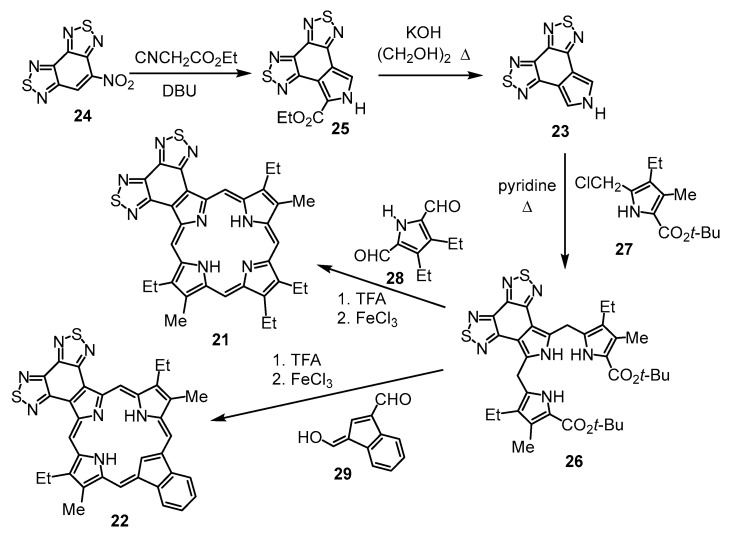
Synthesis of bis(thiadiazolo)benzopyrroles and related porphyrinoids.

In order to better understand this phenomenon, further examples of porphyrinoids with fused electron-withdrawing subunits were required. Specifically, some preliminary investigations had been carried out on the synthesis of a porphyrin **21** and a benzocarbaporphyrin **22** with strongly electron-withdrawing fused bis(thiadiazolo)benzo units [27] (Scheme 1). Unfortunately, the electron-deficient character of intermediary pyrrole **23** with the fused heterocyclic unit drastically reduced reactivity, and this inhibits the formation of a key tripyrrolic intermediate. Nevertheless, this approach provides access to a series of porphyrinoids with an unusual highly electron-withdrawing fused heterocycle and provides important new insights into how ring fusion influences the overall aromatic character of these macrocycles [28].

## 2. Results and Discussion

4-Nitrobenzo[1,2-*c*:3,4-*c*’]bis(1,2,5)thiadiazole (**24**) condensed with ethyl isocyanoacetate in the presence of 1,8-diazabicyclo[5.4.0]undec-7-ene (DBU) afforded bis-thiadiazolobenzo[4,5-*c*]pyrrole **25** in 84% yield [27] (Scheme 1). Cleavage of the ethyl ester with KOH in ethylene glycol at 180 °C gave a quantitative yield of the related unsubstituted heterocycle **23**. Attempts to form tripyrrane **26** under acidic conditions were unsuccessful. However, when chloromethylpyrrole **27** was treated with pyridine for 1 h and then reacted with **23** under refluxing conditions, tripyrrane **26** could be isolated in 25% yield [27]. Treatment of **26** with trifluoroacetic acid (TFA) to cleave the terminal *tert*-butyl ester groups, followed by condensation with pyrrole dialdehyde **28** or indene dialdehyde **29**, afforded porphyrin **21** in 35% yield and carbaporphyrin **22** in 25% yield [27] (Scheme 1).

**Scheme 2:**
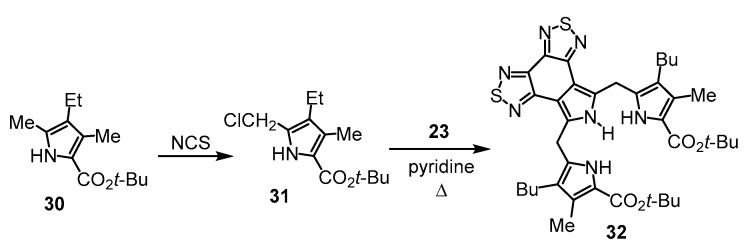
Synthesis of a tripyrrane intermediate.

Unfortunately, these products are virtually insoluble in organic solvents, and characterization of the free base forms by NMR spectroscopy was not possible. In an attempt to overcome this problem, a tripyrrane with butyl substituents was targeted (Scheme 2). *tert*-Butyl 4-butyl-3,5-dimethylpyrrole-2-carboxylate (**30**) was reacted with *N*-chlorosuccinimide (NCS) to give chloromethylpyrrole **31**. As was the case for **27** [14], chloromethylpyrrole **31** proved to be unstable. As it was too soluble in solvents such as hexane to facilitate recrystallization and chromatography led to decomposition, **31** was used without purification. The crude chloromethylpyrrole was stirred with pyridine for 1 h at room temperature to generate a pyridinium derivative, **23** was added, and the resulting mixture was refluxed under nitrogen for 16 h. Following column chromatography on silica, eluting with 1% triethylamine–dichloromethane, crude tripyrrane **32** was isolated in 22% yield.

**Scheme 3:**
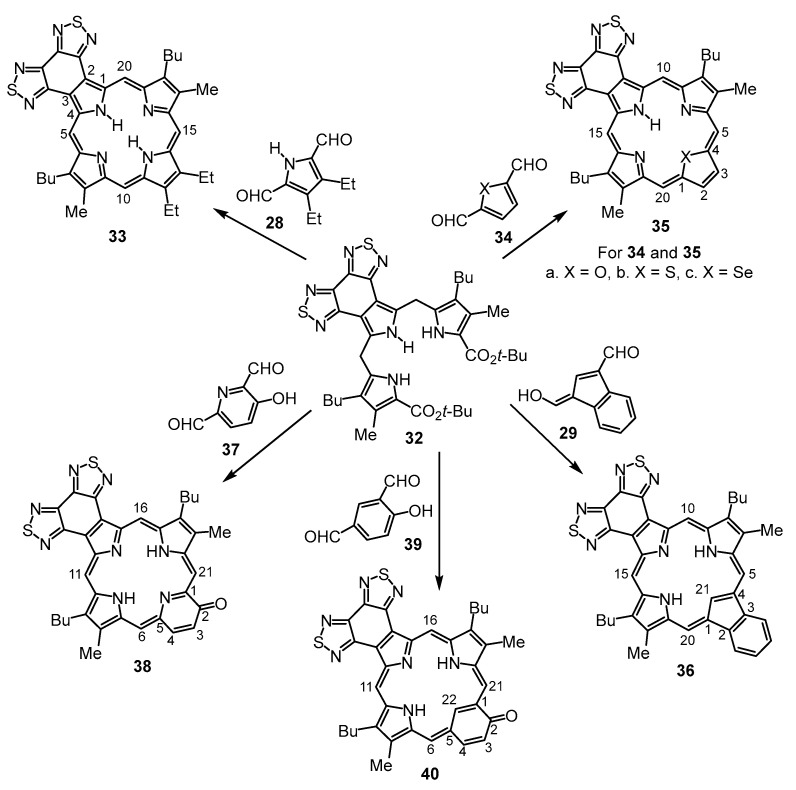
Synthesis of a series of porphyrin analogs fused to bis(thiadiazolo)benzene.

Tripyrrane **32** was deprotected with TFA and reacted with a series of dialdehydes to give, following oxidation with aqueous solutions of ferric chloride, porphyrin **33,** and a series of porphyrin analogs (Scheme 3). Reaction with pyrrole dialdehyde **28** [29], followed by purification by column chromatography on grade 3 alumina and recrystallization from chloroform–methanol, gave bis(thiadiazolo)benzoporphyrin **33** in 36% yield. Similarly, condensation with furan dialdehyde **34a** gave oxaporphyrin **35a**, but it was necessary to purify this system in protonated form after washing the solution with hydrochloric acid. The resulting hydrochloride was isolated in 20% yield. Thiophene dialdehyde **34b** gave relatively poor results affording thiaporphyrin **35b** in only 8% yield. Selenophene dialdehyde gave even worse results, affording only trace amounts of impure selenaporphyrin **35c**. It is unclear why these heteroporphyrins are generated in inferior yields, and while the larger chalconide atoms might inhibit cyclizations, this methodology has been utilized to prepare thia- and selenaporphyrins [30], including phenanthrene- and phenanthroline-fused structures [9,22], in good to excellent yields. Reaction of indene dialdehyde **29** [31] with **32** afforded carbaporphyrin **36** in 24% yield, while reaction with 3-hydroxy-2,6-pyridinedicarbaldehyde (**37**) [32] gave oxypyriporphyrin **38** in 32% yield. Poorer results were obtained with 4-hydroxy-isophthalaldehyde (**39**), but oxybenziporphyrin **40** could still be isolated in 12% yield.

**Figure 5:**
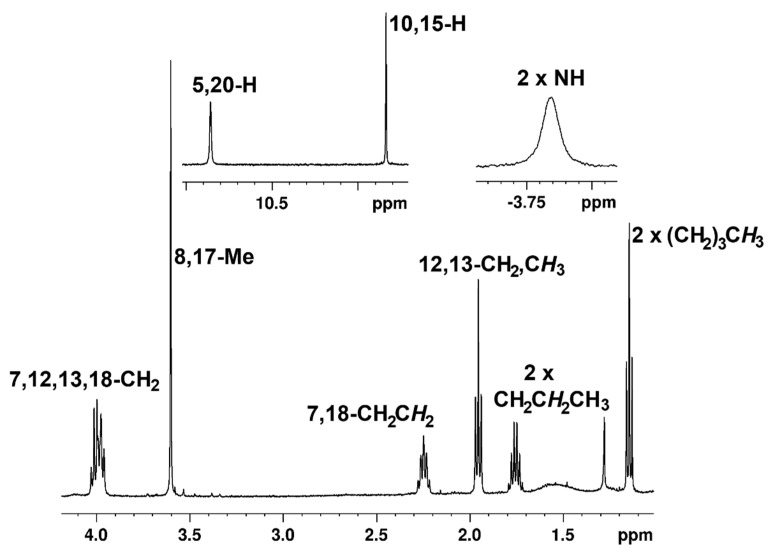
Proton NMR spectrum of bis(thiadiazolobenzoporphyrin **33** in CDCl_3_ at 55 °C.

**Figure 6:**
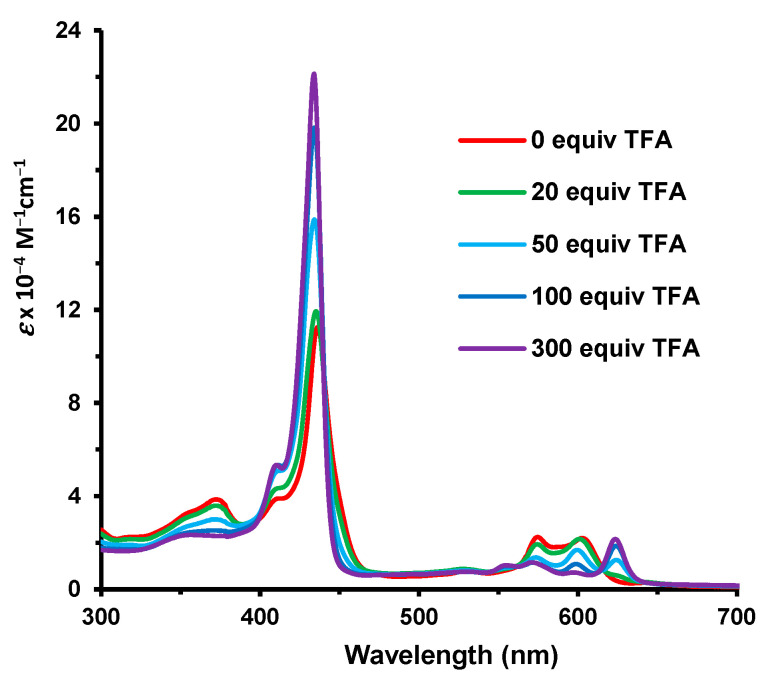
UV-vis spectra of porphyrin **33** in chloroform with 0–300 equivalents of TFA.

The presence of two butyl substituents did improve the solubility of these porphyrinoids, but it remained challenging to obtain proton NMR data for the free base structures. Protonation improved the solubility to a limited extent, but in most cases, only poor-quality ^13^C{^1^H} spectra could be obtained. The free base form of porphyrin **33** gave the best results. At 29 °C, the *meso*-proton resonances appeared downfield as two 2H singlets at 10.50 and 9.76 ppm. The methyl substituents gave rise to a 6H singlet at 3.56 ppm, and the internal NHs gave a broad peak at −4.19 ppm (Table 1). Although some minor shifts were observed at 55 °C (Table 1, Figure 5), the data in both cases showed that the global diatropic ring current for **33** is not significantly affected by the presence of the fused heterocycle. A ^13^C{^1^H} spectrum of **33** could not be obtained, but an HSQC spectrum was recorded over a period of approximately 15 h, and this showed the *meso*-carbon resonances at 101.8 and 96.2 ppm. In TFA-CDCl_3_, the *meso*-protons shifted downfield to give two 2H singlets at 12.19 and 10.78, and the methyl substituents could be identified as a 6H singlet at 3.74 ppm. These results demonstrate increased diatropicity, a phenomenon that is commonly observed for protonated porphyrins [33]. The UV-vis spectrum of **33** is significantly altered as the Soret band is shifted to 430 nm and the visible region is dominated by two Q bands at 575 and 693 nm (Figure 6). The fused heterocycle drastically reduced the basicity of the porphyrin and approximately 300 equivalents of TFA were required to monoprotonate the system. The Soret band for this species appeared at 434 nm and was greatly enhanced compared to the free base (Figure 5). At higher concentrations of TFA, only minor spectroscopic changes were noted.

**Figure 7:**
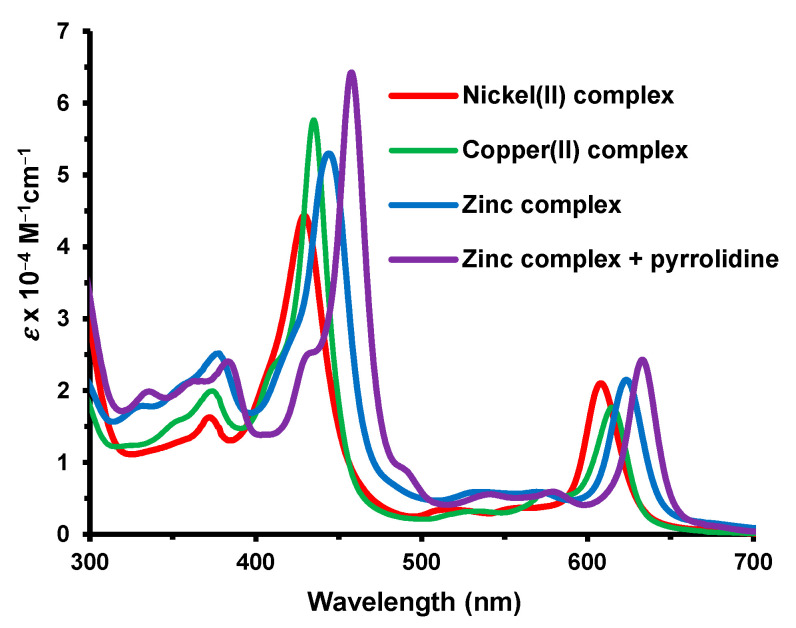
UV-vis spectra of metalated bis(thiadiazolo)benzoporphyrin.

**Scheme 4:**
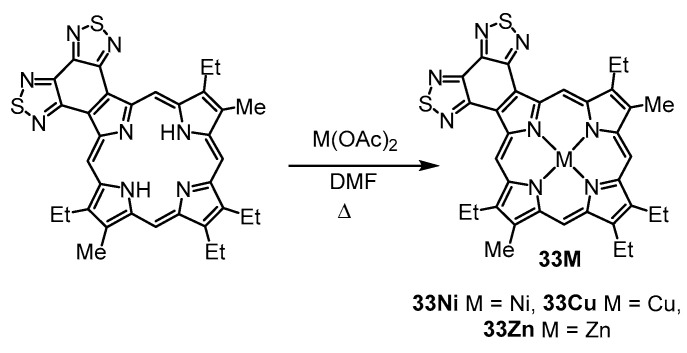
Metalation of bis(thiadiazolo)benzoporphyrin **33**.

Porphyrin **33** was reacted with nickel(II) acetate, copper(II) acetate, and zinc acetate in refluxing *N*,*N*-dimethylformamide (DMF), and the related metal complexes **33M** were isolated in 67–95% yield (Scheme 4). The UV-vis spectra for these derivatives gave rise to intense Soret bands and a strong secondary absorption above 600 nm (Figure 7). Porphyrinoids with strong absorptions in the red or far red are of interest as photosensitizers in photodynamic therapy [34,35], and while these bands are not shifted all that far into this region, they show promise. As expected, bathochromic shifts were observed with increasing atomic number for the coordinating metal cations [8,36]. **33Ni** gave a Soret band at 429 nm and a strong Q band at 608 nm, and these appeared at 435 and 615 nm for **33Cu** and 444 and 623 nm for **33Zn**. The addition of pyrrolidine to solutions of **33Zn** led to further shifts giving a Soret band at 458 nm and a long-wavelength Q band at 633 nm. Pyrrolidine coordinates to zinc porphyrins and is well known to improve solubility [37]. Copper(II) porphyrins are paramagnetic and do not give useful NMR spectra. However, **33Ni** was sufficiently soluble in CDCl_3_ at 55 °C to give a proton NMR spectrum. The *meso*-protons gave rise to two 2H singlets at 10.59 and 9.49 ppm, and the methyl substituents afforded a 6H singlet at 3.34 ppm. Zinc complex **33Zn** was far less soluble but a poor-quality NMR spectrum could be obtained at 55 °C that showed the analogous resonances at 9.75, 9.36, and 3.21 ppm. The data indicate that the nickel complex has a slightly reduced diamagnetic ring current compared to **33**. However, while this also appears to be the case for **33Zn**, the shifts may simply be due to aggregation. The addition of pyrrolidine deaggregates the complex, and this results in the *meso*-protons shifting downfield to give two 2H singlets at 11.28 and 9.80 ppm, while the methyl resonance appeared as a 6H singlet at 3.56 ppm, results that indicate retention of a powerful aromatic ring current. The results show similar trends to other metalated porphyrins [8], and the fused heterocyclic unit does not appear to significantly impact the global aromatic character for these structures.

**Figure 8:**
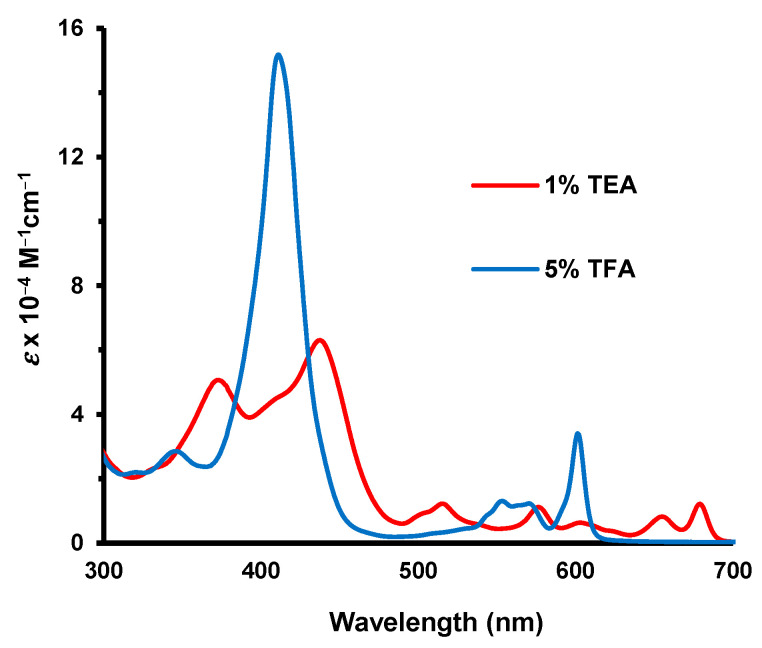
UV-vis spectra of oxaporphyrin 35a in 1% triethylamine–chloroform and in 5% TFA–chloroform.

Oxaporphyrin **35a** was isolated as a hydrochloride salt. In 1% Et_3_N-CHCl_3_, the UV-vis spectrum, which corresponded to the free base, showed two broad Soret bands at 373 and 437 nm and a series of minor absorptions in the visible region (Figure 8). In 5% TFA–chloroform, a strong Soret band emerges at 411 nm that was attributed to a monoprotonated species. A proton NMR spectrum was obtained in TFA-CDCl_3_, and this showed the *meso*-protons as two 2H singlets at 12.35 and 11.96 ppm, while the methyl groups directly attached to the macrocycle afforded a 6H singlet at 3.91 ppm. Although NMR data could not be obtained for free base **35a**, the protonated form clearly has a very strong diamagnetic ring current.

**Figure 9:**
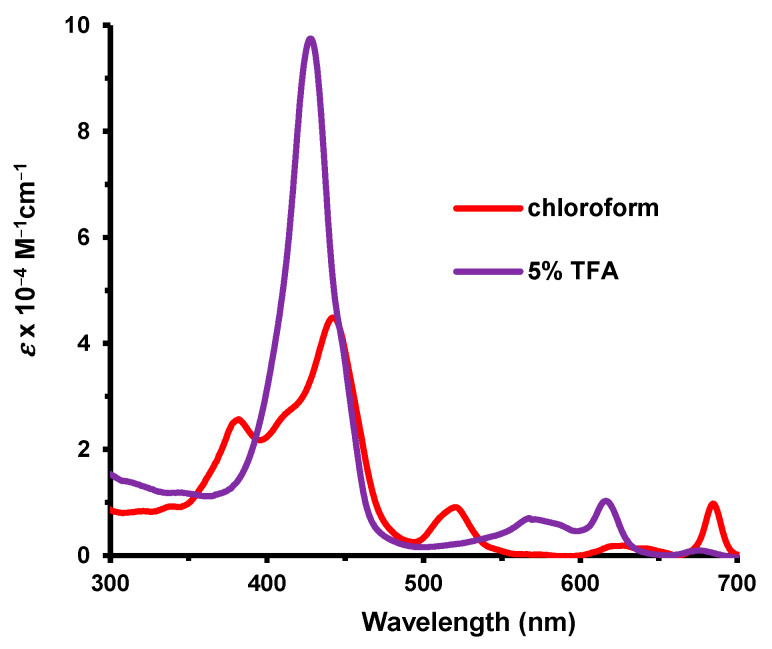
UV-vis spectra of thiaporphyrin 35b in chloroform and in 5% TFA–chloroform.

Thiaporphyrin **35b** gave a proton NMR spectrum at 50 °C that showed the *meso*-protons as two 2H singlets at 10.33 and 10.21 ppm, while the methyl groups gave a 6H singlet at 3.32 ppm (Table 1). The NH resonance was identified at −4.30 ppm. Although selenaporphyrin **35c** was isolated as an impure sample in very low yield, the corresponding resonances were observed at 10.69, 10.53, 3.33, and −3.63 ppm. These spectra show that these heteroporphyrins are strongly diatropic. When contrasting the free base forms of porphyrins, thiaporphyrins, and selenaporphyrins with fused phenanthrene, phenanthroline, and bis(thiadiazolo)benzene rings, only the phenanthroline-fused porphyrinoids show significant upfield shifts to the *meso*-protons together with the anomalous upfield shifts to the NH protons [22], indicating that the previously observed effects are not simply due to the presence of a fused strongly electron-withdrawing unit (Table 1). The addition of TFA to **35b** enhanced the ring current for the protonated form, and the *meso*-protons appeared downfield at 12.63 and 11.57 ppm. The UV-vis spectrum for **35b** gave a Soret band at 428 nm and a longer-wavelength Q band at 685 nm (Figure 9). In 5% TFA–chloroform, a strong Soret band emerged at 428 nm.

**Figure 10:**
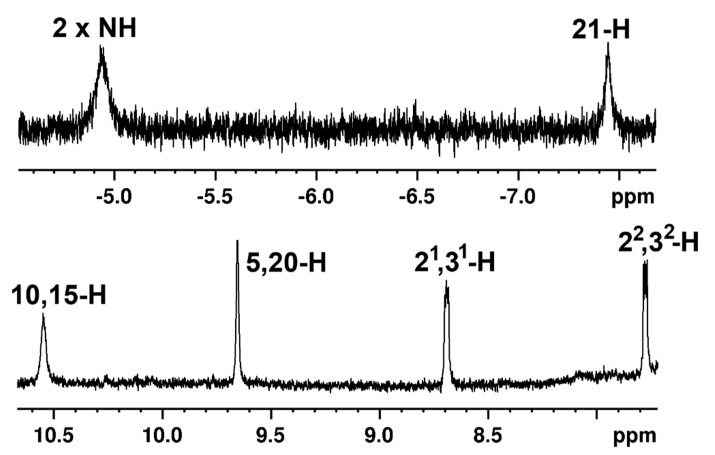
Partial proton NMR spectrum of carbaporphyrin **36** in CDCl_3_ at 55 °C showing details of the upfield and downfield regions.

**Figure 11:**
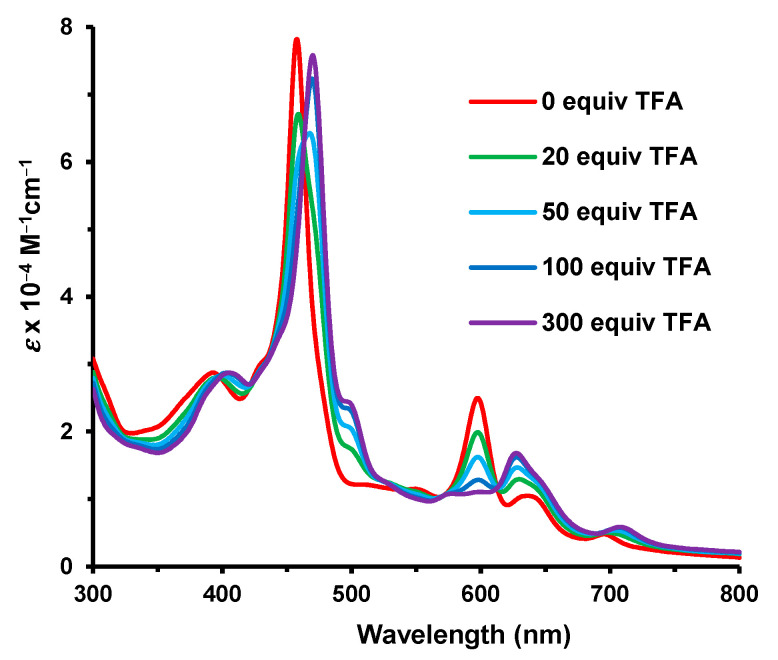
UV-vis spectra of benzocarbaporphyrin **36** in chloroform with 0–300 equivalents of TFA.

Carbaporphyrin **36** was very poorly soluble, but a low-quality proton NMR spectrum could be obtained in CDCl_3_ at 55 °C (Figure 10). The *meso*-protons appeared as two 2H singlets at 10.55 and 9.65 ppm, the methyl groups gave a peak at 3.45 ppm, and the internal NH and 21-H protons showed up at −4.93 and −7.45 ppm. Although the internal and external protons are shifted slightly upfield compared to benzocarbaporphyrins [25,38] without the fused heterocycle, the results indicate that the global aromatic properties for this structure have not been significantly altered. In the presence of trace amounts of TFA, the *meso*-protons shifted downfield to give two 2H singlets at 11.63 and 10.28 ppm, while the NH and 21-H resonances appeared at −2.57 and −6.51 ppm, respectively. These data indicate that protonation slightly enhances the aromatic properties. The UV-vis spectrum for **36** gave rise to a Soret band at 458 nm and a series of Q bands that tapered off at 700 nm (Figure 11). Protonation with TFA required >200 equivalents to complete the formation of the monoprotonated form, and this afforded a Soret band at 471 nm.

**Figure 12:**
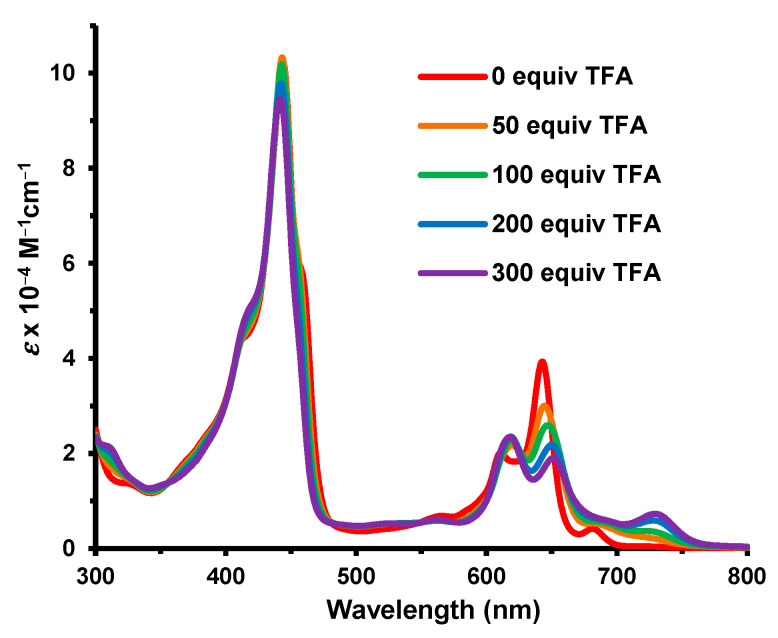
UV-vis spectra of oxypyriporphyrin **38** with 0–300 equivalents of TFA.

Oxypyriporphyrin **38** also has very low solubility, but proton NMR data could be obtained in CDCl_3_ at 55 °C. Due to the asymmetry of this system, the *meso*-protons gave rise to four 1H singlets, and these appeared at 10.89, 10.83, 10.78, and 9.39 ppm (Table 3). The inner NH protons produced two broad peaks at −3.91 and −4.01 ppm. These results again show that the bis(thiadiazolo)benzo unit does not significantly impact the aromatic properties of the porphyrinoid system, and the contradictory shifts noted for phenanthroline-fused oxypyriporphyrin **19a** are not seen for **38**. As expected, the addition of TFA led to the *meso*-proton resonances being shifted downfield to give four 1H singlets at 11.82, 11.81, 11.12, and 10.19 ppm. The UV-vis spectrum for **38** in chloroform gave a strong Soret band at 444 nm and a series of Q bands that extend to nearly 700 nm (Figure 12). Monoprotonation required the addition of ca. 300 equivalents of TFA, but further changes were observed at higher concentrations of TFA. Similar observations were made for **19a**.

**Figure 13:**
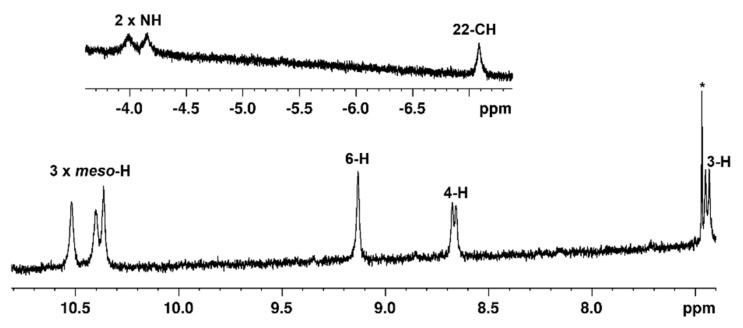
Partial proton NMR spectrum of oxybenziporphyrin 40 showing the upfield and downfield regions and the poor baseline results from the very low solubility of this porphyrinoid.

**Figure 14:**
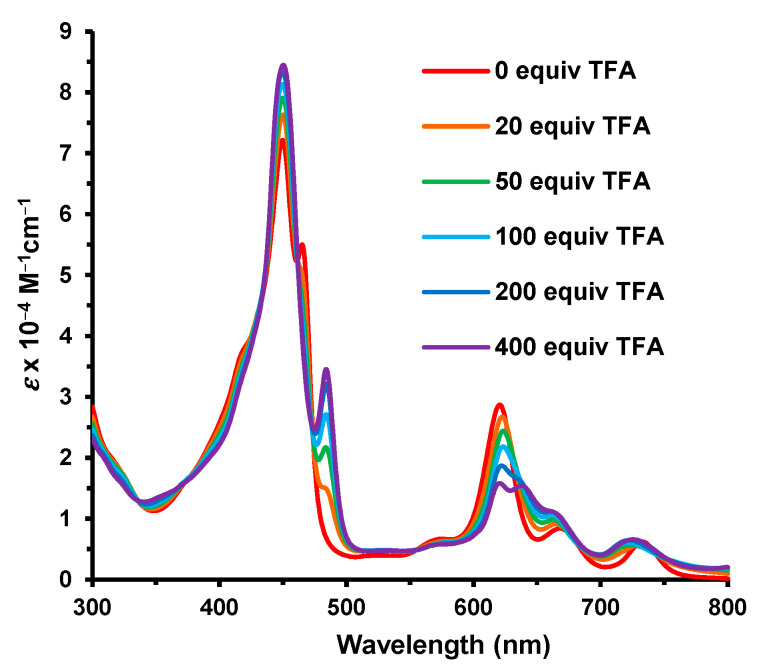
UV-vis spectra of oxybenziporphyrin 40 in chloroform with 0–400 equivalents of TFA.

Oxybenziporphyrin **40** was also rather insoluble, but a proton NMR spectrum could be obtained at 55 °C (Figure 13 and Table 3), although this required approximately 1500 transients to obtain an adequate signal-to-noise ratio. The *meso*-protons gave signals at 10.52, 10.40, 10.36, and 9.13 ppm; the methyl substituents appeared as two 3H singlets at 3.49 and 3.40 ppm; the internal NH protons gave broad peaks at −3.99 and −4.15 ppm; and the 22-H resonance showed up at −7.08 ppm. The results confirm that the macrocycle is strongly diatropic, but the fused heterocycle does not significantly perturb these results. Once again, the bis(thiadiazolo)benzo unit does not exert the effects observed for tropone-fused or phenanthroline-fused porphyrinoids. Hence, the presence of a strongly electron-withdrawing fused moiety does not in and of itself lead to the effects described for **11**–**20**. The addition of trace amounts of TFA resulted in a slight downfield shift to the *meso*-proton resonances, but this is reversed at higher concentrations of TFA. The 22-H shifted downfield towards 0 ppm upon the addition of TFA. This is attributed to protonation onto the carbonyl oxygen as this will result in the arene unit taking on phenolic character, and this interrupts the global delocalization pathways. The UV-vis spectrum of **40** in chloroform gave a Soret band at 450 nm, a secondary absorption at 465 nm, and Q bands at 621, 668, and 733 nm (Figure 14). Monoprotonation required ca. 400 equivalents of TFA and resulted in an enhancement in the Soret band. At higher concentrations of TFA, this trend was reversed.

**Scheme 5:**
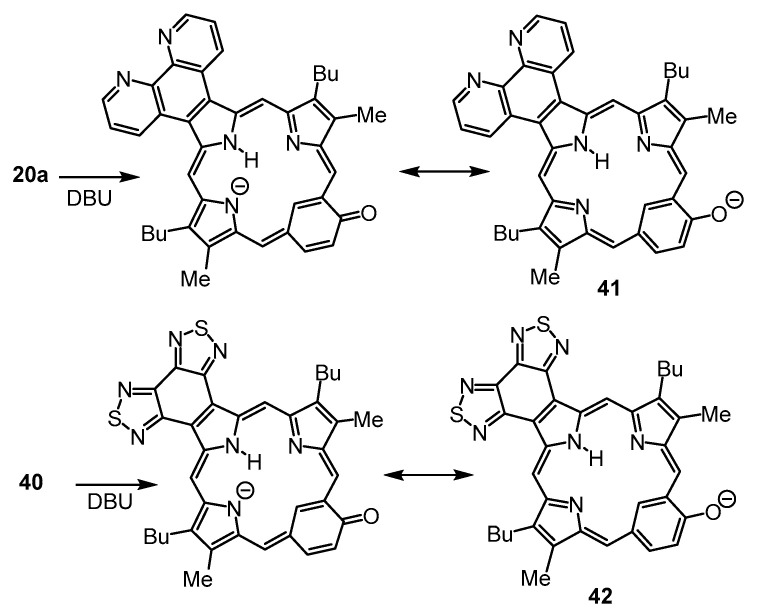
Deprotonation of oxybenziporphyrins.

**Figure 15:**
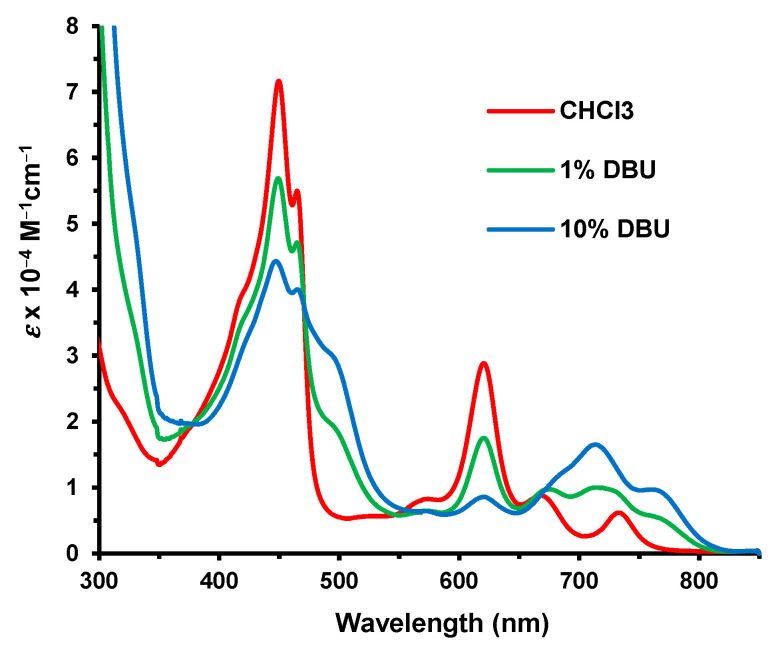
UV-vis spectra of oxybenziporphyrin **40** in chloroform, 1% DBU–chloroform, and 10% DBU–chloroform.

Phenanthrolino-oxybenziporphyrin **20a** gave rise to a new species in 1% DBU–chloroform [22]. This was attributed to deprotonation generating an anionic species **41** (Scheme 5) that is resonance-stabilized by the carbonyl moiety. We speculated that a similar phenomenon might be observed for oxybenziporphyrin **40**. Although some spectroscopic changes to the electronic absorption spectra were observed in 1% DBU–chloroform (Figure 15), even 10% DBU was insufficient to fully convert **40** into a new species (assumed to be **42**; Scheme 5). This result shows that the bis(thiadiazolo)benzo unit is far less effective in stabilizing the negative charge in this type of anionic species compared to 1,10-phenanthroline.

**Figure 16:**
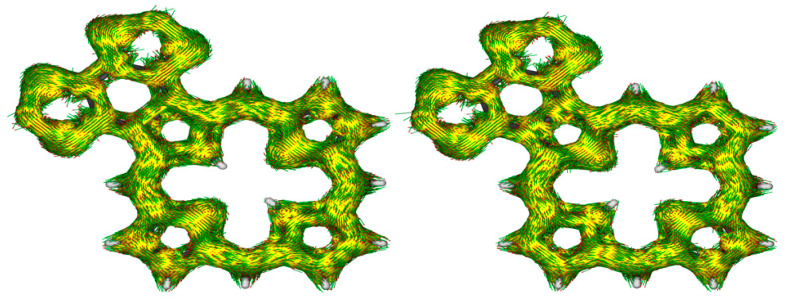
AICD plots (isovalue 0.05) of bis(thiadiazolo)benzoporphyrin tautomers **S_2_-BPa** (**left**) and **S_2_-BPb** (**right**). These demonstrate that the macrocyclic aromatic circuits are disconnected from the fused heterocycle.

Even though the 1,10-phenanthroline and bis(thiadiazolo)benzene units are both strongly electron-withdrawing, their impact on the aromatic properties of annelated porphyrinoids is quite different. In order to gain insights into these differences, density functional theory (DFT) calculations [39,40,41,42,43] were carried out. The structures were optimized using M06-2X with the triple-*ζ* basis set 6-311++G(d,p). Four tautomers of unsubstituted bis(thiadiazolo)benzoporphyrin (**S_2_-BP**) were considered, and the two forms with opposite N−H protons were shown to have the lowest energies (Table 4). Tautomer **S_2_-BPa**, which has an NH on the pyrrole that is fused to the heterocycle, is slightly less stable than **S_2_-BPb**. The aromatic character of these structures was assessed using nucleus-independent chemical shift (NICS) calculations [44]. Standard NICS calculations include effects due to *σ* and *π* electrons and are not always accurate, and in this study, NICS(0) and NICS(1)*_zz_* calculations were carried out. In NICS(1)*_zz_*, the calculations were performed 1 Å above the ring, and the results provide a more accurate measure of the diatropic ring currents. Negative values correspond to aromatic species, while positive values are obtained for antiaromatic systems. Positive values may also be observed when the NICS values are measured at points that are external to the aromatic delocalization pathways. All four tautomers gave strongly aromatic NICS(0) and NICS(1)*_zz_* values (Table 4). The NICS*_zz_* values are much larger than those for standard NICS calculations, but otherwise, the observed trends were similar. The porphyrinoid structures were also assessed using anisotropy of induced current density (AICD) [45]. The AICD plots for **S_2_-BPa and S_2_-BPb** show the presence of 18*π*-electron circuits within the macrocycle, and the heterocyclic unit appears to be totally disconnected, showing no significant interactions (Figure 16). This differs from the results for phenanthroline-fused porphyrins as the AICD plot for the tautomer analogous to **S_2_-BPa** shows the presence of a significant 30*π-*electron diatropic circuit that extends around the periphery of the fused phenanthroline unit.

**Figure 17:**
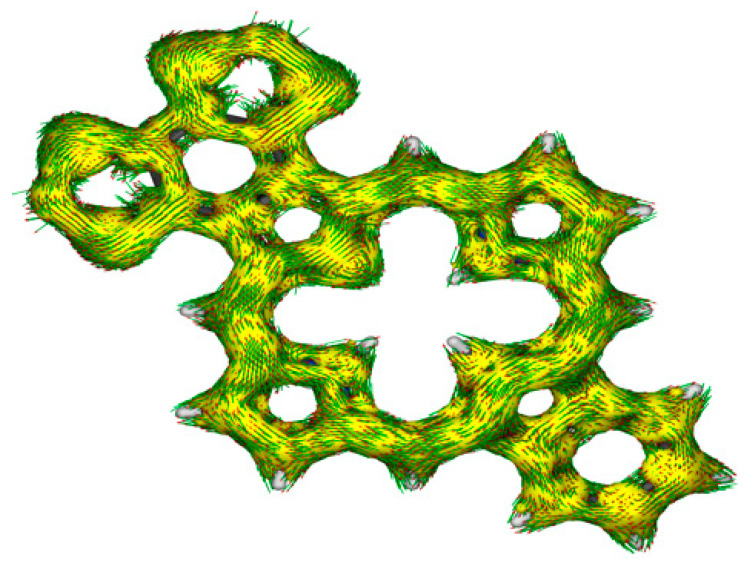
AICD plot (isovalue 0.05) of bis(thiadiazolo)benzocarbaporphyrin **S_2_-BCBPa**.

**Figure 18:**
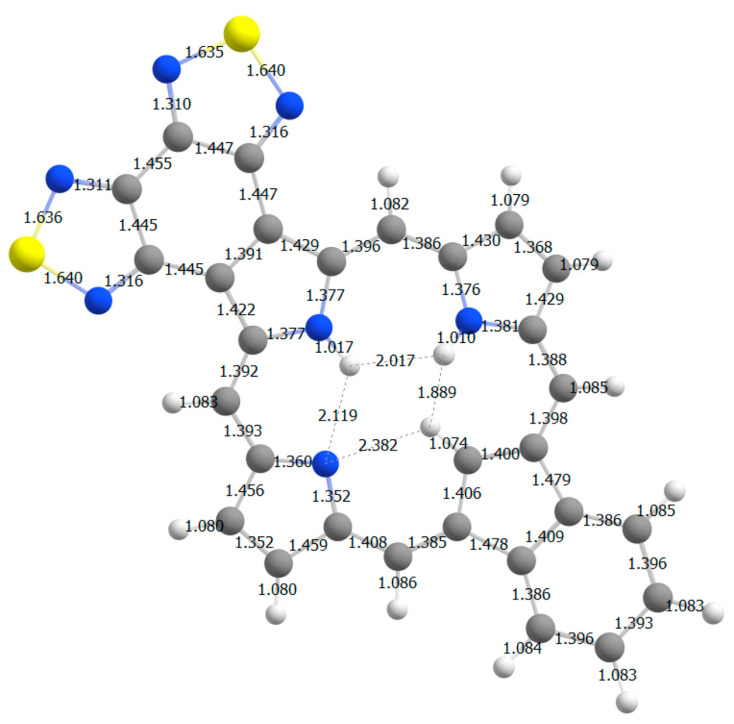
Calculated bond lengths for bis(thiadiazolo)benzene-fused carbaporphyrin **S2-BCBPa** showing relatively long carbon–carbon bonds between the porphyrin nucleus and the fused aromatic rings.

NICS and NICS*_zz_* calculations for unsubstituted oxaporphyrin **S_2_-OxBP**, thiaporphyrin **S_2_-BTP**, and selenaporphyrin **S_2_-BSP** all show strongly aromatic values for the two most likely tautomers, although structures with the NH opposite to the chalconide atom are favored (Table 5). The AICD plots again showed that the fused heterocycle’s *π*-system does not interact with the conventional porphyrin-type 18*π*-electron pathways. In contrast, heteroporphyrins with fused phenanthroline units show an extended 30*π* aromatic circuit through the phenanthroline [22]. Four tautomers of unsubstituted bis(thiadiazolobenzo)carbaporphyrin (**S_2_-BCBP**) were considered, all of which have 18*π*-electron delocalization pathways (Table 6). Tautomers **S_2_-BCBPc** and **S_2_-BCBPd** with internal methylene units are far higher in energy, and **S_2_-BCBPa** with opposite NHs is favored. NICS and NICS*_zz_* values for **S_2_-BCBPa** show that the macrocycle is strongly aromatic. Ring g gives a low positive value indicating that it lies outside of the aromatic pathways, a feature that is seen in the calculations for all of the bis(thiadiazolobenzo)porphyrinoids. The AICD plot for **S_2_-BCBPa** (Figure 17) reinforces this interpretation, once again showing that the fused heterocycle does not facilitate extended aromatic conjugation pathways. However, this type of extended aromatic circuit is seen in the AICD plot for the analogous phenanthroline-fused carbaporphyrin [22]. A bond length analysis of **S_2_-BCBPa** is also consistent with this conclusion (Figure 18). The bonds connecting the thiadiazole units to the porphyrinoid macrocycle are relatively long, as is the case for the bonds connected to the benzo-unit, indicating that both of the fused rings are only weakly interacting with the aromatic *π*-system.

Five tautomers of unsubstituted pyriporphyrin **S_2_-OPBP** were considered (Table 7). Hydroxypyridine tautomer **S_2_-OPBPd** is much less stable, but an unconventional tautomer **S_2_-OPBPe** bearing an NH on the pyridine moiety is only ca. 5 kcal less stable than the favored tautomer **S_2_-OPBPa**, suggesting that this structural arrangement should be easily accessible. Otherwise, as expected, the tautomer with opposite NHs on the pyrrole subunits is the most stable form. NICS and NICS*_zz_* calculations show that all five tautomers are fully aromatic. The AICD plot for **S_2_-OPBPa** shows that the fused heterocycle is disconnected from the macrocycle’s *π*-system even though the related phenanthroline-fused system shows the presence of an extended 30*π*-electron pathway. Four tautomers of oxybenziporphyrin **S_2_-OBBP** were also investigated (Table 8). Phenolic tautomer **S_2_-OBBPd** is the least stable for this set, and semiquinone tautomer **S_2_-OBBPa** with opposite NHs is the most stable form. NICS and NICS*_zz_* calculations show that tautomer **S_2_-OBBPa** is at most weakly aromatic, but the remaining tautomers exhibit strongly diatropic properties. For **S_2_-OBBPa**, rings c and g have low positive values, indicating that they lie outside of the global aromatic circuit. AICD plots also show the presence of an 18*π*-electron aromatic pathway that does not interact to any extent with the fused heterocycle. The analogous phenanthroline-fused oxybenziporphyrin possesses a 30*π*-electron circuit, as is the case for the other phenanthrolinoporphyrinoids discussed in this paper.

The results indicate that the anomalous proton NMR spectra observed for phenanthroline-fused porphyrinoids are triggered by the presence of extended conjugation pathways through the phenanthroline moiety. Extended aromatic circuits do not contribute to the delocalization pathways in porphyrinoids with fused bis(thiadiazolo)benzene units, and this, presumably, is the reason that these two series are so different from one another. While the results for the bis(thiadiazolo)benzene-fused porphyrinoids are self-consistent and the computational data are in good agreement with the experimental results, further studies will be needed to explain the anomalous results for tropone-fused and phenanthroline-fused porphyrinoids.

## 3. Experiments

Chemicals and solvents were purchased from Fisher (Pittsburgh, PA, USA) or Sigma-Aldrich (Burlington, MA, USA). Melting points were uncorrected. NMR spectra were recorded using a 400 or 500 MHz NMR spectrometer (Bruker, Billerica, MA, USA) and were recorded at 302 K unless otherwise indicated. ^1^H NMR values are reported as chemical shifts δ, relative integral, multiplicity (s, singlet; d, doublet; t, triplet; q, quartet; m, multiplet; br, broad peak) and coupling constant (*J*). Chemical shifts are reported in parts per million (ppm) relative to CDCl_3_ (^1^H residual CHCl_3_ singlet δ 7.26 ppm, ^13^C CDCl_3_ triplet δ 77.23 ppm), and coupling constants were taken directly from the spectra. NMR assignments were made with the aid of ^1^H-^1^H COSY, HSQC, DEPT-135, and nOe difference proton NMR spectroscopy. Two-dimensional NMR experiments were performed using standard software. Mass spectral data were acquired using positive-mode electrospray ionization (ESI^+^) and a high-resolution time-of-flight mass spectrometer (Agilent, Santa Clara, CA, USA).

**Tripyrrane 32.** *N*-Chlorosuccinimide (0.350 g. 2.6 mmol) was added to a solution of *tert*-butyl 4-butyl-3,5-dimethyl-pyrrole-2-carboxylate (**30**) [20] (0.650 g, 2.59 mmol) in dichloromethane (50 mL), and the mixture was stirred at room temperature overnight. The solution was washed with water (3 × 50 mL) and evaporated under reduced pressure to give crude chloromethylpyrrole **31** as a yellow-brown oil. The residue was taken up in pyridine (20 mL) and stirred under nitrogen at room temperature for 1 h. Bis-thiadiazolobenzo[4,5-*c*]pyrrole **23** [27] (200 mg, 0.858 mmol) was then added, and the mixture was refluxed with stirring under a nitrogen atmosphere for 16 h. The solution was cooled, diluted with dichloromethane, and washed with water. The organic layer was evaporated, and the residue was purified on a silica column, eluting with 1% triethylamine–dichloromethane, giving the crude tripyrrane as a brown solid (138 mg, 0.188 mmol, 22%). ^1^H NMR (500 MHz, CDCl_3_): δ 9.22 (br s, 2H, 2 × NH), 8.58 (br s, 1H, NH), 4.45 (s, 4H, 2 × bridge-CH_2_), 2.38 (t, 4H, *J* = 7.5 Hz, 2 × pyrrole-CH_2_), 2.23 (s, 6H, 2 × pyrrole-CH_3_), 1.39–1.26 (m, 8H), 1.41 (s, 18H, 2 × *t*-Bu), 0.86 (t, 6H, *J* = 7.0 Hz, 2 × (CH_2_)_3_C*H*_3_). ^13^C{^1^H} NMR (CDCl_3_): δ 161.4, 154.3, 148.8, 128.4, 127.3, 126.4, 122.9, 119.5, 111.5, 80.5, 33.7, 28.7, 24.0, 23.9, 22.8, 14.1, 10.8. HRMS (ESI): *m/z* [M + H]^+^ calcd for C_38_H_50_N_7_O_4_S_2_ 732.3360; found 732.3361.

**7,18-Dibutyl-12,13-diethyl-8,17-dimethylbis(thiadiazolo)benzo[4,5-*c*]porphyrin** (**33**). The foregoing crude tripyrrane **32** (50.0 mg, 0.068 mmol) was dissolved in trifluoroacetic acid (1 mL) and stirred at room temperature for 7 min under nitrogen. The mixture was diluted with dichloromethane (20 mL); this was followed by the addition of pyrroledialdehyde **28** (12.2 mg, 0.068 mmol). The mixture was stirred under nitrogen at room temperature for an additional 2 h. The dark solution was diluted with chloroform and vigorously shaken with 0.15% aqueous ferric chloride solution (100 mL) for 5–7 min. The organic solution was washed with water, 5% sodium bicarbonate solution, and water, and the solvent was evaporated under reduced pressure. The residue was purified on a grade 3 alumina column, eluting with dichloromethane. A deep-green fraction was collected and recrystallized from chloroform–methanol to give the title porphyrin (16.6 mg, 0.0247 mmol, 36%) as deep purple crystals, mp > 260 °C. UV-vis (CHCl_3_): λ_max_/nm (log ε) 372 (4.58), 410 (sh, 4.59), 436 (5.05), 530 (3.89), 575 (4.35), 603 (4.34), 642 (3,45). UV-vis (300 eq TFA-CHCl_3_): λ_max_/nm (log ε) 410 (4.73), 434 (5.34), 554 (4.00), 571 (4.06), 598 (3.86), 623 (4.33). UV-vis (1% TFA-CHCl_3_): λ_max_/nm (log ε) 353 (4.00), 409 (4.33), 433 (5.37), 570 (4.05), 622 (4.33). UV-vis (5% TFA-CHCl_3_): λ_max_/nm (log ε) 408 (4.75), 431 (5.39), 569 (4.05), 622 (3.31). ^1^H NMR (500 MHz, CDCl_3_, 32 °C): δ 10.50 (s, 2H, 5,20-H), 9.76 (s, 2H, 10,15-H), 4.00 (q, 4H, *J* = 7.7 Hz, 12,13-CH_2_), 3.85 (t, 4H, *J* = 7.8 Hz, 7,18-CH_2_), 3.56 (s, 6H, 8,17-Me), 2.15 (pentet, 4H, *J* = 7.5 Hz, 7,19-CH_2_C*H*_2_), 1.95 (t, 6H, *J* = 7.7 Hz, 12,13-CH_2_C*H*_3_), 1.68 (sextet, 4H, *J* = 7.4 Hz, 2 × CH_2_C*H*_2_CH_3_), 1.09 (t, 6H, *J* = 7.4 Hz, 2 × (CH_2_)_3_C*H*_3_), −4.19 (br s, 2H, 2 × NH). ^1^H NMR (500 MHz, CDCl_3_, 55 °C): δ 10.86 (s, 2H, 5,20-H), 9.85 (s, 2H, 10,15-H), 4.03–3.96 (m, 8H, 7,12,13,18-CH_2_), 3.60 (s, 6H, 8,17-Me), 2.25 (pentet, 4H, *J* = 7.5 Hz, 7,19-CH_2_C*H*_2_), 1.95 (t, 6H, *J* = 7.7 Hz, 12,13-CH_2_C*H*_3_), 1.75 (sextet, 4H, *J* = 7.4 Hz, 2 × CH_2_C*H*_2_CH_3_), 114 (t, 6H, *J* = 7.4 Hz, 2 × (CH_2_)_3_C*H*_3_), −3.77 (br s, 2H, 2 × NH). Partial ^13^C NMR derived from HSQC spectrum (CDCl_3_, 55 °C): δ 101.8 (5,20-CH), 96.2 (10.15-CH), 34.8 (7,18-CH_2_*C*H_2_), 26.0 (7,18-CH_2_), 23.0 (2 × CH_2_*C*H_2_CH_3_), 19.8 (12,13-CH_2_), 18.2 (12,13-CH_2_C*H*_3_), 14.0 (2 × (CH_2_)_3_C*H*_3_), 11.3 (8,17-Me). ^1^H NMR (500 MHz, TFA-CDCl_3_): δ 12.19 (s, 2H, 5,20-H), 10.76 (s, 2H, 10,15-H), 4.31 (t, 4H, *J* = 7.8 Hz, 7,18-CH_2_), 4.20 (q, 4H, *J* = 7.7 Hz, 12,13-CH_2_), 3.74 (s, 6H, 8,17-CH_3_), 2.31–2.35 (m, 4H, 8,17-CH_2_C*H*_2_), 1.84–1.74 (m, 10H, 12,13-CH_2_C*H*_3_ and 7,18-CH_2_CH_2_C*H*_2_), 1.15 (t, *J* = 7.4 Hz, 7,18-(CH_2_)_3_C*H*_3_), −3.32 (br s), −3.37 (br s) (3H). ^13^C{^1^H} NMR (TFA-CDCl_3_): δ 152.6, 150.3, 146.3, 145.8, 144.8, 143.8, 143.5, 139.8, 136.4, 126.4, 103.2 (5,20-CH), 99.8 (10,15-CH), 34.7 (7,18-CH_2_*C*H_2_), 27.1 (7,18-CH_2_), 23.4 (2 × CH_2_*C*H_2_CH_3_), 20.2 (12,13-CH_2_), 17.3 (12,13-CH_2_*C*H_3_), 13.8 (2 × (CH_2_)_3_C*H*_3_), 12.1 (8,17-Me). HRMS (ESI+): *m/z* [M + H]^+^ calcd for C_38_H_41_N_8_S_2_: 673.2890; found 673.2875.

Metal complexes of **33** were prepared by refluxing the porphyrin (9.0 mg, 0.0134 mmol) with 20 mg of a zinc, copper(II), or nickel(II) acetate in DMF (10 mL) for 3–16 h. After diluting the mixture with chloroform, washing with water, and evaporating the solvent under reduced pressure, the residues were recrystallized from chloroform–methanol to give purple crystals. **33Ni**: 6.6 mg, 0.0090 mmol, 67%, mp > 260 °C. UV-vis (CHCl_3_): λ_max_/nm (log ε) 372 (4.21), 429 (4.65), 608 (4.32). ^1^H NMR (500 MHz, CDCl_3_, 55 °C): δ 10.59 (s, 2H, 5,20-H), 9.49 (s, 2H, 10,15-H), 3.91–3.86 (m, 4H, 12,13-CH_2_), 3.72–3.68 (m, 4H, 7,18-CH_2_), 3.34 (s, 6H, 8,17-Me), 2.14–2.07 (m, 4H, 7,19-CH_2_C*H*_2_), 1.85 (t, 6H, *J* = 7.5 Hz, 12,13-CH_2_C*H*_3_), 1.72–1.68 (m, 4H, 2 × CH_2_C*H*_2_CH_3_), 1.11 (t, 6H, *J* = 7.4 Hz, 2 × (CH_2_)_3_C*H*_3_). HRMS (EI): *m/z* M^+^ calcd for C_38_H_38_N_8_NiS_2_ 728.2014; found 728.2020. **33Cu**: 9.4 mg, 0.0128 mg, 95%, mp > 260 °C. UV-vis (CHCl_3_): λ_max_ (log ε) 374 (4.30), 435 (4.76), 574 (3.77), 615 (4.25). HRMS (MALDI): *m/z* M^+^ calcd for C_38_H_38_CuN_8_S_2_ 733.1957; found 733.1940. **33Zn**: 7.3 mg, 0.0099 mmol, 74%, mp > 260 °C. UV-vis (CHCl_3_): λ_max_/nm (log ε) 378 (4.40), 444 (4.72), 534 (sh, 3.77), 569 (3.77), 623 (4.33). UV-vis (1% pyrrolidine-CHCl_3_): λ_max_/nm (log ε) 383 (4.38), 432 (sh, 4.40), 458 (4.81), 540 (3.75), 580 (3.77), 633 (4.38). ^1^H NMR (500 MHz, CDCl_3_, 55 °C, *meso*-protons only): δ 9.75 (br s, 2H, 5,20-H), 9.36 (s, 2H, 10,15-H). ^1^H NMR (500 MHz, pyrrolidine-CDCl_3_): δ 11.28 (s, 2H, 5,20-H), 9.80 (s, 2H, 10,15-H), 4.09 (t, 4H, *J* = 7.5 Hz, 7,18-CH_2_), 4.02 (q, 4H, *J* = 7.5 Hz, 12,13-CH_2_), 3.56 (s, 6H, 8,17-Me), 2.36–2.30 (m, 4H, 7,19-CH_2_C*H*_2_), 1.89 (t, 6H, obscured by pyrrolidine, 12,13-CH_2_C*H*_3_), 1.53–1.47 (m, 4H, 2 × CH_2_C*H*_2_CH_3_), 1.16 (t, 6H, *J* = 7.5 Hz, 2 × (CH_2_)_3_C*H*_3_). HRMS (ESI): *m/z* [M + H]^+^ calcd for C_38_H_39_N_8_S_2_Zn 735.2025; found 735.2037.

**8,17-Dibutyl-7,18-dimethyl-benzo[*b*]bis(thiadiazolo)benzo[4,5-*l*]-21-carbaporphyrin** (**36**). Tripyrrane **32** (50.0 mg, 0.068 mmol) was dissolved in trifluoroacetic acid (1 mL) and stirred at room temperature for 7 min under nitrogen. The mixture was diluted with dichloromethane (20 mL); this was followed by the addition of indenedialdehyde **29** (11.7 mg, 0.068 mmol). The mixture was stirred under nitrogen at room temperature for an additional 2 h. The dark solution was diluted with chloroform and vigorously shaken with 0.15% aqueous ferric chloride solution (100 mL) for 5–7 min. The organic solution was washed with water, 5% sodium bicarbonate solution, and water, and the solvent evaporated under reduced pressure. The residue was purified on a grade 3 alumina column, eluting with dichloromethane. Recrystallization from chloroform–methanol gave the carbaporphyrin (11.0 mg, 0.0165 mmol, 24%) as a dark solid, mp > 260 °C. UV-vis (CHCl_3_): λ_max_/nm (log ε) 393 (4.46), 458 (4.89), 597 (4.40), 635 (4.02), 696 (3.68). UV-vis (1% TFA-CHCl_3_): λ_max_/nm (log ε) 411 (4.43), 471 (4.83), 500 (sh, 4.36), 629 (4.21), 708 (3.79). ^1^H NMR (500 MHz, CDCl_3_, 55 °C, partial data): δ 10.55 (s, 2H, 5,20-H), 9.65 (s, 2H, 10,15-H), 8.70–8.68 (m, 2H, 2^1^,3^1^-H), 7.79–7.77 (m, 2H, 2^2^,3^2^-H), 3.81 (br t, 4H), 3.45 (s, 6H), −4.93 (br s, 2H, 2 × NH), −7.45 (s, 1H, 21-H). ^1^H NMR (500 MHz, 5 mL TFA-CDCl_3_): δ 11.63 (s, 2H, 5,20-H), 10.28 (s, 2H, 10,15-H), 8.65–8.63 (m, 2H, 2^1^,3^1^-H), 7.72–7.70 (m, 2H, 2^2^,3^2^-H), 4.18 (t, 4H, *J* = 7.4 Hz, 8,17-CH_2_), 3,59 (s, 6H, 7,18-Me), 2.26–2.19 (m, 4H, 8,17-CH_2_C*H*_2_), 1.79–1.72 (m, 4H, 2 × CH_2_C*H*_2_CH_3_), 1.14 (t, 6H, *J* = 7.2 Hz, 2 × (CH_2_)_3_C*H*_3_), −2.57 (s, 2H, 2 × NH), −6.51 (s, 1H, 21-H). ^13^C{^1^H} NMR (5 mL TFA-CDCl_3_, 125 MHz): δ 143.8, 142.3, 142.1, 139.6, 136.1, 128.9, 121.9, 105.2, 99.1, 34.7, 26.6, 23.3, 14.2, 12.0. HRMS (ESI): *m/z* [M + H]^+^ calcd for C_39_H_36_N_7_S_2_ 666.2468; found 666.2472.

**8,17-Dibutyl-7,18-dimethyl-bis(thiadiazolo)benzo[4,5-*l*]-21-oxaporphyrin hydrochloride** (**35a.HCl**). Tripyrrane **32** (50.0 mg, 0.068 mmol) was dissolved in trifluoroacetic acid (1 mL) and stirred at room temperature for 7 min under nitrogen. The mixture was diluted with dichloromethane (20 mL); this was followed by the addition of furandialdehyde **34a** (8.4 mg, 0.068 mmol). The mixture was stirred under nitrogen at room temperature for an additional 2 h. The dark solution was diluted with chloroform and vigorously shaken with 0.15% aqueous ferric chloride solution (100 mL) for 5–7 min. The organic solution was washed with water, 5% sodium bicarbonate solution, water, and 10% hydrochloric acid, and the solvent evaporated under reduced pressure. The residue was purified on a silica column, eluting with chloroform and then 5% methanol–chloroform. Recrystallization from chloroform-hexanes gave the title compound (9.0 mg 0.0137 mmol, 20%) as a dark solid, mp > 260 °C. UV-vis (1% Et_3_N-CHCl_3_): λ_max_/nm (log ε) 373 (4.70), 437 (4.80), 515 (4.09), 576 (4.05), 603 (3.80), 655 (3.91), 679 (4.08). UV-vis (CHCl_3_): λ_max_/nm (log ε) 348 (4.43), 413 (5.13), 554 (4.05), 570 (sh, 4.01), 602 (4.45), 645 (2.87), UV-vis (5% TFA-CHCl_3_): λ_max_/nm (log ε) 345 (4.45), 411 (5.18), 470 (sh, 4.09), 553 (4.12), 570 (4.09), 601 (4.53). ^1^H NMR (500 MHz, TFA-CDCl_3_): δ 12.35 (s, 2H, 5,20-H), 11.06 (s, 2H, 10,15-H), 10.57 (s, 2H, 2,3-H), 4.45 (t, 4H, *J* = 7.8 Hz, 8,17-CH_2_), 3,91 (s, 6H, 7,18-Me), 2.52–2.46 (m, 4H, 8,17-CH_2_C*H*_2_), 1.96–1.89 (m, 4H, 2 × CH_2_C*H*_2_CH_3_), 1.26 (t, 6H, *J* = 7.4 Hz, 2 × (CH_2_)_3_C*H*_3_), −4.7 (v br, 2H). ^13^C{^1^H} NMR (TFA-CDCl_3_): δ 154.6, 153.0, 150.0, 146.2, 141.4, 141.3, 139.7, 132.6), 107.9, 97.8, 35.4, 26.9, 23.5, 14.2, 12.1. HRMS (ESI): *m/z* [M + H]^+^ calcd for C_34_H_32_N_7_OS_2_ 618.2104; found 618.2096.

**8,17-Dibutyl-7,18-dimethyl-bis(thiadiazolo)benzo[4,5-*l*]-21-thiaporphyrin** (**35b**). Tripyrrane **32** (50.0 mg, 0.068 mmol) was dissolved in trifluoroacetic acid (1 mL) and stirred at room temperature for 7 min under nitrogen. The mixture was diluted with dichloromethane (20 mL); this was followed by the addition of 2,5-thiophenedicarbaldehyde **34b** (9.5 mg; 0.068 mmol). The mixture was stirred under nitrogen at room temperature for an additional 2 h. The dark solution was diluted with chloroform and vigorously shaken with 0.15% aqueous ferric chloride solution (100 mL) for 5–7 min. The organic solution was washed with water, 5% sodium bicarbonate solution, and water, and the solvent evaporated under reduced pressure. The residue was purified on a grade 3 alumina column, eluting with dichloromethane. Recrystallization from chloroform–methanol gave the thiaporphyrin (3.6 mg 0.0057 mmol, 8.3%) as purple crystals, mp > 260 °C. UV-vis (CHCl_3_): λ_max_/nm (log ε) 382 (4.41), 442 (4.65), 520 (3.96), 628 (3.28), 685 (3.99). UV-vis (1% TFA-CHCl_3_): λ_max_/nm (log ε) 428 (4.96), 571 (3.76), 616 (4.00). ^1^H NMR (500 MHz, CDCl_3_, 50 °C): δ 10.33 (s, 2H, 5,20-H), 10.21 (s, 2H, 10,15-H), 9.94 (s, 2H, 2,3-H), 3.52 (t, 4H, *J* = 7.8 Hz, 8,17-CH_2_), 3,32 (s, 6H, 7,18-Me), 2.09–2.03 (m, 4H, 8,17-CH_2_C*H*_2_), 1.71–1.64 (m, 4H, 2 × CH_2_C*H*_2_CH_3_), 1.08 (t, 6H, *J* = 7.3 Hz, 2 × (CH_2_)_3_C*H*_3_), −4.30 (br s, 1H, NH). ^1^H NMR (500 MHz, TFA-CDCl_3_): δ 12.63 (s, 2H, 5,20-H), 11.57 (s, 2H, 10,15-H), 10.61 (s, 2H, 2,3-H), 4.45 (t, 4H, *J* = 7.8 Hz, 8,17-CH_2_), 3,91 (s, 6H, 7,18-Me), 2.56–2.52 (m, 4H, 8,17-CH_2_C*H*_2_), 1.98–1.90 (m, 4H, 2 × CH_2_C*H*_2_CH_3_), 1.26 (t, 6H, *J* = 7.2 Hz, 2 × (CH_2_)_3_C*H*_3_). ^13^C{^1^H} NMR (TFA-CDCl_3_): δ 153.0, 150.1, 146.4, 145.6, 140.9, 137.8, 111.6, 108.5, 35.2, 26.7, 23.4, 14.2, 12.1. HRMS (ESI): *m/z* [M + H]^+^ calcd for C_34_H_31_N_7_S_3_ 634.1876; found 634.1876.

**9,18-Dibutyl-8,19-dimethyl-bis(thiadiazolo)benzo[4,5-*m*]oxypyriporphyrin** (**38**). Tripyrrane **32** (50.0 mg, 0.068 mmol) was dissolved in trifluoroacetic acid (1 mL) and stirred at room temperature for 7 min under nitrogen. The mixture was diluted with dichloromethane (20 mL); this was followed by the addition of 3-hydroxy-2,6-pyridinedicarbaldehyde **37** (10.3 mg, 0.068 mmol). The mixture was stirred under nitrogen at room temperature for an additional 2 h. The dark solution was diluted with chloroform and vigorously shaken with 0.15% aqueous ferric chloride solution (100 mL) for 5–7 min. The organic solution was washed with water, 5% sodium bicarbonate solution, and water, and the solvent evaporated under reduced pressure. The residue was purified on a grade 3 alumina column, eluting with chloroform. Recrystallization from chloroform–methanol gave the oxypyriporphyrin (14.1 mg. 0.0219 mmol, 32%) as purple crystals, mp > 260 °C. UV-vis (CHCl_3_): λ_max_/nm (log ε) 412 (sh, 4.64), 444 (5.01), 459 (sh, 4.76), 611 (4.30), 643 (4.59), 681 (3.62). UV-vis (200 equivalents TFA-CHCl_3_): λ_max_/nm (log ε) 413 (sh, 4.66), 442 (4.99), 619 (4.37), 650 (4.34), 727 (3.77). UV-vis (1% TFA-CHCl_3_): λ_max_/nm (log ε) 437 (4.92), 448 (4.97), 610 (4.33), 672 (4.14), 736 (4.03). UV-vis (5% TFA-CHCl_3_): λ_max_/nm (log ε) 450 (5.30), 580 (3.98), 612 (3.97), 675 (4.38). ^1^H NMR (500 MHz, CDCl_3_, 55 °C, partial data): δ 10.90 (s, 1H), 10.84 (s, 1H), 10.78 (s, 1H), 9.39 (s, 1H), 9.21–9.19 (m, 1H), 7.89 (d, 1H, *J* = 8.5 Hz), −3.91–−4.01 (br, 2H). ^1^H NMR (500 MHz, TFA-CDCl_3_): δ 11.82 (s, 1H), 11.81 (s, 1H), 11.12 (s, 1H), 10.19 (s, 1H), 9.86 (d, 1H, *J* = 9.7 Hz), 8.72 (d, 1H, *J* = 9.7 Hz), 4.14 (t, 4H, *J* = 7.8 Hz, 9,18-CH_2_), 3,60 (s, 3H), 3.56 (s, 3H) (8,19-Me), 2.22–2.16 (m, 4H, 9,18-CH_2_C*H*_2_), 1.76–1.68 (m, 4H, 2 × CH_2_C*H*_2_CH_3_), 1.13–1.10 (2 overlapping triplets, 6H, 2 × (CH_2_)_3_C*H*_3_). ^13^C{^1^H} NMR (TFA-CDCl_3_, 125 MHz): δ 151.9, 150.4, 146.4, 146.2, 145.3, 144.5, 143.6, 142.2, 141.1, 136.3, 134.7, 134.1, 107.6, 105.0, 104.7, 103.6, 34.5, 26.7, 23.2, 13.9, 12.2, 12.0. HRMS (ESI): *m/z* [M + H]^+^ calcd for C_39_H_33_N_8_OS_2_ 645.2213; found 645.2236.

**9,18-Dibutyl-8,19-dimethyl-bis(thiadiazolo)benzo[4,5-*m*]oxybenziporphyrin** (**40**). Tripyrrane **32** (50.0 mg, 0.068 mmol) was dissolved in trifluoroacetic acid (1 mL) and stirred at room temperature for 7 min under nitrogen. The mixture was diluted with dichloromethane (20 mL); this was followed by the addition of 4-hydroxyisophthalaldehyde **39** (10.2 mg, 0.068 mmol). The mixture was stirred under nitrogen at room temperature for an additional 2 h. The dark solution was diluted with chloroform and vigorously shaken with 0.15% aqueous ferric chloride solution (100 mL) for 5–7 min. The organic solution was washed with water, 5% sodium bicarbonate solution, and water, and the solvent evaporated under reduced pressure. The residue was purified on a grade 3 alumina column, eluting with chloroform. Recrystallization from chloroform–methanol gave the oxybenziporphyrin (5.4 mg. 0.0084 mmol, 12%) as purple crystals, mp > 260 °C. UV-vis (CHCl_3_): λ_max_/nm (log ε) 417 (sh, 4.57), 450 (4.86), 465 (4.74), 570 (sh, 4.11), 621 (4.46), 668 (3.93), 723 (3.79). UV-vis (400 equivalents TFA-CHCl_3_): λ_max_/nm (log ε) 450 (4.93), 484 (4.54), 620 (4.20), 637 (4.19), 663 (sh, 4.04), 726 (3.82). UV-vis (5% TFA-CHCl_3_): λ_max_/nm (log ε) 378 (4.48), 448 (4.85), 576 (3.87), 627 (4.22), 712 (3.74), 783 (3.78). UV-vis (10% DBU-CHCl_3_): λ_max_/nm (log ε) 447 (4.65), 465 (4.60), 497 (4.47), 620 (3.93), 713 (4.22), 764 (3.99). ^1^H NMR (500 MHz, CDCl_3_, 55 °C): δ 10.52 (s, 1H), 10.40 (s, 1H), 10.36 (s, 1H), 9.13 (s, 1H), 8.67 (d, 1H, *J* = 9.2 Hz), 7.44 (d, 1H, *J* = 9.2 Hz), 3.86–3.80 (m, 4H), 3.49 (s, 3H), 3.40 (s, 3H), 2.17–2.11 (m, 4H), 1.74–1.67 (m, 4H), 1.14 (t, 6H, *J* = 7.4 Hz), −3.99 (br s, 1H), −4.16 (br s, 1H), −7.08 (s, 1H). ^1^H NMR (500 MHz, TFA-CDCl_3_): δ 10.42 (s, 1H), 10.39 (s, 1H), 10.14 (s, 1H), 9.38 (s, 1H), 8.79 (dd, 1H, *J* = 2.1, 8.8 Hz), 7.71 (d, 1H, *J* = 9.0 Hz), 3.74–3.69 (t, 4H, 9,18-CH_2_), 3,25 (s, 3H), 3.23 (s, 3H) (8,19-Me), 2.04–1.97 (m, 4H, 9,18-CH_2_C*H*_2_), 1.67–1.61 (m, 4H, 2 × CH_2_C*H*_2_CH_3_), 1.11–1.07 (2 overlapping triplets, 6H, 2 × (CH_2_)_3_C*H*_3_), −6.59 (s, 1H, 22-H). ^13^C{^1^H} NMR (TFA-CDCl_3_, 125 MHz): δ 151.2, 150.3, 150.2, 142.2, 141.2, 129.1, 124.7, 100.1, 98.6, 33.4, 33.3, 25.5, 23.0, 14.0, 11.6. HRMS (ESI): *m/z* [M + H]^+^ calcd for C_36_H_34_N_7_OS_2_ 644.2261; found 644.2256.

**Computational Studies.** All calculations were performed using Gaussian 16, Revision C.01 [46]. Geometry optimizations were performed using the M06-2X functional and the 6-311++G(d,p) basis set [47,48,49,50]. Vibrational frequencies were computed to confirm the absence of imaginary frequencies and derive zero-point energy and vibrational entropy corrections from unscaled frequencies. Single-point energy calculations were performed on the optimized minima using M06-2X/cc-PVTZ [51]. NICS values were calculated using the GIAO method [52] using CAM-B3LYP/6-31+G(d,p), and AICD plots were obtained from CGST calculations using B3LYP/6-31+G(d) [53]. NICS(0) was calculated at the mean position of all four heavy atoms in the middle of the macrocycle. NICS(a), NICS(b), NICS(c), NICS(d), NICS(e), NICS(f), and NICS(g) values were obtained by applying the same method to the mean position of the heavy atoms that comprise the individual rings of each macrocycle. In addition, NICS(1)*_zz_*, NICS(1a)*_zz_*, NICS(1b)*_zz_*, NICS(1c)*_zz_*, NICS(1d)*_zz_*, NICS(1e)*_zz_*, NICS(1f)*_zz_*, NICS(1f)*zz*, and NICS(1h)*_zz_* were obtained by applying the same method to ghost atoms placed 1 Å above each of the corresponding NICS(0) points and extracting the *zz* contribution of the magnetic tensor. The resulting energies, Cartesian coordinates, and AICD plots can be found in the Supporting Information.

## 4. Conclusions

A series of novel bis(thiadiazolo)benzoporphyrinoids were prepared and characterized. These unusual derivatives retain fully aromatic characteristics but have modified UV-vis spectra. The highly electron-withdrawing fused heterocyclic unit drastically reduces the basicity of the porphyrinoid systems. Nickel(II), copper(II), and zinc(II) complexes of a bis(thiadiazolo)benzoporphyrin were prepared, and these gave strong absorptions above 600 nm, particularly in the case of the zinc complex. The free base structures were poorly soluble, but proton NMR spectra could be obtained, mostly at 55 °C, and these showed that the chemical shifts for the internal and external protons fall into the expected range for these aromatic structures. In this respect, these new porphyrinoids differ from porphyrinoids with annulated electron-withdrawing tropone or phenanthroline units as these show seemingly contradictory upfield shifts to both the peripheral and interior protons. NICS calculations and AICD plots show that the thiadiazole units do not significantly interact with the 18*π*-electron circuits within the macrocycles, providing useful insights into the spectroscopic properties of these structures.

## Figures and Tables

**Table 1:** Selected proton NMR resonances for 1,10-phenanthroline-, phenanthrene-, and bis(thiadiazolo)benzene-fused porphyrins and heteroporphyrins.

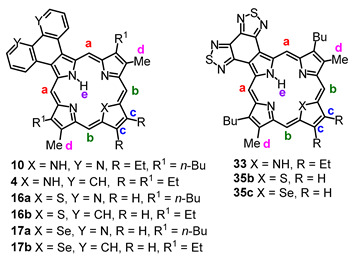					
	**a**	**b**	**c**	**d**	**e**
**10** [20]	10.14	9.49	-	3.52	−4.07
**4** [9]	11.03	10.03	-	3.68	−3.60
**33** ^a^	10.50	9.76	-	3.56	−4.19
**33**	10.78	9.82	-	3.60	−3.77
**16a** [9]	9.80	10.42	9.96	3.25	−4.49
**16b** [22]	11.15	10.67	10.08	3.45	−3.21
**35b** ^b^	10.33	10.21	9.94	3.32	−4.30
**17a** ^a^ [22]	9.78	10.55	10.24	3.22	−4.81
**17a** [22]	10.08	10.60	10.26	3.27	−4.38
**17b** [9]	11.28	10.87	10.41	3.47	−3.40
**35c**	10.69	10.53	10.19	3.33	−3.63

^a^ Proton NMR spectra obtained from CDCl_3_ at 29 °C; ^b^ proton NMR spectrum of **35b** obtained at 50 °C; all other NMR spectra obtained at 55 °C.

**Table 2:** Selected proton NMR resonances for annelated carbaporphyrins.

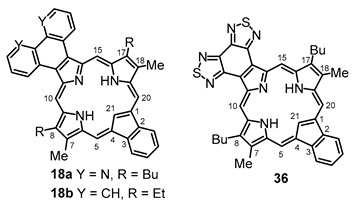					
	5,20-H	10,15-H	7,18-Me	NH	21-CH
**18a** [22]	9.05	8.63	2.87	−6.83	−8.84
**18a** ^†^ [22]	9.41	9.13	2.94	−6.83	−8.75
**18b** [25]	10.36	9.77	3.46	−4.8	−7.12
**36**	10.55	9.65	3.45	−4.93	−7.45

^†^ Proton NMR spectrum obtained at 29 °C; other NMR spectra obtained at 55 °C.

**Table 3:** Selected proton NMR resonances for annelated oxypyri- and oxybenziporphyrins.

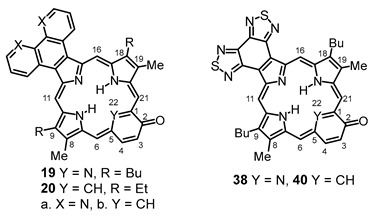					
	21-H	*meso*-H	8,19-Me	NH	22-H
**19a** [22]	10.30	8.82, 8.76, 8.49	3.05, 3.01	−5.97, −6.11	-
**19b** [26]	10.59	10.00, 9.98, 9.06	3.45, 3.29	−4.79	-
**38**	10.90	10.84, 10.78, 9.39	3.61, 3.53	−3.91, −4.01	-
**20a** [22]	9.90	8.97, 8.84, 8.60	3.06, 3.01	−5.4	−7.88
**20a** ^†^ [22]	9.80	8.73. 8.46, 8.38	2.93, 2.90	−5.73, 5.95	−8.22
**20b** [26]	10.13	9.74, 9.65, 8.90	3.31, 3.21	-	−6.90
**40**	10.52	10.40, 10.36, 9.13	3.49, 3.40	−3.99, −4.16	−7.08

^†^ Proton NMR spectrum obtained at 29 °C; other NMR spectra obtained at 55 °C.

**Table 4:** Calculated relative energies (kcal/mol) and NICS values for selected bis(thiadiazolo)benzoporphyrin tautomers.

	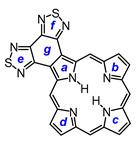 **S_2_-BPa**	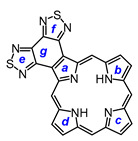 **S_2_-BPb**	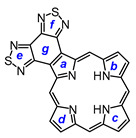 **S_2_-BPc**	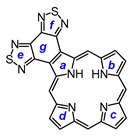 **S_2_-BPd**
Δ*E*	2.18	0.00	8.94	11.16
Δ*G*_298_	2.13	0.00	8.68	10.80
*NICS(0)|NICS(0_zz_)*	−14.14|−35.80	−14.05|−35.72	−13.84|−35.18	−14.01|−35.53
*NICS(a)|NICS(a_zz_)*	−11.40|−27.16	−1.32|−8.37	−0.77|−7.20	−11.78|−27.69
*NICS(b)|NICS(b_zz_)*	−1.97|−12.67	−11.57|−30.83	−11.99|−31.28	−11.62|−30.59
*NICS(c)|NICS(c_zz_)*	−11.40|−30.50	−1.87|−12.54	−11.72|−30.79	−1.35|−11.44
*NICS(d)|NICS(d_zz_)*	−1.97|−12.68	−11.57|−30.82	−1.77|−12.29	−1.49|−11.62
*NICS(e)|NICS(e_zz_)*	−11.92|−27.27	−12.10|−27.58	−12.09|−27.61	−11.97|−27.36
*NICS(f)|NICS(f_zz_)*	−11.92|−27.27	−12.10|−27.58	−12.11|−27.68	−11.95|−27.14
*NICS(g)|NICS(g_zz_)*	+4.12|+4.18	+3.60|+2.66	+3.54|+2.46	+4.09|+4.22

**Table 5:** Calculated relative energies (kcal/mol) and NICS values for bis(thiadiazolo)benzoheteroporphyrin tautomers.

	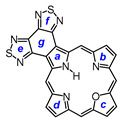 **S_2_-OxBPa**	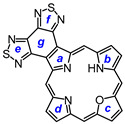 **S_2_-OxBPb**	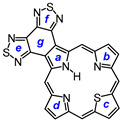 **S_2_-BTPa**	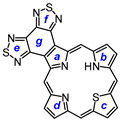 **S_2_-BTPb**
Δ*E*	0.00	2.40	0.00	6.32
Δ*G*_298_	0.00	2.14	0.00	6.52
*NICS(0)|NICS(0_zz_)*	−13.16|−33.10	−13.16|−33.11	−13.52|−32.90	−13.62|−33.22
*NICS(a)|NICS(a_zz_)*	−11.40|−27.37	+0.34|−4.78	−11.75|−28.21	−0.54|−6.88
*NICS(b)|NICS(b_zz_)*	−0.07|−8.46	−11.91|−31.43	−9.25|−11.96	−11.96|−30.98
*NICS(c)|NICS(c_zz_)*	−14.20|−38.20	−15.05|−39.43	−14.01|−32.77	−14.88|−34.18
*NICS(d)|NICS(d_zz_)*	−0.07|−8.46	+0.93|−6.55	−0.67|−9.23	0.00|−7.96
*NICS(e)|NICS(e_zz_)*	−11.89|−27.19	−12.12|−27.77	−11.90|−27.17	−12.08|−27.61
*NICS(f)|NICS(f_zz_)*	−11.89|−27.19	−12.14|−27.80	−11.90|−27.17	−12.11|−27.68
*NICS(g)|NICS(g_zz_)*	+4.14|+4.28	+3.38|+2.07	+4.14|+4.47	+3.52|+2.41
	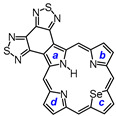 **S_2_-BSPa**	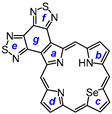 **S_2_-BSPb**		
Δ*E*	0.00	7.11		
Δ*G*_298_	0.00	6.86		
*NICS(0)|NICS(0_zz_)*	−14.10|−31.50	−14.24|−32.41		
*NICS(a)|NICS(a_zz_)*	−12.07|−28.59	+1.22|−8.11		
*NICS(b)|NICS(b_zz_)*	−1.06|−9.76	−12.05|−30.83		
*NICS(c)|NICS(c_zz_)*	−12.19|−27.62	−12.95|−29.15		
*NICS(d)|NICS(d_zz_)*	−1.06|−9.76	−1.01|−9.87		
*NICS(e)|NICS(e_zz_)*	−11.92|−27.16	−12.10|−27.59		
*NICS(f)|NICS(f_zz_)*	−11.92|−27.16	−12.14|−27.69		
*NICS(g)|NICS(g_zz_)*	+4.20|+4.52	+3.49|+2.46		

**Table 6:** Calculated relative energies (kcal/mol) and NICS values for bis(thiadiazolo)benzocarbaporphyrin tautomers.

	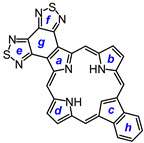 **S_2_-BCBPa**	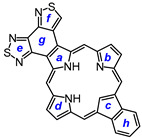 **S_2_-BCBPb**	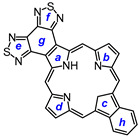 **S_2_-BCBPc**	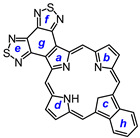 **S_2_-BCBPd**
Δ*E*	0.00	7.67	10.46	15.55
Δ*G*_298_	0.00	7.03	8.71	13.91
*NICS(0)|NICS(0_zz_)*	−13.21|−35.80	−12.78|−31.71	−4.59|−9.46	−5.23|−10.89
*NICS(a)|NICS(a_zz_)*	−2.61|−11.48	−12.42|−26.86	−11.09|−24.45	−2.49|−9.84
*NICS(b)|NICS(b_zz_)*	−12.34|−29.82	−13.33|−39.47	−1.06|−9.21	−0.11|−7.65
*NICS(c)|NICS(c_zz_)*	+6.40|+8.65	+8.27|+21.28	−4.22|−17.35	−5.31|−19.25
*NICS(d)|NICS(d_zz_)*	−12.34|−29.83	−2.88|−14.36	−1.06|−9.21	−11.02|−27.16
*NICS(e)|NICS(e_zz_)*	−12.10|−27.76	−11.97|−27.01	−12.16|−27.82	−12.57|−28.82
*NICS(f)|NICS(f_zz_)*	−12.10|−27.76	−11.95|−26.67	−12.16|−27.82	−12.52|−28.73
*NICS(g)|NICS(g_zz_)*	+3.61|+2.49	+4.09|+4.80	+3.56|+3.20	+2.66|+0.46
*NICS(h)|NICS(h_zz_)*	−5.24|−19.96	−4.76|−16.06	−8.57|−26.58	−8.80|−28.99

**Table 7:** Calculated relative energies (kcal/mol) and NICS values for bis(thiadiazolo)benzo-oxypyriporphyrin tautomers.

	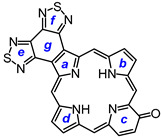 **S_2_-OPBPa**	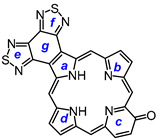 **S_2_-OPBPb**	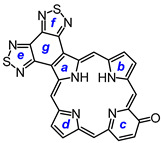 **S_2_-OPBPc**
Δ*E*	0.00	9.56	8.63
Δ*G*_298_	0.00	9.69	8.90
*NICS(0)|NICS(0_zz_)*	−13.26|−33.80	−13.47|−33.92	−13.31|−33.47
*NICS(a)|NICS(a_zz_)*	+1.35|−8.21	−11.18|−26.11	−11.40|−26.62
*NICS(b)|NICS(b_zz_)*	−11.16|−29.96	−1.82|−12.62	−11.46|−30.21
*NICS(c)|NICS(c_zz_)*	+8.75|+7.64	+9.30|+8.59	+8.83|+7.77
*NICS(d)|NICS(d_zz_)*	−12.03|−31.58	−12.39|−32.07	−2.66|−14.13
*NICS(e)|NICS(e_zz_)*	−12.21|−27.91	−12.09|−27.54	−12.07|−27.68
*NICS(f)|NICS(f_zz_)*	−12.18|−27.86	−12.09|−27.72	−12.06|−27.46
*NICS(g)|NICS(g_zz_)*	+3.42|+2.19	+3.86|+3.59	+3.87|+3.65
	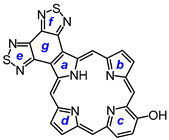 **S_2_-OPBPd**	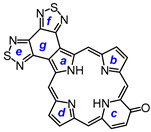 **S_2_-OPBPe**	
Δ*E*	27.74	4.62	
Δ*G*_298_	28.42	5.47	
*NICS(0)|NICS(0_zz_)*	−11.15|−27.31	−11.15|−27.31	
*NICS(a)|NICS(a_zz_)*	−11.53|−26.95	−11.53|−26.95	
*NICS(b)|NICS(b_zz_)*	−1.61|−11.60	−1.61|−11.60	
*NICS(c)|NICS(c_zz_)*	−3.56|−0.70	+3.56|−0.70	
*NICS(d)|NICS(d_zz_)*	−2.14|−12.56	−2.14|−12.56	
*NICS(e)|NICS(e_zz_)*	−12.07|−27.61	−12.07|−27.61	
*NICS(f)|NICS(f_zz_)*	−12.04|−27.58	−12.04|−27.58	
*NICS(g)|NICS(g_zz_)*	+3.87|+3.67	+3.87|+3.67	

**Table 8:** Calculated relative energies (kcal/mol) and NICS values for bis(thiadiazolo)benzo-oxybenziporphyrin tautomers.

	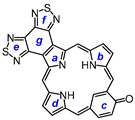 **S_2_-OBBPa**	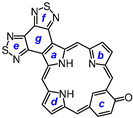 **S_2_-OBBPb**	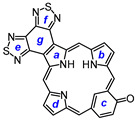 **S_2_-OBBPc**	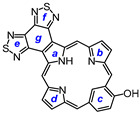 **S_2_-OBBPd**
Δ*E*	0.00	6.66	5.76	11.41
Δ*G*_298_	0.00	6.57	5.68	11.27
*NICS(0)|NICS(0_zz_)*	−12.51|−32.14	−11.86|−29.90	−11.87|−29.91	−1.61|−2.61
*NICS(a)|NICS(a_zz_)*	−1.59|−8.23	−10.22|−22.03	−11.55|−25.05	−3.30|−6.05
*NICS(b)|NICS(b_zz_)*	−11.48|−28.17	−3.08|+15.42	−12.45|−37.57	−0.35|−6.59
*NICS(c)|NICS(c_zz_)*	+9.48|+11.43	+9.99|+20.20	−10.22|−20.75	−4.16|−12.91
*NICS(d)|NICS(d_zz_)*	−13.18|−31.88	−14.44|−41.50	−4.27|−17.72	−1.31|−9.26
*NICS(e)|NICS(e_zz_)*	−12.28|−28.16	−12.29|−27.65	−12.14|−27.55	−13.12|−30.46
*NICS(f)|NICS(f_zz_)*	−12.24|−28.08	−12.24|−27.89	−12.11|−27.26	−13.08|−30.39
*NICS(g)|NICS(g_zz_)*	+3.33|+1.89	+3.59|+3.23	+3.81|+4.04	+1.96|−1.54

## Data Availability

The data presented in this article are available in the Supporting Information Section.

## Supplementary Materials

The following supporting information can be downloaded at https://www.mdpi.com/article/10.3390/molecules30081822/s1, Figures S1–S26: Selected UV-vis spectra; Figures S27–S52: Selected proton, ^1^H-^1^H COSY, HSQC, DEPT-135, and ^13^C{^1^H} NMR spectra; Figures S53–S63: Selected HR-TOF-ESI mass spectra; Figures S64–S86: AICD plots; Figures S87–S92: Calculated bond lengths for selected porphyrinoids; Table S1: Calculated Gibb’s free energies and electronic energies of porphyrinoid tautomers; Table S2: Cartesian coordinates.

## References

[1] Lash T.D., Kadish K.M., Smith K.M., Guilard R. (2000). Synthesis of Novel Porphyrinoid Chromophores. The Porphyrin Handbook.

[2] Ono N., Yamada H., Okujima T., Smith K.M., Kadish K.M., Guilard R. (2010). Synthesis of Porphyrins with Fused Aromatic Rings. Handbook of Porphyrin Science—With Applications to Chemistry, Physics, Material Science, Engineering, Biology and Medicine.

[3] Cheprakov A.V., Smith K.M., Kadish K.M., Guilard R. (2011). The Synthesis of π-Extended Porphyrins. Handbook of Porphyrin Science—With Applications to Chemistry, Physics, Material Science, Engineering, Biology and Medicine.

[4] Clezy P.S., Fookes C.J.R., Mirza A.H. (1977). The Chemistry of Pyrrolic Compounds. XXXVII. Monobenzoporphyrins: The Rhodoporphyrin of Petroleum Deposits. Aust. J. Chem..

[5] Lash T.D. (1993). Geochemical Origins of Sedimentary Benzoporphyrins and Tetrahydrobenzoporphyrins. Energy Fuels.

[6] Lash T.D., Denny C.P. (1995). Porphyrins with Exocyclic Rings. Part 5. Synthesis of a Naphtho[1,2-*b*]porphyrin. Tetrahedron.

[7] Manley J.M., Roper T.J., Lash T.D. (2005). Synthesis of Isomeric Angularly Annealed Dinaphthoporphyrin Systems:  Examination of the Relative Positioning and Orientation of Ring Fusion as Factors Influencing the Porphyrin Chromophore. J. Org. Chem..

[8] Novak B.H., Lash T.D. (1998). Porphyrins with Exocyclic Rings. 11. Synthesis and Characterization of Phenanthroporphyrins, a New Class of Modified Porphyrin Chromophores. J. Org. Chem..

[9] Lash T.D., Rauen P.J. (2021). Extended Porphyrinoid Chromophores: Heteroporphyrins Fused to Phenanthrene and Acenaphthylene. Tetrahedron.

[10] Gandhi V., Thompson M.L., Lash T.D. (2010). Porphyrins with Exocyclic Rings. Part 24. Synthesis and Spectroscopic Properties of Pyrenoporphyrins, Potential Building Blocks for Porphyrin Molecular Wires. Tetrahedron.

[11] Lash T.D., Mathius M.A., AbuSalim D.I. (2022). Synthesis of Chrysoporphyrins and a Related Benzopyrene-Fused System. J. Org. Chem..

[12] Lash T.D., Chandrasekar P., Osuma A.T., Chaney S.T., Spence J.D. (1998). Porphyrins with Exocyclic Rings. 13. Synthesis and Spectroscopic Characterization of Highly Modified Porphyrin Chromophores with Fused Acenaphthylene and Benzothiadiazole Rings. J. Org. Chem..

[13] Spence J.D., Lash T.D. (2000). Porphyrins with Exocyclic Rings. Part 14. Synthesis of Tetraacenaphthoporphyrins, a New Family of Highly Conjugated Porphyrins with Record Breaking Long Wavelength Electronic Absorptions. J. Org. Chem..

[14] Cillo C.M., Lash T.D. (2005). Porphyrins with Exocyclic Rings. Part 20: Synthesis and Spectroscopic Characterization of Porphyrins with Fused 2,1,3-Benzoxadiazole and 2,1,3-Benzoselenadiazole Moieties. Tetrahedron.

[15] Lash T.D. (2001). Modification of the Porphyrin Chromophore by Ring Fusion: Identifying Trends due to Annelation of the Porphyrin Nucleus. J. Porphyr. Phthalocyanines.

[16] Salrin J.S., Carpenter B.G., AbuSalim D.I., Lash T.D. (2024). Assessment of Conjugation Pathways in *N*-Methylporphyrins that Are Fused to Acenaphthylene, Phenanthrene, or Pyrene: Evidence for the Presence of Alternative Aromatic Circuits. J. Org. Chem..

[17] Cooper C., Paul R., Alsaleh A., Washburn S., Rackers W., Kumar S., Nesterov V.N., D’Souza F., Vinogradov S.A., Wang H. (2023). Naphthodithiophene-Fused Porphyrins: Synthesis, Characterization, and Impact of Extended Conjugation on Aromaticity. Chem. Eur. J..

[18] Lash T.D., Gandhi V. (2000). Porphyrins with Exocyclic Rings. 15. Synthesis of Quino- and Isoquinoporphyrins, Aza Analogues of the Naphthoporphyrins. J. Org. Chem..

[19] Lin Y., Lash T.D. (1995). Porphyrin Synthesis by the “3 + 1” Methodology: A Superior Approach for the Preparation of Porphyrins with Fused 9,10-Phenanthroline Subunits. Tetrahedron Lett..

[20] Lash T.D., Lin Y., Novak B.H., Parikh M.D. (2005). Porphyrins with Exocyclic Rings. Part 19: Efficient Syntheses of Phenanthrolinoporphyrins. Tetrahedron.

[21] Cramer E.K., Lash T.D. (2022). Synthesis of a Series of Tropone-fused Porphyrinoids. J. Org. Chem..

[22] Ujah V.C., AbuSalim T.D., Lash T.D. (2025). Synthesis, Protonation and Aromatic Characteristics of a Series of 1,10-Phenanthroline-Fused Porphyrinoids. J. Org. Chem..

[23] Siri O., Jaquinod L., Smith K.M. (2000). Coplanar Conjugated *β*- Nitroporphyrins and Some Aspects of Nitration of Porphyrins with N_2_O_4_. Tetrahedron Lett..

[24] Ko M.-S., Roh T.-H., Desale P.P., Choi S.-W., Cho D.G. (2024). Effects of Electron-Withdrawing and Electron-Donating Groups on Aromaticity in Cyclic Conjugated Polyenes. J. Am. Chem. Soc..

[25] Lash T.D., Hayes M.J., Spence J.D., Muckey M.A., Ferrence G.M., Szczepura L.F. (2002). Conjugated Macrocycles Related to the Porphyrins. Part 21. Synthesis, Spectroscopy, Electrochemistry and Structural Characterization of Carbaporphyrins. J. Org. Chem..

[26] Lash T.D., Chaney S.T., Richter D.T. (1998). Conjugated Macrocycles Related to the Porphyrins. Part 12. Oxybenzi- and Oxypyriporphyrins: Aromaticity and Conjugation in Highly Modified Porphyrinoid Structures. J. Org. Chem..

[27] Cillo C.M., Geiger M.A., Lash T.D. (2020). Exploring the Limitations of the MacDonald ‘3 + 1’ Condensation in the Preparation of Porphyrins with Fused Electron-withdrawing Heterocyclic Rings: Synthesis of a Bis(thiadiazolo)benzoporphyrin and a Related Benzocarbaporphyrin. Tetrahedron Lett..

[28] Donzello M.P., Ercolani C., Kadish K.M., Ricciardi G., Rosa A., Stuzhin P.A. (2007). Tetrakis(thiadiazolo)porphyrinazines. 5. Electrochemical and DFT/TDDFT Studies of the Free-Base Macrocycle and Its Mg^II^, Zn^II^, and Cu^II^ Complexes. Inorg. Chem..

[29] Tardieux C., Bolze F., Gros C.P., Guilard R. (1998). New One-Step Synthesis of 3,4-Disubstituted Pyrrole-2,5-dicarbaldehydes. Synthesis.

[30] Latham A.N., Lash T.D. (2020). Synthesis and Characterization of *N*-Methylporphyrins, Heteroporphyrins, Carbaporphyrins, and Related Systems. J. Org. Chem..

[31] Arnold Z. (1965). Synthetic Reactions of Dimethylformamide. XXII. Formation and Preparation of Formyl Derivatives of Indene. Collect. Czech. Chem. Commun..

[32] Lash T.D., Chaney S.T. (1996). Conjugated Macrocycles Related to the Porphyrins. Part 6. Oxypyriporphyrin, the First Fully Aromatic Porphyrinoid Macrocycle with a Pyridine Subunit. Chem. Eur. J..

[33] Medforth C.J., Kadish K.M., Smith K.M., Guilard R. (2000). NMR Spectroscopy of Diamagnetic Porphyrins. The Porphyrin Handbook.

[34] Bonnett R. (1995). Photosensitizers of the Porphyrin and Phthalocyanine Series for Photodynamic Therapy. Chem. Soc. Rev..

[35] Ethirajan M., Chen Y., Joshi P., Pandey R.K. (2011). The Role of Porphyrin Chemistry in Tumor Imaging and Photodynamic Therapy. Chem. Soc. Rev..

[36] Smith K.M. (1975). Porphyrins and Metalloporphyrins.

[37] Smith K.M., Abraham R.J., Pearson H. (1982). The NMR Spectra of Porphyrins-19. 13C and Proton NMR Spectra of Metal-free porphyrins with the Type-IX Substituent Orientation, and of their Zinc(II) Complexes. Tetrahedron.

[38] Lash T.D. (2017). Carbaporphyrinoid Systems. Chem. Rev..

[39] Wu J.I., Fernandez I., Schleyer P.v.R. (2013). Description of Aromaticity in Porphyrinoids. J. Am. Chem. Soc..

[40] Ghosh A. (1998). First-Principles Quantum Chemical Studies of Porphyrins. Acc. Chem. Res..

[41] Aihara J.-I., Nakagami Y., Sekine R., Makino M. (2012). Validity and Limitations of the Bridged Annulene Model for Porphyrins. J. Phys. Chem. A.

[42] Valiev R.R., Fliegl H., Sundholm D. (2015). Predicting the Degree of Aromaticity of Novel Carbaporphyrinoids. Phys. Chem. Chem. Phys..

[43] AbuSalim D.I., Lash T.D. (2023). Aromatic Character and Relative Stability of Pyrazoloporphyrin Tautomers and Related Protonated Species: Insights into How Pyrazole Changes the Properties of Carbaporphyrinoid Systems. Molecules.

[44] Schleyer P.v.R., Maerker C., Dransfeld A., Jiao H., van Eikema Hommes N.J.R. (1996). Nucleus-Independent Chemical Shifts: A Simple and Efficient Aromaticity Probe. J. Am. Chem. Soc..

[45] Geuenich D., Hess K., Köhler F., Herges R. (2005). Anisotropy of Induced Current Density (ACID), a General Method to Quantify and Visualize Electronic Delocalization. Chem. Rev..

[46] Frisch M.J., Trucks G.W., Schlegel H.B., Scuseria G.E., Robb M.A., Cheeseman J.R., Scalmani G., Barone V., Petersson G.A., Nakatsuji H. (2019). Gaussian 16. Revision C.01.

[47] Clark T., Chandrasekhar J., Spitznagel G.W., Schleyer R.v.P. (1983). Efficient Diffuse Function-augmented Basis Sets for Anion Calculations. III. The 3-21+G Basis Set for First-Row Elements, Li-F. J. Comput. Chem..

[48] Ditchfield R., Hehre W.J., Pople J.A. (1971). Self-Consistent Molecular-Orbital Methods. IX. An Extended Gaussian-Type Basis for Molecular-Orbital Studies of Organic Molecules. J. Chem. Phys..

[49] Hariharan P.C., Pople J.A. (1973). The Influence of Polarization Functions on Molecular Orbital Hydrogenation Energies. Theor. Chim. Acta.

[50] Hehre W.J., Ditchfield R., Pople J.A. (1972). Self-Consistent Molecular Orbital Methods. XII. Further Extensions of Gaussian-Type Basis Sets for Use in Molecular Orbital Studies of Organic Molecules. J. Chem. Phys..

[51] Dunning T.H. (1989). Gaussian Basis Sets for use in Correlated Molecular Calculations. I. The Atoms Boron through Neon and Hydrogen. J. Chem. Phys..

[52] Chen Z., Wannere C.S., Corminboeuf C., Puchta R., Schleyer P.v.R. (2005). Nucleus-Independent Chemical Shifts (NICS) as an Aromaticity Criterion. Chem. Rev..

[53] Herges R., Geuenich D. (2001). Delocalization of Electrons in Molecules. J. Phys. Chem. A.

