# Supplementary material for: Synthesis and Spectroscopic Characterization of Bis(thiadiazolo)benzoporphyrinoids: Insights into the Properties of Porphyrin-Type Systems with Strongly Electron-Withdrawing β,β’-Fused Rings
Source: Molecules. 2025 Apr 18;30(8):1822. doi: 10.3390/molecules30081822 (PMC12029806; doi:10.3390/molecules30081822)
Supplement: Supplementary file 1 [file molecules-30-01822-s001.zip › molecules-3578242-supplementary.pdf]

Supporting information for

Synthesis and spectroscopic characterization of bis(thiadiazolo)benzo-porphyrinoids: insights into the properties of porphyrin-type systems with strongly electron-withdrawing β,β'-fused rings

Timothy D. Lash,[1*] Catherine M. Cillo,[1] and Deyaa I. AbuSalim,[1,2,3]

[1]Department of Chemistry, Illinois State University, Normal, Illinois 61790-4160

[2]Department of Chemistry, Rowan University, Glassboro, New Jersey 08028

[3]STEM Department, Rowan College of South Jersey, Vineland, New Jersey 08360

Table of Contents

Page

S2-S14	Selected UV-Vis spectra (Figures S1-S26)
S15-S45	Selected proton, DEPT-135, ^1H-^1H COSY, HSQC and carbon-13 NMR spectra (Figures S27-S45)
S46-S52	Selected mass spectra (Figures S53-S63)
S53-S75	AICD plots (Figures S64-S86)
S76-S81	Calculated bond lengths for selected structures (Figures S87-S92)
S82	Calculated Gibbs free energies for the optimized structures (Table S1)
S83-S106	Cartesian Coordinates (Table S2)

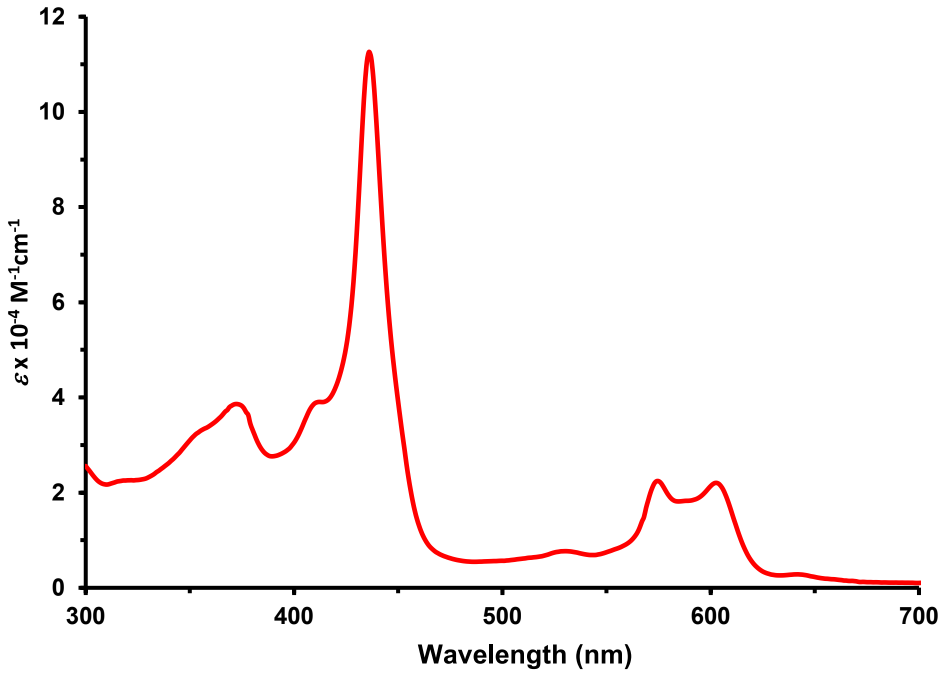

Figure S1. UV-vis spectrum of bis(thiadiazolobenzo)porphyrin **33** in chloroform.

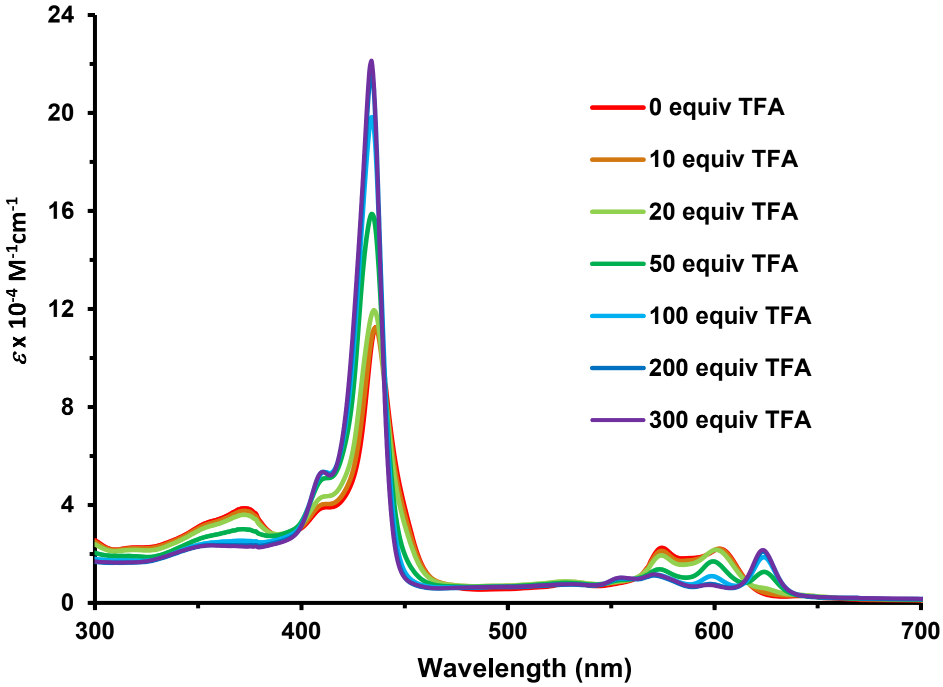

Figure S2. UV-vis spectra of bis(thiadiazolobenzo)porphyrin **33** in chloroform with 0-300 equivalents of TFA.

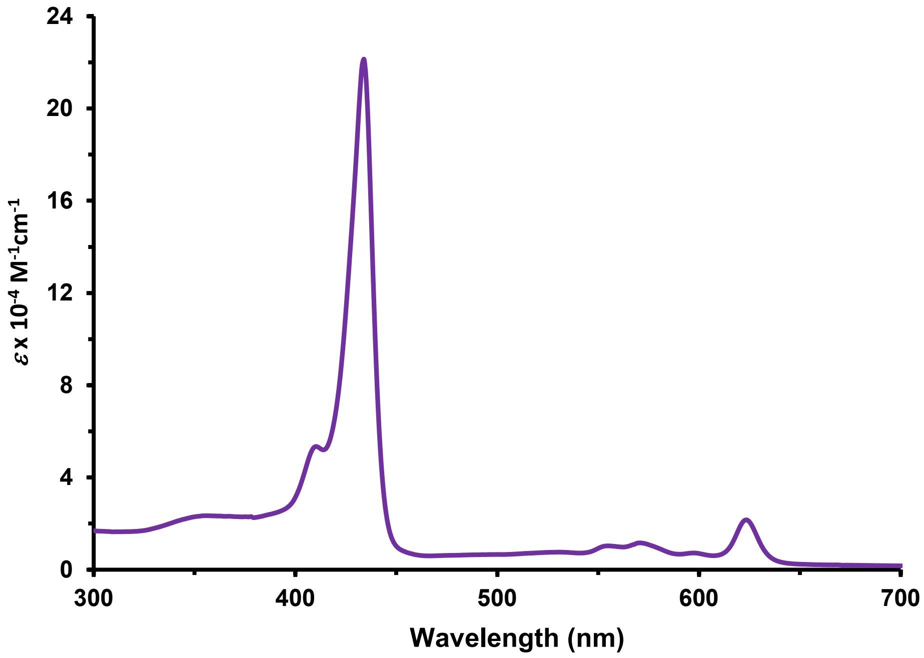

Figure S3. UV-vis spectrum of porphyrin **33** in chloroform with 300 equivalents of TFA.

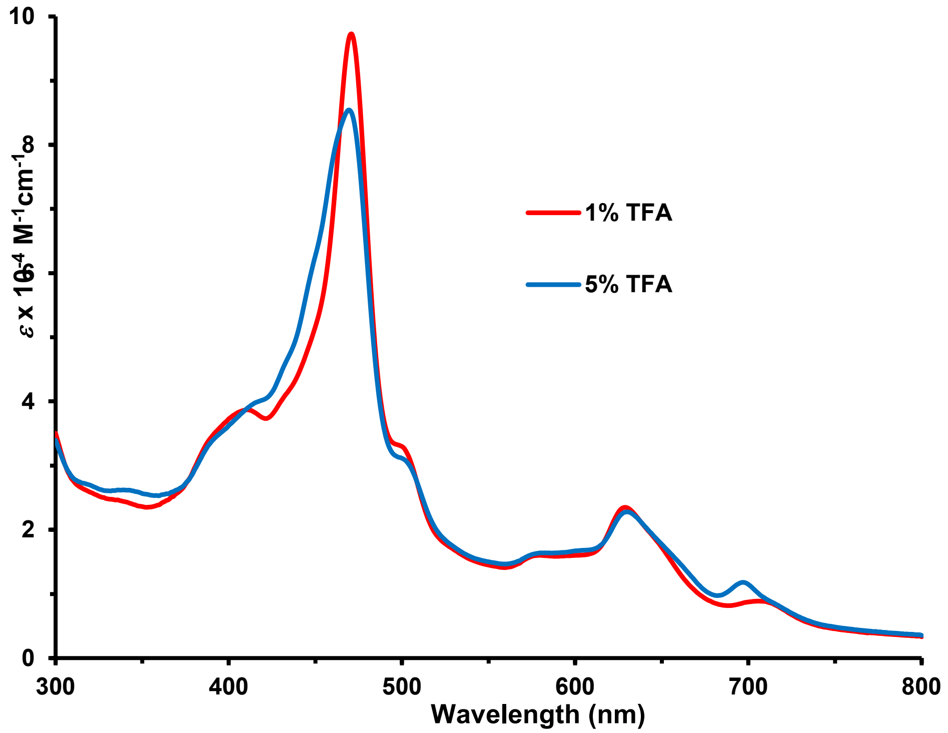

Figure S4. UV-vis spectra of porphyrin **33** in 1% TFA-chloroform and 5% TFA-chloroform.

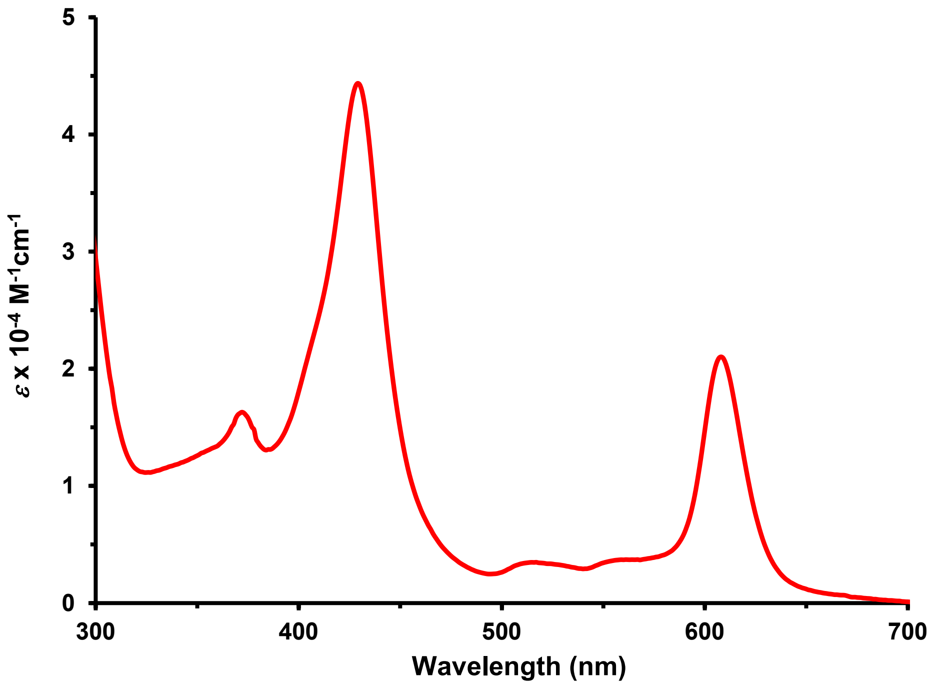

Figure S5. UV-vis spectrum of nickel(II) complex **22Ni** in chloroform.

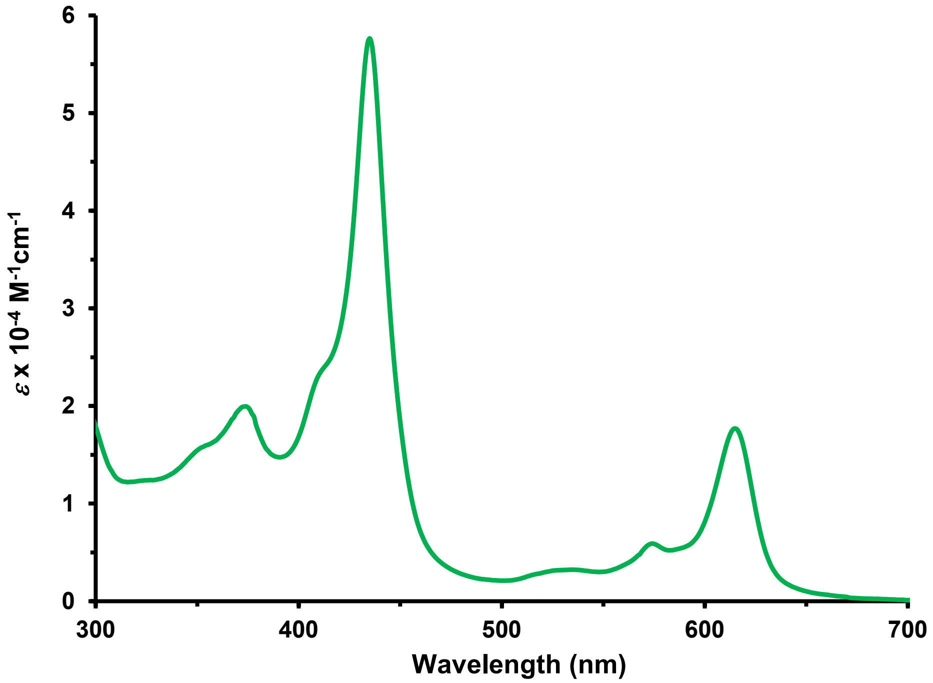

Figure S6. UV-vis spectrum of copper(II) complex **33Cu** in chloroform.

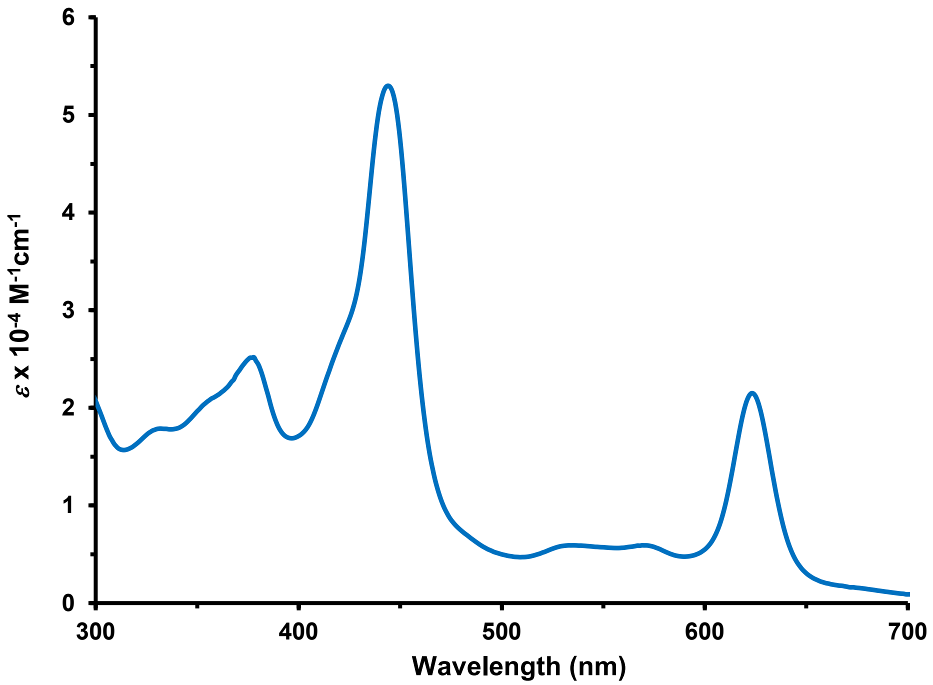

Figure S7. UV-vis spectrum of zinc complex **33Zn** in chloroform.

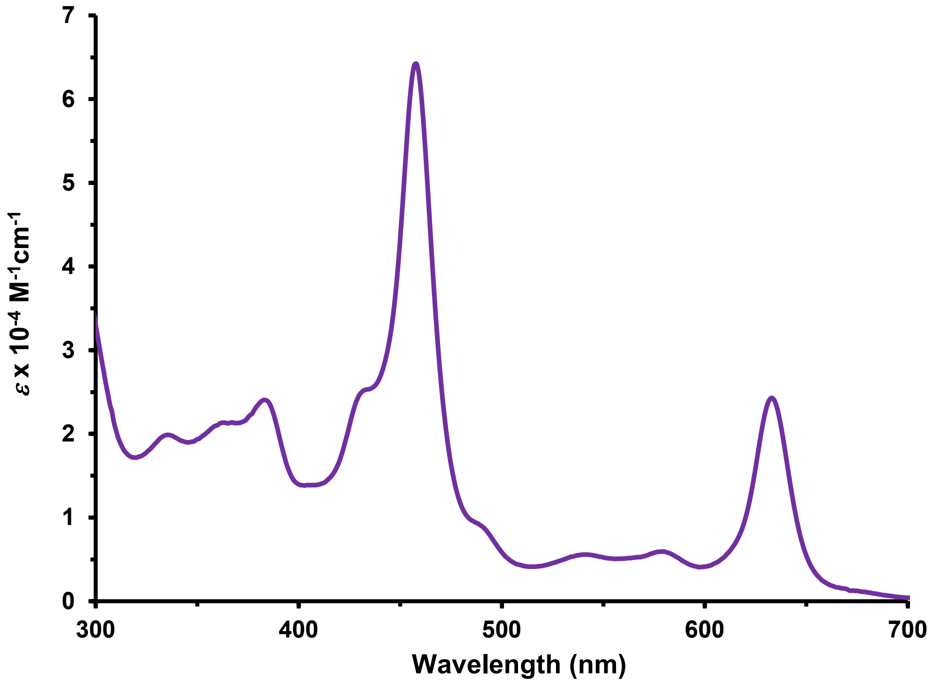

Figure S8. UV-vis spectrum of zinc complex **33Zn** in 1% pyrrolidine-chloroform.

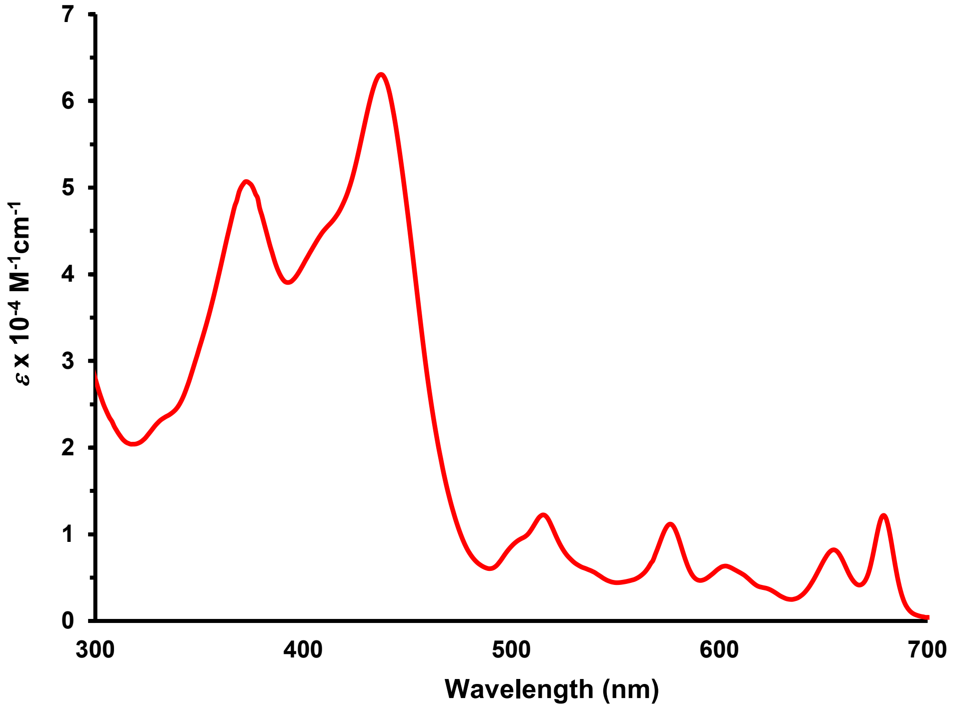

Figure S9. UV-vis spectrum of oxaporphyrin **35a** in 1% triethylamine-chloroform.

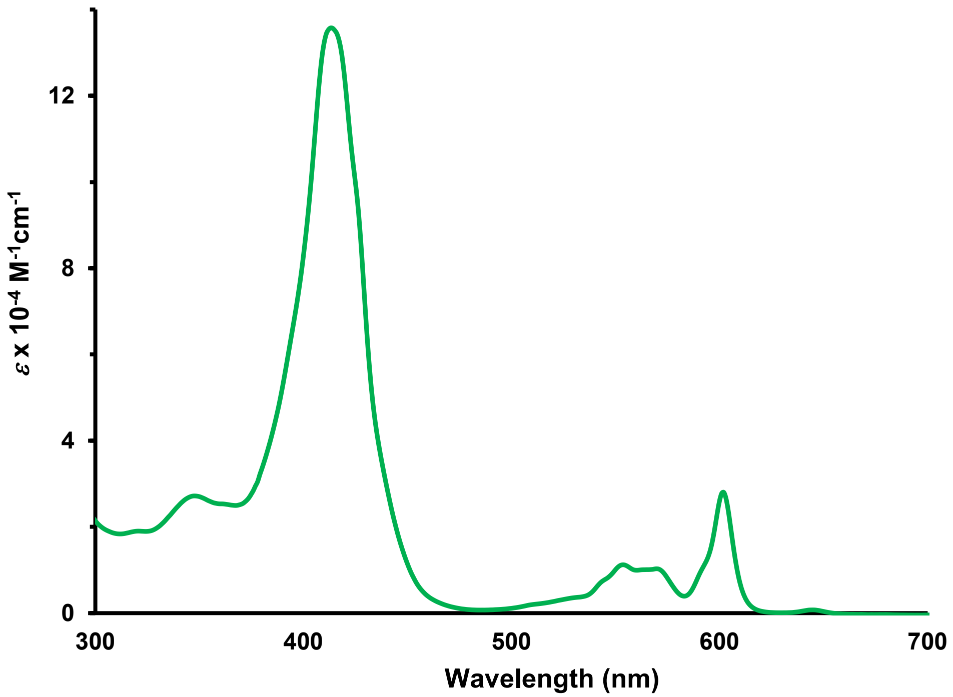

Figure S10. UV-vis spectrum of oxaporphyrin **35a.HCl** in chloroform.

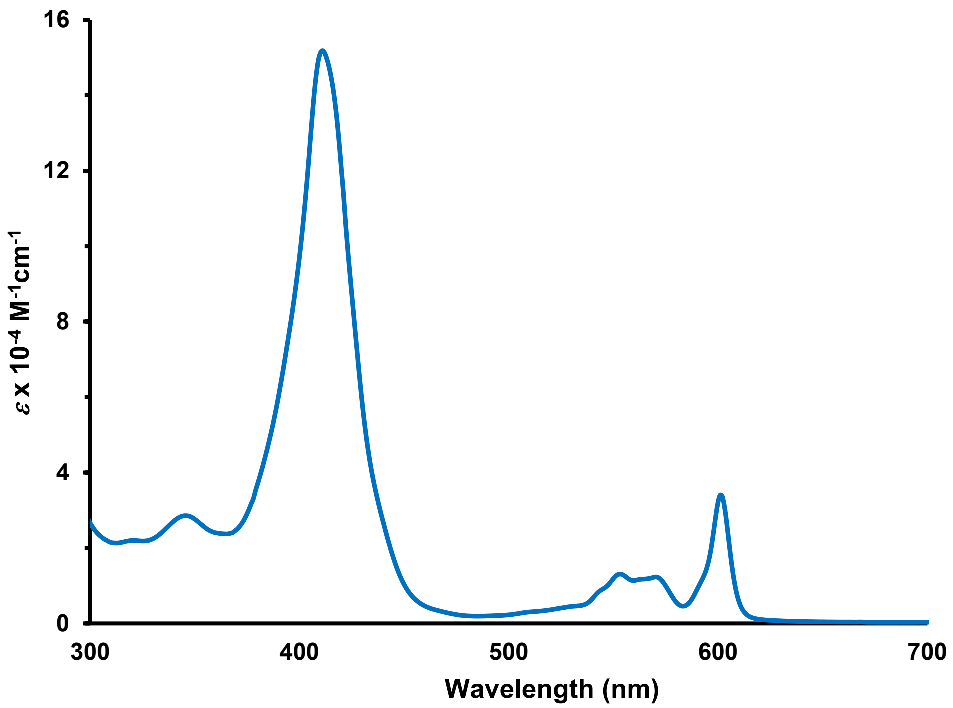

Figure S11. UV-vis spectrum of oxaporphyrin **35a.HCl** in 5% TFA-chloroform.

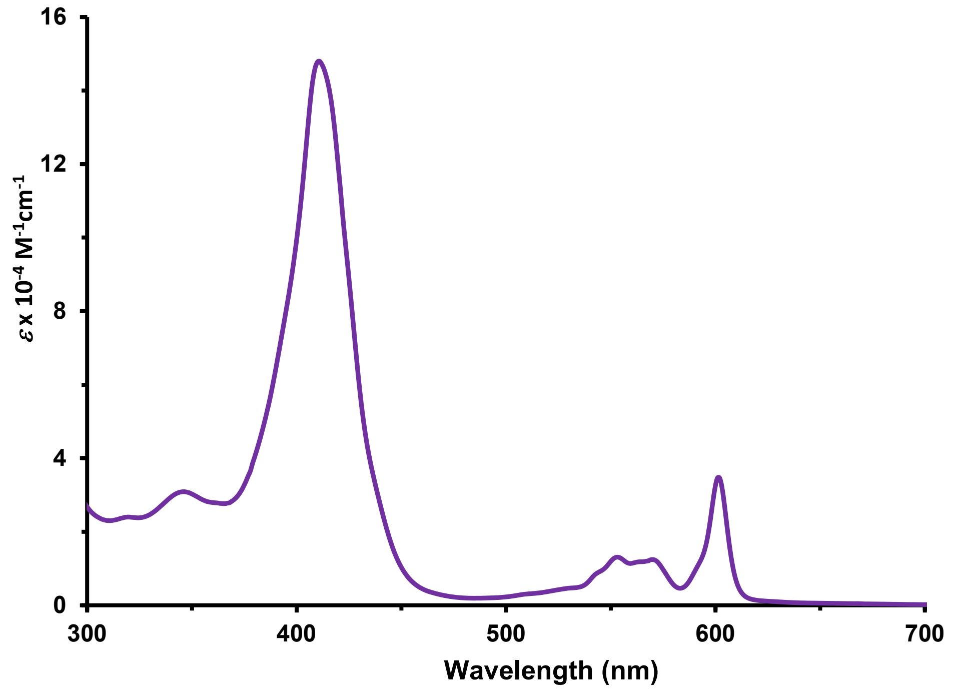

Figure S12. UV-vis spectrum of oxaporphyrin **35a.HCl** in 20% TFA-chloroform.

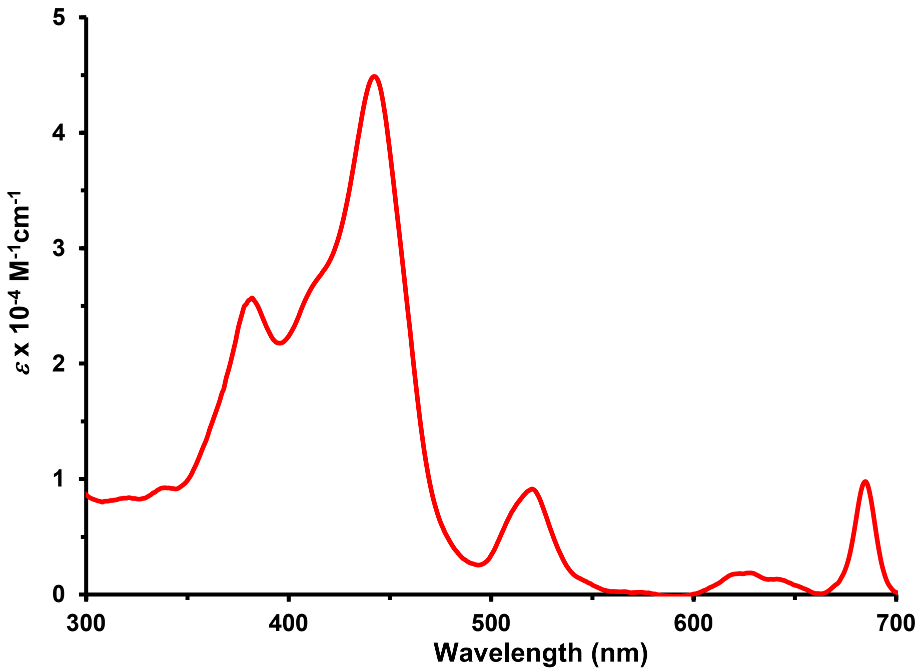

Figure S13. UV-vis spectrum of thiaporphyrin **35b** in chloroform.

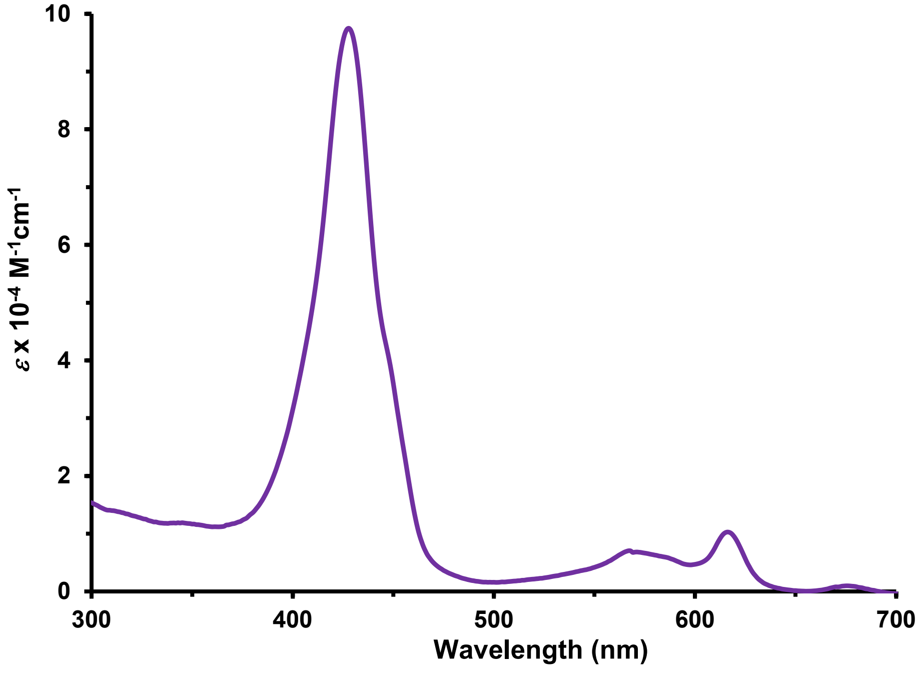

Figure S14. UV-vis spectrum of thiaporphyrin **35b** in 5% TFA-chloroform.

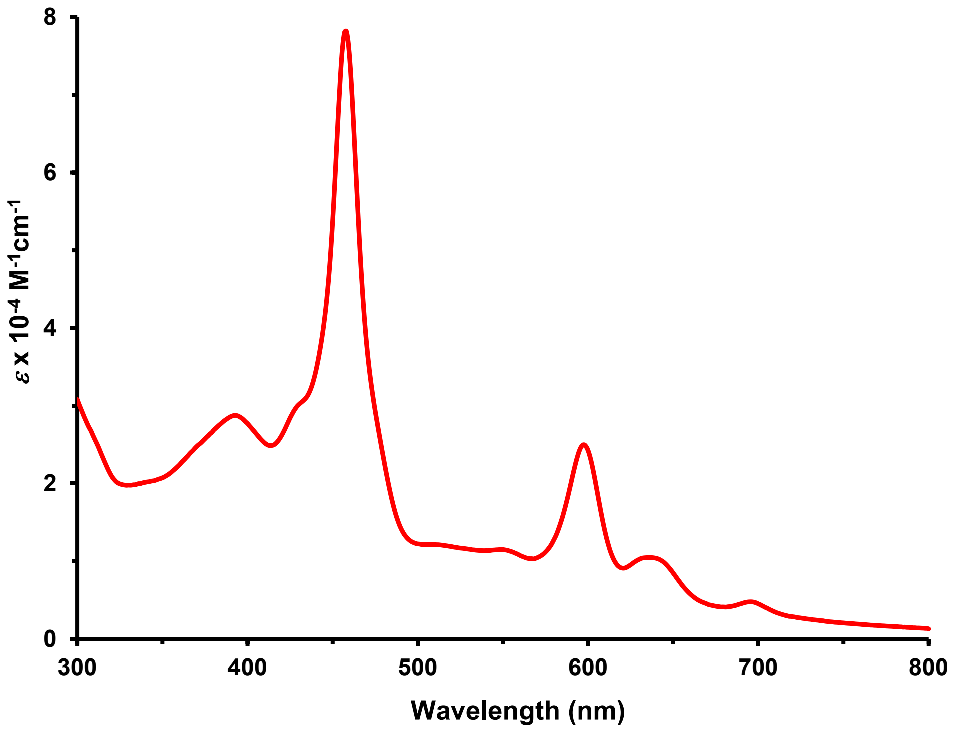

Figure S15. UV-vis spectrum of carbaporphyrins **36** in chloroform.

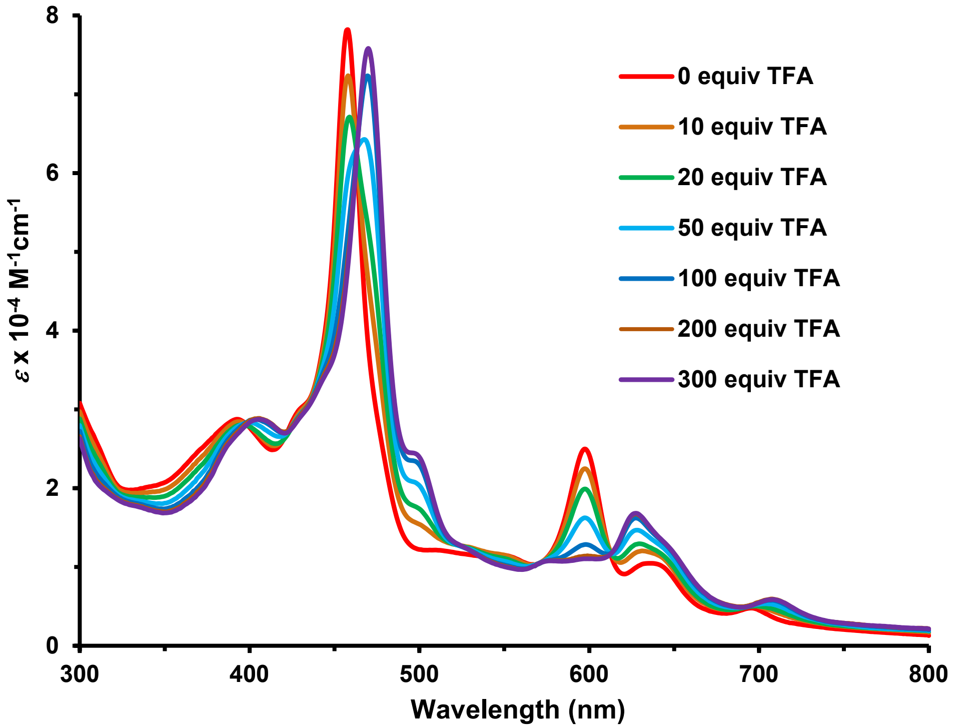

Figure S16. UV-vis spectra of carbaporphyrins **36** in chloroform with 0-300 equivalents of TFA.

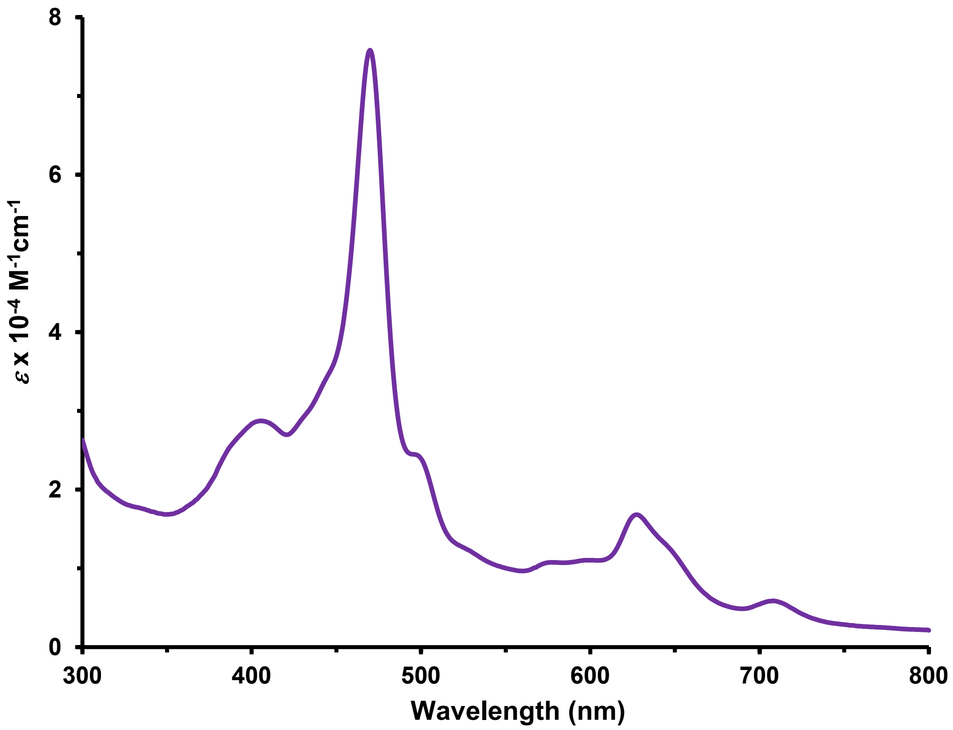

Figure S17. UV-vis spectrum of carbaporphyrins **36** in chloroform with 300 equivalents of TFA.

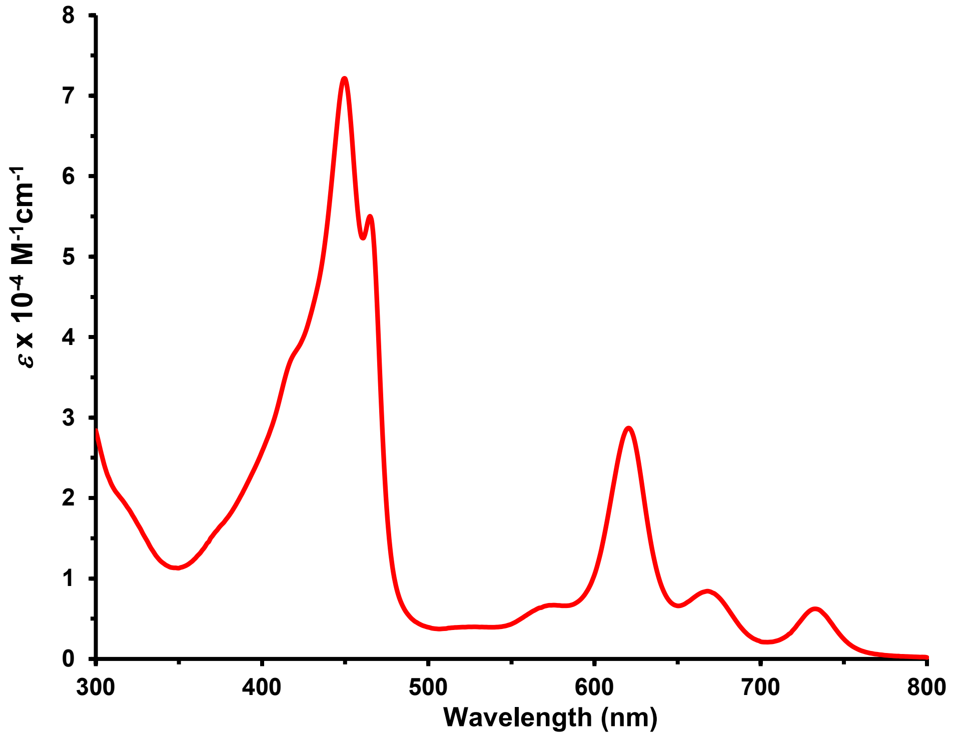

Figure S18. UV-vis spectrum of oxybenziporphyrin **40** in chloroform.

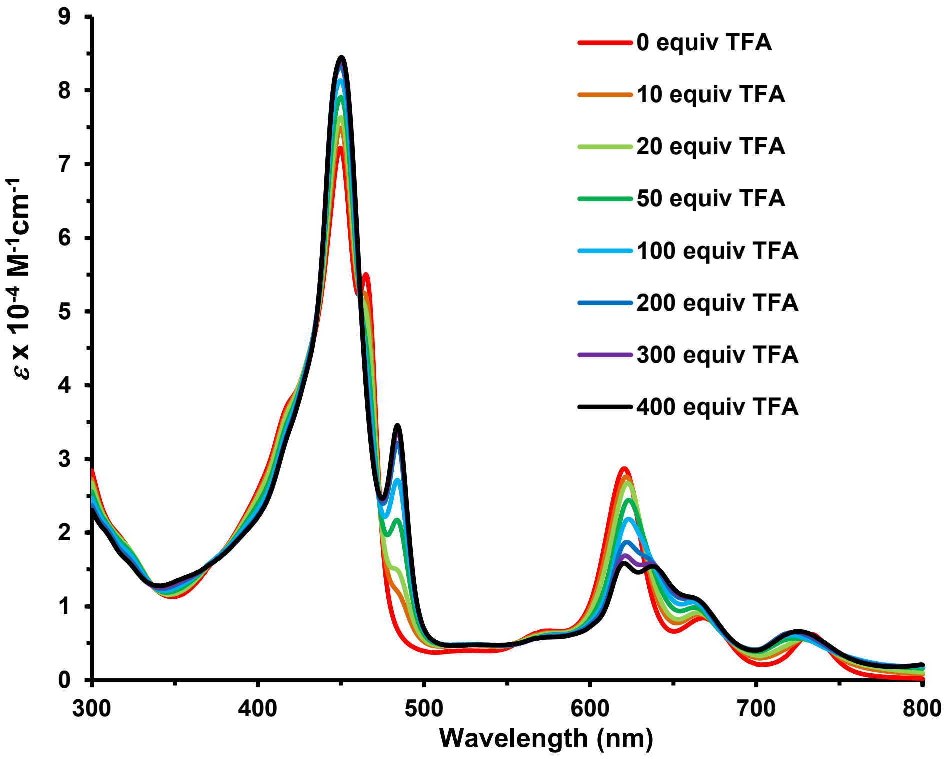

Figure S19. UV-vis spectra of oxybenziporphyrin **40** in chloroform with 0-400 equivalents of TFA.

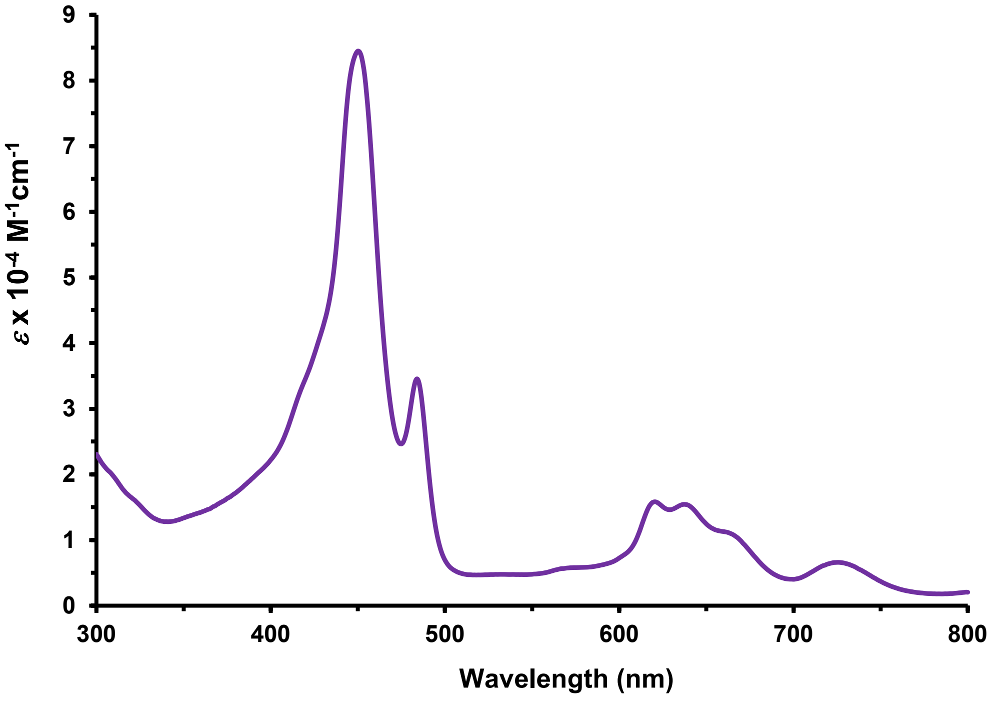

Figure S20. UV-vis spectrum of oxybenziporphyrin **40** in chloroform with 400 equivalents of TFA.

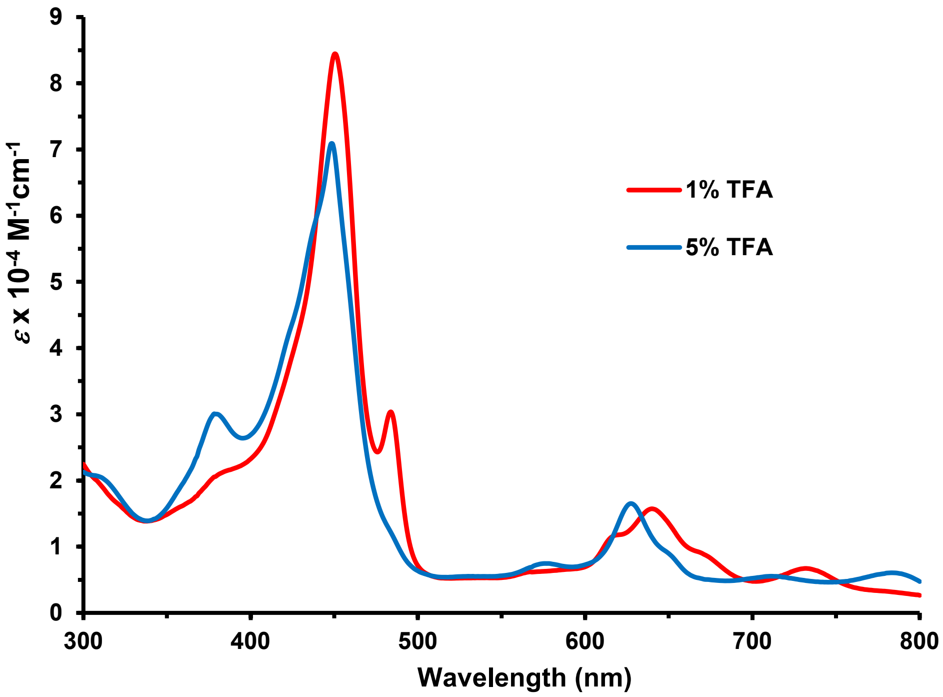

Figure S21. UV-vis spectra of oxybenziporphyrin **40** in 1% TFA-$CHCl_3$ and 5% TFA-$CHCl_3$.

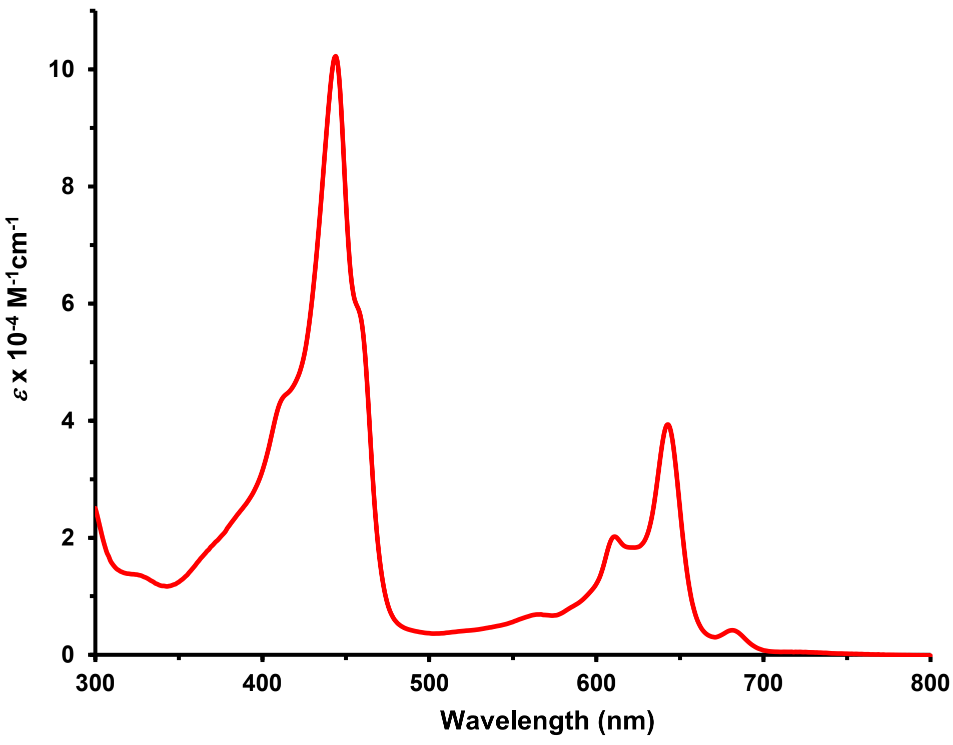

Figure S22. UV-vis spectrum of oxypyriporphyrin **38** in chloroform.

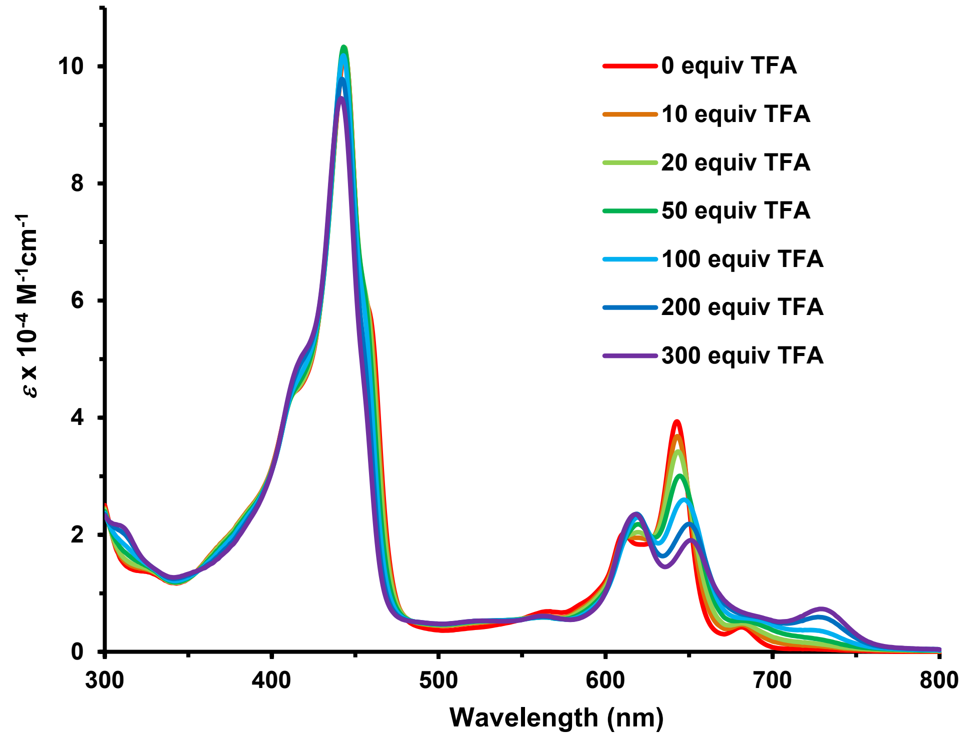

Figure S23. UV-vis spectra of oxypyriporphyrin **38** in chloroform with 0-300 equivalents of TFA.

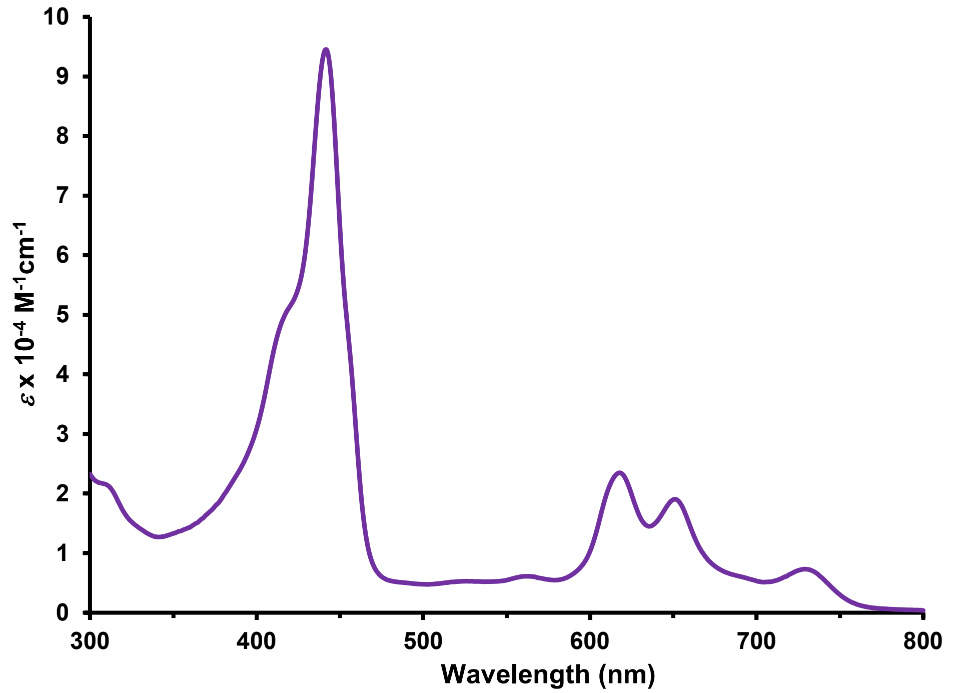

Figure S24. UV-vis spectrum of oxypyriporphyrin **38** in chloroform with 300 equivalents of TFA.

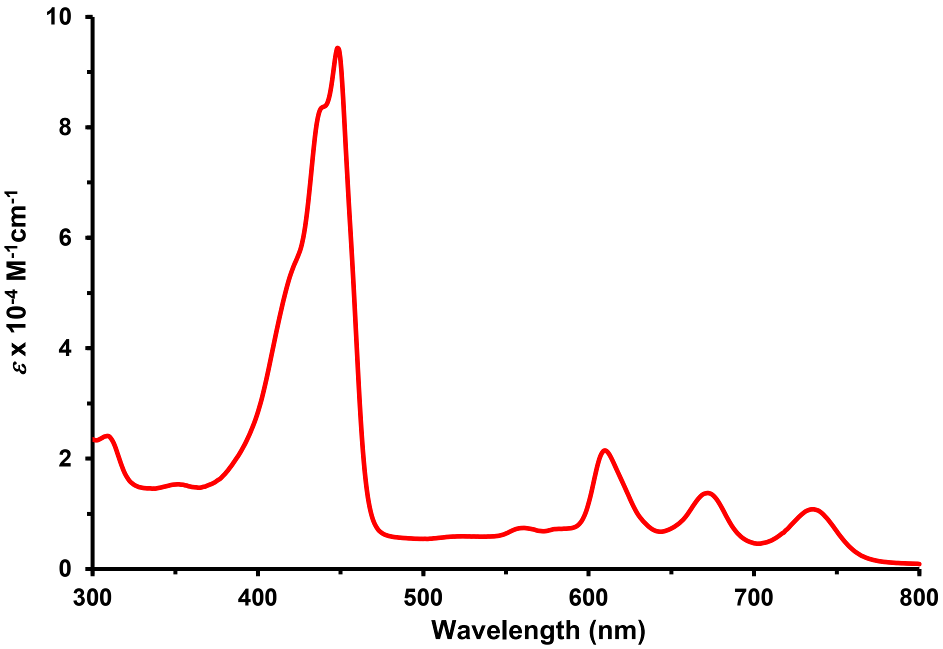

Figure S25. UV-vis spectrum of oxypyriporphyrin **38** in 1% TFA-chloroform.

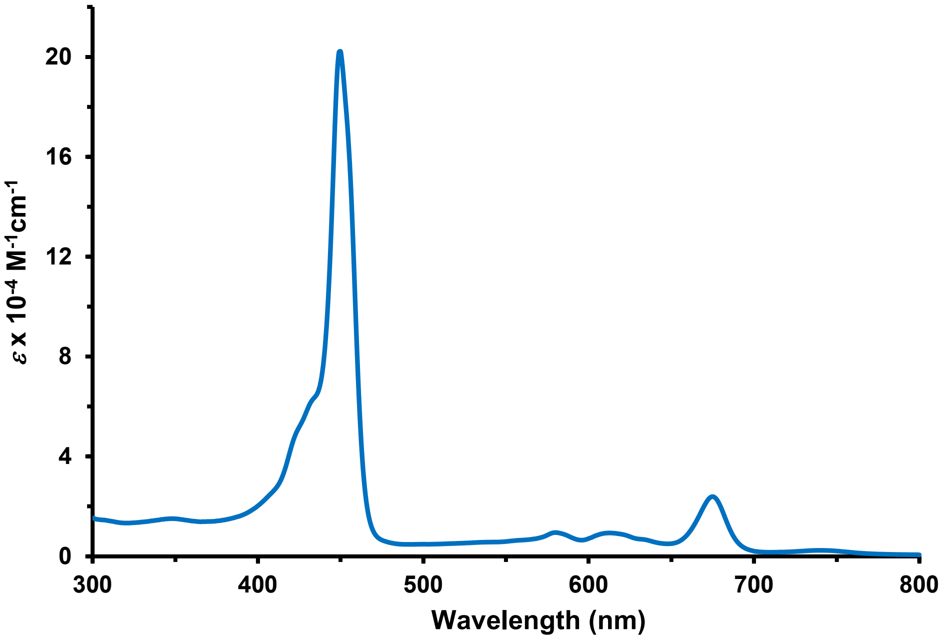

Figure S26. UV-vis spectra of oxypyriporphyrin **38** in chloroform with 1% TFA and 5% TFA in $CHCl_3$.

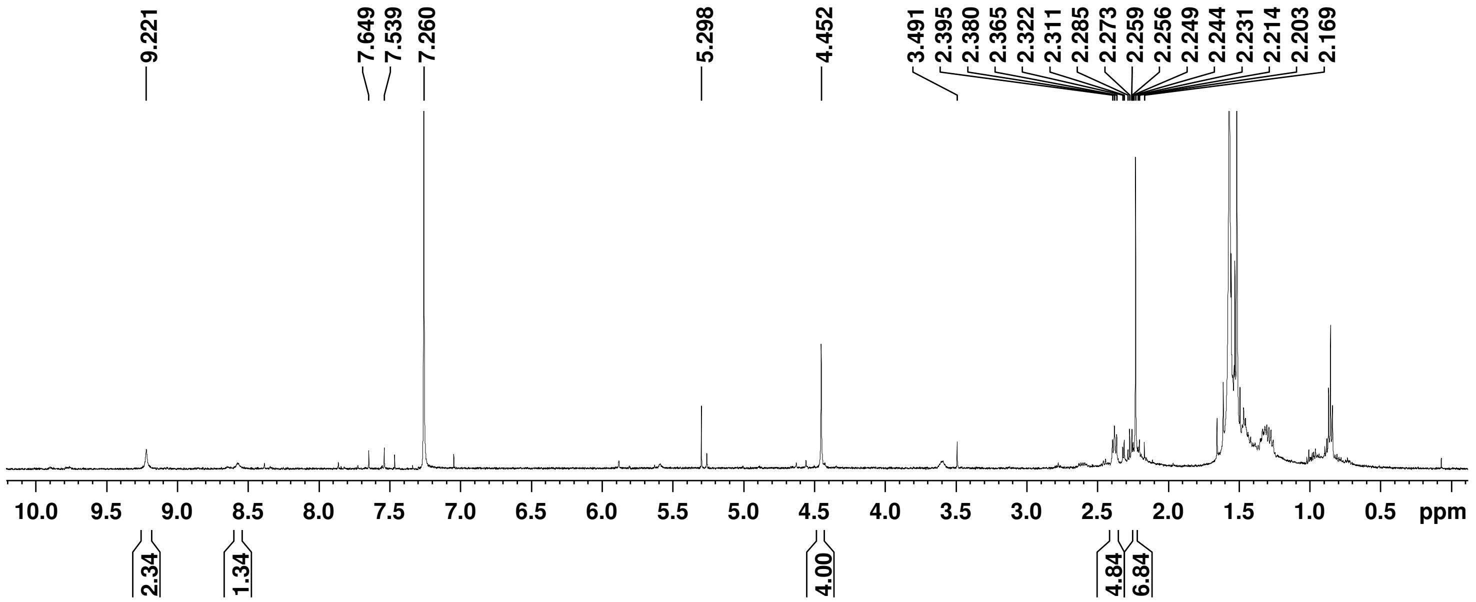

Figure S27. 500 MHz proton NMR spectrum of crude tripyrrane **32** in $CDCl_3$.

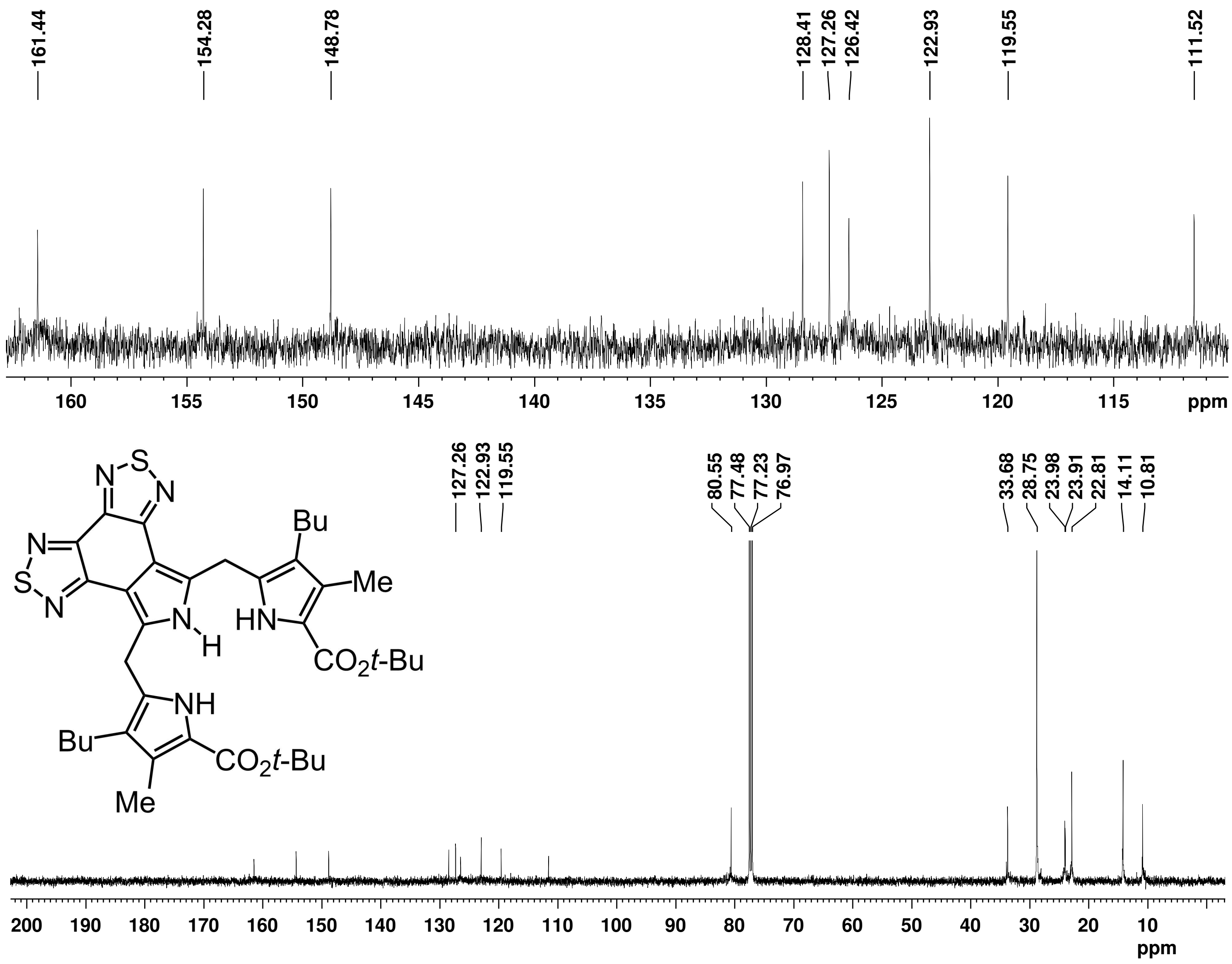

Figure S28. 125 MHz $^{13}C\{^1H\}$ NMR spectrum of crude tripyrrane **32** in $CDCl_3$.

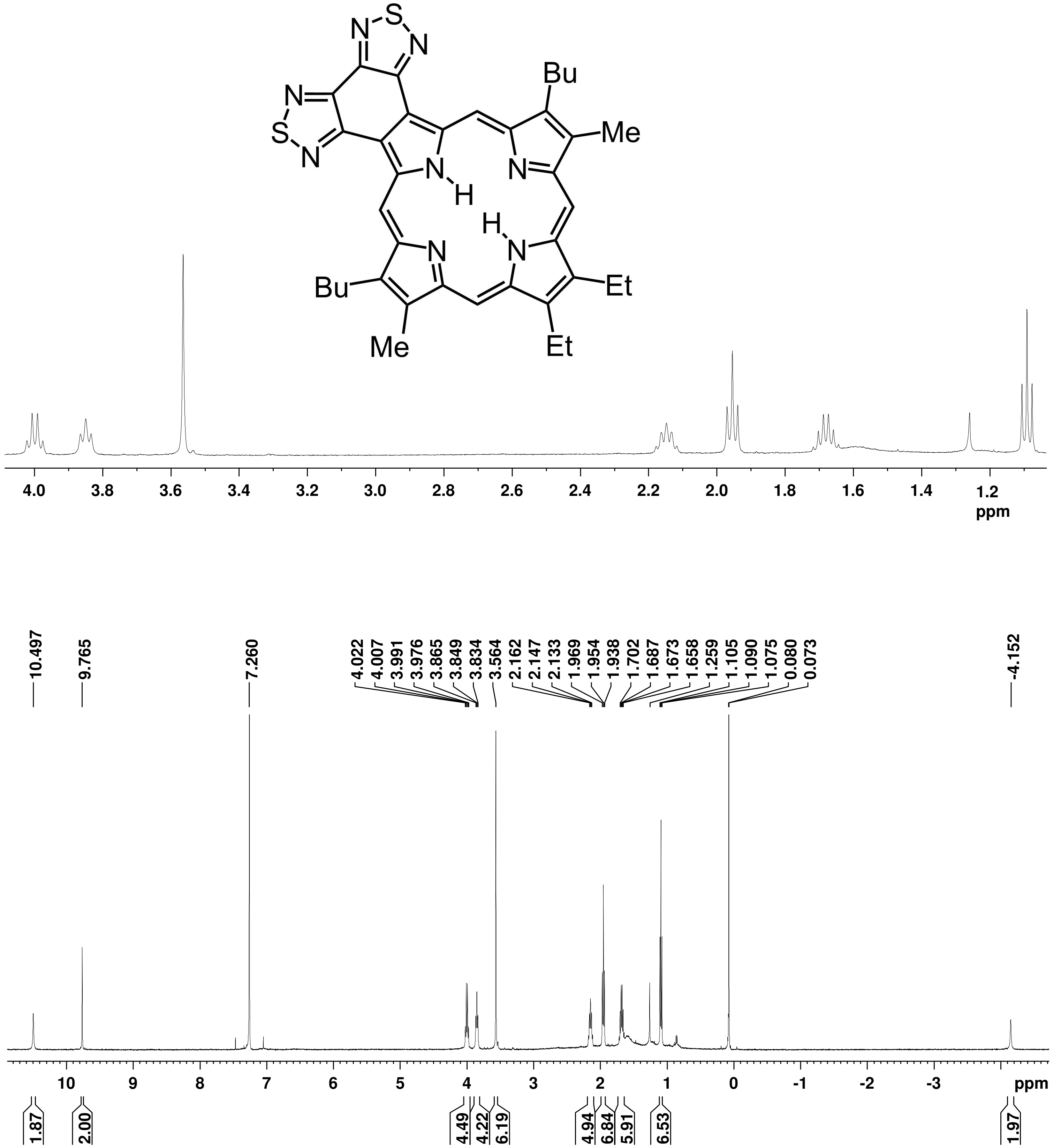

Figure S29. 500 MHz proton NMR spectrum of bis(thiadiazolo)benzoporphyrin **33** in $CDCl_3$ at 29 °C.

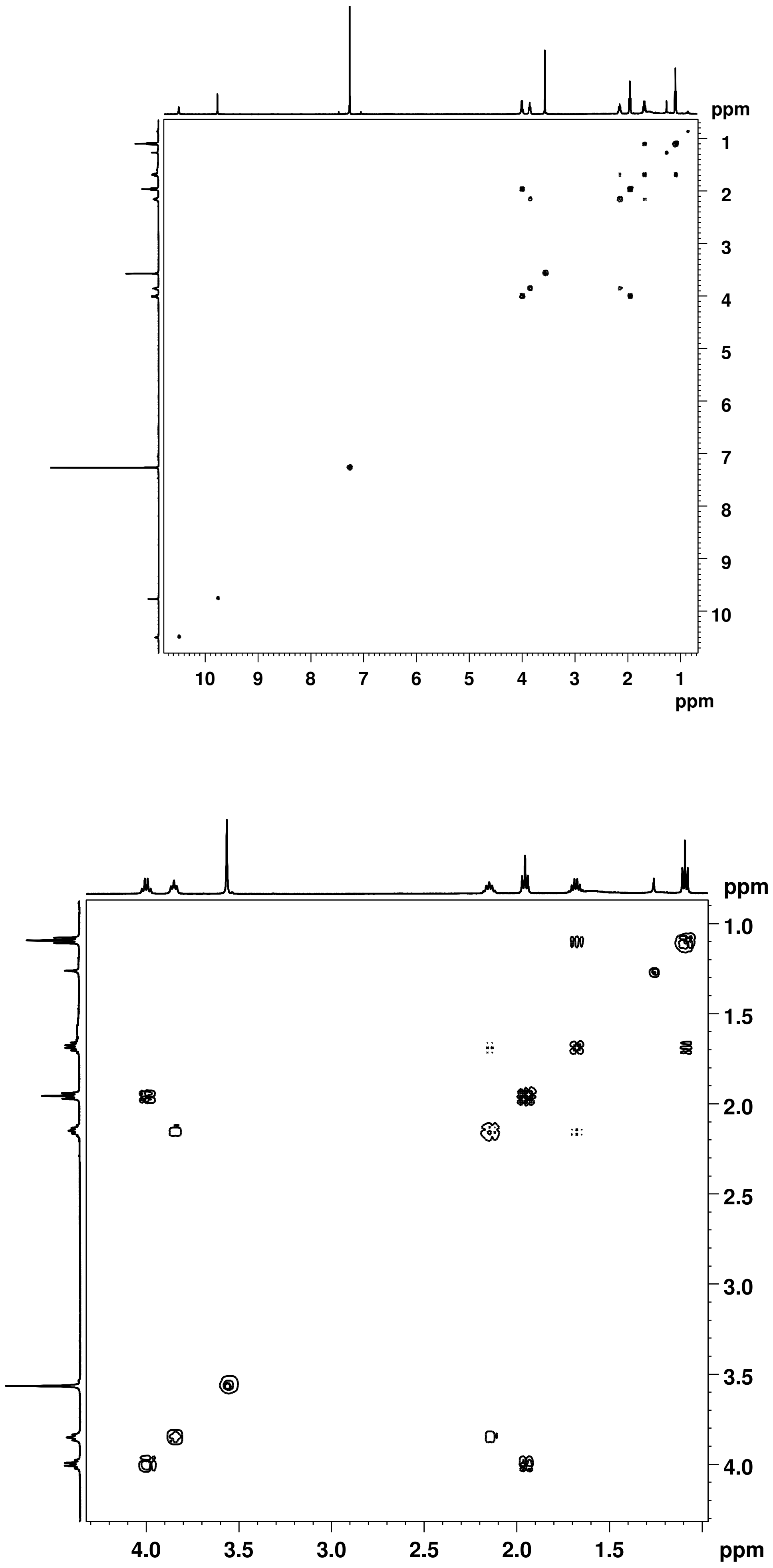

Figure S30. ^{1}H-^{1}H COSY NMR spectrum of bis(thiadiazolo)benzoporphyrin **33** in $CDCl_3$ at 29 °C.

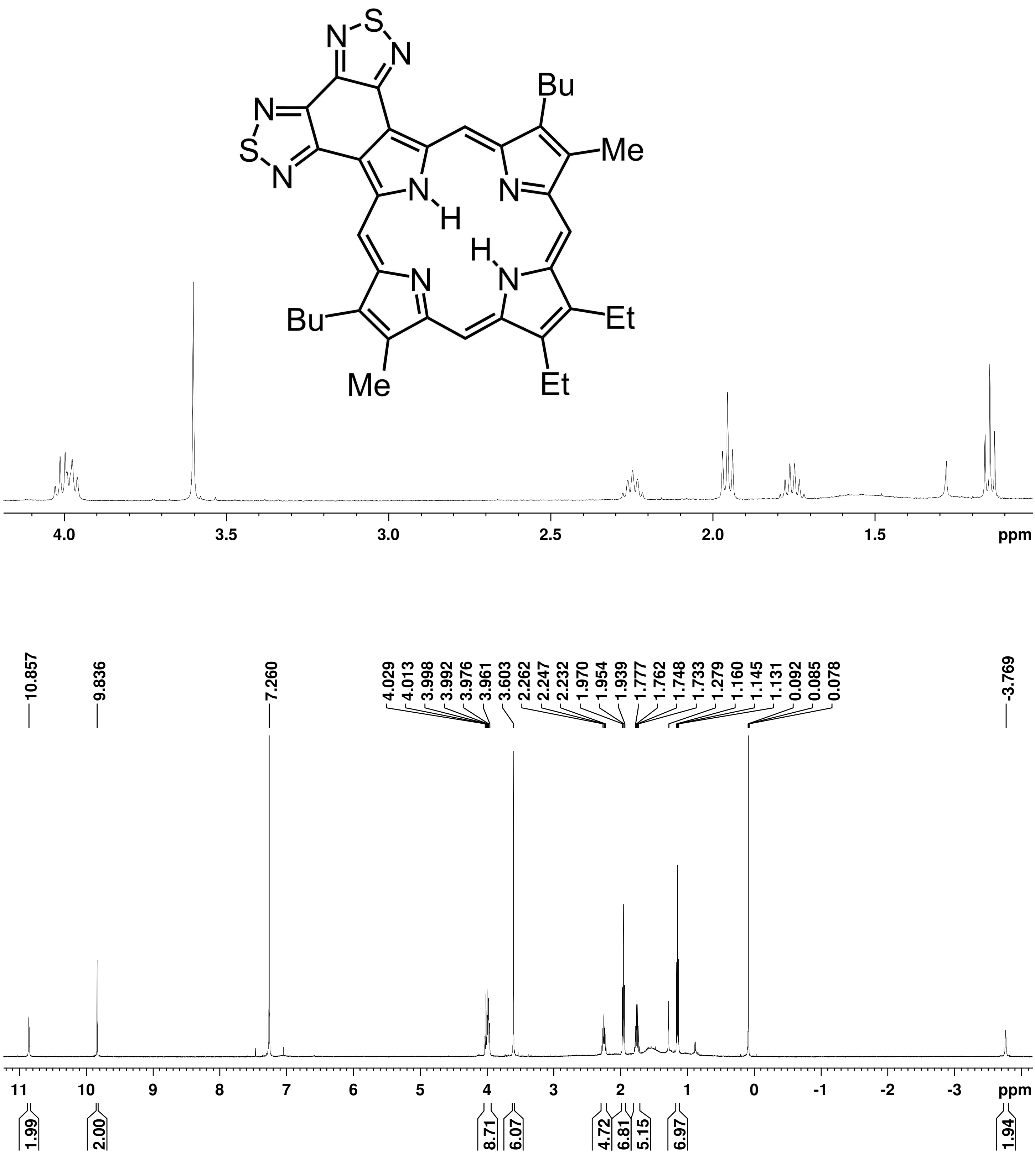

Figure S31. 500 MHz proton NMR spectrum of bis(thiadiazolo)benzoporphyrin **33** in $CDCl_3$ at 55 °C.

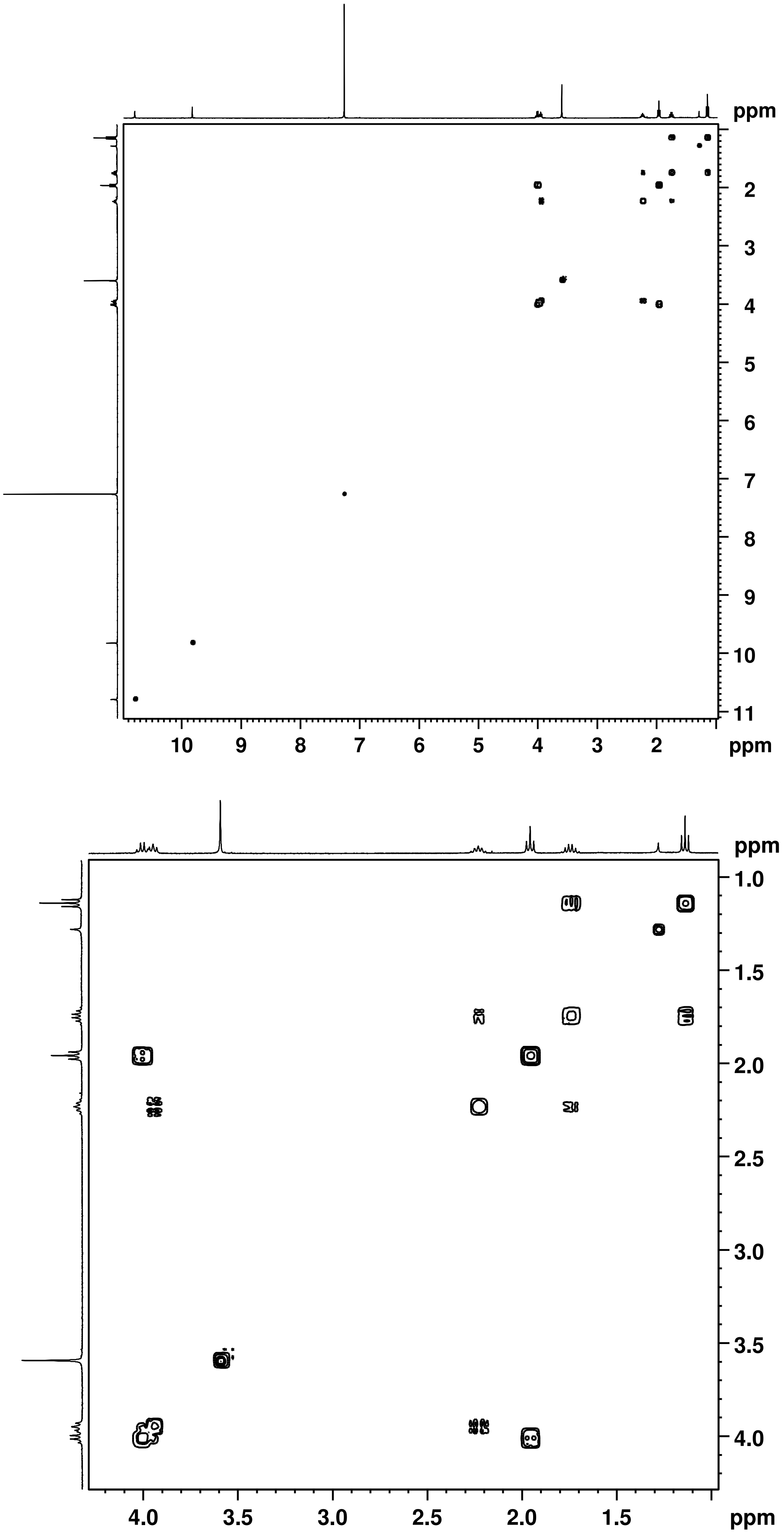

Figure S32. ^{1}H-^{1}H COSY NMR spectrum of bis(thiadiazolo)benzoporphyrin **33** in $CDCl_3$ at 55 °C.

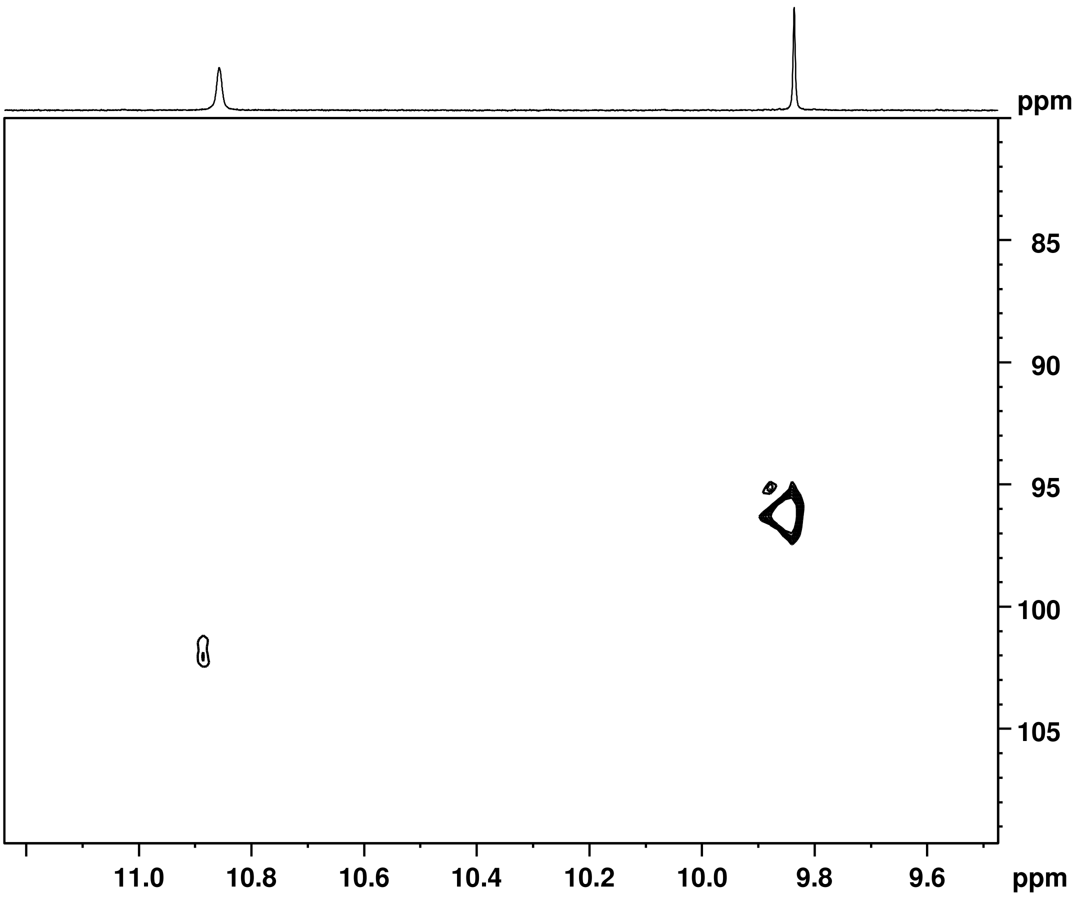

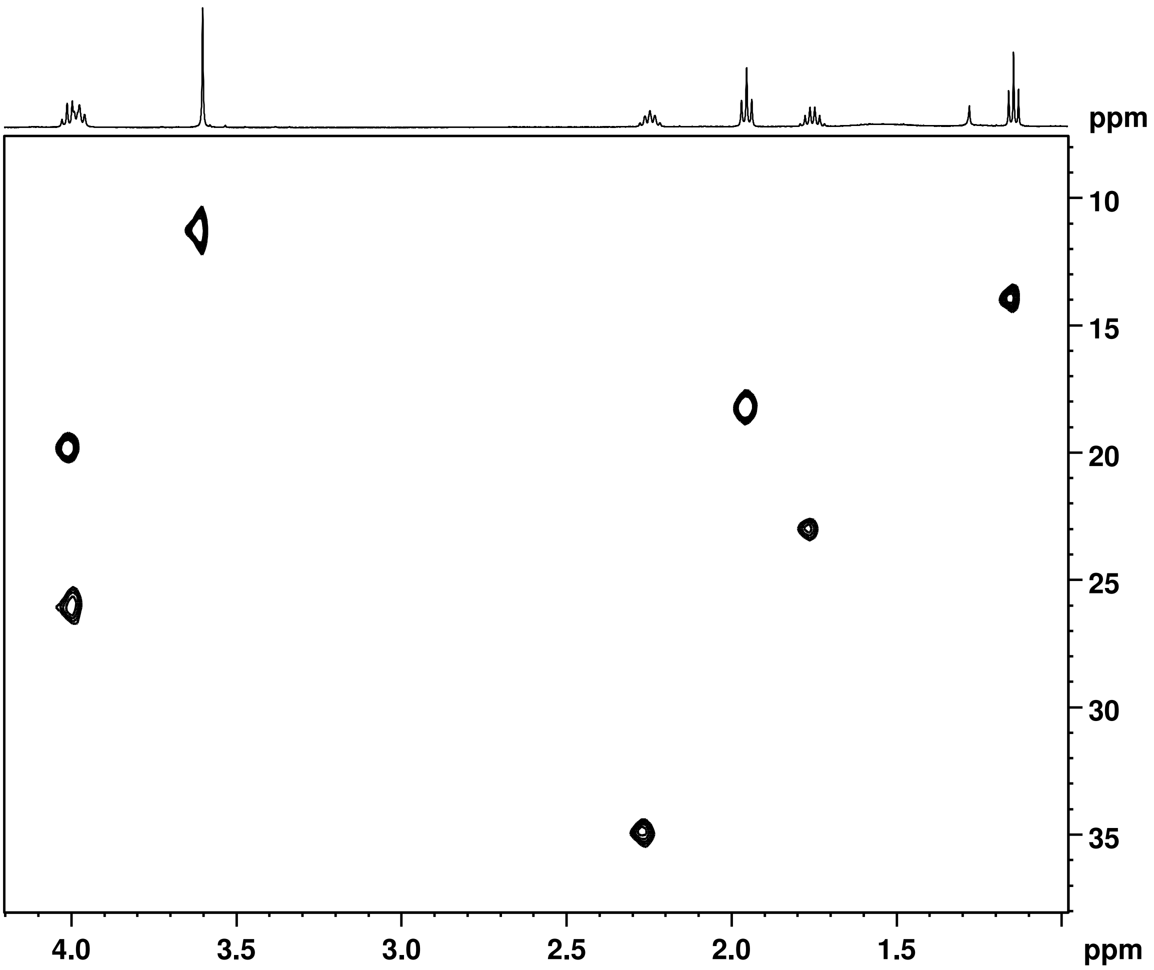

Figure S33. HSQC NMR spectrum of bis(thiadiazolo)benzoporphyrin **33** in $CDCl_3$ at 55 ^oC.

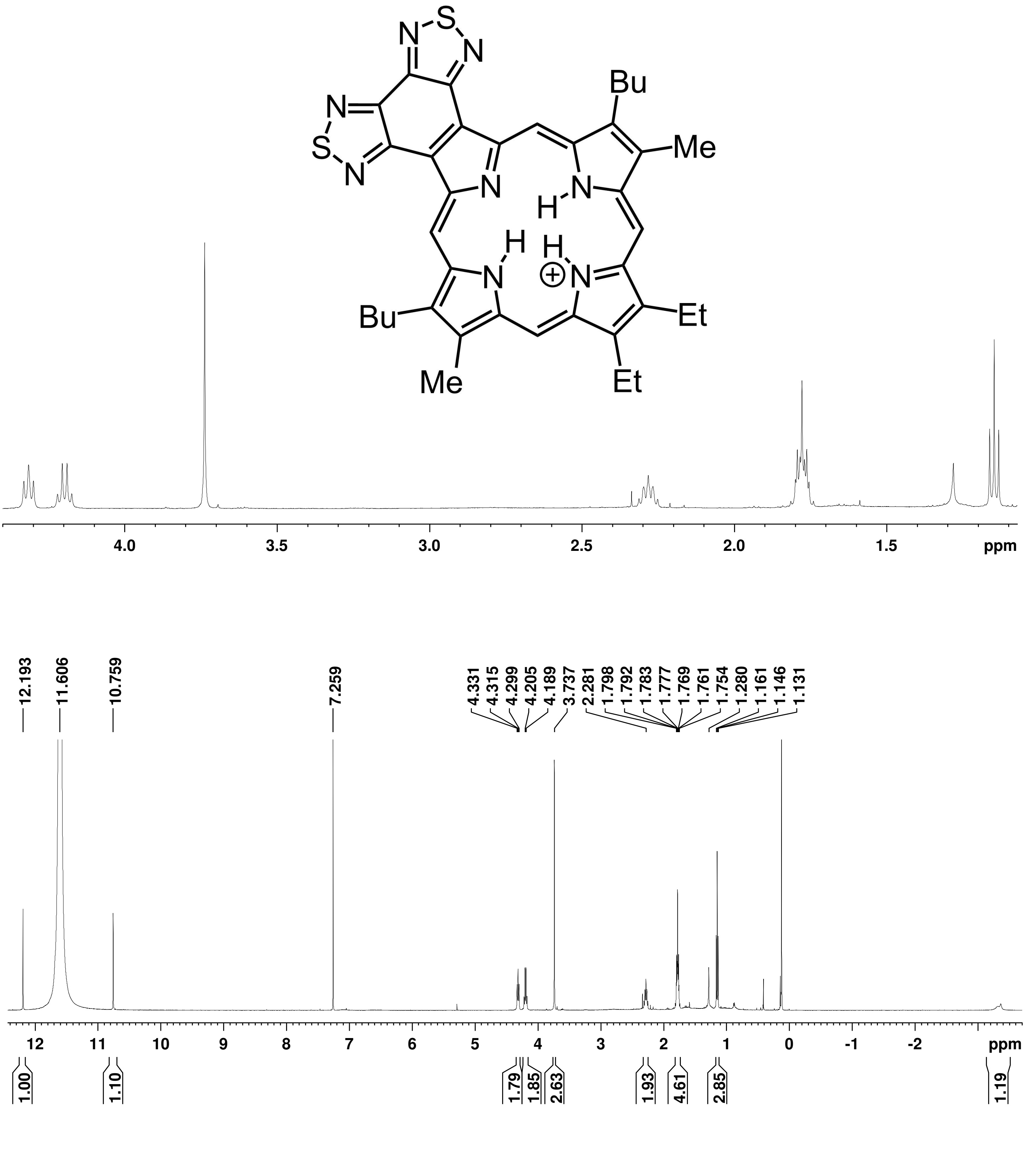

Figure S34. 500 MHz proton NMR spectrum of bis(thiadiazolo)benzoporphyrin **33** in TFA-$CDCl_3$.

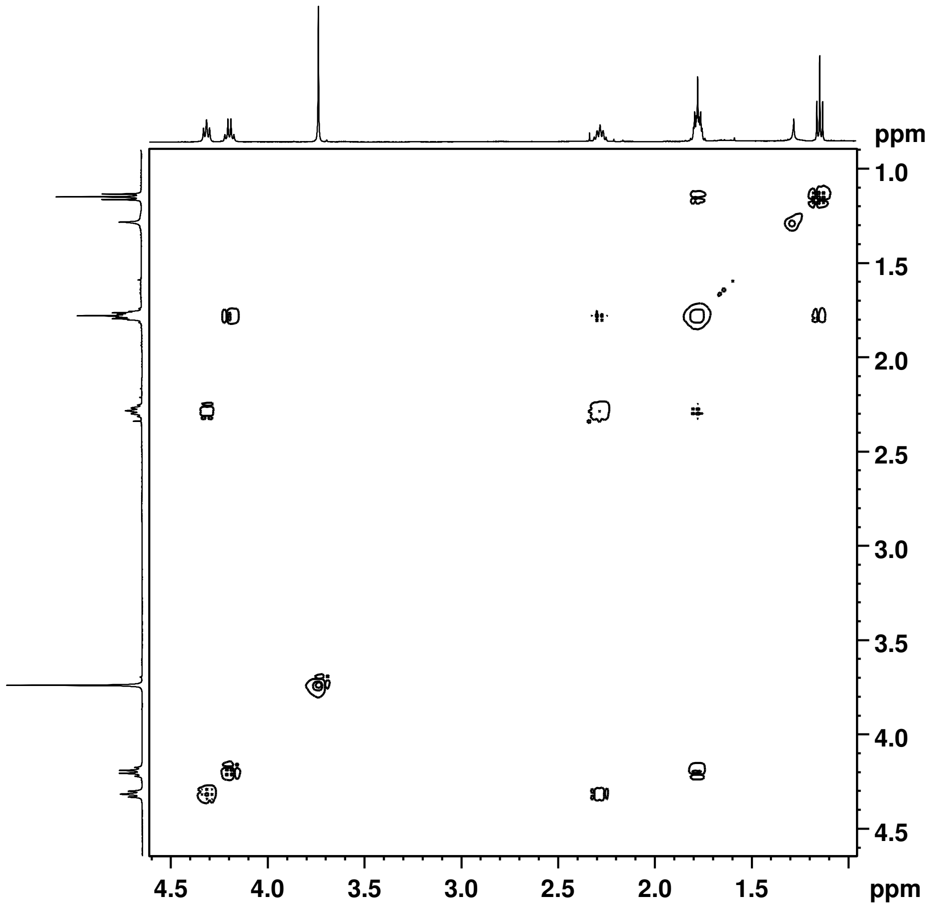

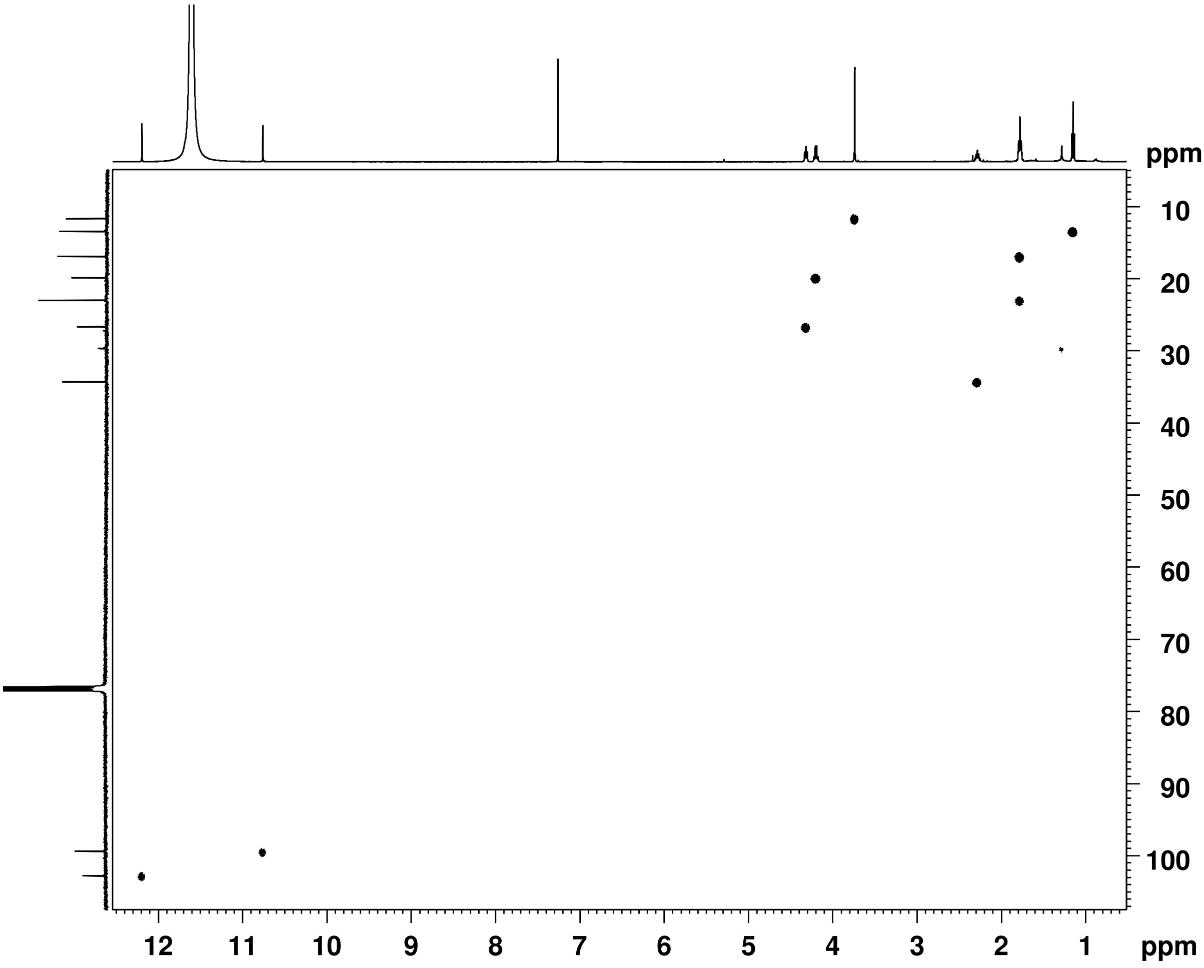

Figure S35. ^{1}H-^{1}H COSY (above) and HSQC (below) NMR spectra of **33** in TFA-$CDCl_3$.

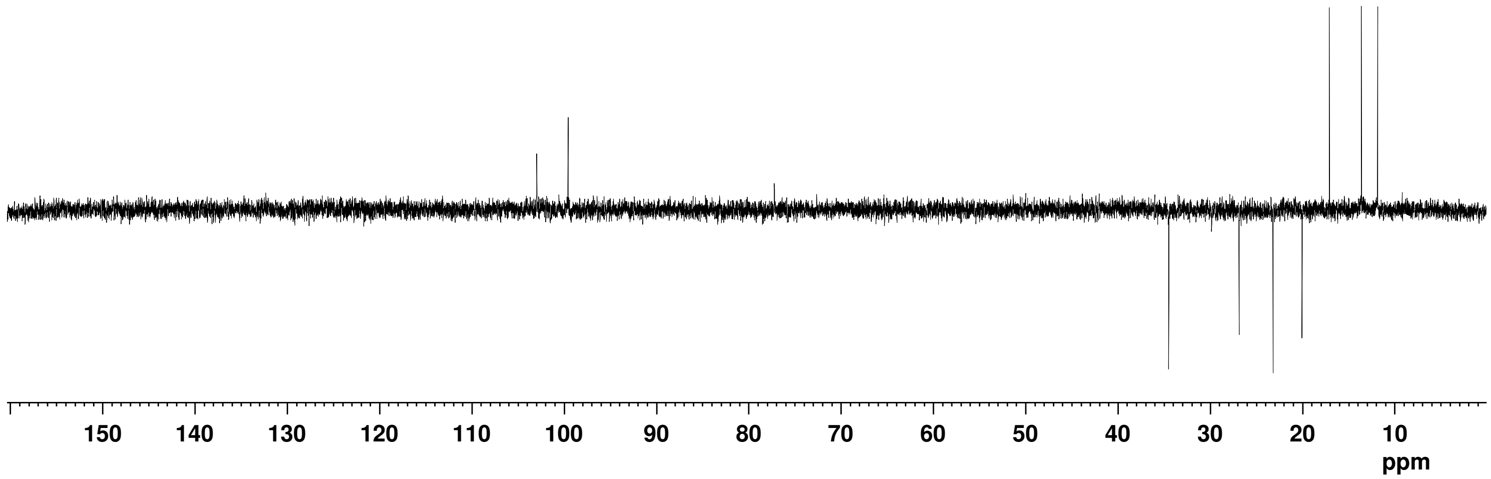

Figure S36. DEPT-135 NMR spectrum of bis(thiadiazolo)benzoporphyrin **33** in TFA-$CDCl_3$.

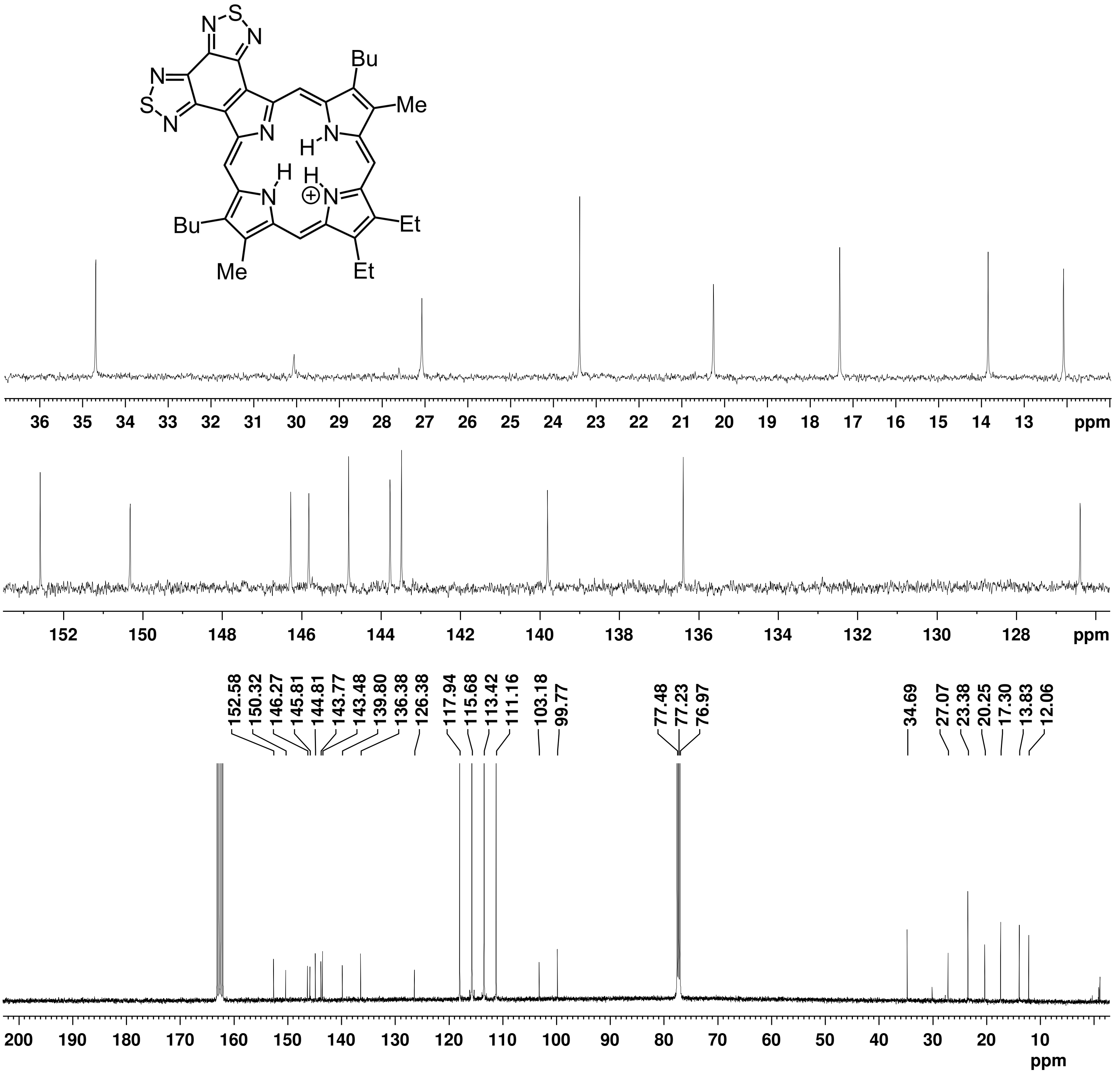

Figure S37. 125 Mz $^{13}C\{^1H\}$ NMR spectrum of bis(thiadiazolo)benzoporphyrin **33** in TFA-$CDCl_3$.

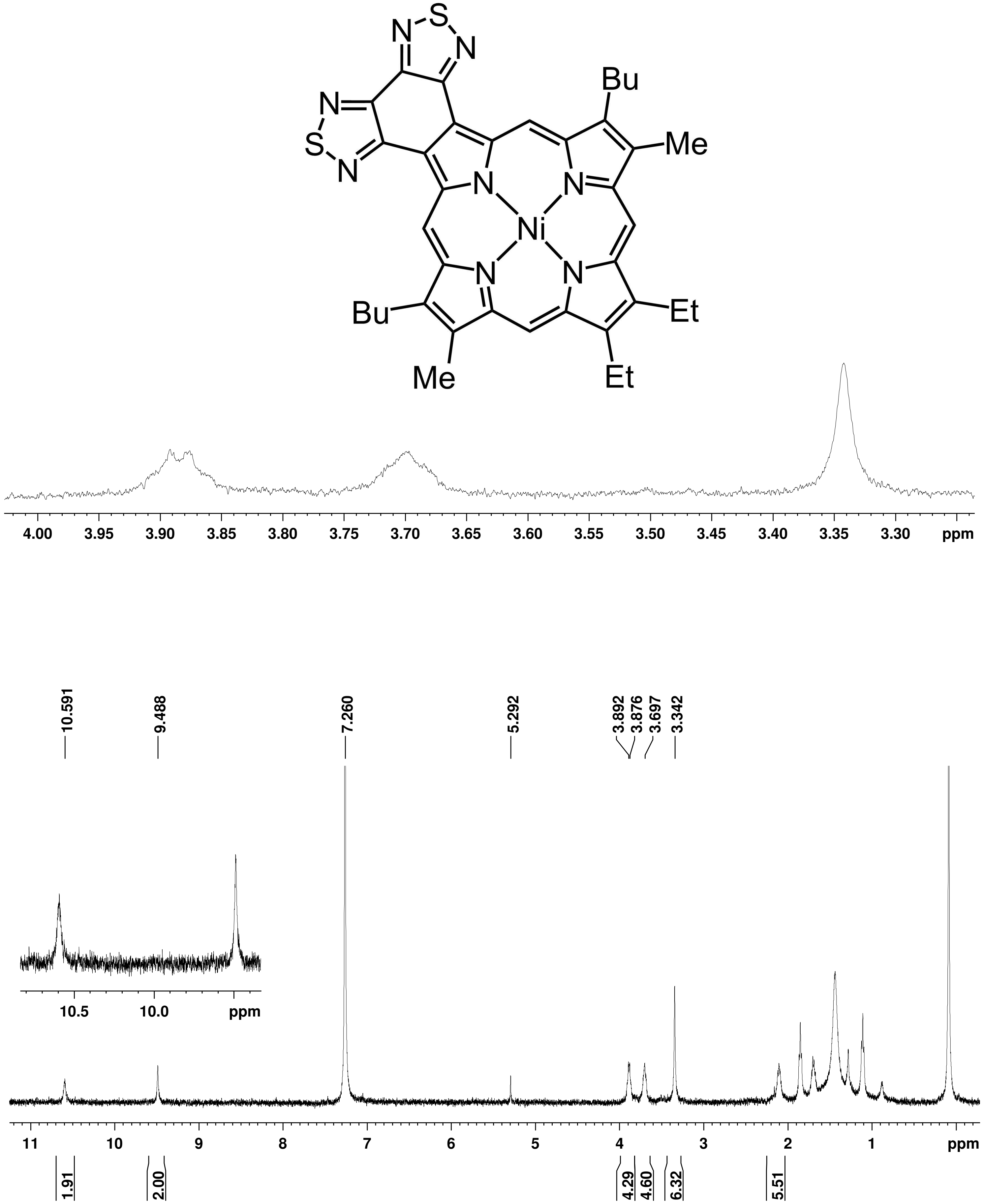

Figure S38. 500 MHz proton NMR spectrum of **33Ni** in $CDCl_3$ at 55 ^{o}C.

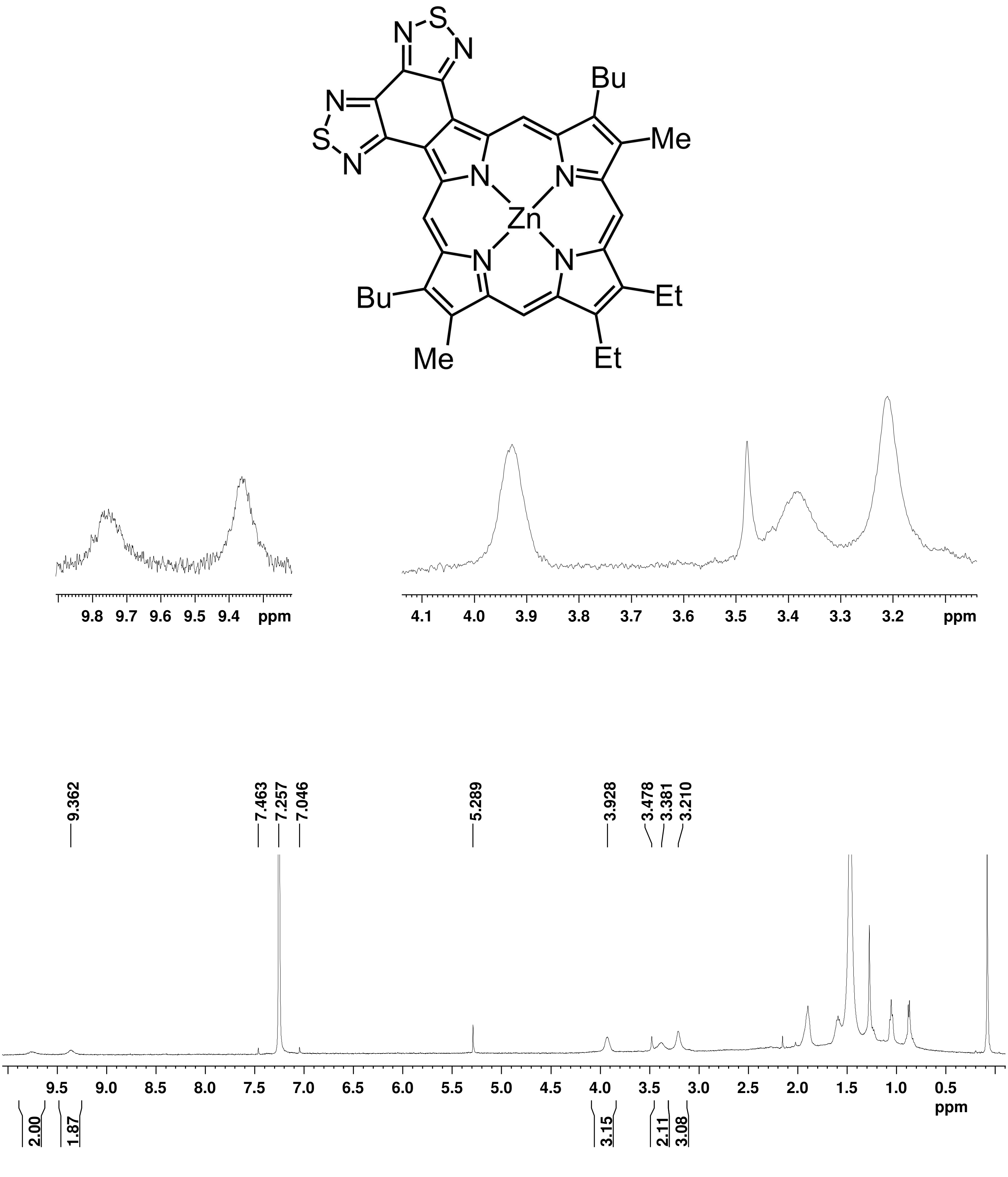

Figure S39. 500 MHz proton NMR spectrum of **33Zn** in $CDCl_3$ at 55 °C. The broad peaks result from the low solubility of the zinc complex.

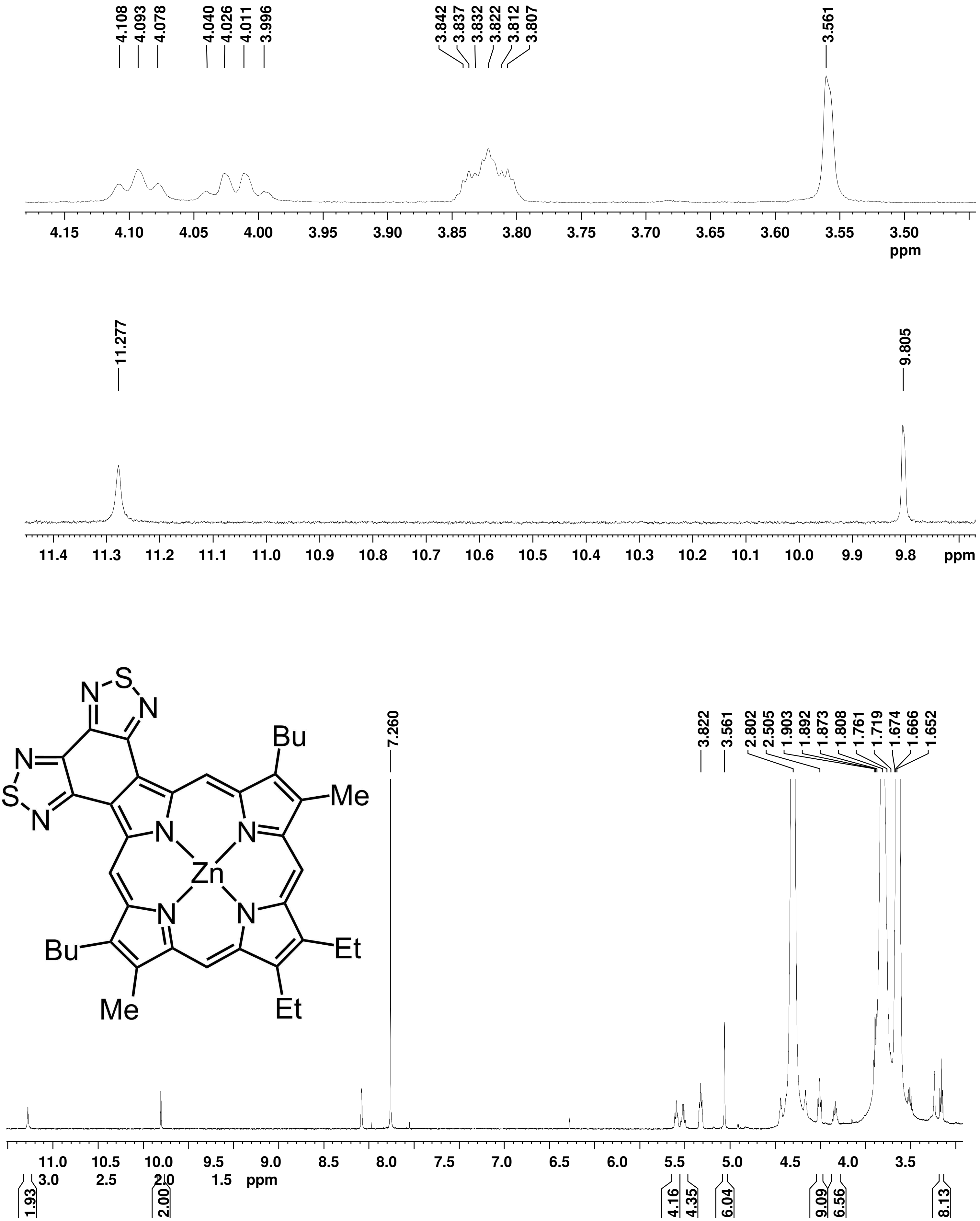

Figure S40. 500 MHz proton NMR spectrum of **33Zn** in $CDCl_3$ awith one drop of pyrrolidine

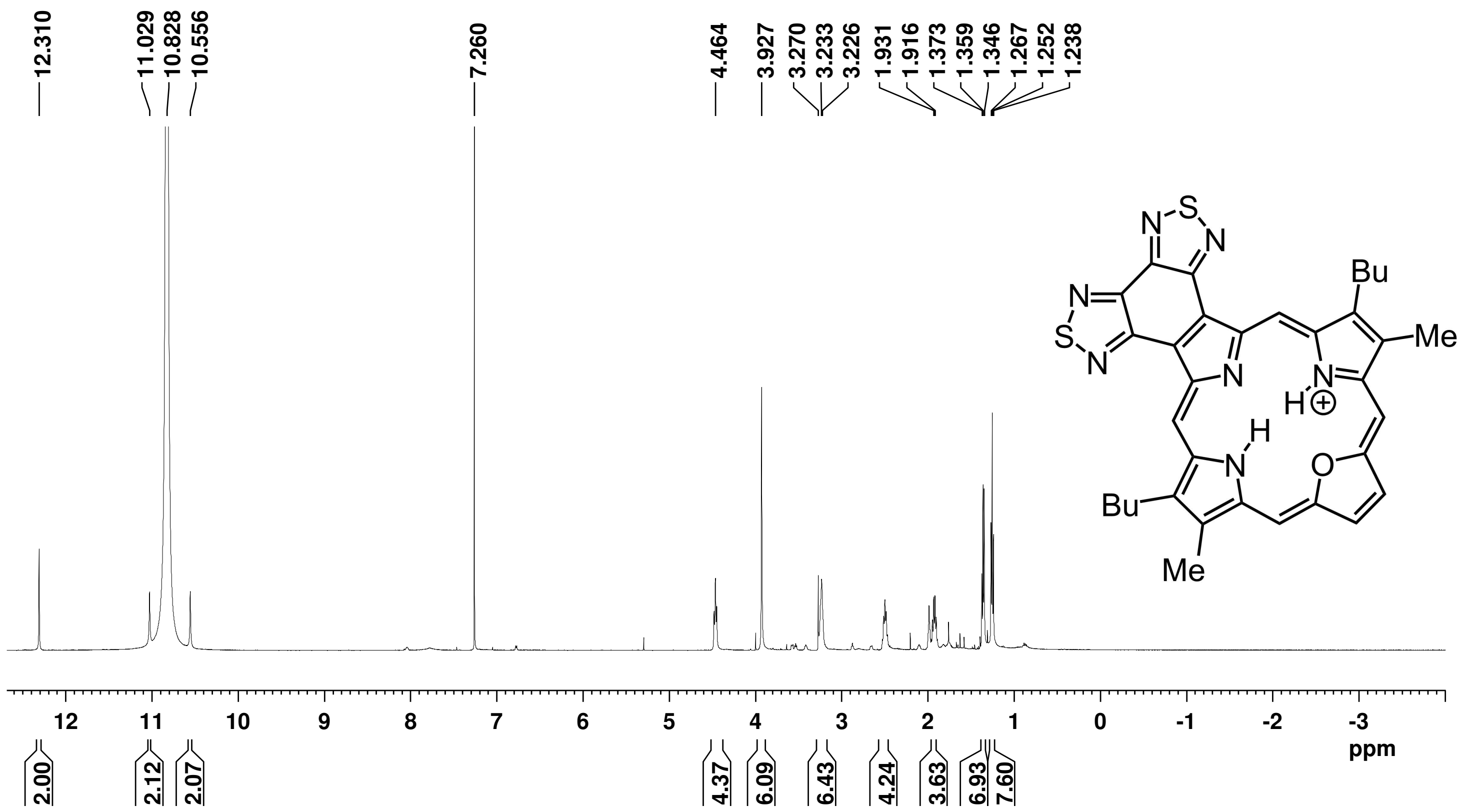

Figure 41. 500 MHz proton NMR spectrum of oxaporphyrin **35a** in TFA-$CDCl_3$.

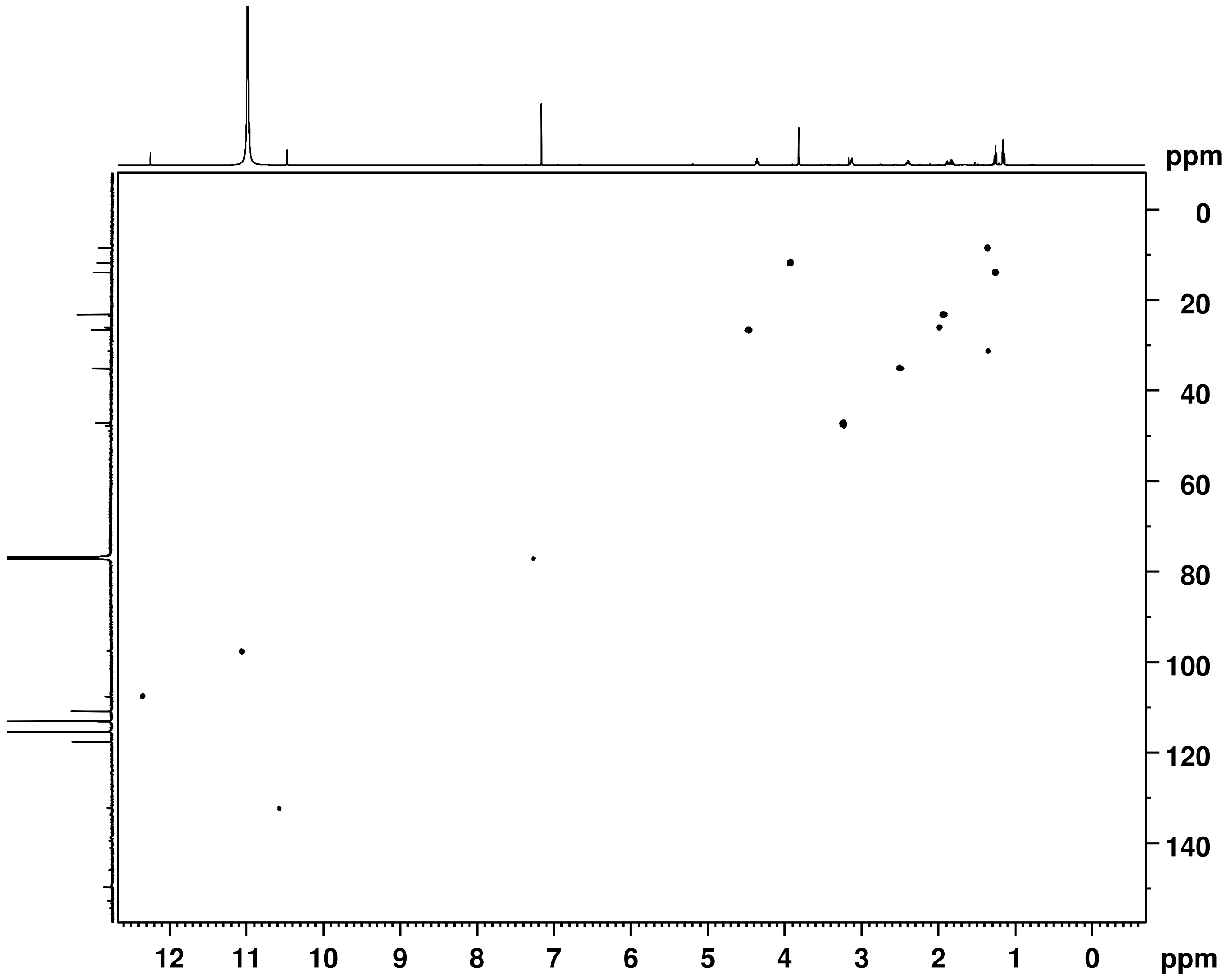

Figure 42. HSQC NMR spectrum of oxaporphyrin **35a** in TFA-$CDCl_3$.

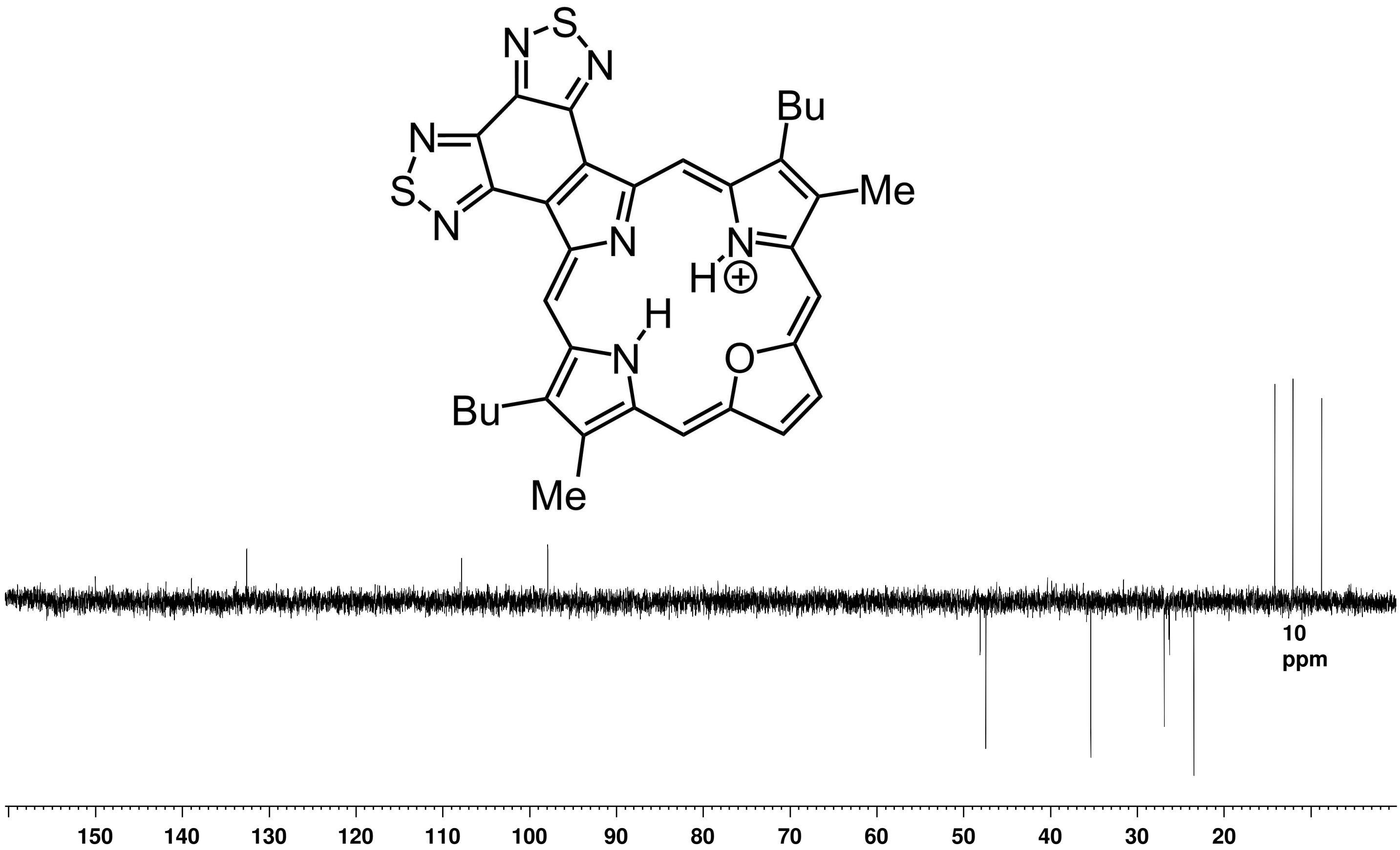

Figure 43. DEPT-135 NMR spectrum of oxaporphyrin **35a** in TFA-$CDCl_3$.

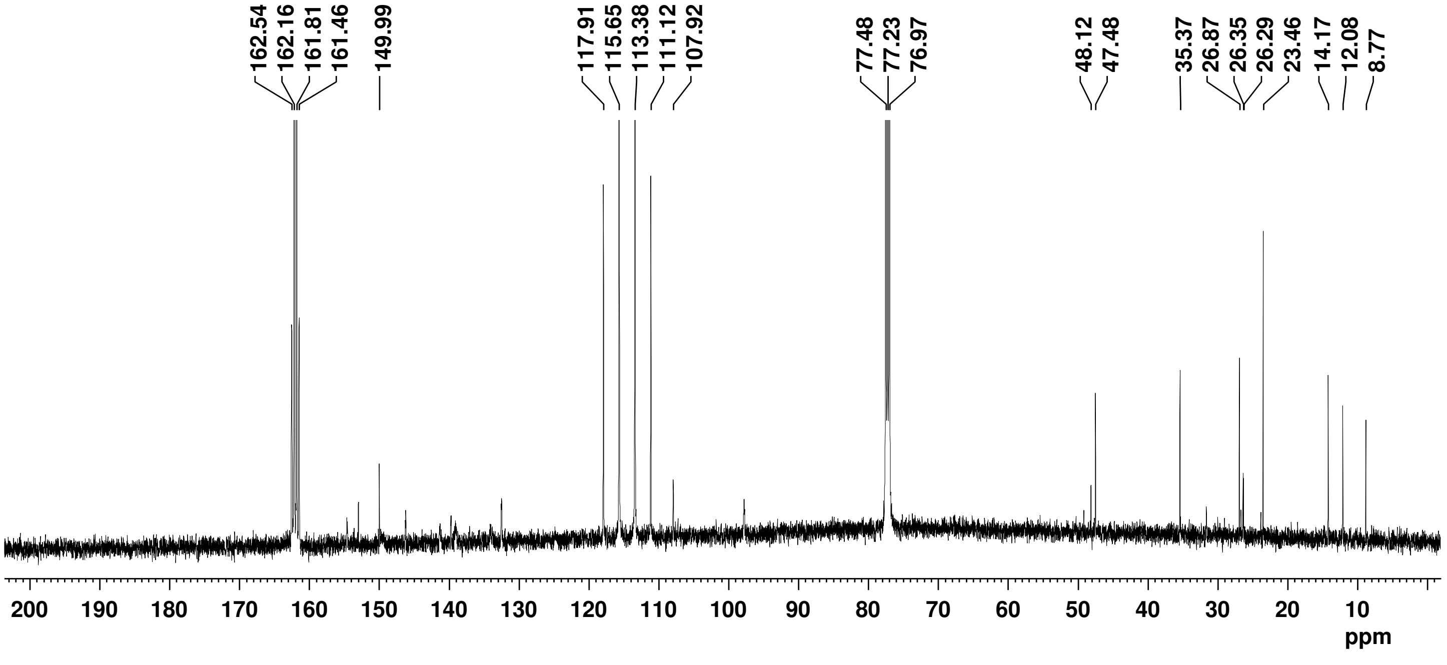

Figure 44. 125 MHz carbon-13 NMR spectrum of oxaporphyrin **35a** in TFA-$CDCl_3$.

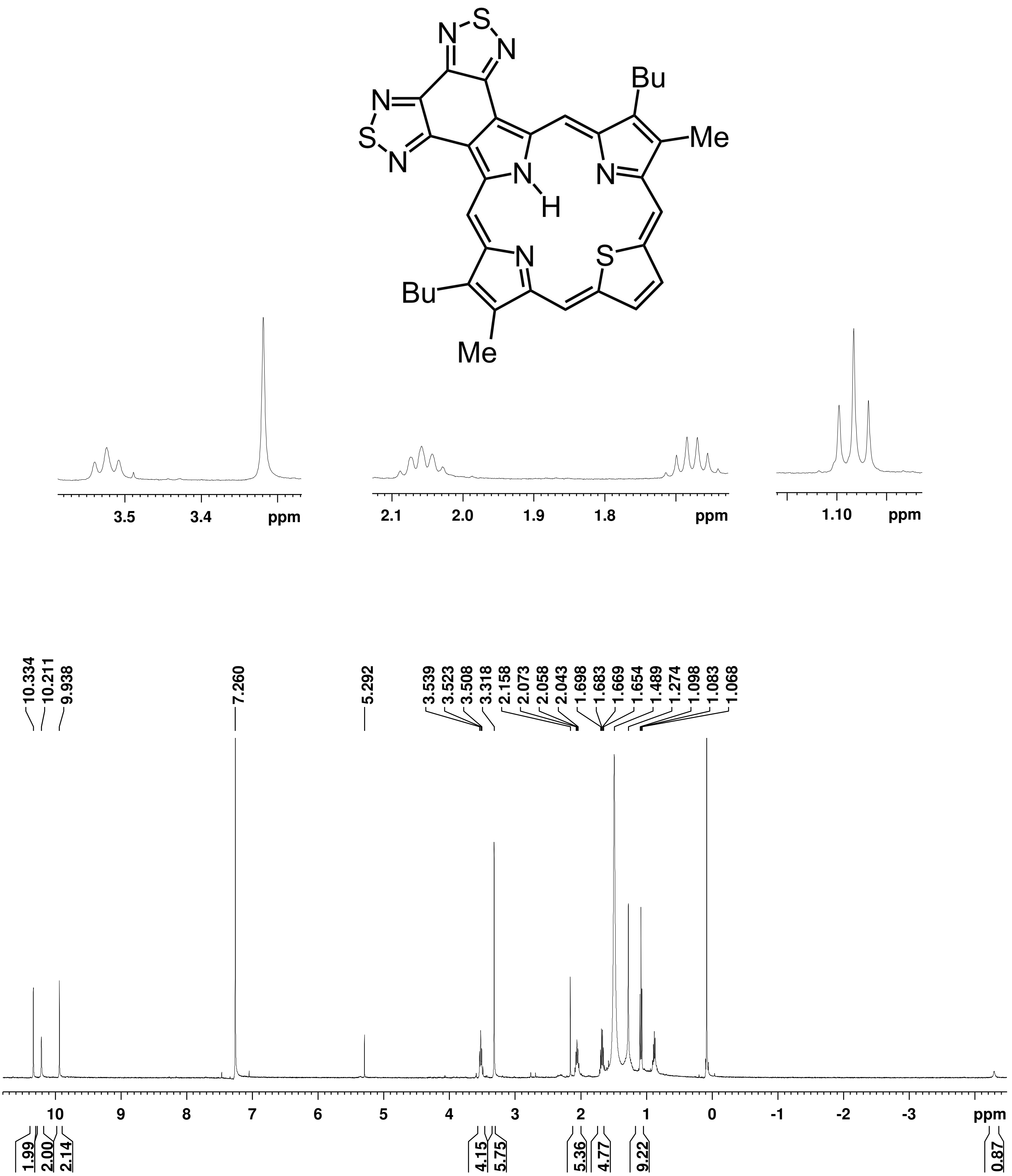

Figure 45. 500 MHz proton NMR spectrum of thiaporphyrin **35b** in $CDCl_3$ at 50 ^{o}C.

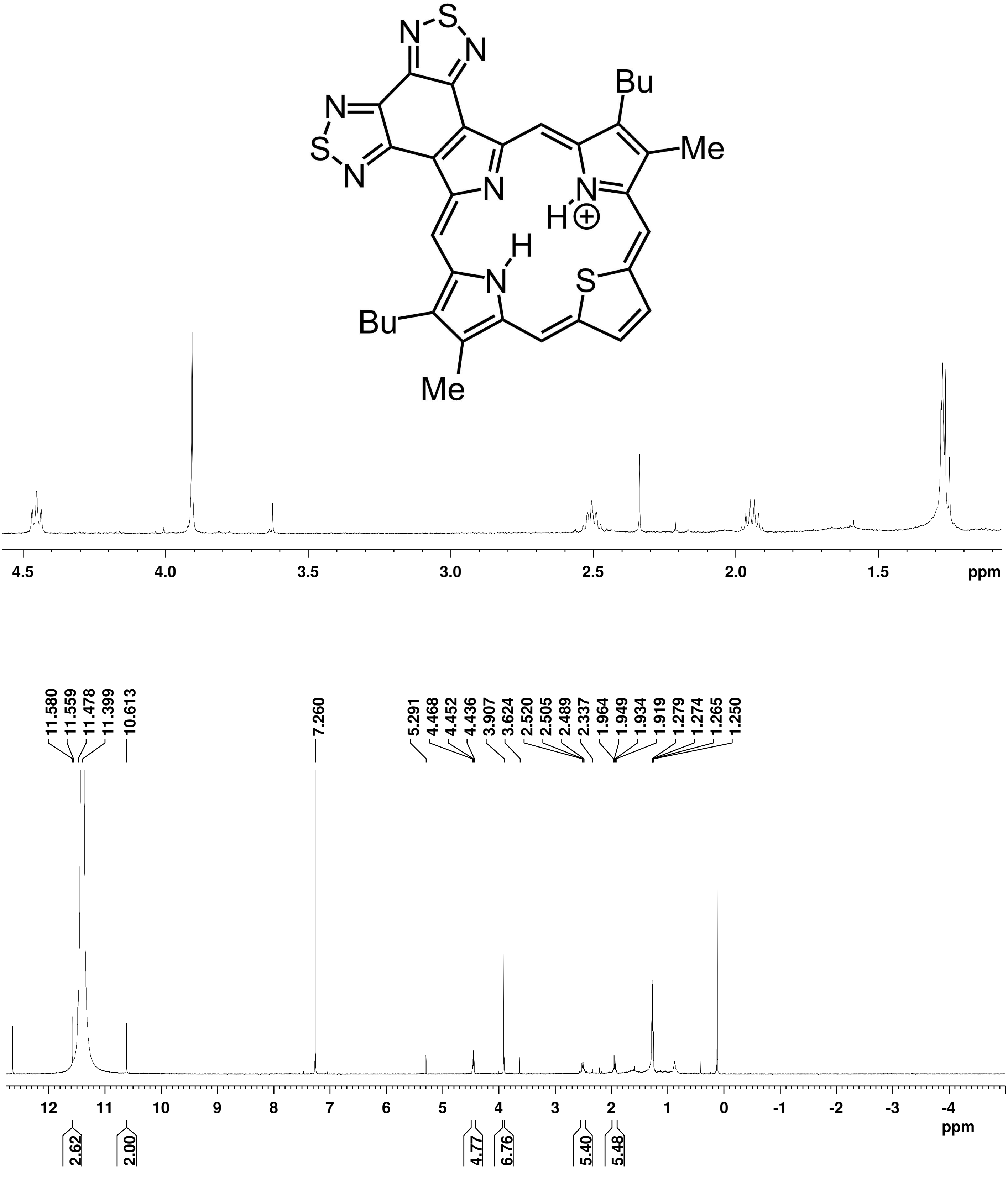

Figure 46. 500 MHz proton NMR spectrum of thiaporphyrin **35b** in TFA-$CDCl_3$.

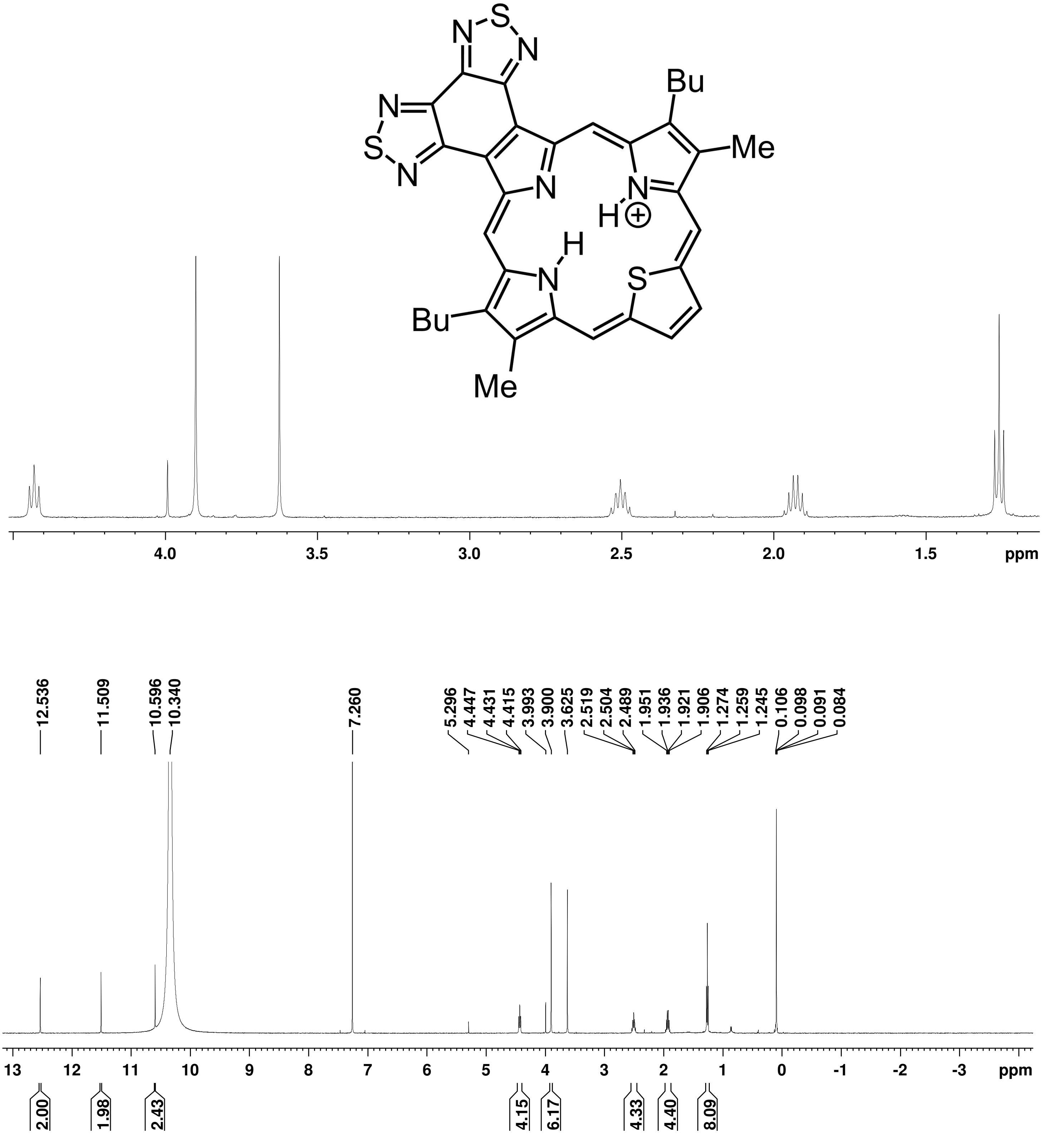

Figure 47. 500 MHz proton NMR spectrum of thiaporphyrin **35b** in $CDCl_3$ with a slightly lower concentration of TFA.

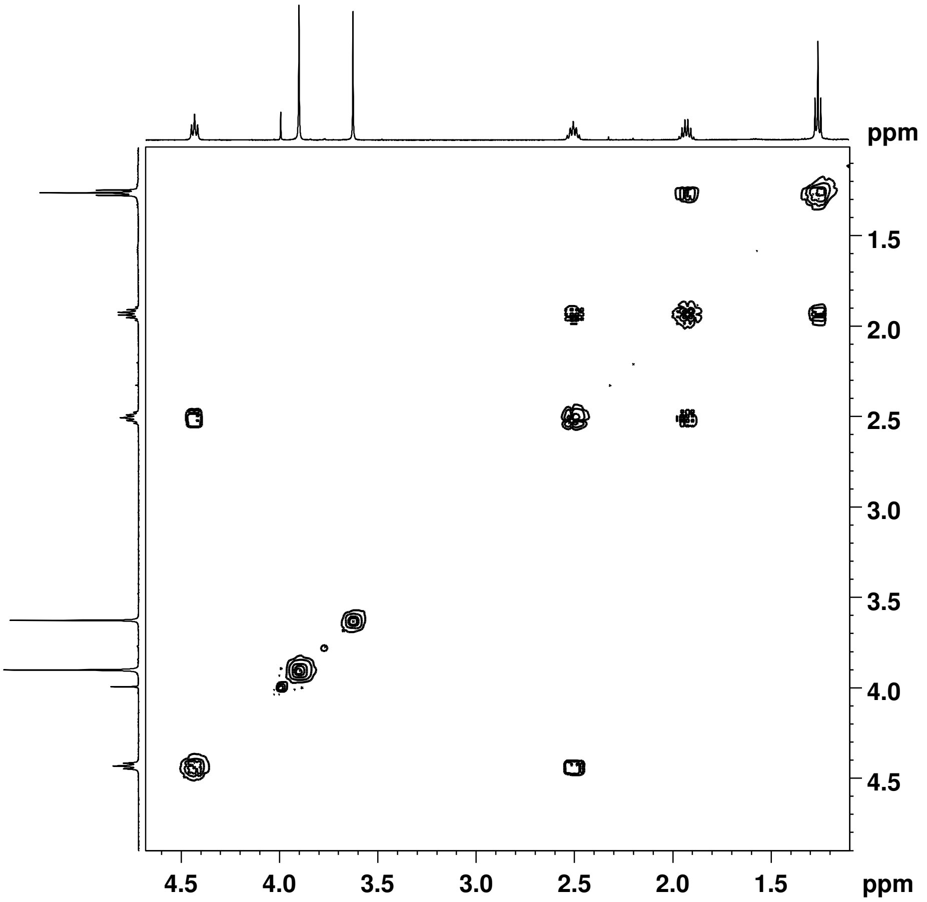

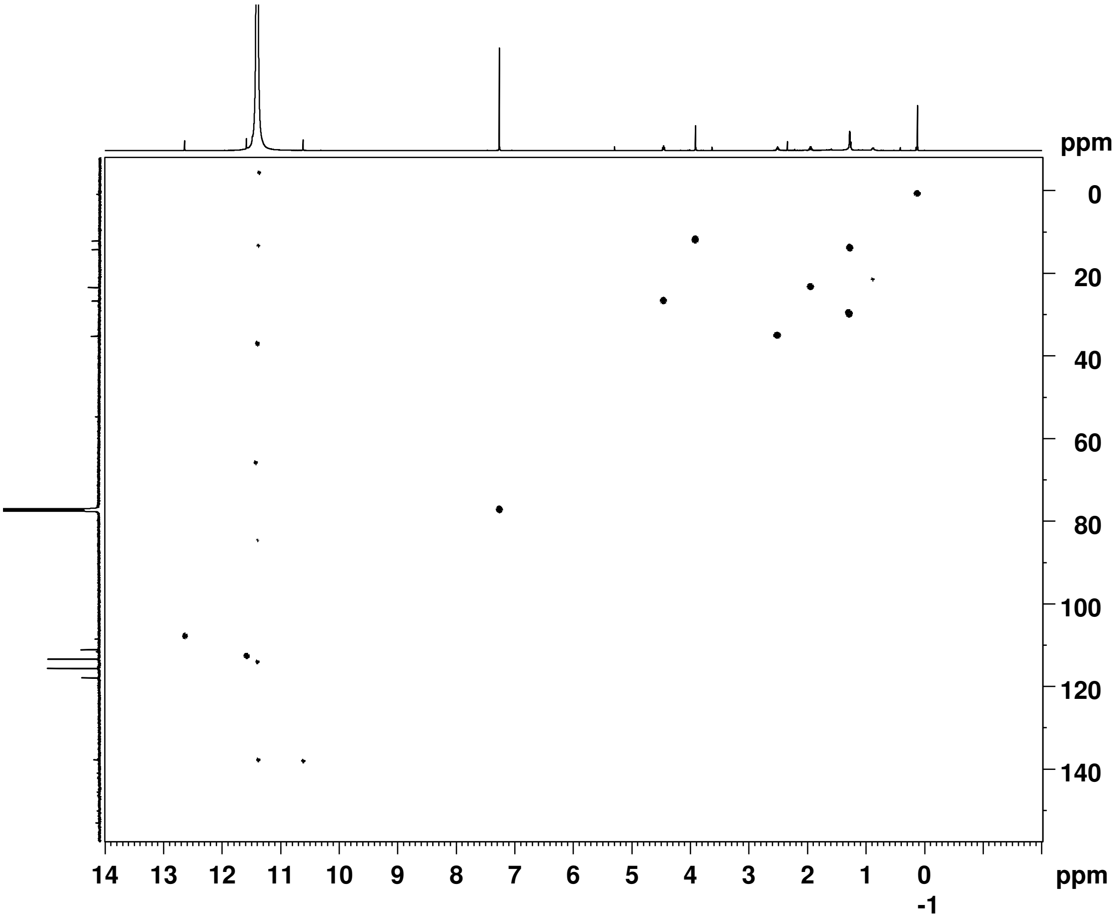

Figure 48. ^{1}H-^{1}H COSY (above) and HSQC (below) NMR spectra of thiaporphyrin **35b** in TFA-$CDCl_3$.

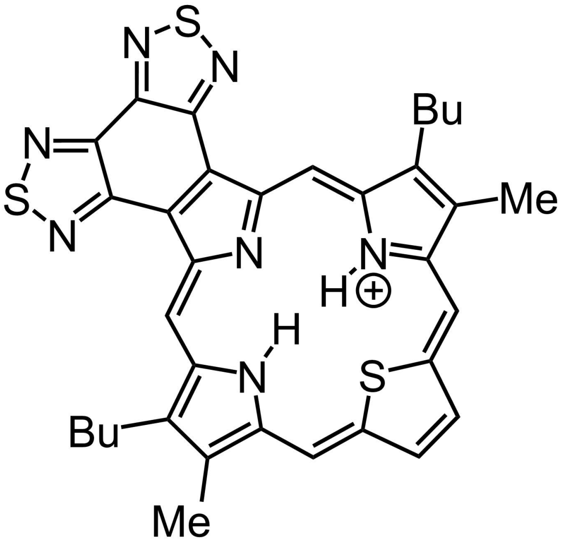

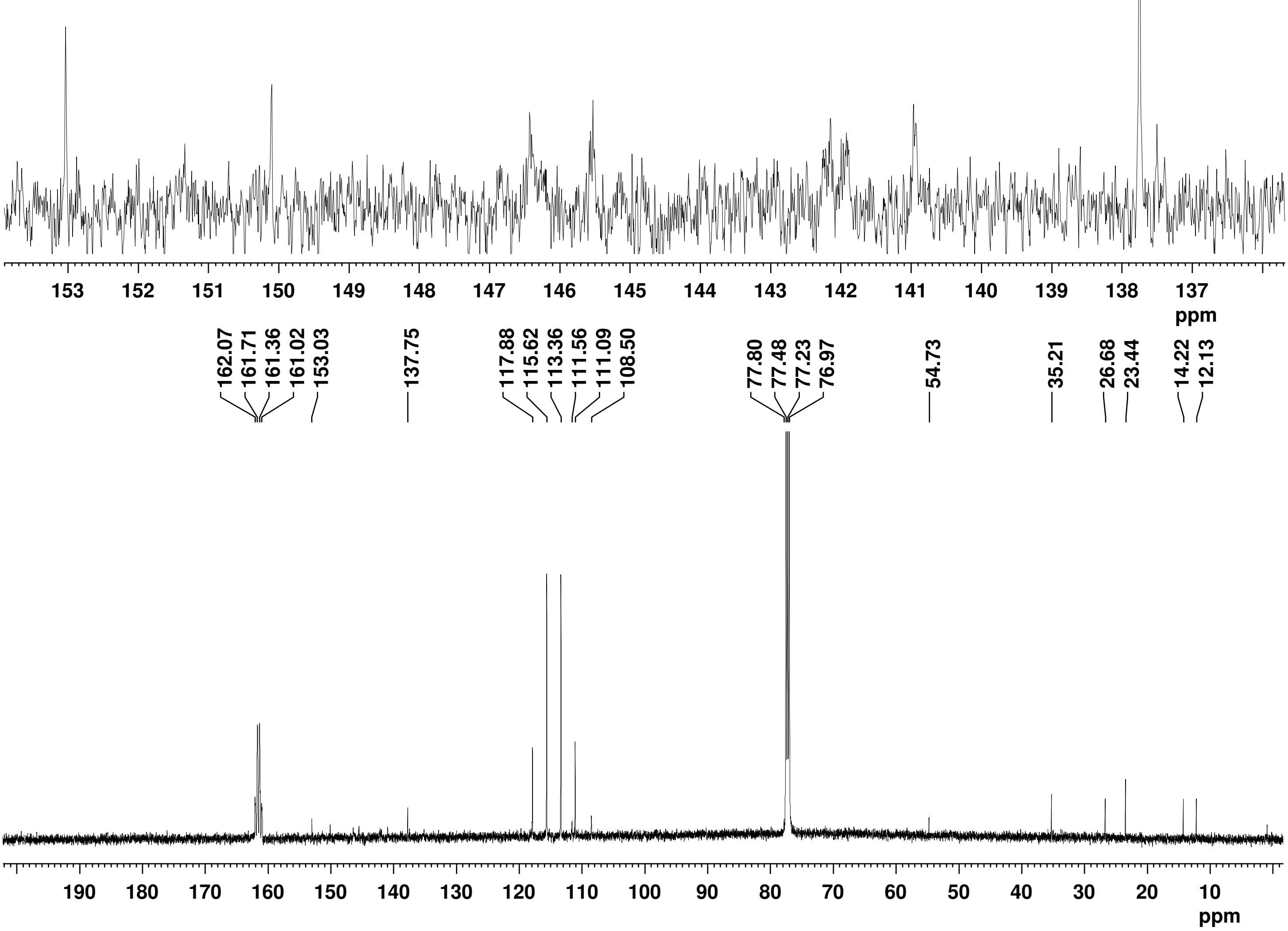

Figure 49. 125 MHz carbon-13 NMR spectrum of poorly soluble thiaporphyrin **35b** in TFA-$CDCl_3$.

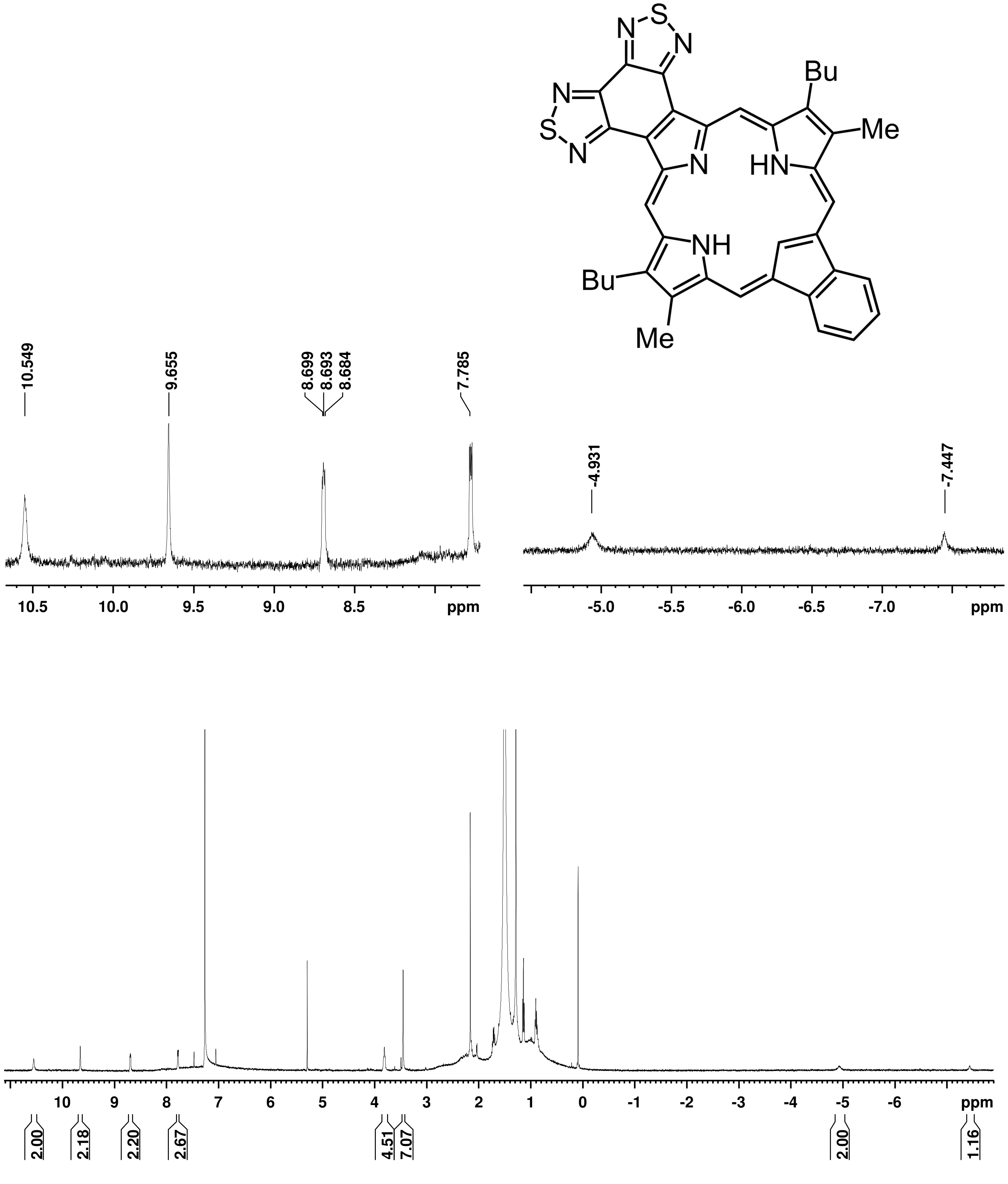

Scheme S50. 500 MHz NMR spectrum of highly insoluble carbaporphyrin **36** in $CDCl_3$ at 55 ^{o}C.

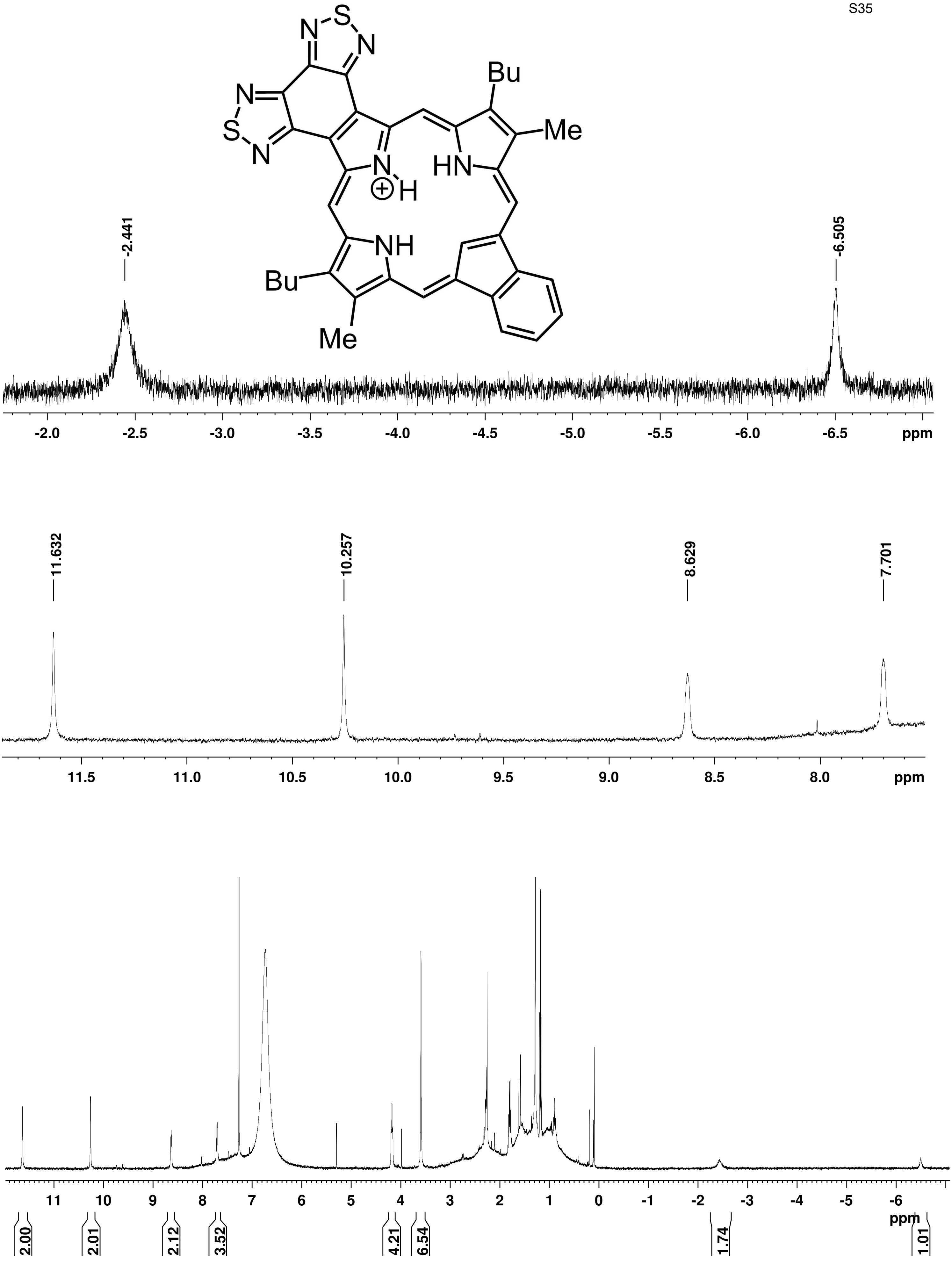

Scheme S51. 500 MHz NMR spectrum of carbaporphyrin **36** in TFA-$CDCl_3$.

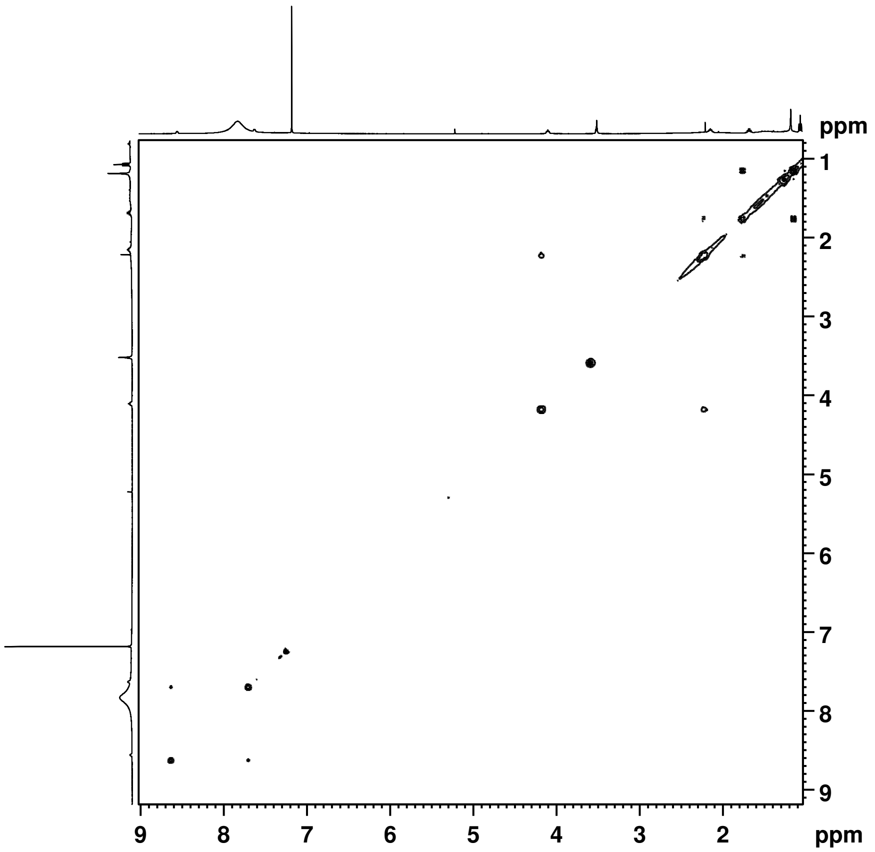

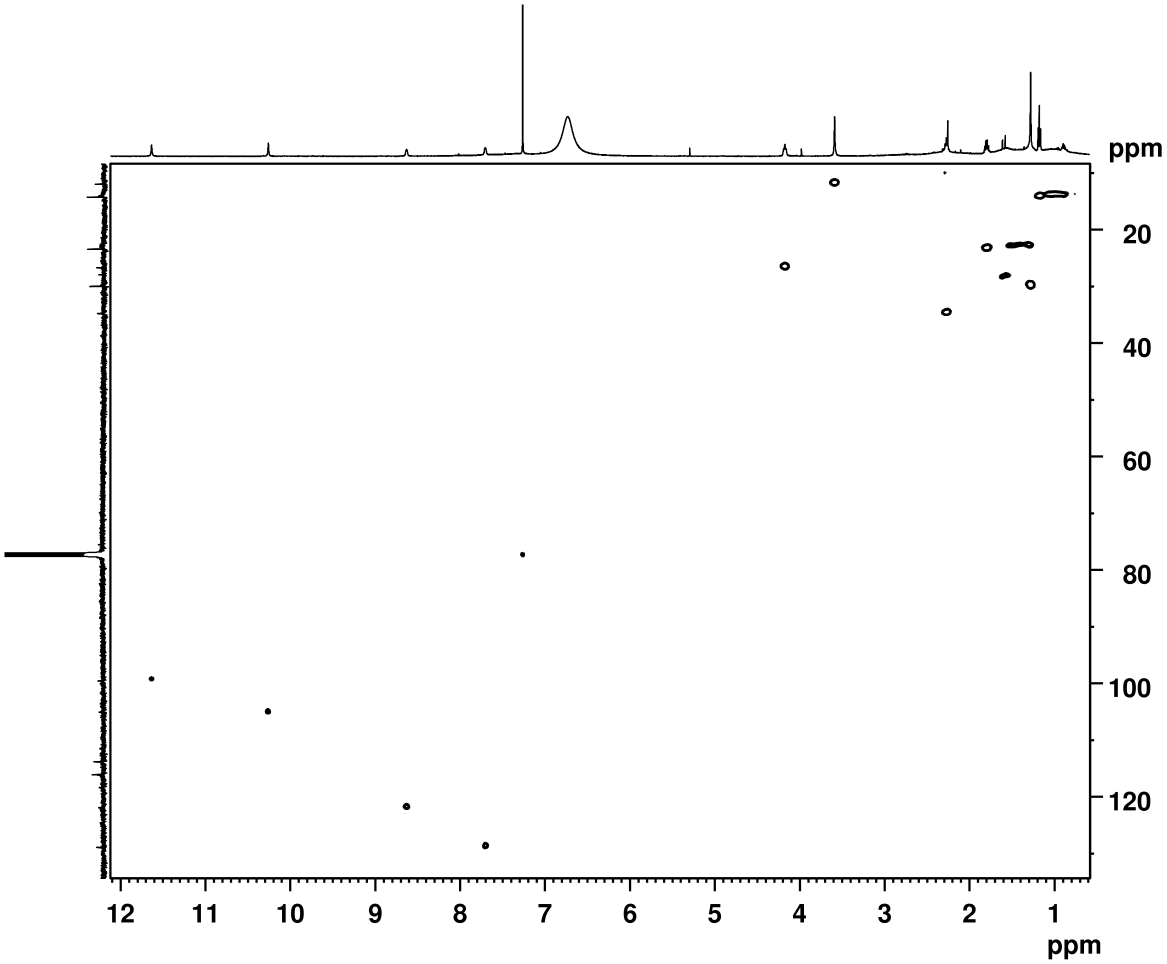

Scheme S52. ^{1}H-^{1}H COSY (above) and HSQC (below) NMR spectra of carbaporphyrin **36** in TFA-CDCl$_3$.

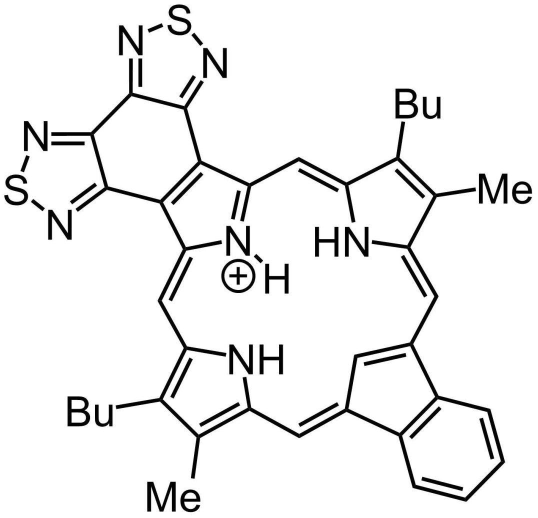

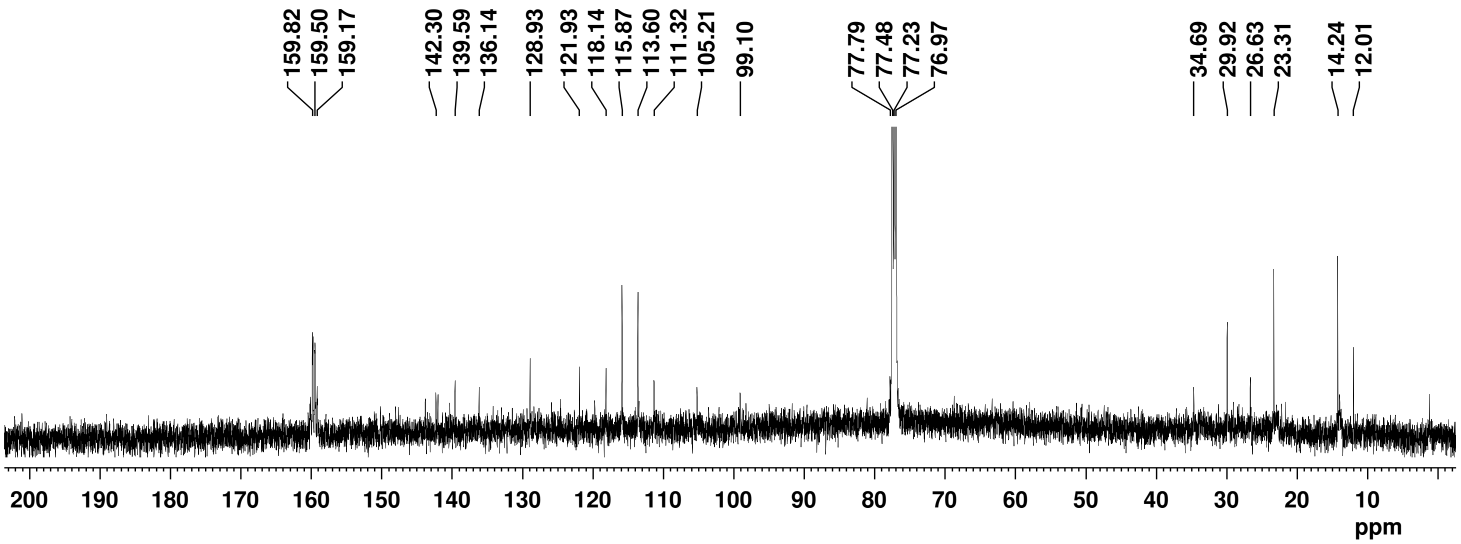

Scheme S53. 125 MHz $^{13}C\{^{1}H\}$ NMR spectrum of carbaporphyrin **36** in TFA-$CDCl_3$.

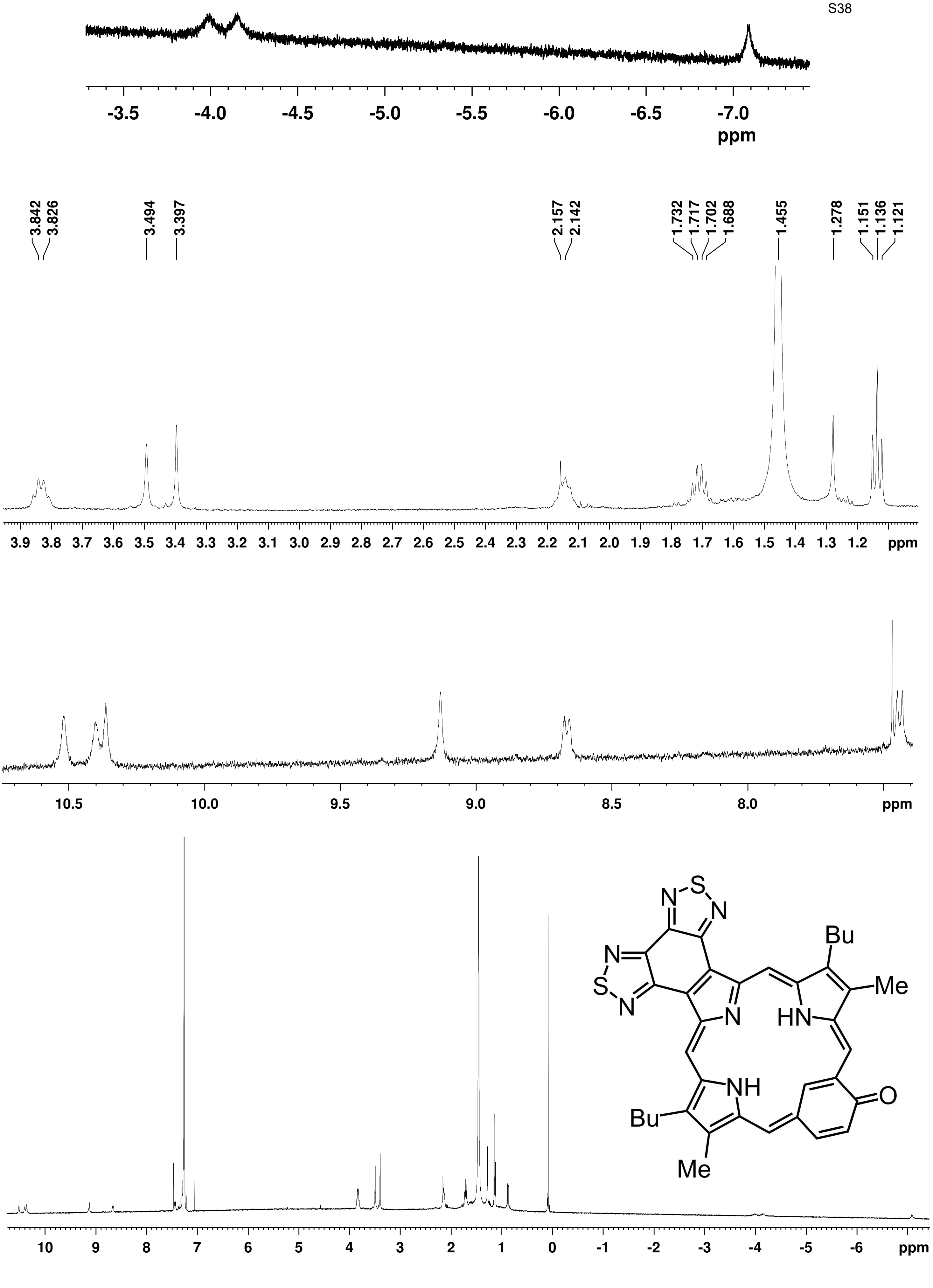

Figure S54. 500 MHz proton NMR spectrum of highly insoluble oxybenziporphyrin **40** in $CDCl_3$ at 55 °C.

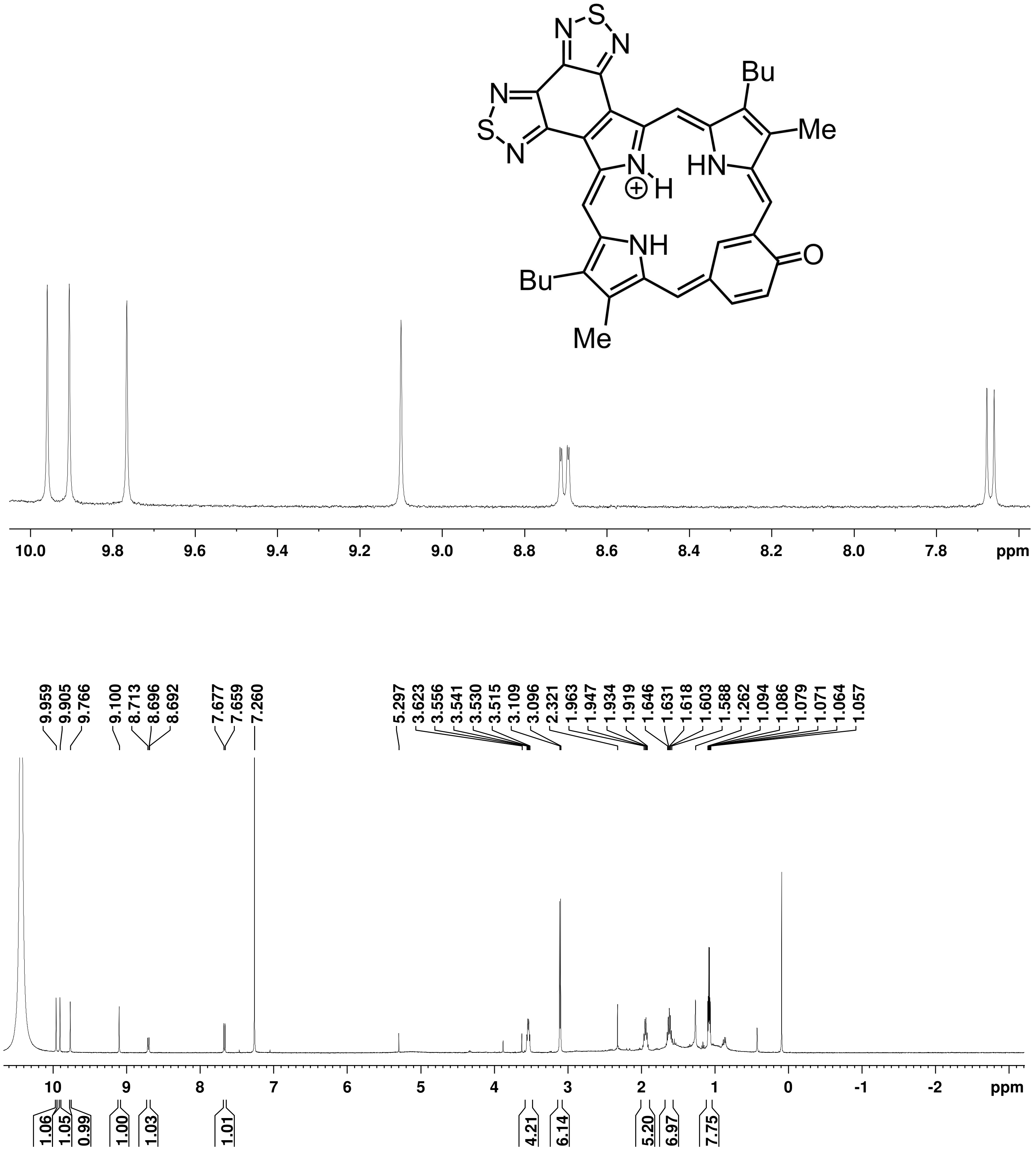

Figure S55. 500 MHz proton NMR spectrum of oxybenziporphyrin **40** in TFA-$CDCl_3$.

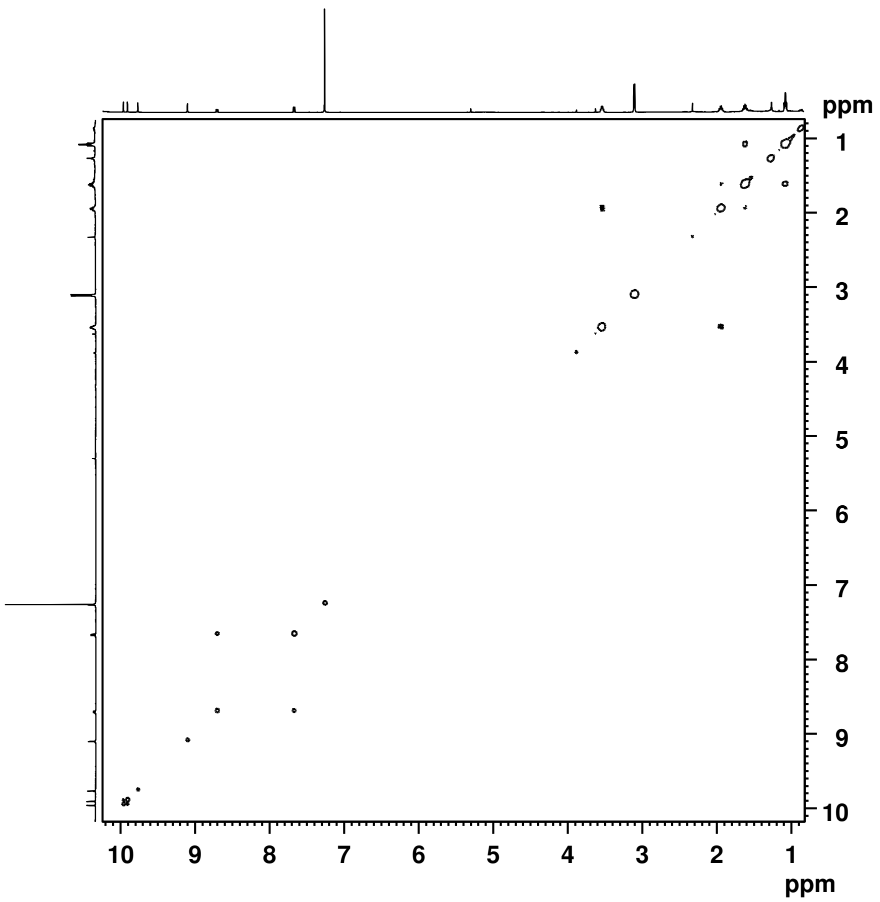

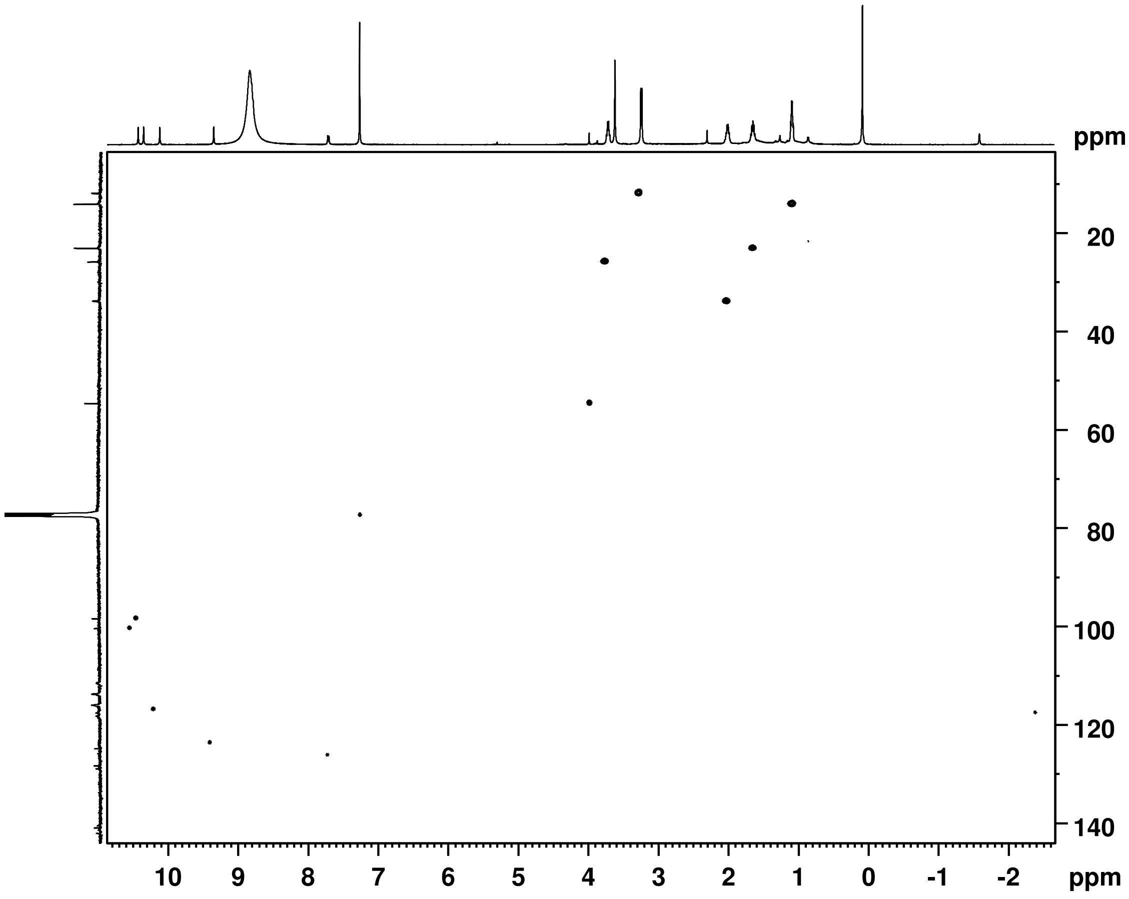

Figure S56. ^{1}H-^{1}H COSY (above) and HSQC (below) NMR spectra of oxybenziporphyrin **40** in TFA-$CDCl_3$.

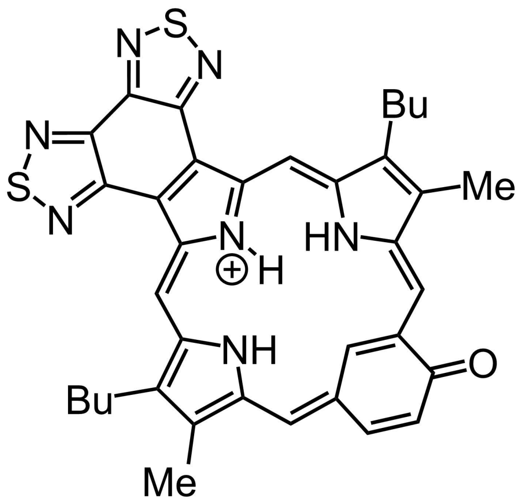

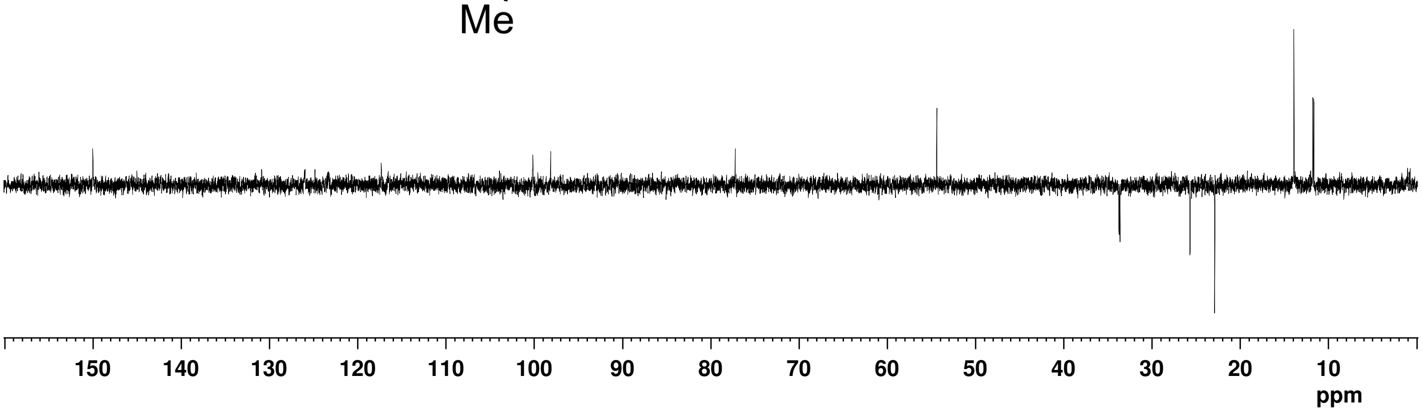

Figure S57. DEPT-135 NMR spectrum of oxybenziporphyrin **40** in TFA-$CDCl_3$.

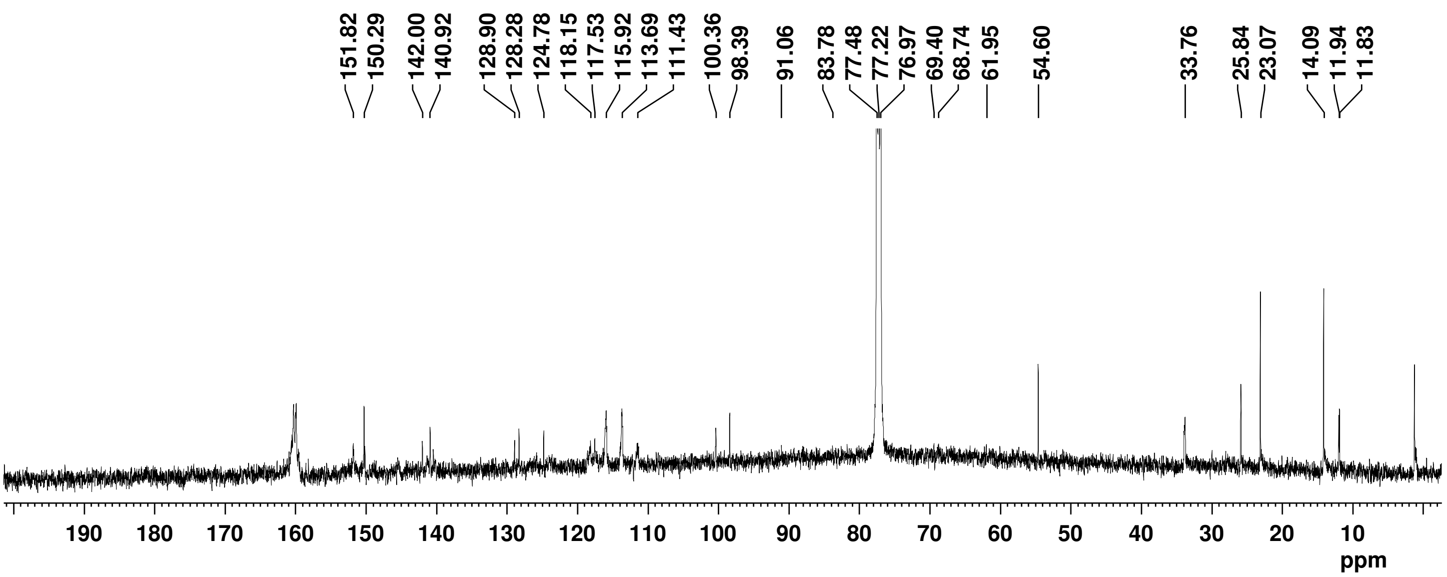

Figure S58. 125 MHz $^{13}C\{^{1}H\}$ NMR spectrum of oxybenziporphyrin **40** in TFA-$CDCl_3$.

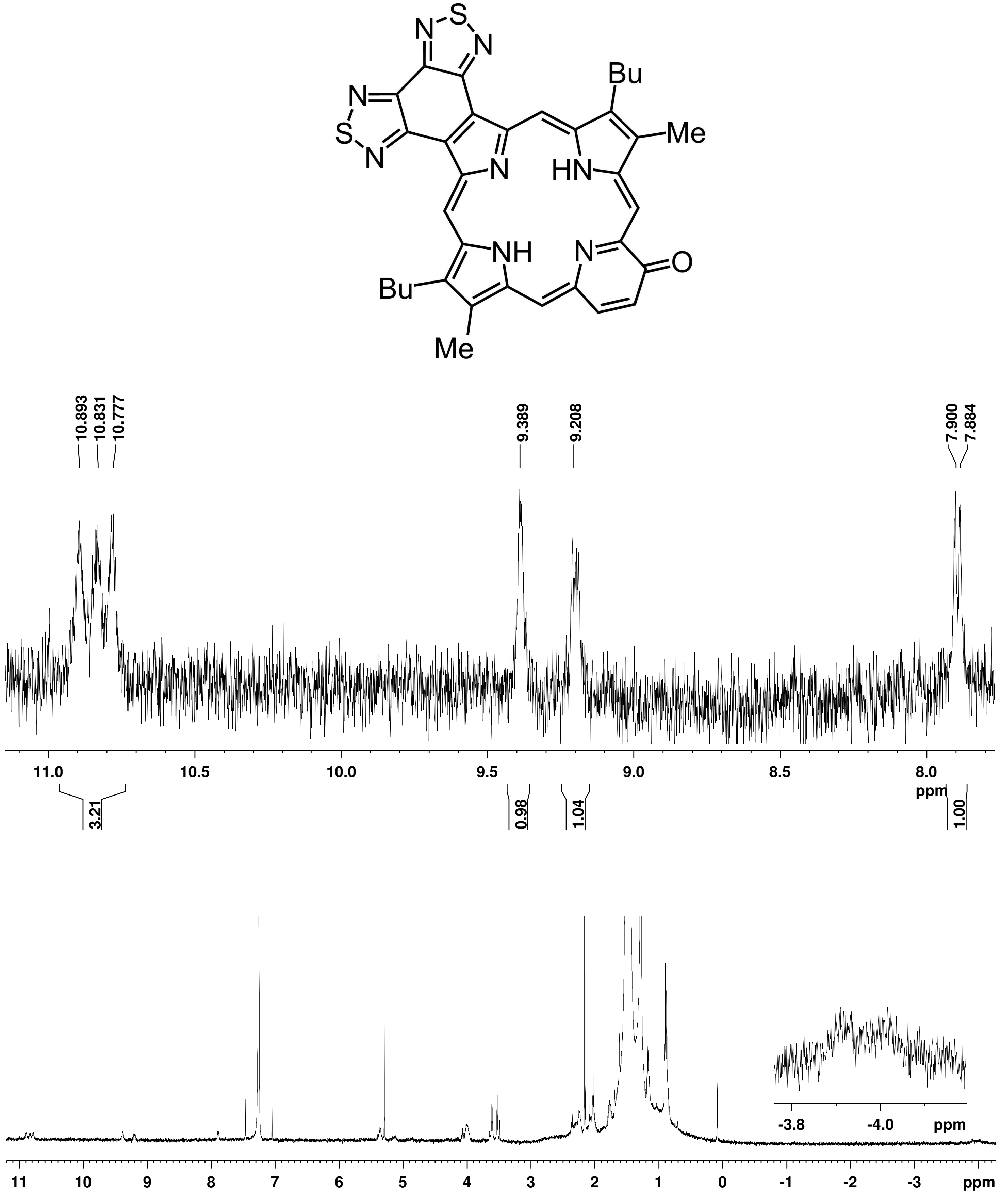

Figure S49. 500 MHz proton NMR spectrum of highly insoluble oxypyriporphyrin **38** in $CDCl_3$ at 55 ^{o}C.

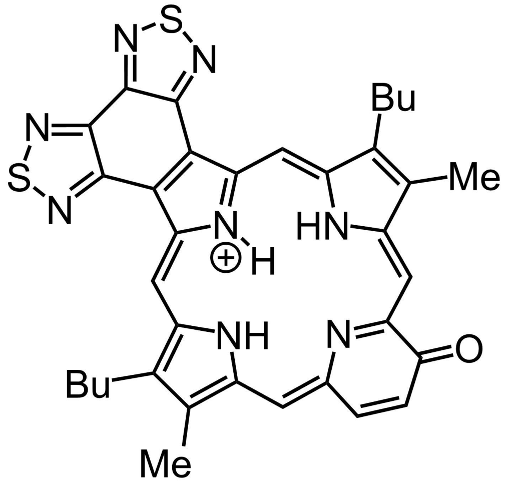

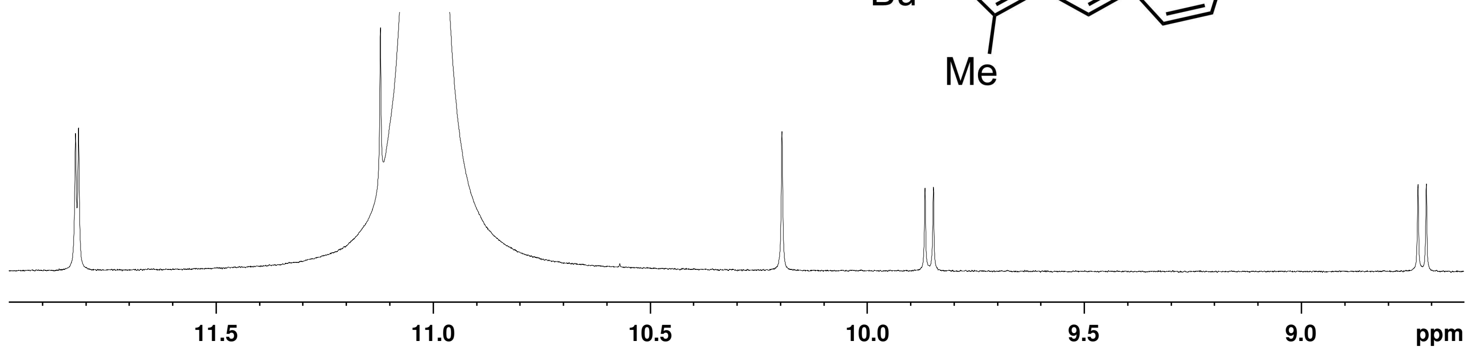

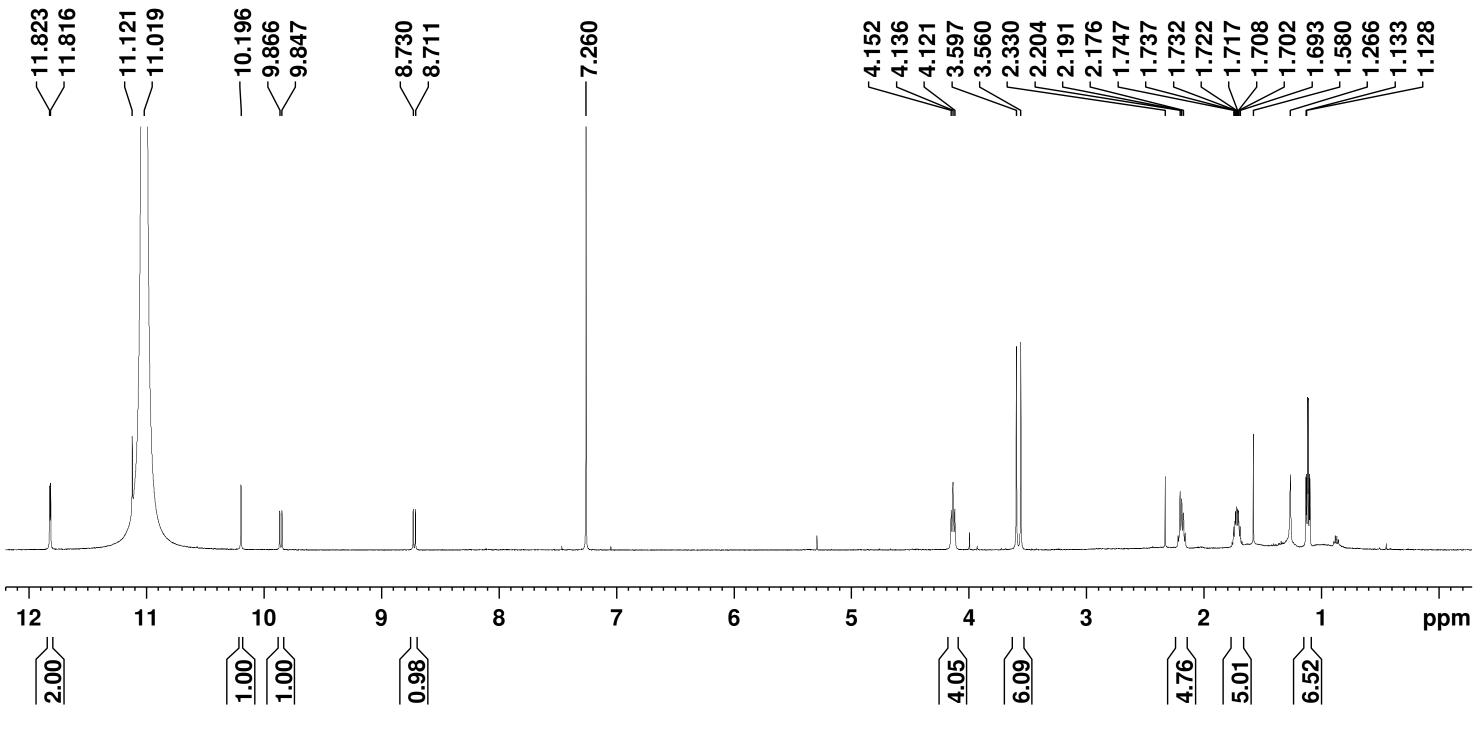

Figure S50. 500 MHz proton NMR spectrum of oxypyriporphyrin **38** in TFA-$CDCl_3$.

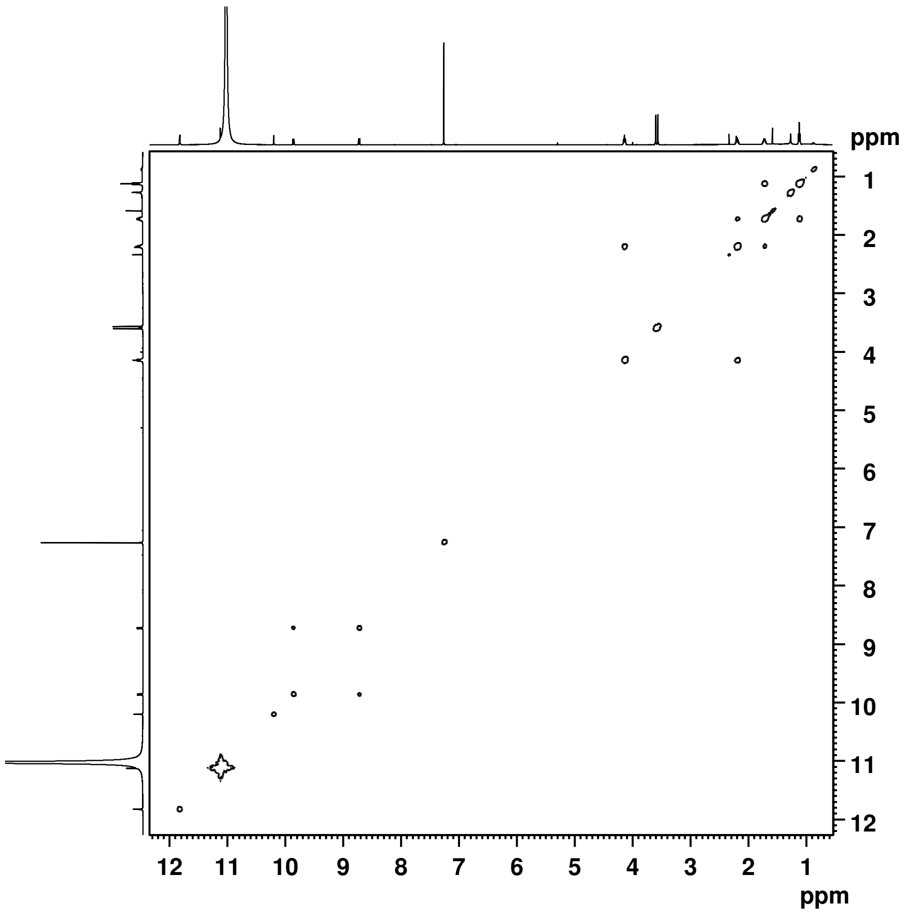

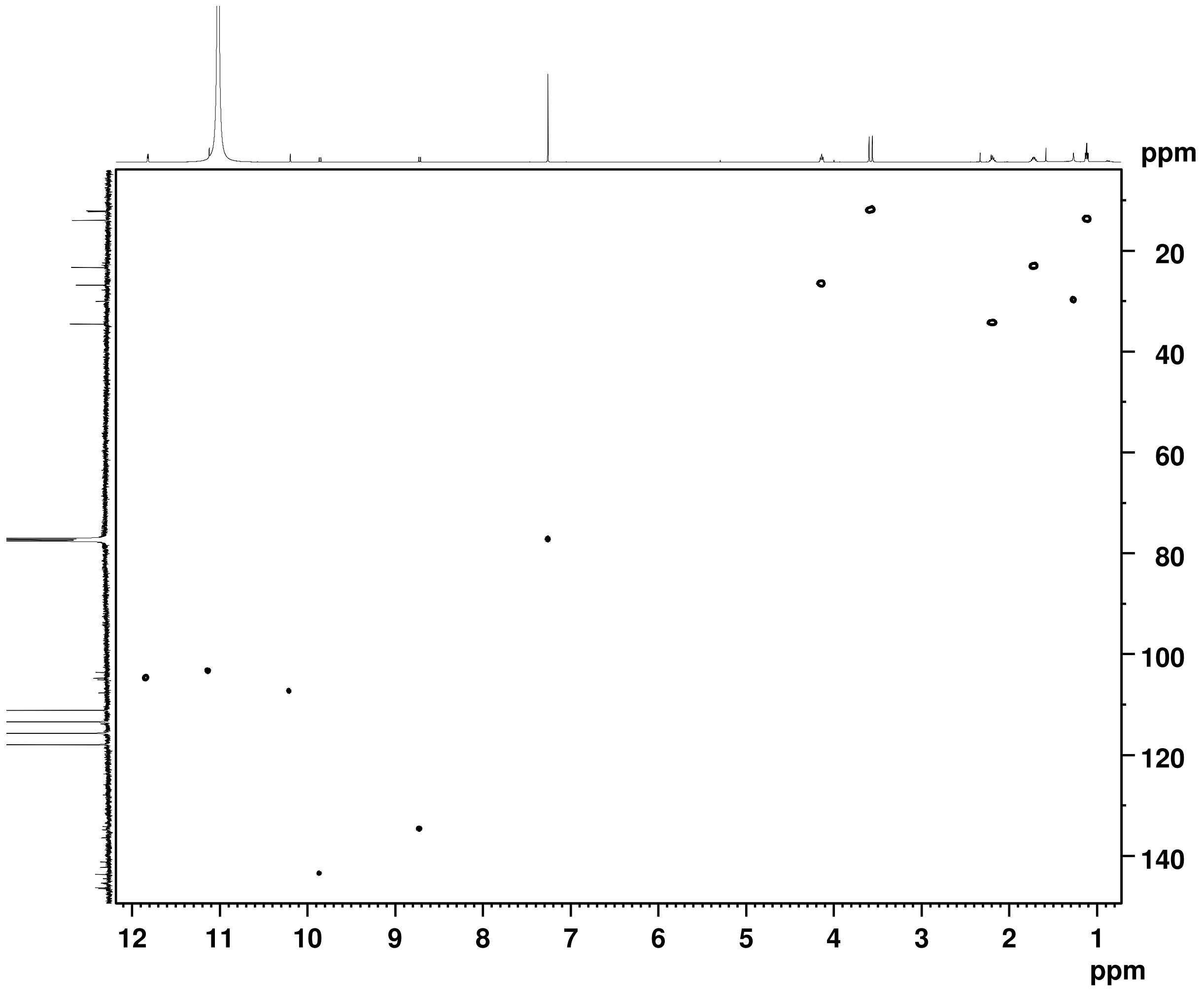

Figure S51. ^{1}H-^{1}H COSY (above) and HSQC (below) NMR spectra of oxypyriporphyrin **38** in TFA-$CDCl_3$.

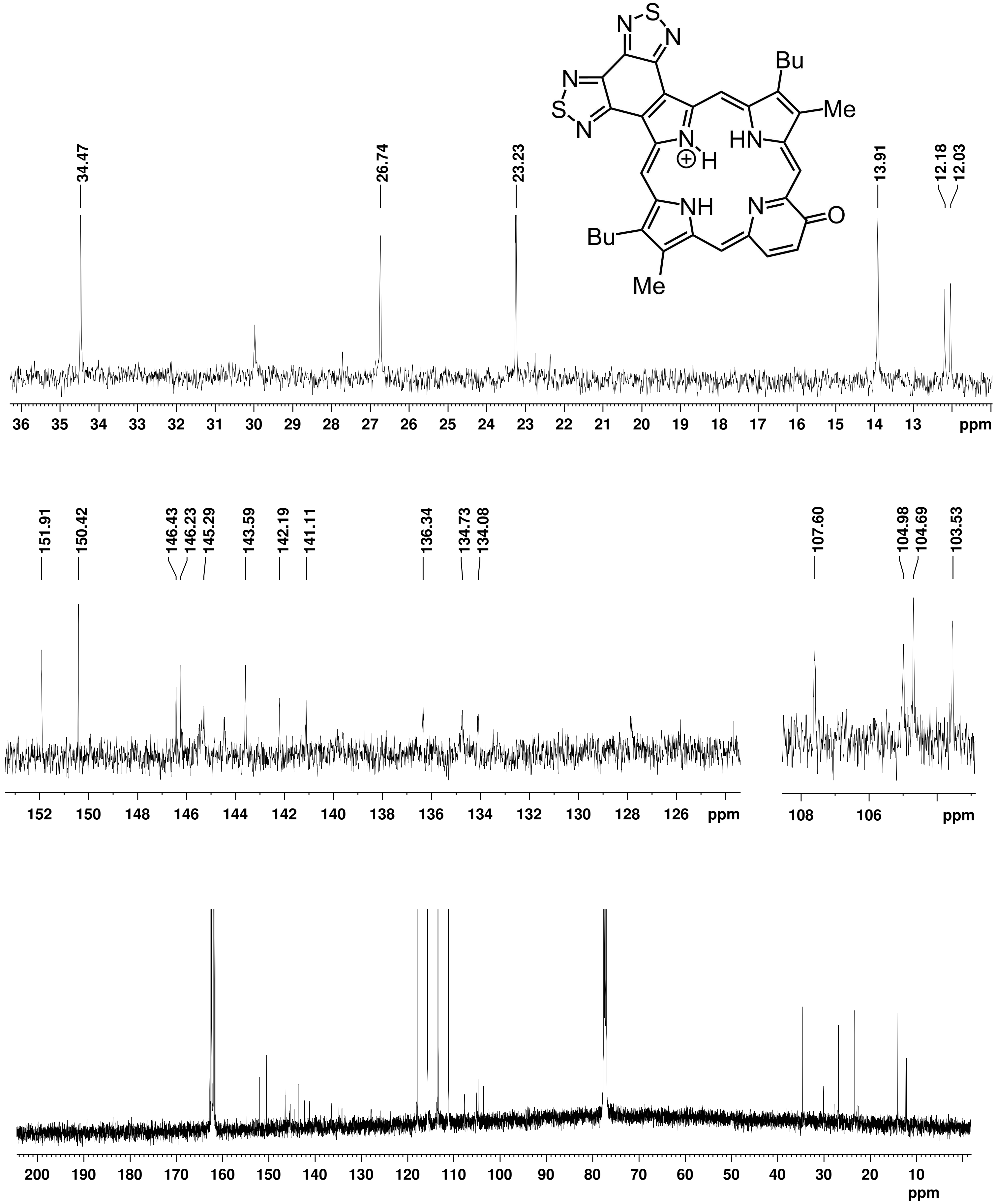

Figure S52. 125 MHz $^{13}C\{^{1}H\}$ NMR spectrum of oxypyriporphyrin **38** in TFA-$CDCl_3$.

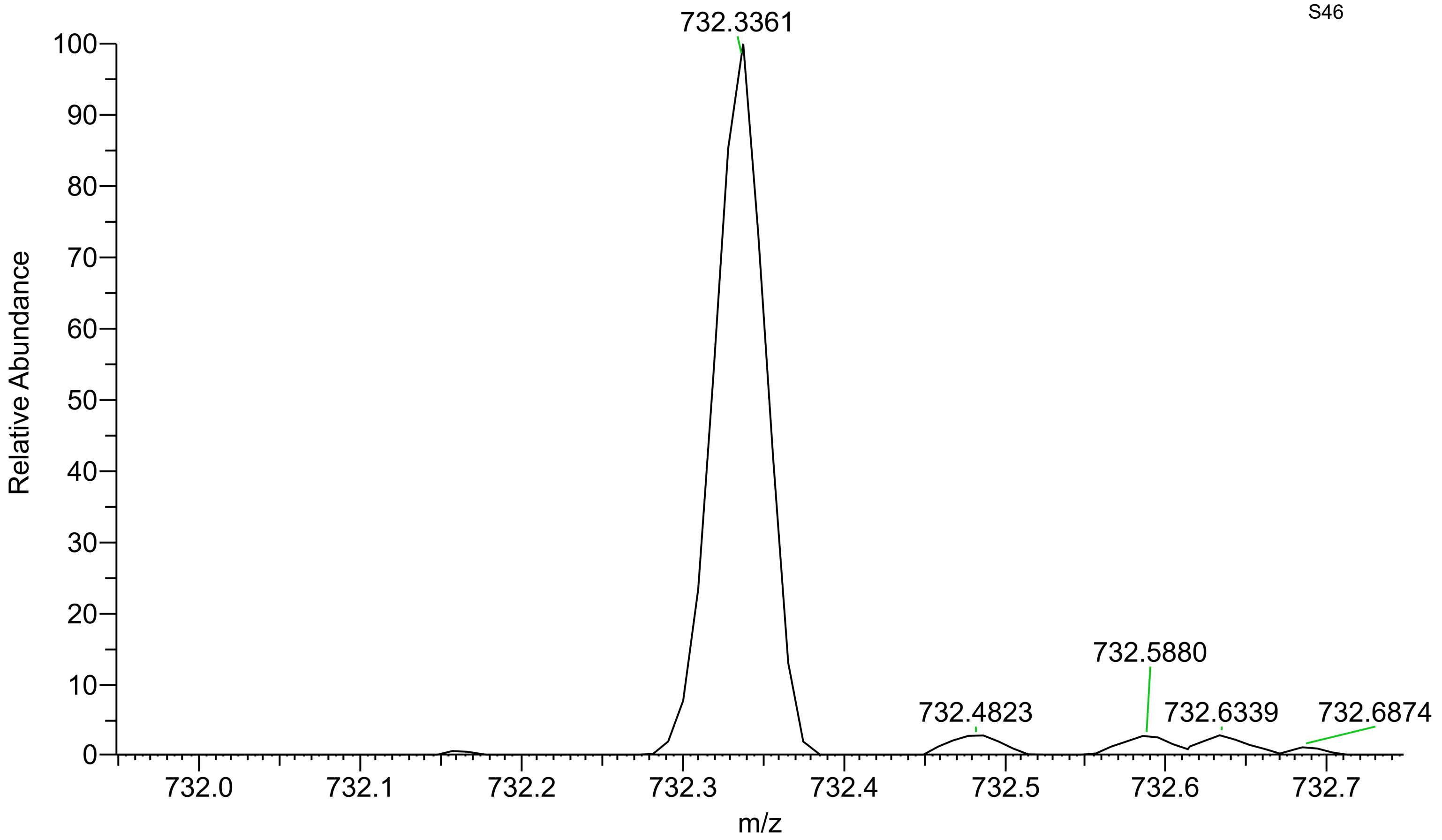

Figure S53. High resolution TOF ESI mass spectrum of tripyrrane **32**.

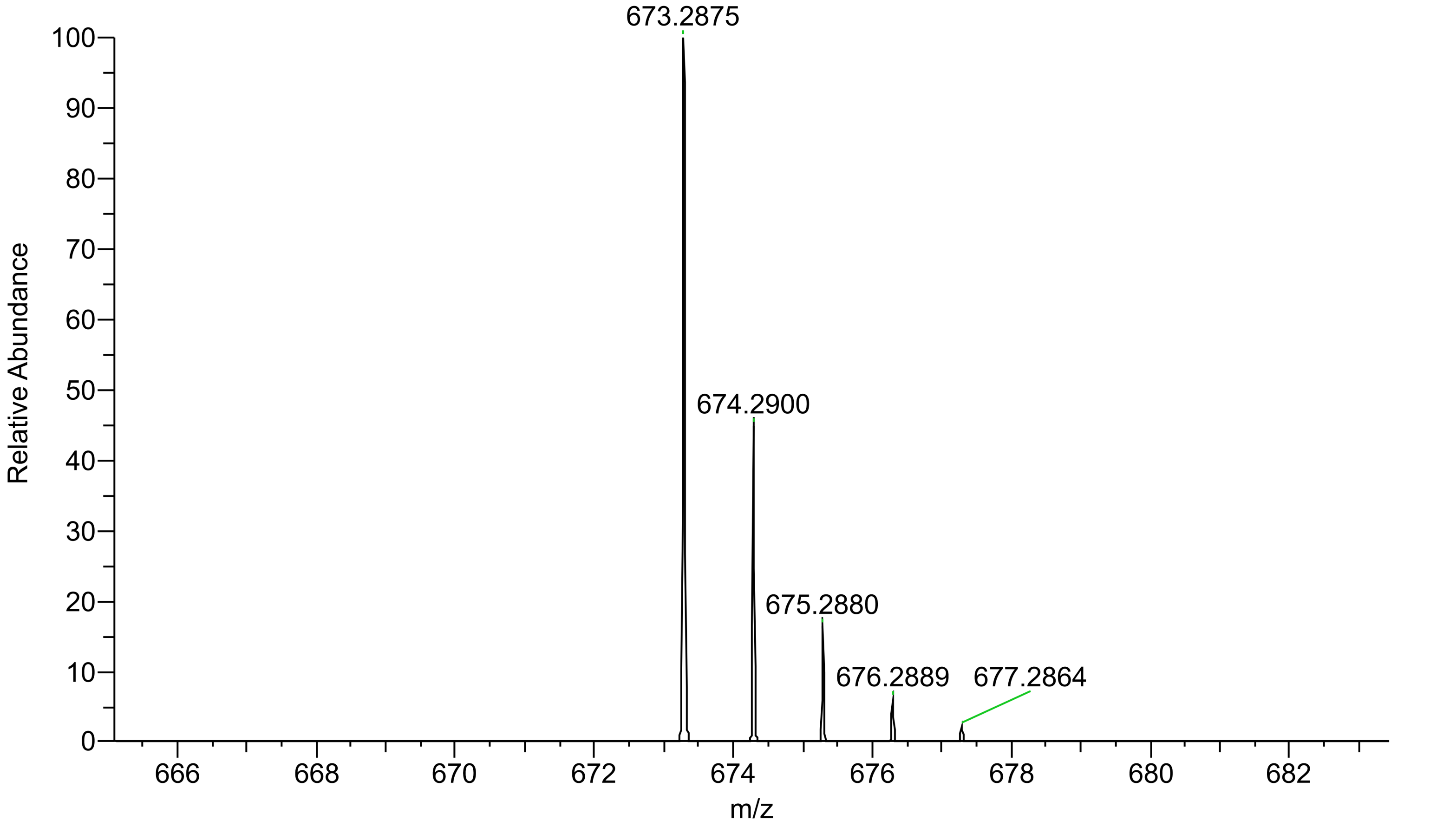

Figure S54. High resolution TOF ESI mass spectrum of porphyrin **33**.

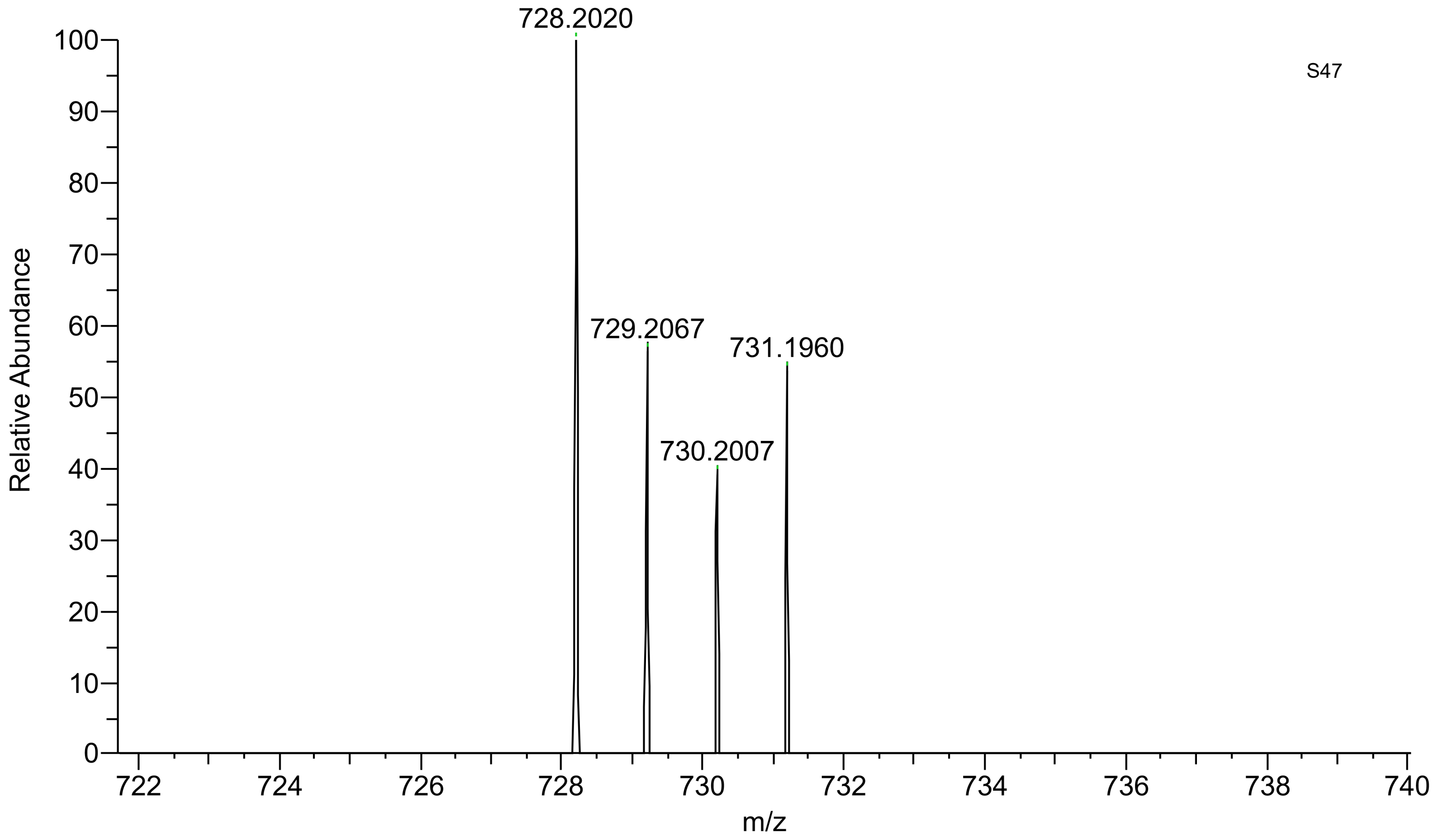

Figure S55. High resolution TOF ESI mass spectrum of nickel complex **33Ni**.

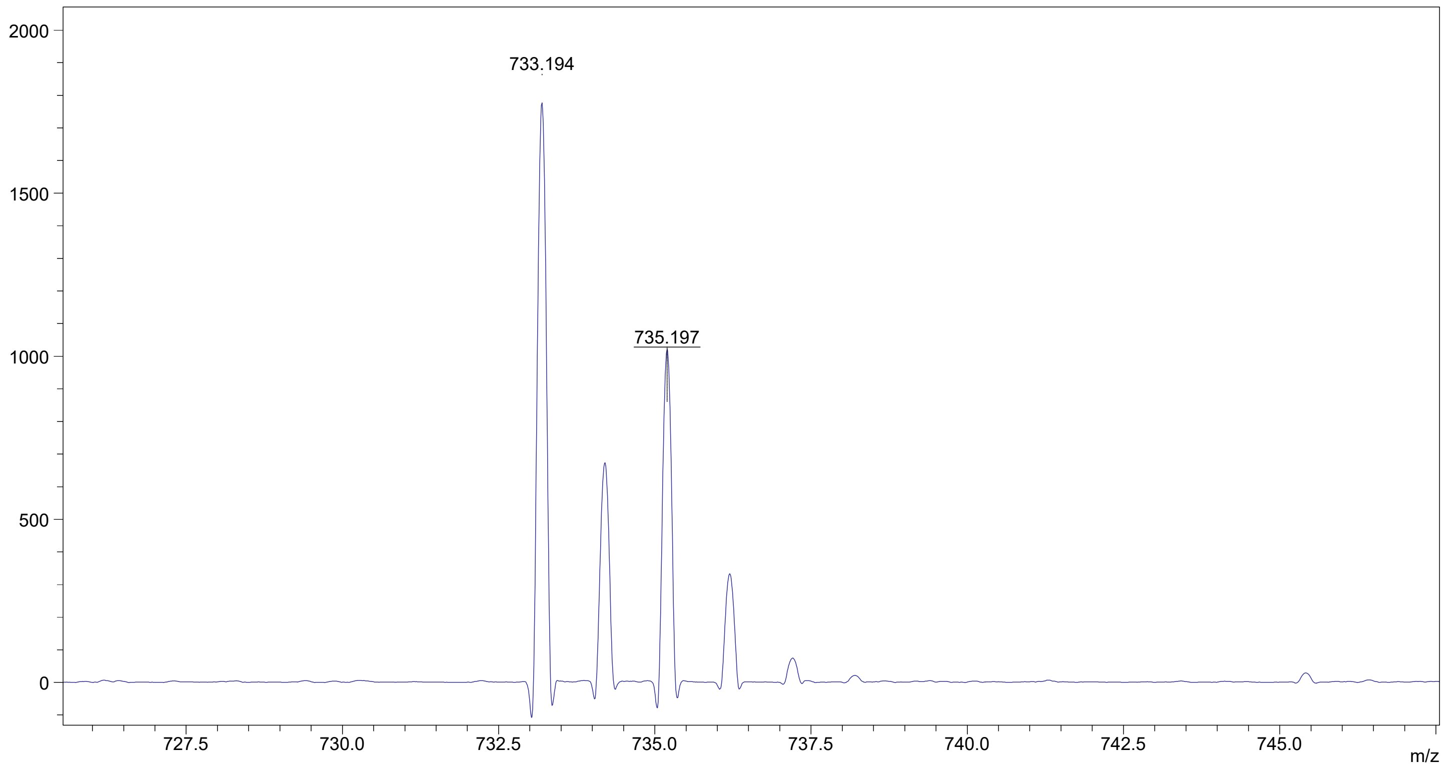

Figure S56. High resolution MALDI mass spectrum of copper complex **33Cu**.

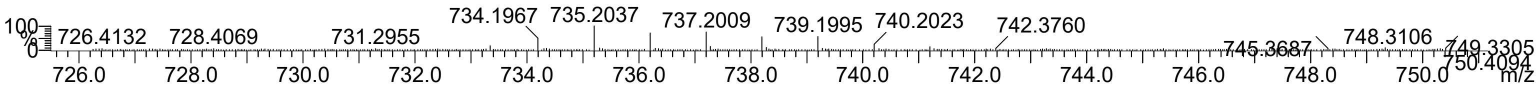

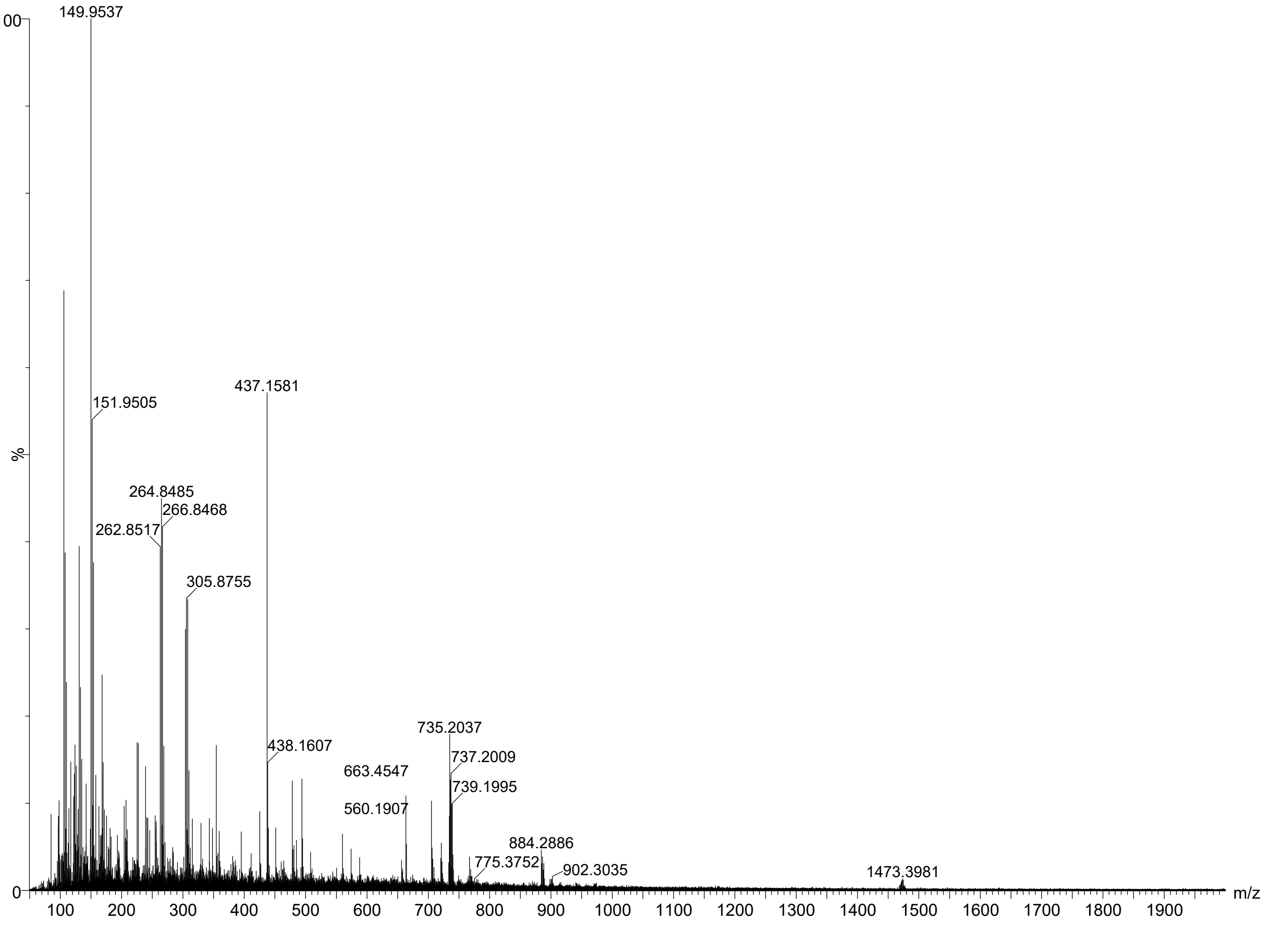

Figure S57. High resolution TOF ESI mass spectrum of zinc complex **33Zn**.

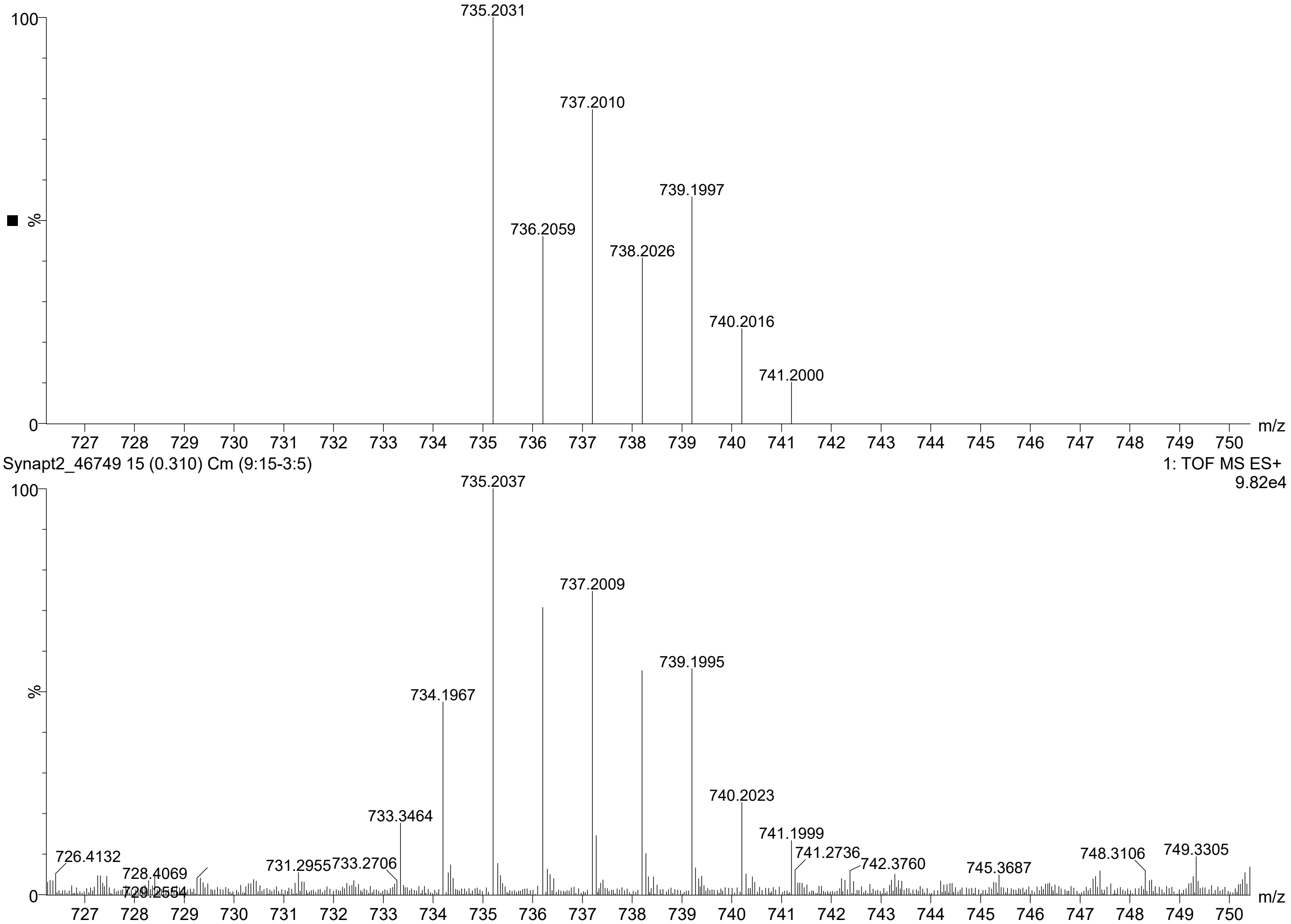

Figure S58. Comparison of the theoretical isotope distribution (top) for **33Zn** with the observed ESI MS (bottom).

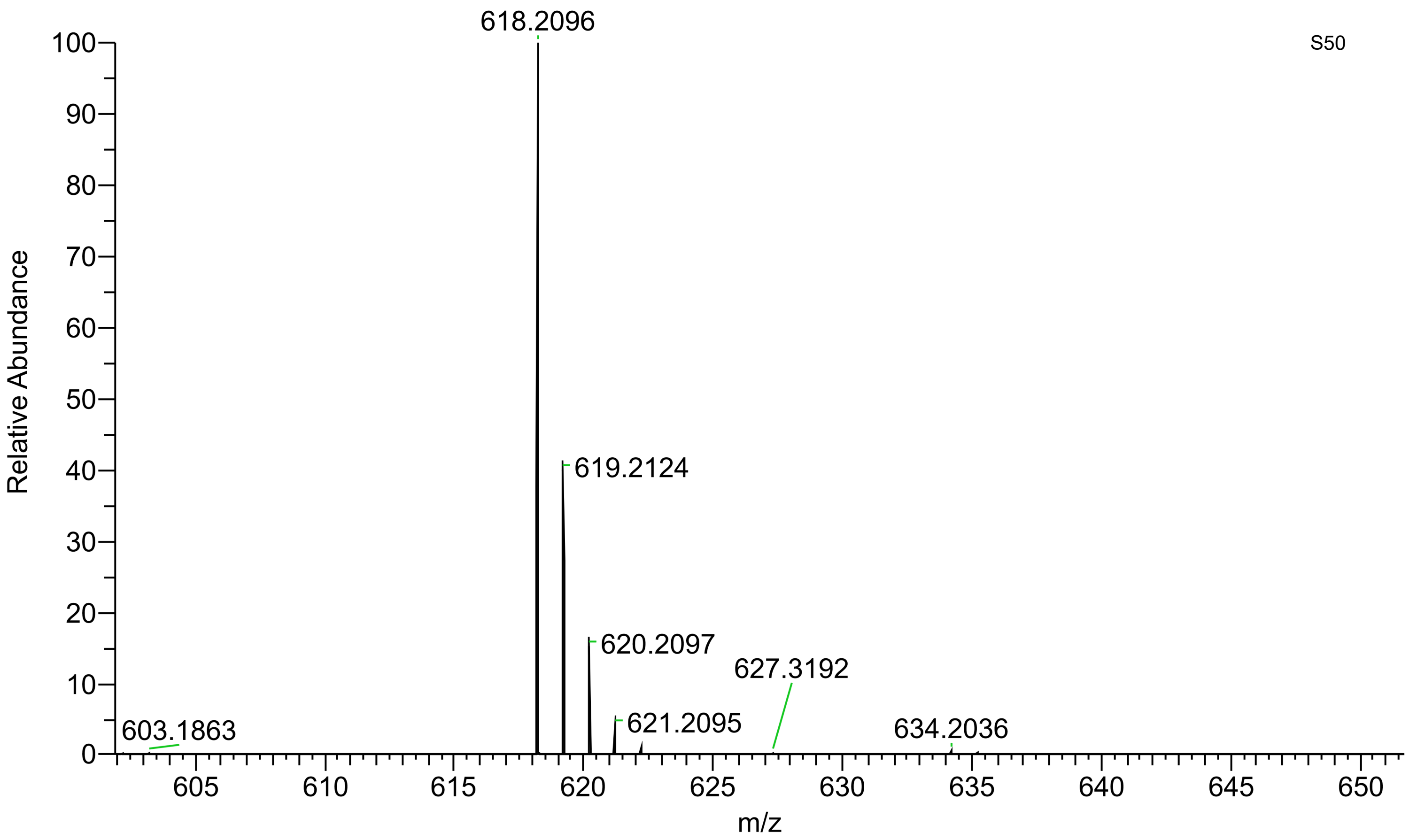

Figure S59. High resolution TOF ESI mass spectrum of oxaporphyrin **35a**.

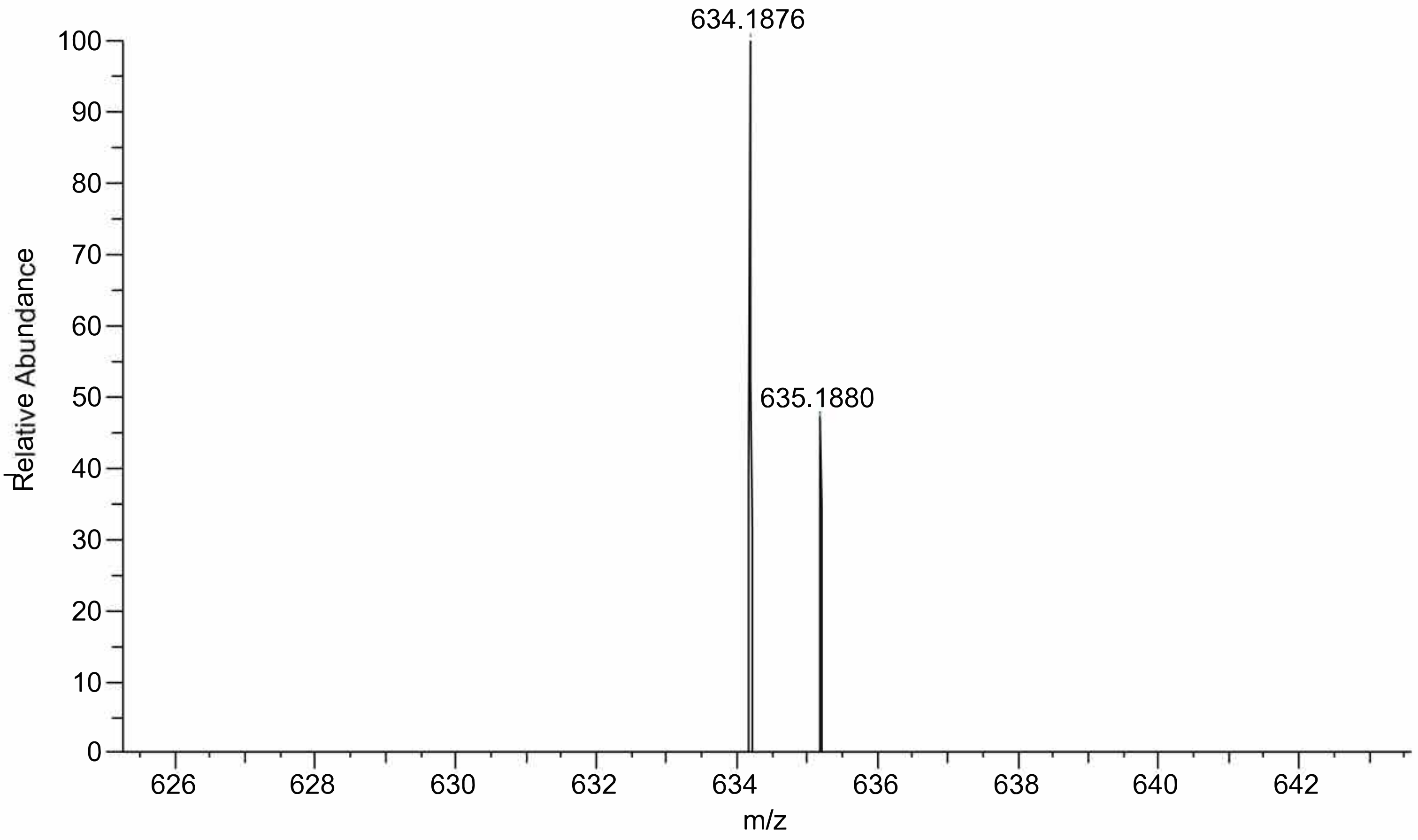

Figure S60. High resolution TOF ESI mass spectrum of thiaporphyrin **35b**.

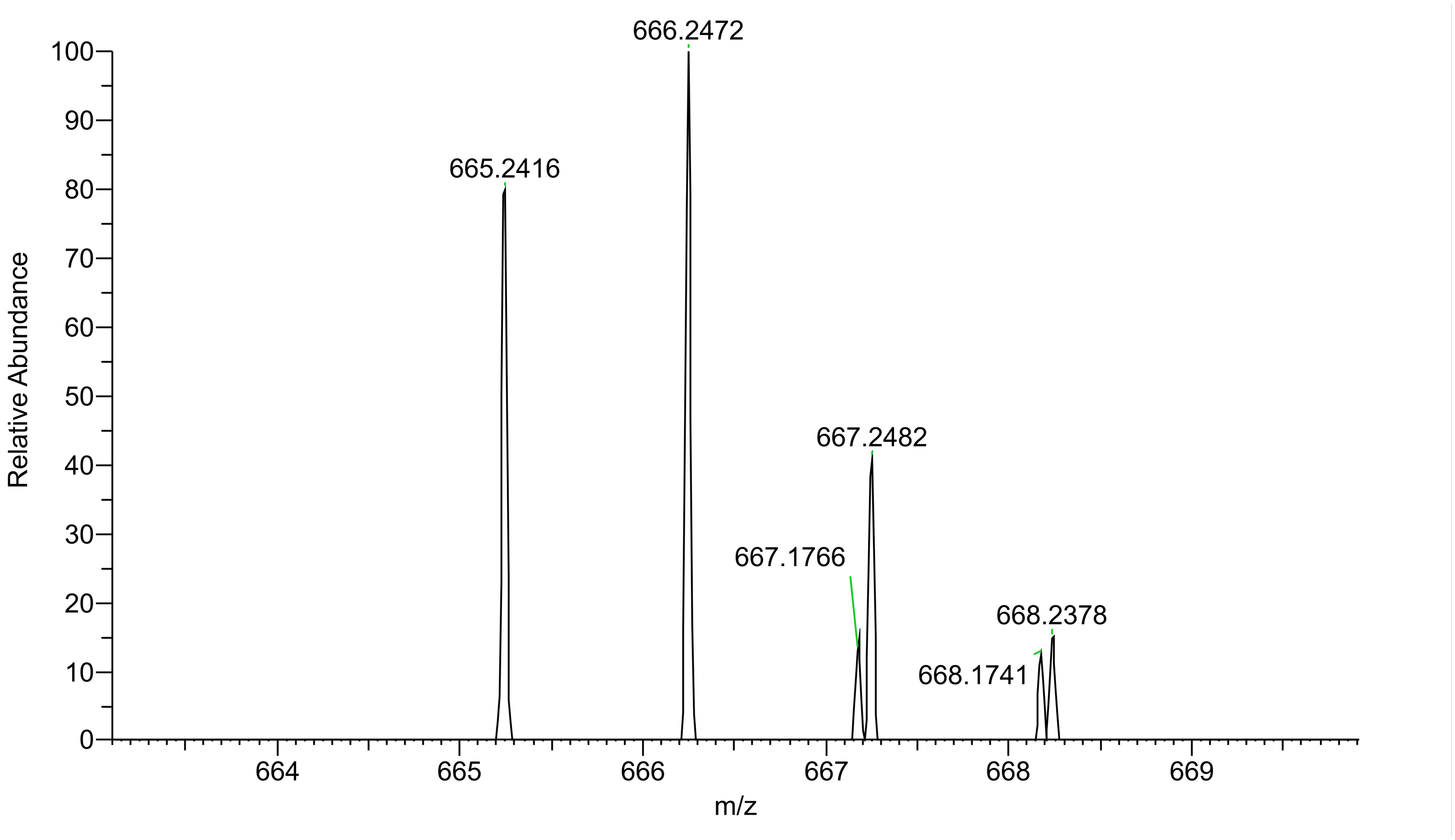

Figure S61. High resolution TOF ESI mass spectrum of carbaporphyrin **36**.

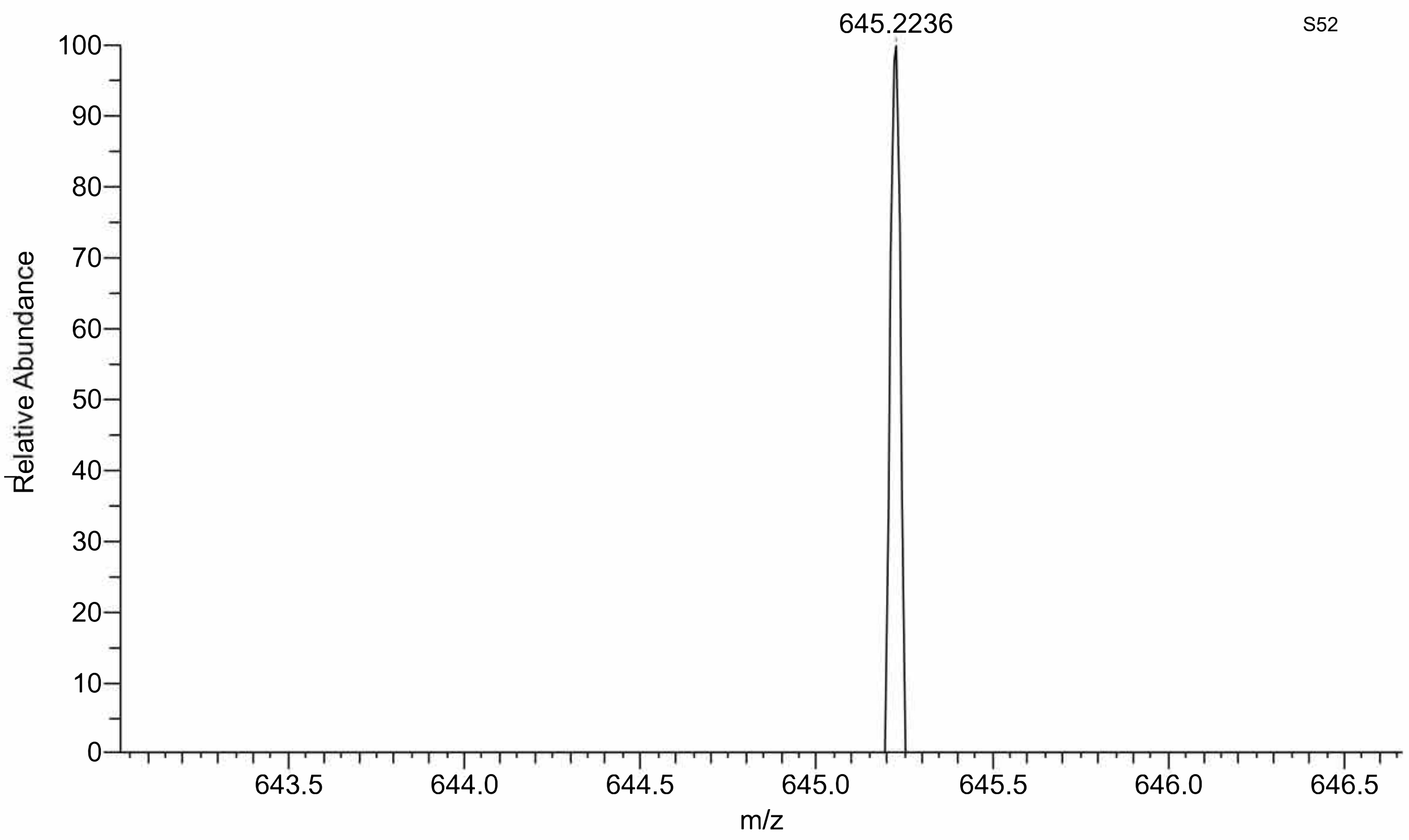

Figure S62. High resolution TOF ESI mass spectrum of oxypyriporphyrin **38**.

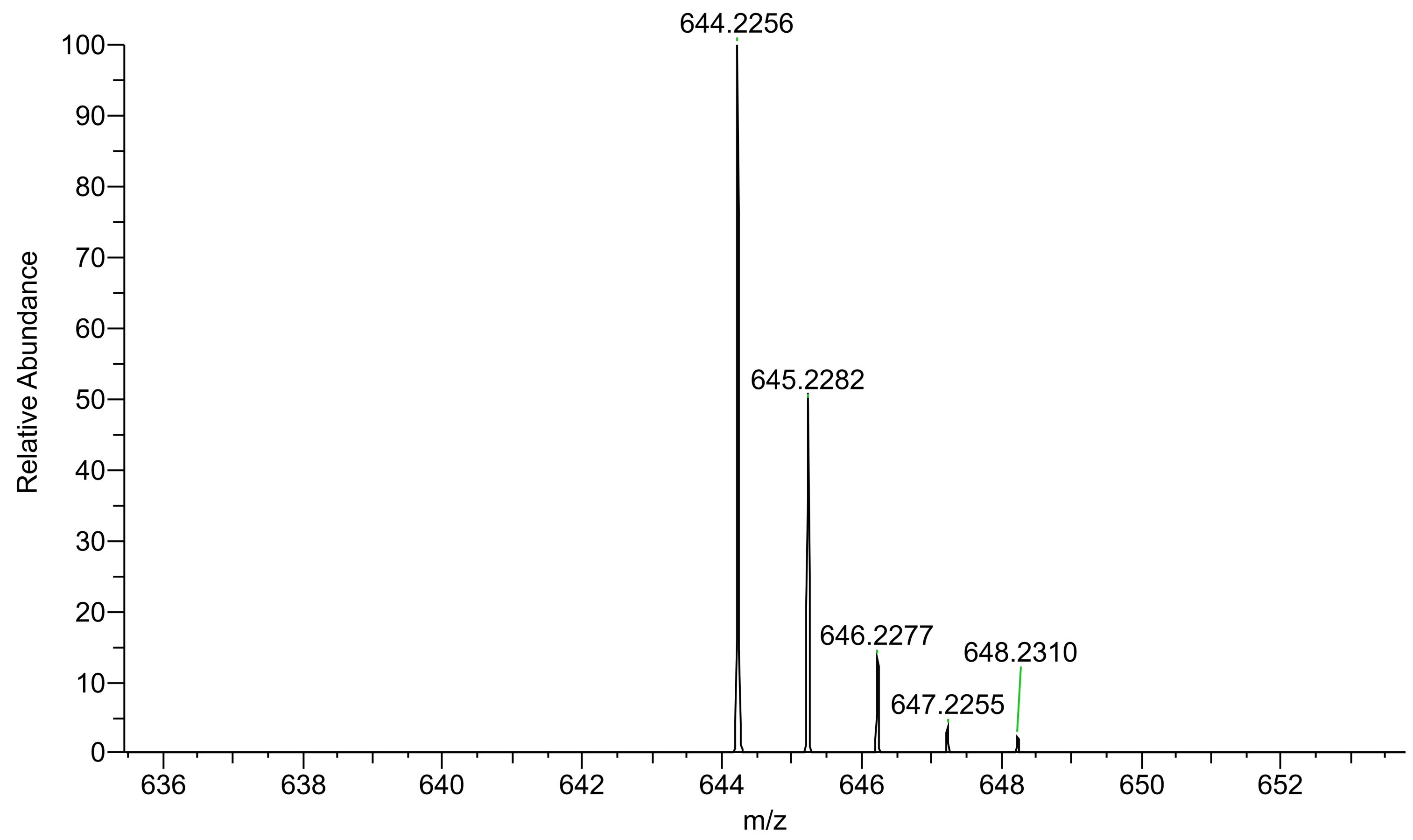

Figure S63. High resolution TOF ESI mass spectrum of oxybenziporphyrin **40**.

S_2-BPa

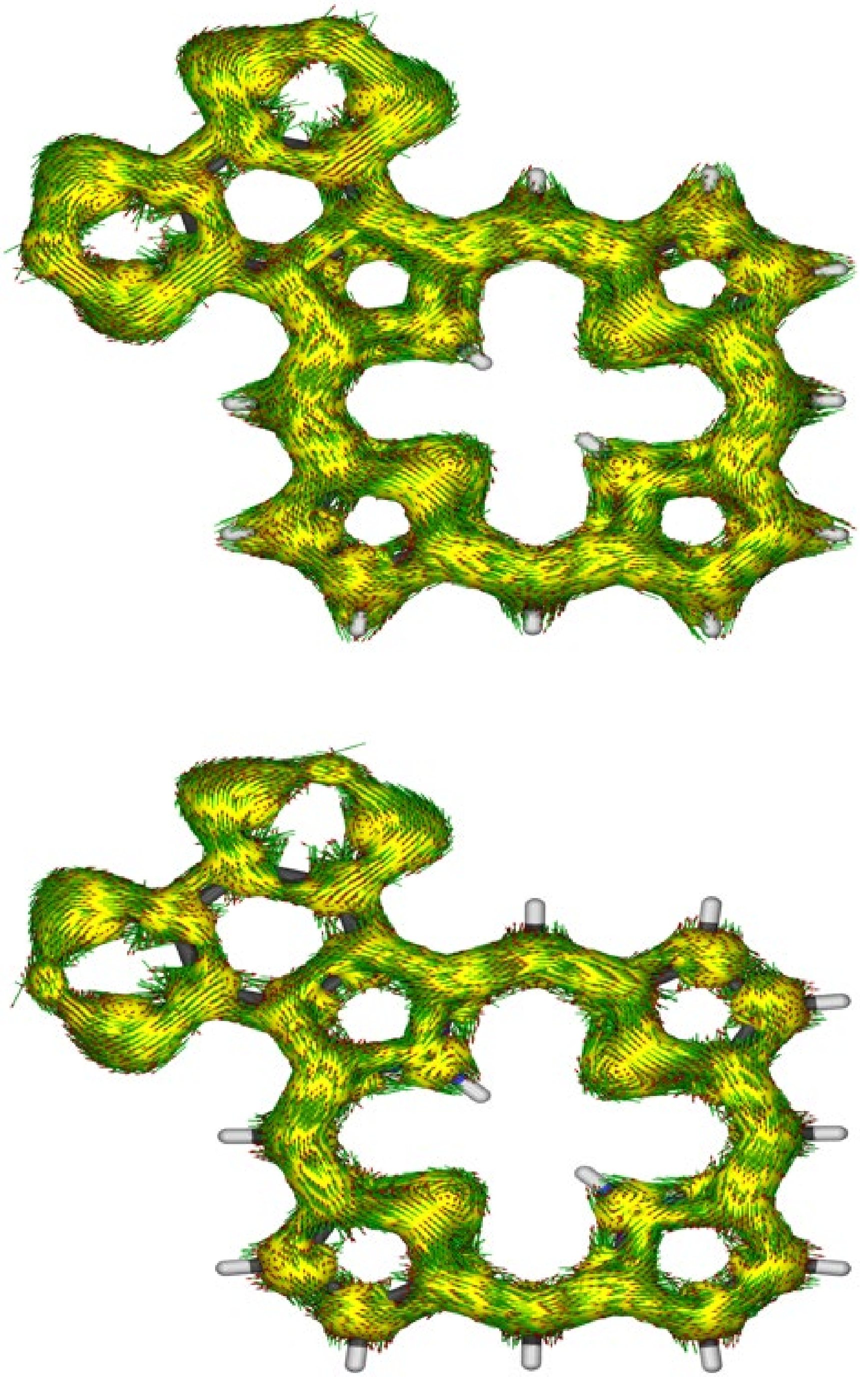

Figure S64. AICD plots for bis(thiadiazolobenzo-porphyrin tautomer **S_2-BPa** (isovalues 0.05 and 0.07, respectively).

S_2-BPb

0.05

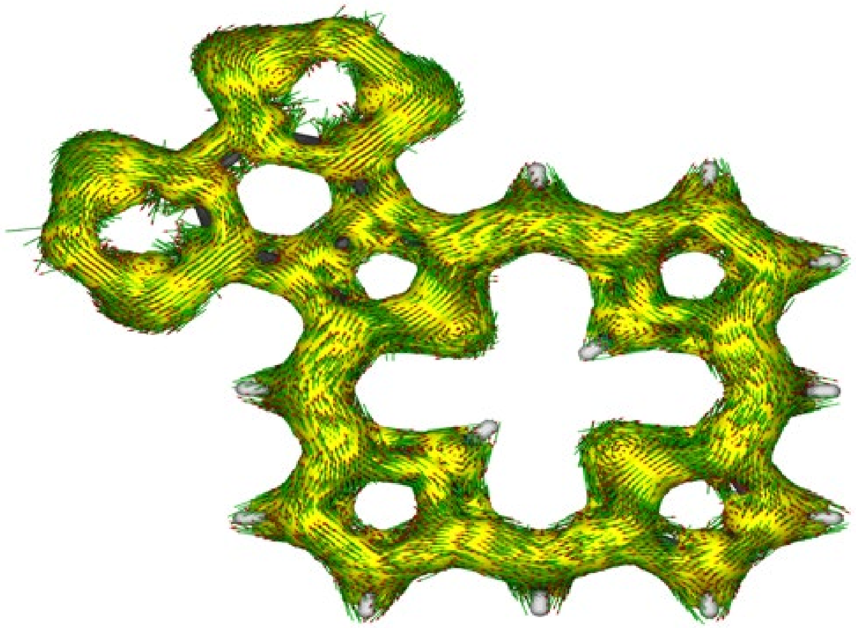

0.07

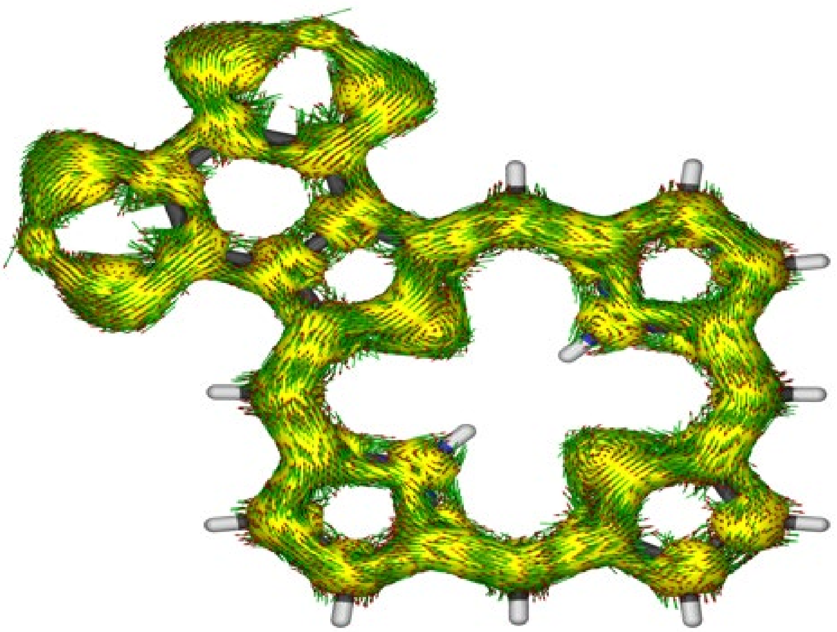

Figure S65. AICD plots for bis(thiadiazolobenzo-porphyrin tautomer **S_2-BPb** (isovalues 0.05 and 0.07, respectively).

S_2-BPc

0.05

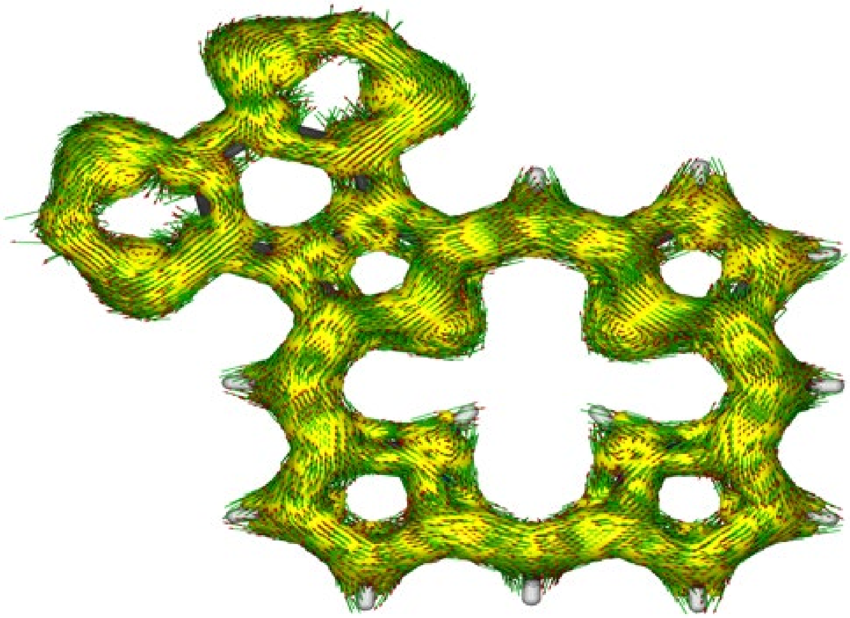

0.07

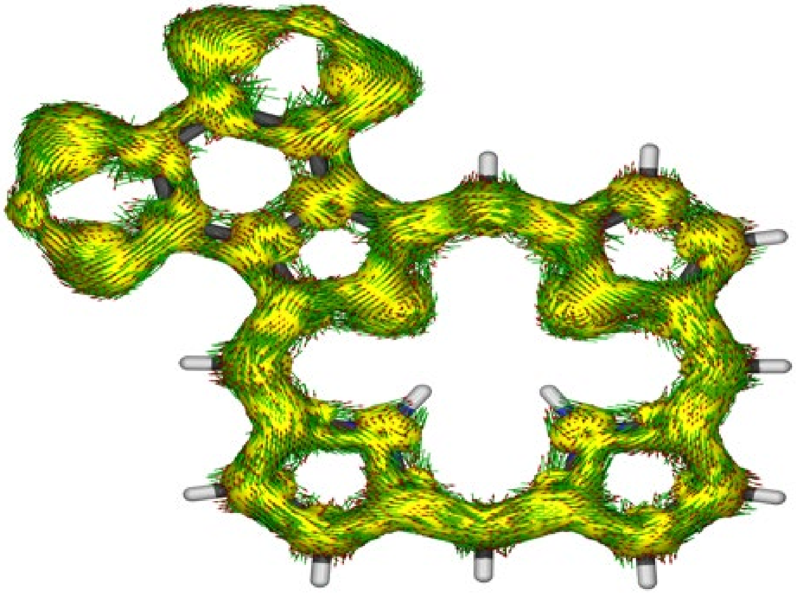

Figure S66. AICD plots for bis(thiadiazolobenzo-porphyrin tautomer **S_2-BPc** (isovalues 0.05 and 0.07, respectively).

S_2-BPd

0.05

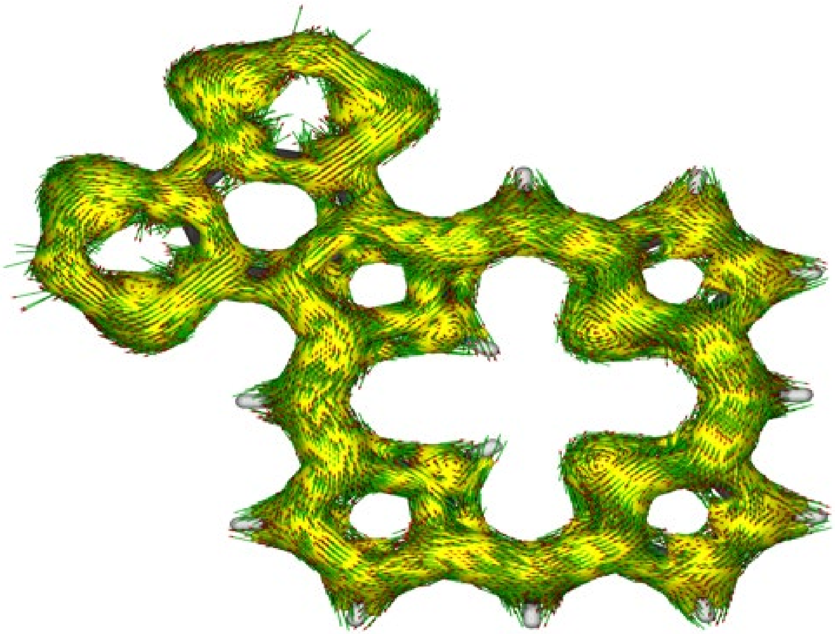

0.07

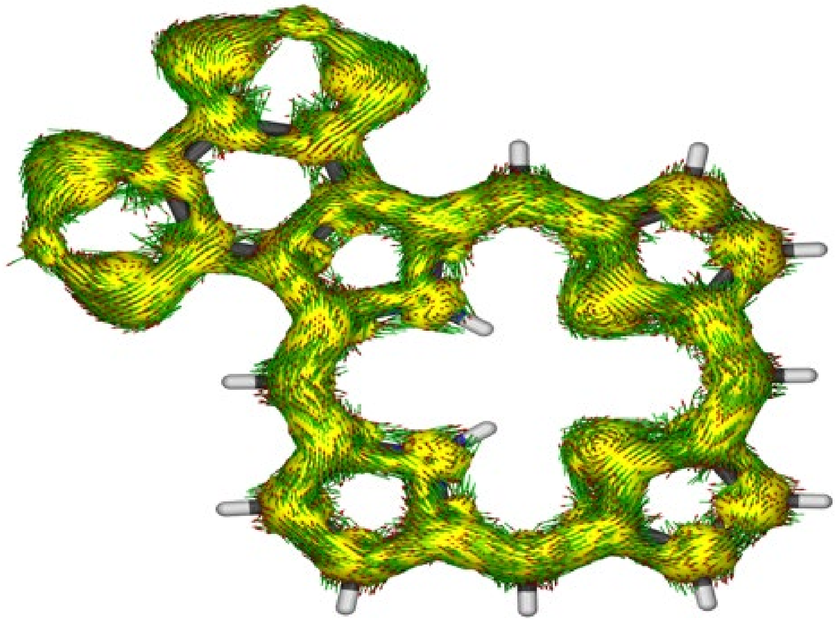

Figure S67. AICD plots for bis(thiadiazolobenzo-porphyrin tautomer **S_2-BPd** (isovalues 0.05 and 0.07, respectively).

S_2-OxBPa

0.05

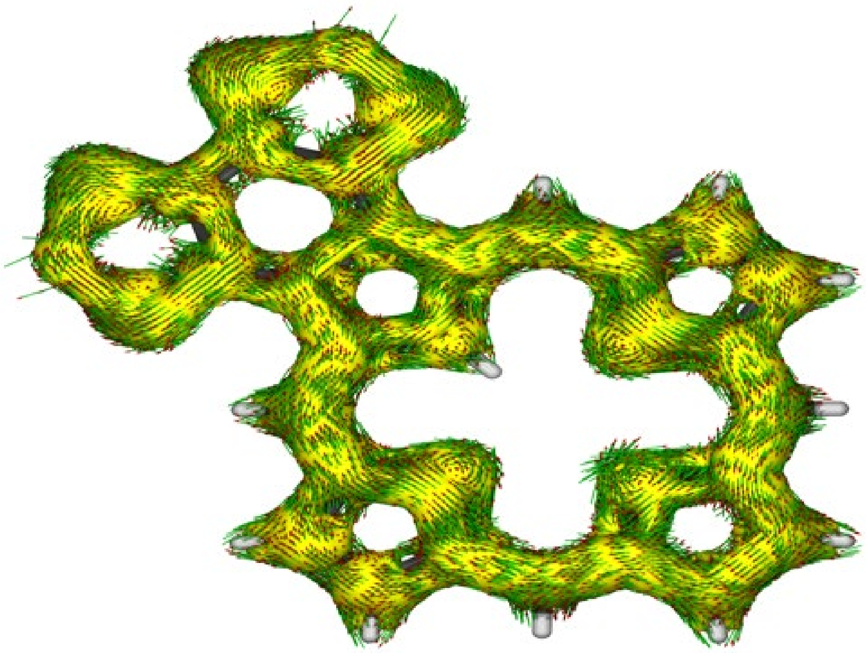

0.07

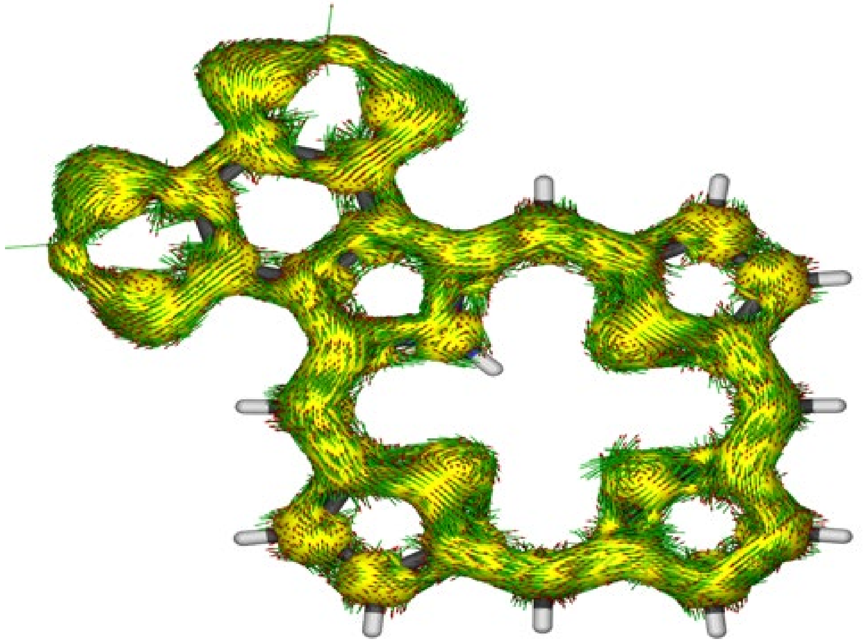

Figure S68. AICD plots for bis(thiadiazolobenzo-oxaporphyrin tautomer **S_2-OxPBa** (isovalues 0.05 and 0.07, respectively).

S_2-OxBPb

0.05

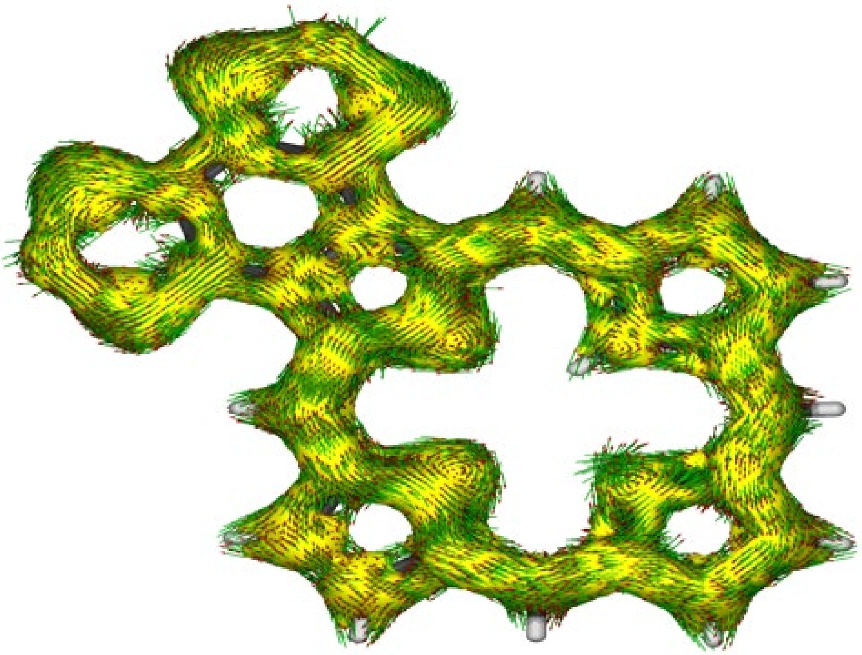

0.07

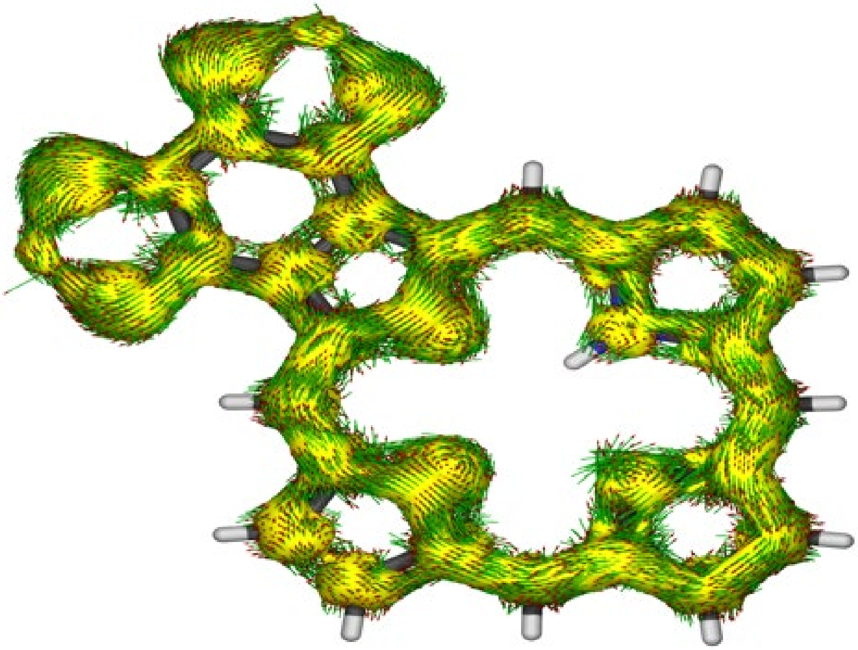

Figure S69. AICD plots for bis(thiadiazolobenzo-oxaporphyrin tautomer **S_2-OxPBb** (isovalues 0.05 and 0.07, respectively).

S_2-BTPa

0.05

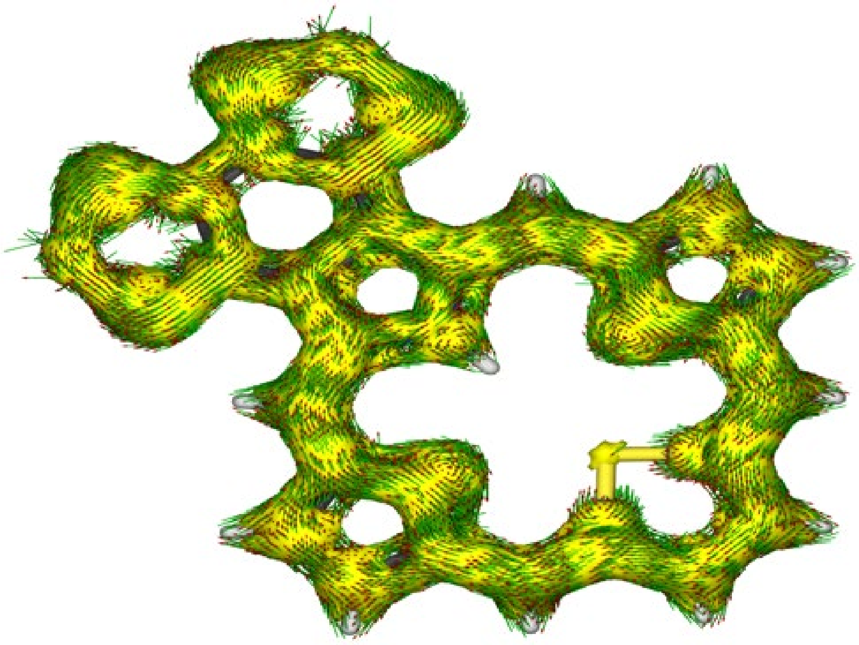

0.07

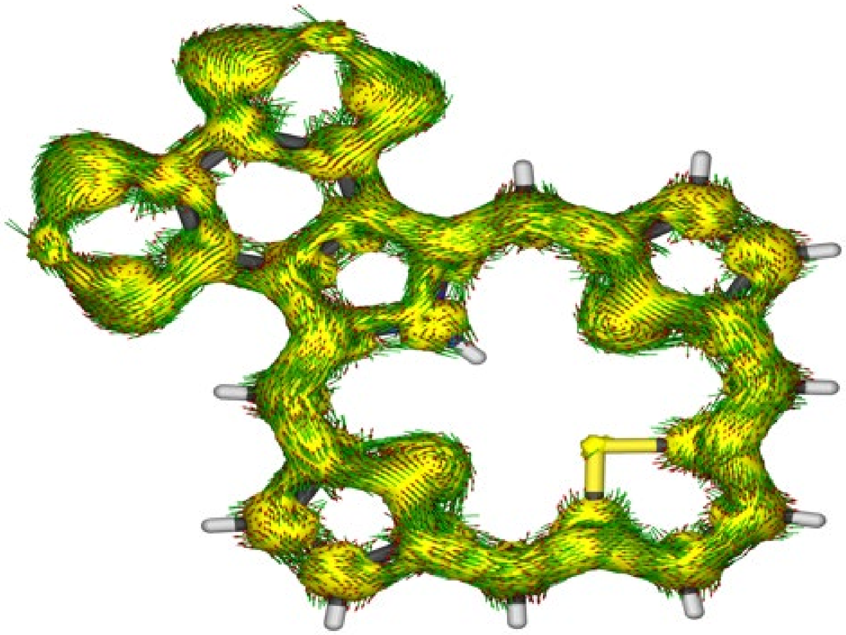

Figure S70. AICD plots for bis(thiadiazolobenzo-thiaporphyrin tautomer **S_2-BTPa** (isovalues 0.05 and 0.07, respectively).

S_2-BTPb

0.05

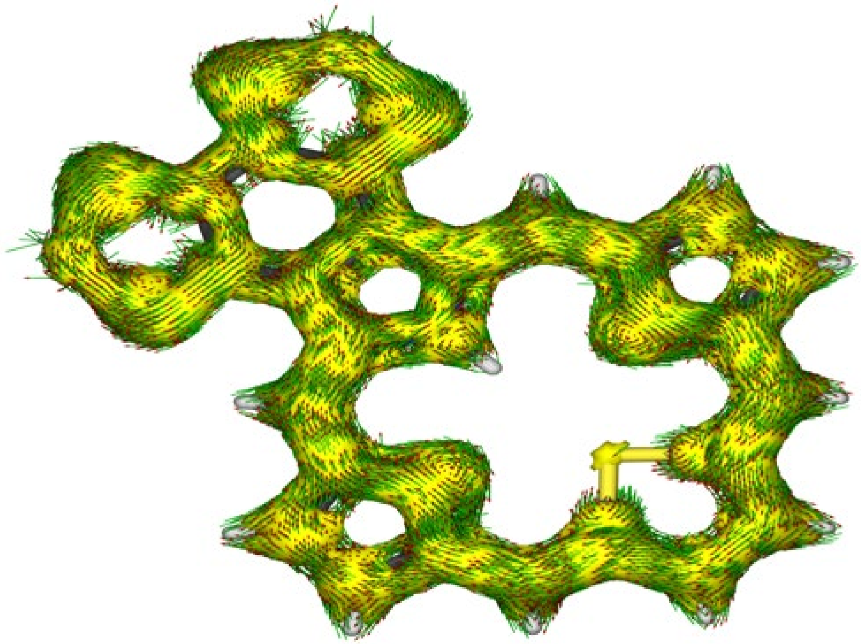

0.07

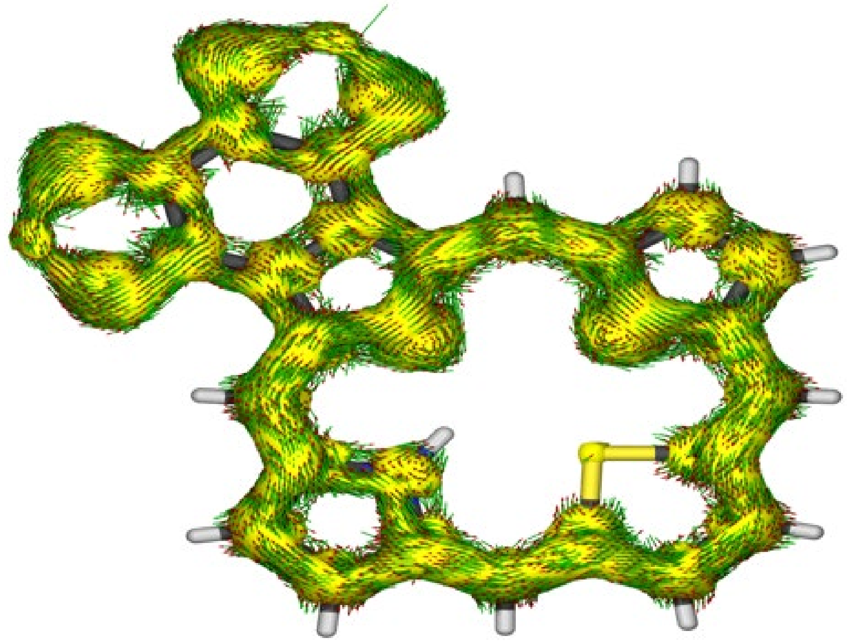

Figure S71. AICD plots for bis(thiadiazolobenzo-thiaporphyrin tautomer **S_2-BTPb** (isovalues 0.05 and 0.07, respectively).

S_2-BSPa

0.05

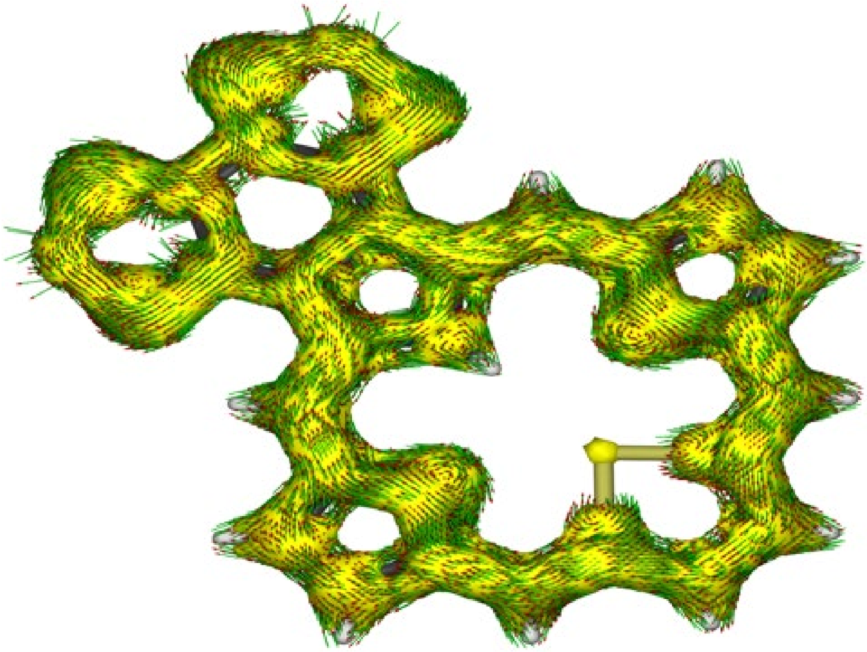

0.07

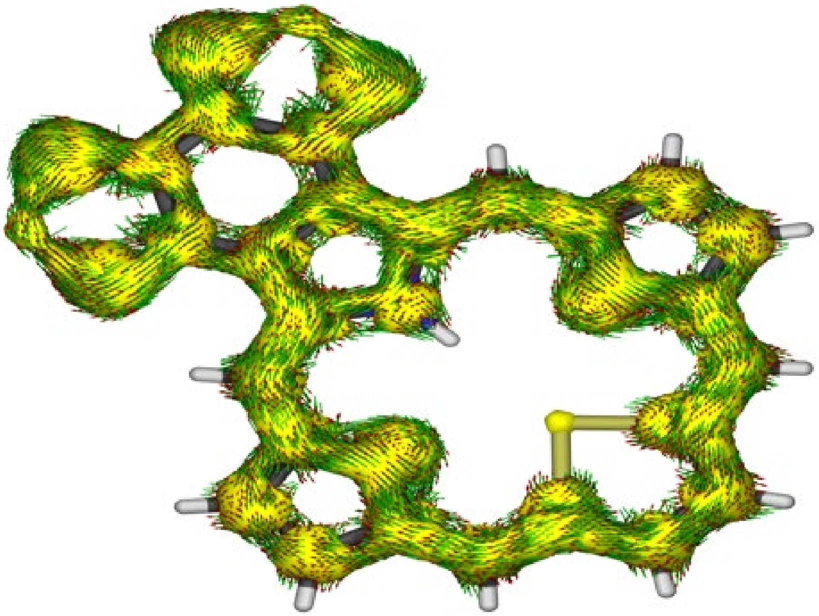

Figure S72. AICD plots for bis(thiadiazolobenzo-selenaporphyrin tautomer **S_2-BSPa** (isovalues 0.05 and 0.07, respectively).

S_2-BSPb

0.05

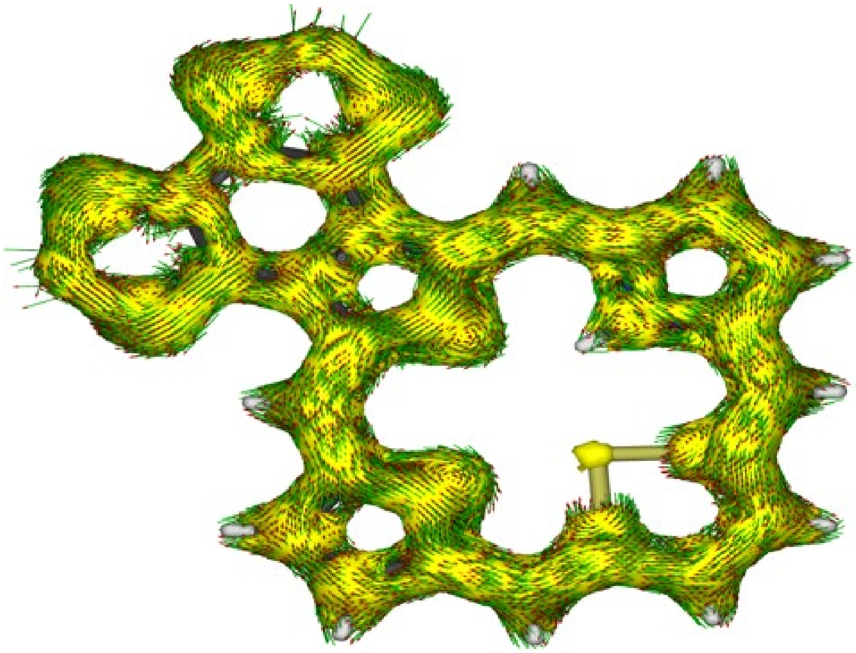

0.07

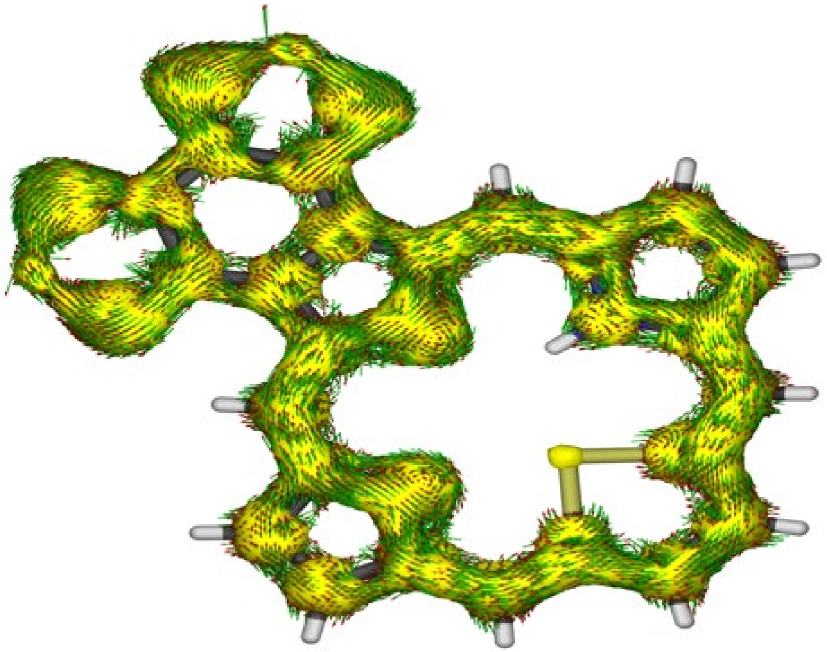

Figure S73. AICD plots for bis(thiadiazolobenzo-selenaporphyrin tautomer **S_2-BSPb**(isovalues 0.05 and 0.07, respectively).

S_2-BCBPa

0.05

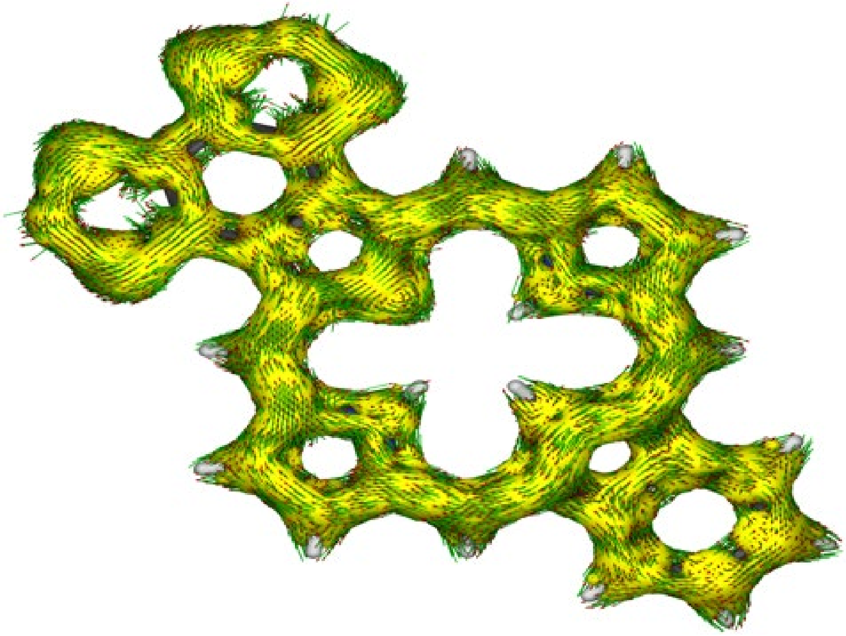

0.07

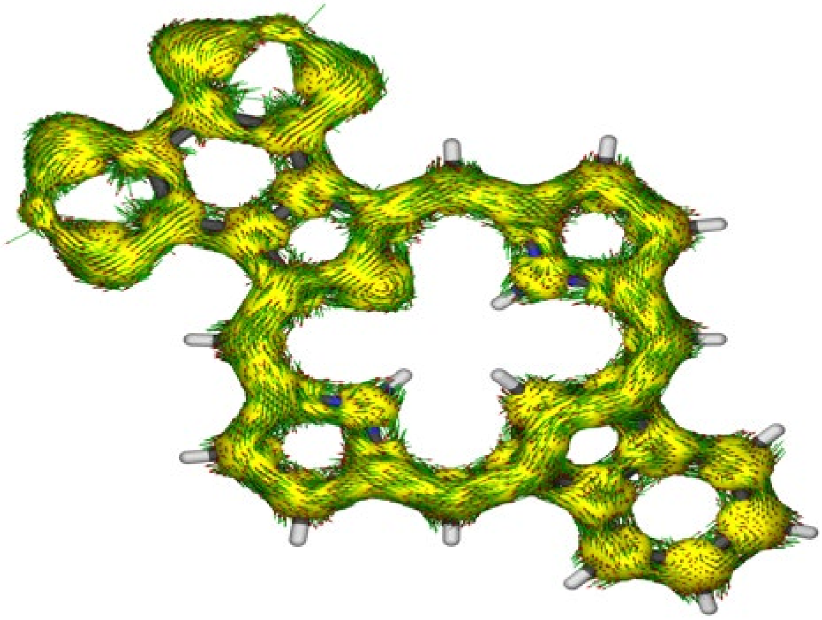

Figure S74. AICD plots for bis(thiadiazolobenzo-benzocarbaporphyrin tautomer **S_2-BCBPa** (isovalues 0.05 and 0.07, respectively).

S_2-BCBPb

<u>0.05:</u>

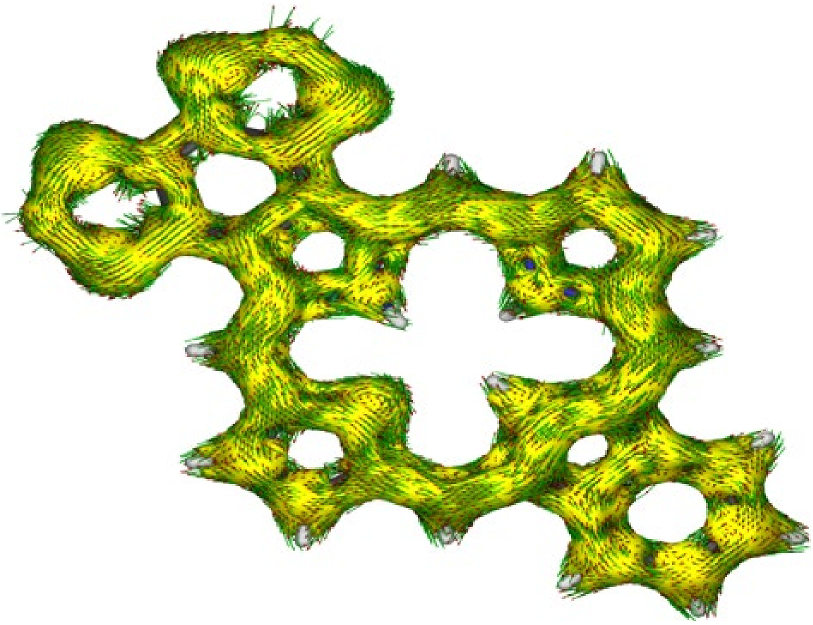

<u>0.07:</u>

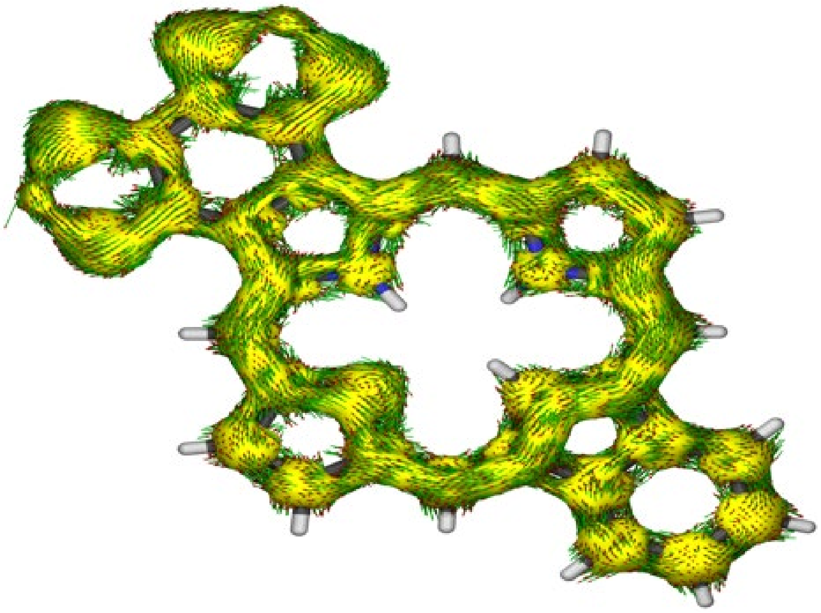

Figure S75. AICD plots for bis(thiadiazolobenzo-benzocarbaporphyrin tautomer **S_2-BCBPb** (isovalues 0.05 and 0.07, respectively).

S_2-BCBPc

0.05

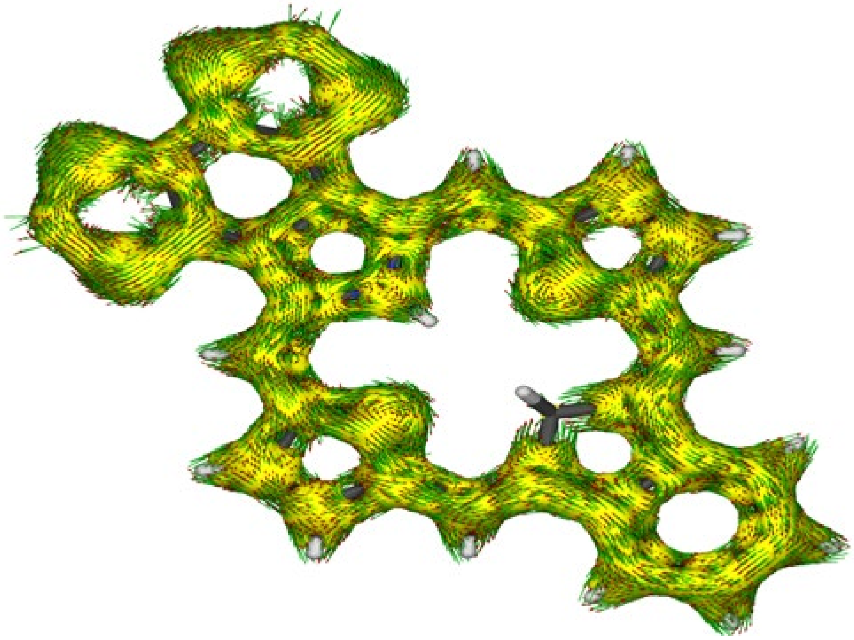

0.07

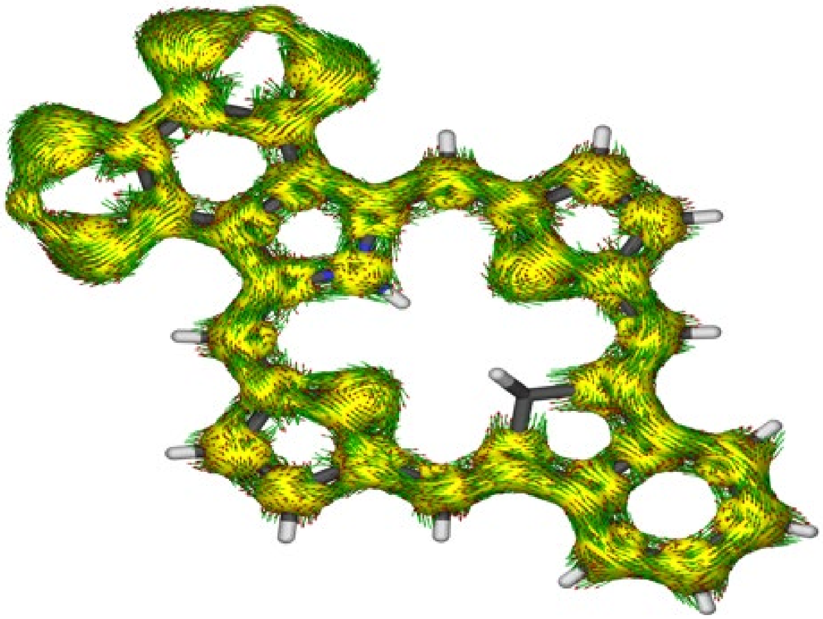

Figure S76. AICD plots for bis(thiadiazolobenzo-benzocarbaporphyrin tautomer **S_2-BCBPc** (isovalues 0.05 and 0.07, respectively).

S_2-BCBPd

0.05

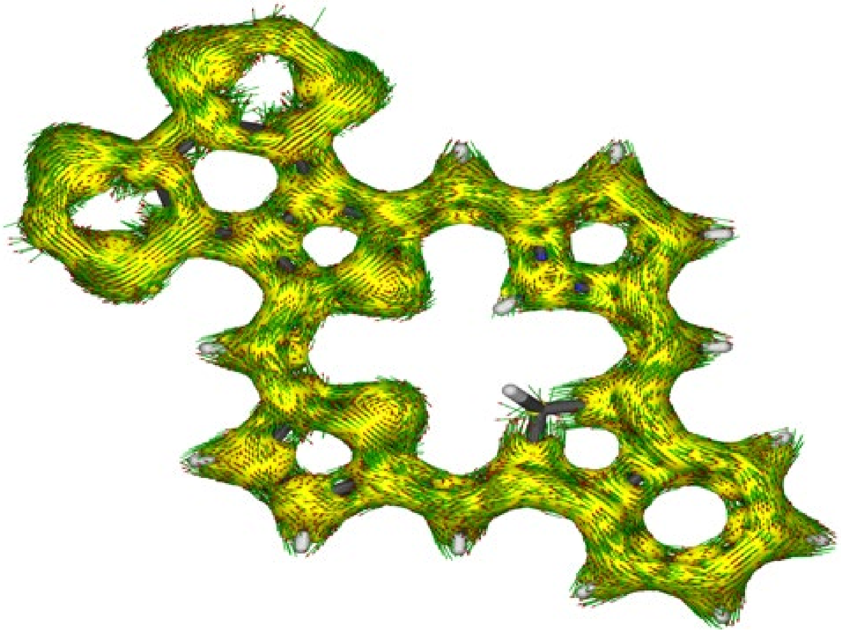

0.07

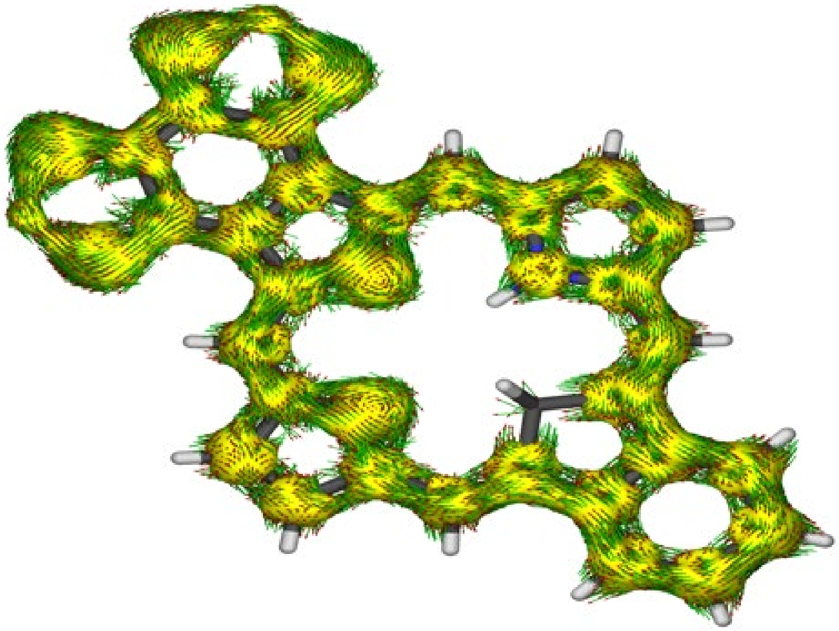

Figure S77. AICD plots for bis(thiadiazolobenzo-benzocarbaporphyrin tautomer **S_2-BCBPd** (isovalues 0.05 and 0.07, respectively).

S_2-OBBPa

0.05

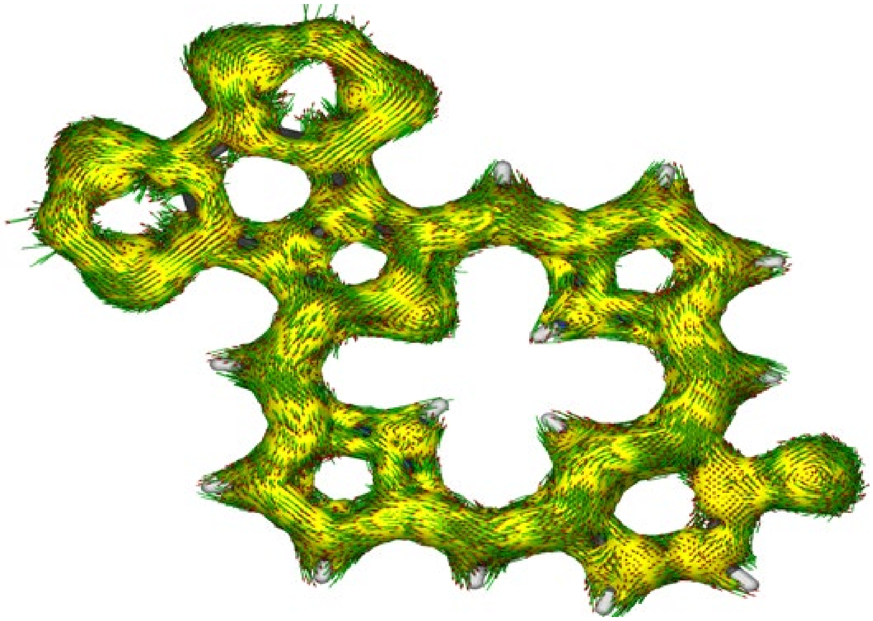

0.07

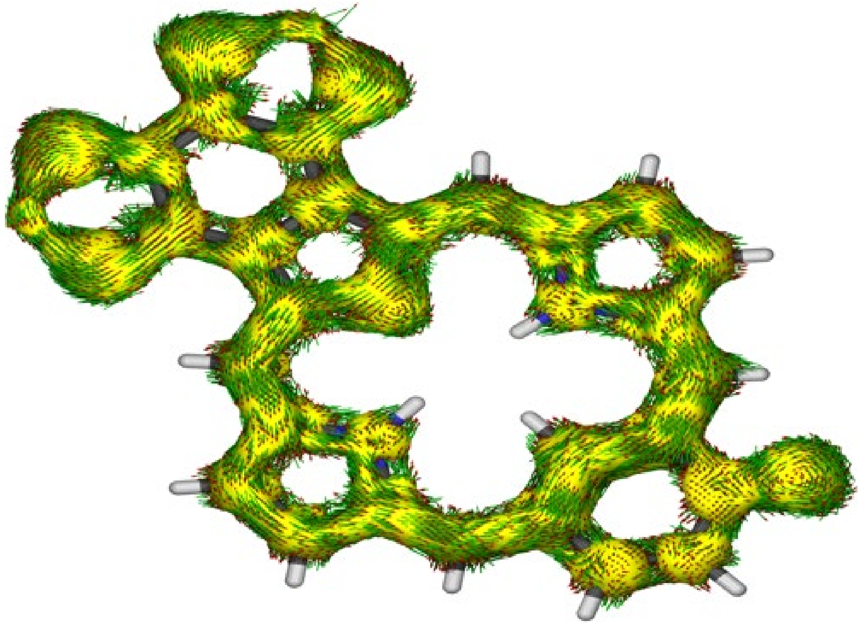

Figure S78. AICD plots for bis(thiadiazolobenzo-oxybenziporphyrin tautomer **S_2-OBBPa** (isovalues 0.05 and 0.07, respectively).

S_2-OBBPb

0.05

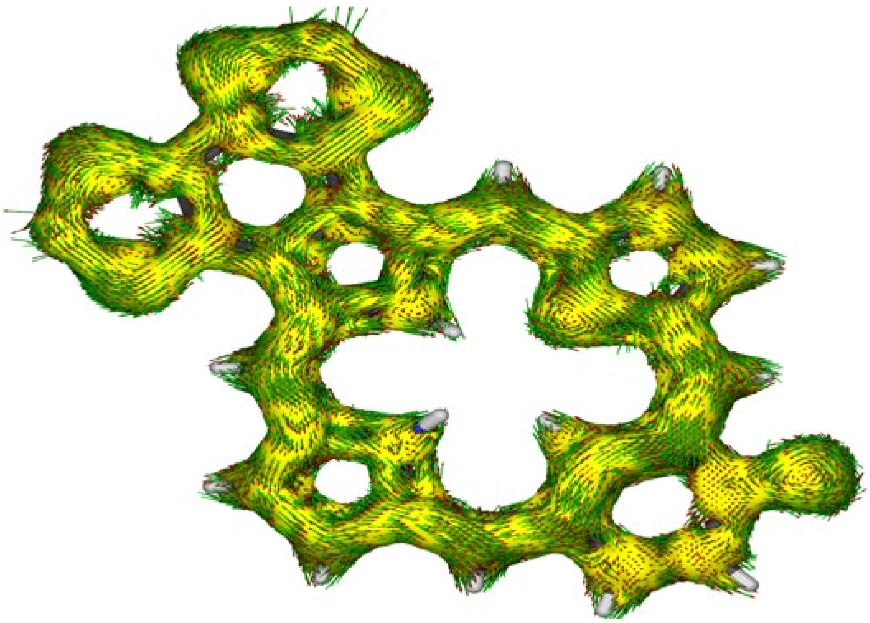

0.07

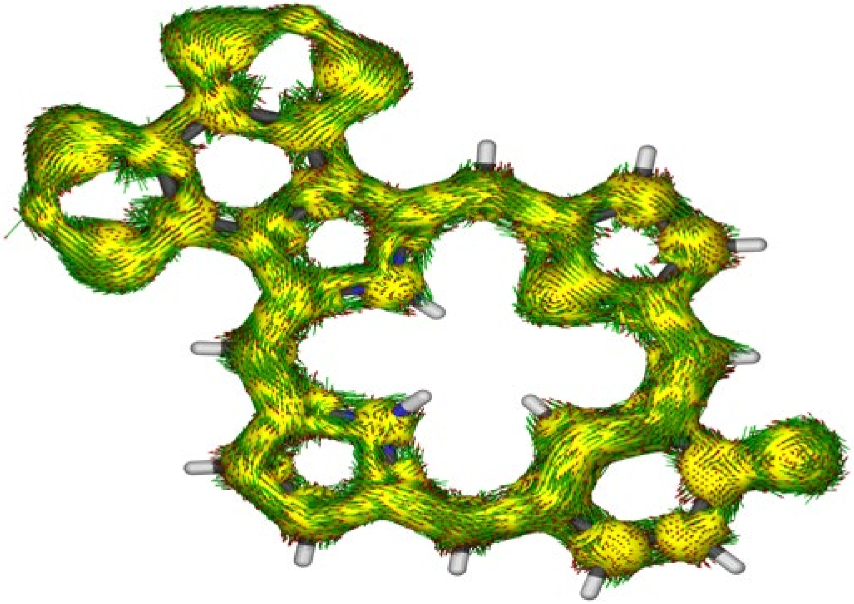

Figure S79. AICD plots for bis(thiadiazolobenzo-oxybenziporphyrin tautomer **S_2-OBBPb** (isovalues 0.05 and 0.07, respectively).

S_2-OBBPc

0.05

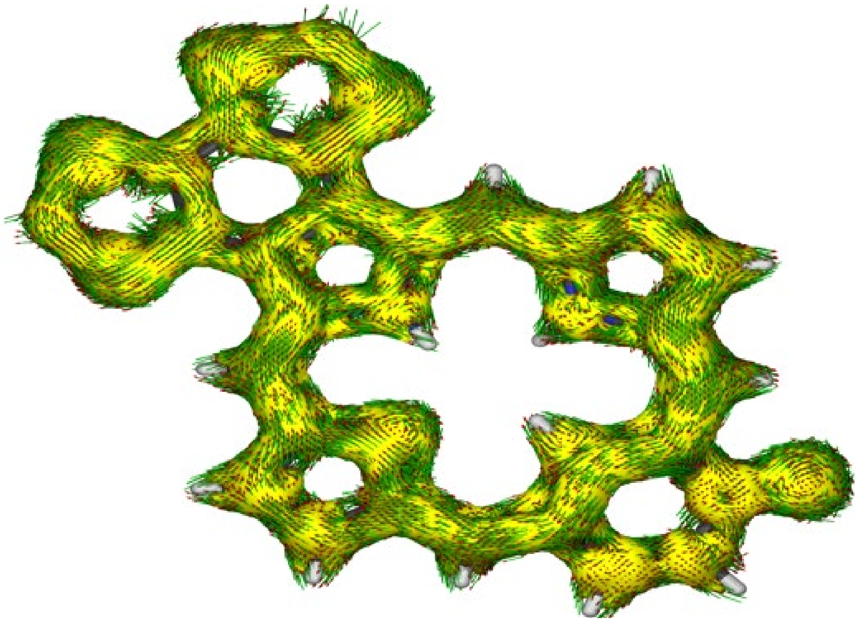

0.07

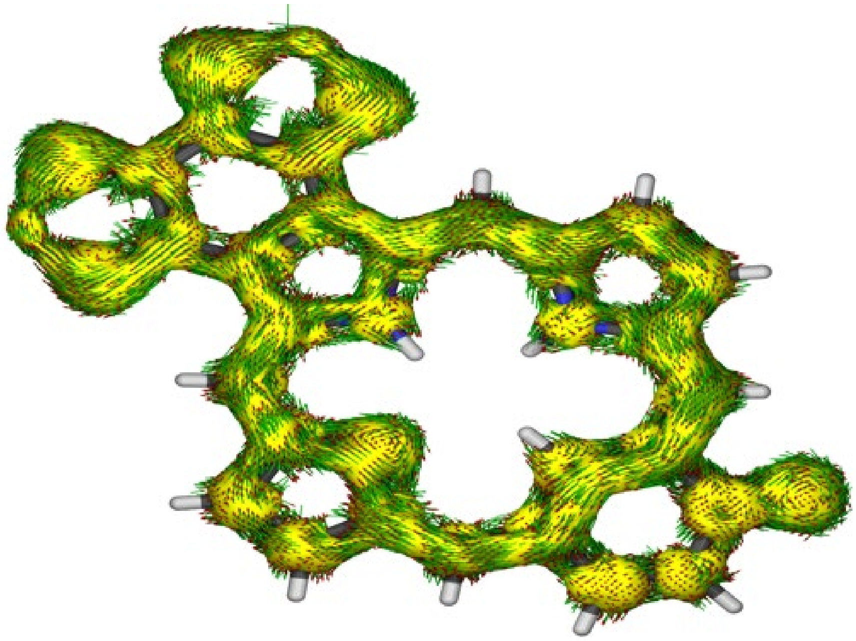

Figure S80. AICD plots for bis(thiadiazolobenzo-oxybenziporphyrin tautomer **S_2-OBBPc** (isovalues 0.05 and 0.07, respectively).

S_2-OBBPd

0.05

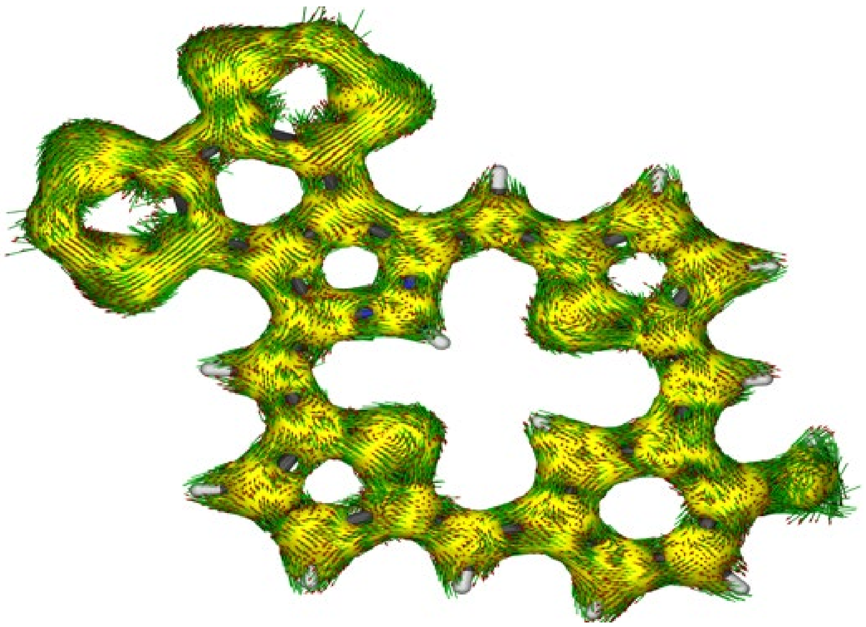

0.07

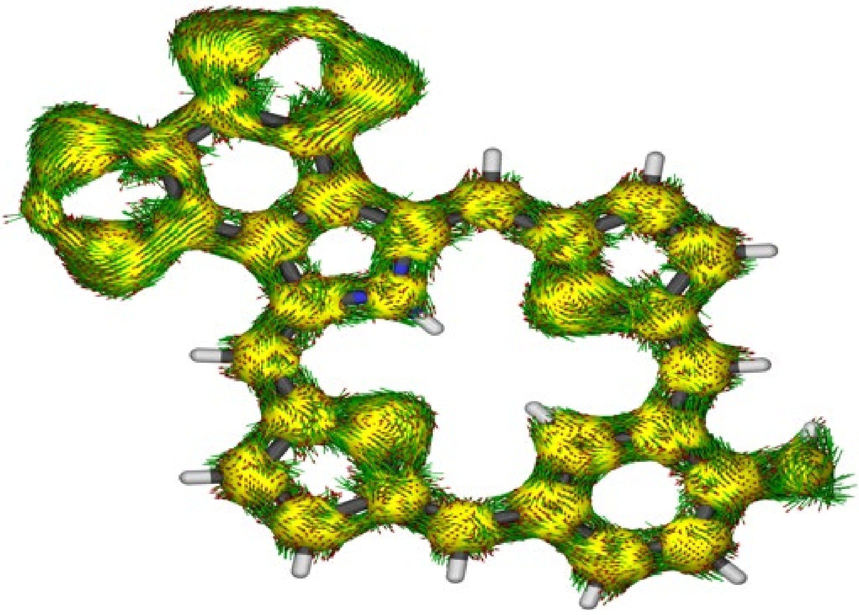

Figure S81. AICD plots for bis(thiadiazolobenzo-oxybenziporphyrin tautomer **S_2-OBBPd** (isovalues 0.05 and 0.07, respectively).

S_2-OPBPa

0.05

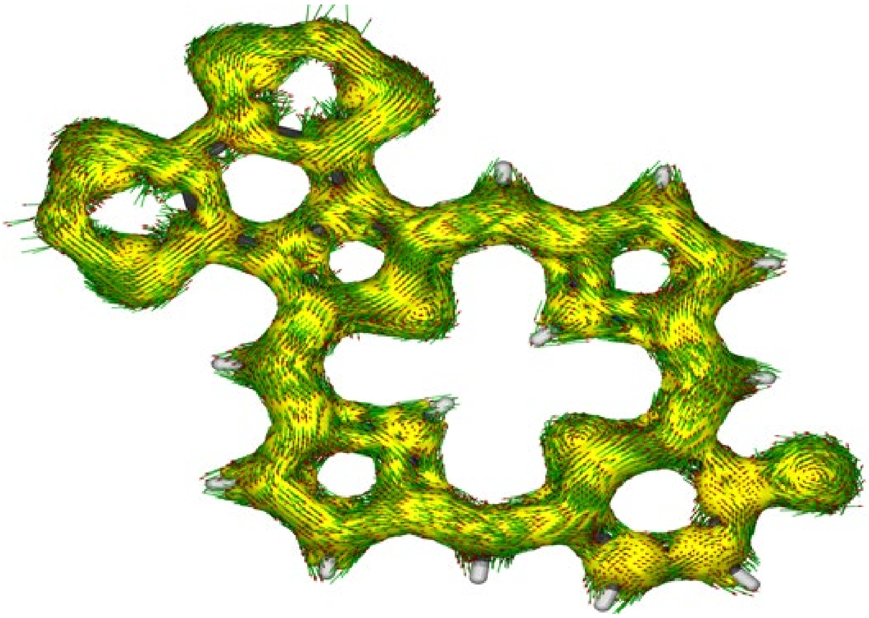

0.07

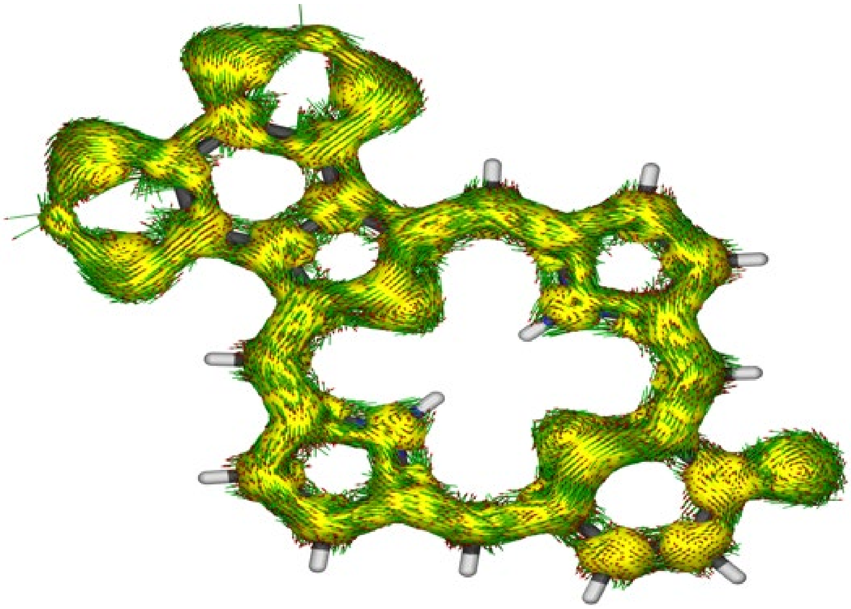

Figure S82. AICD plots for bis(thiadiazolobenzo-oxypyriporphyrin tautomer **S_2-OPBPa** (isovalues 0.05 and 0.07, respectively).

S_2-OPBPb

0.05

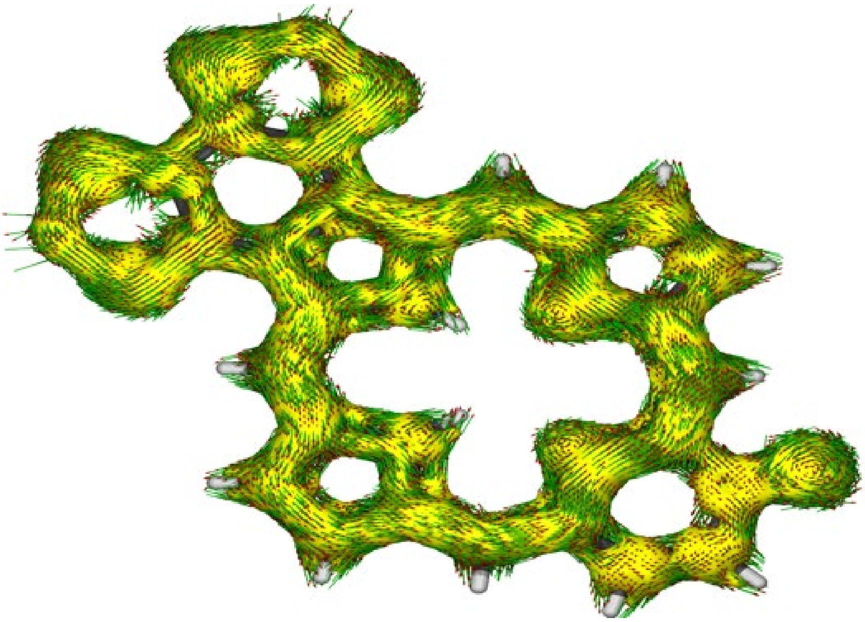

0.07

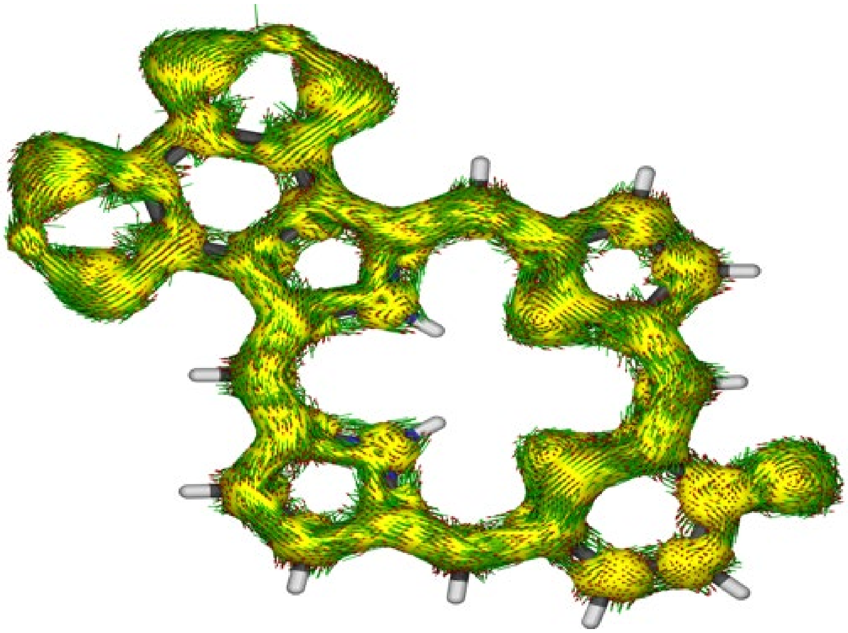

Figure S83. AICD plots for bis(thiadiazolobenzo-oxypyriporphyrin tautomer **S_2-OPBPb** (isovalues 0.05 and 0.07, respectively).

S$_2$-OPBPc

0.05

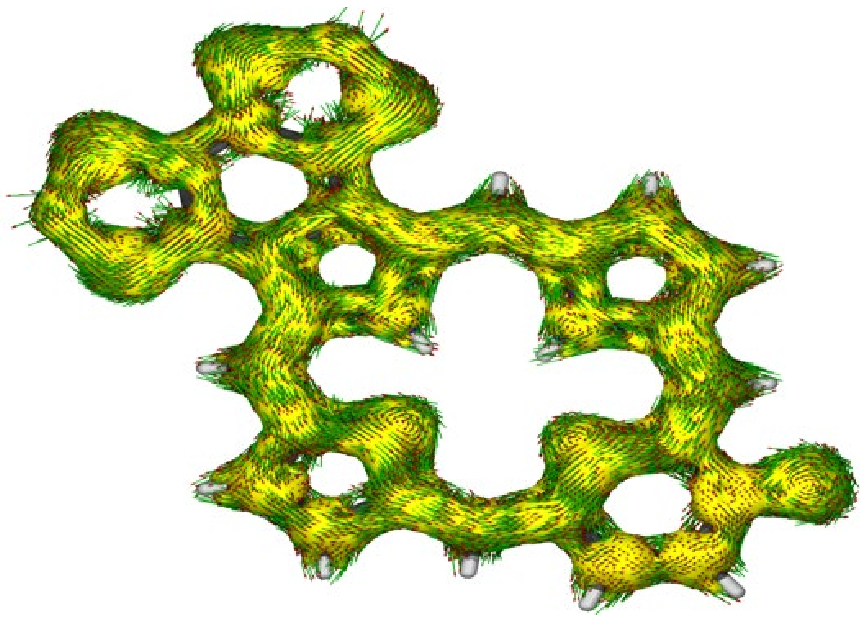

0.07

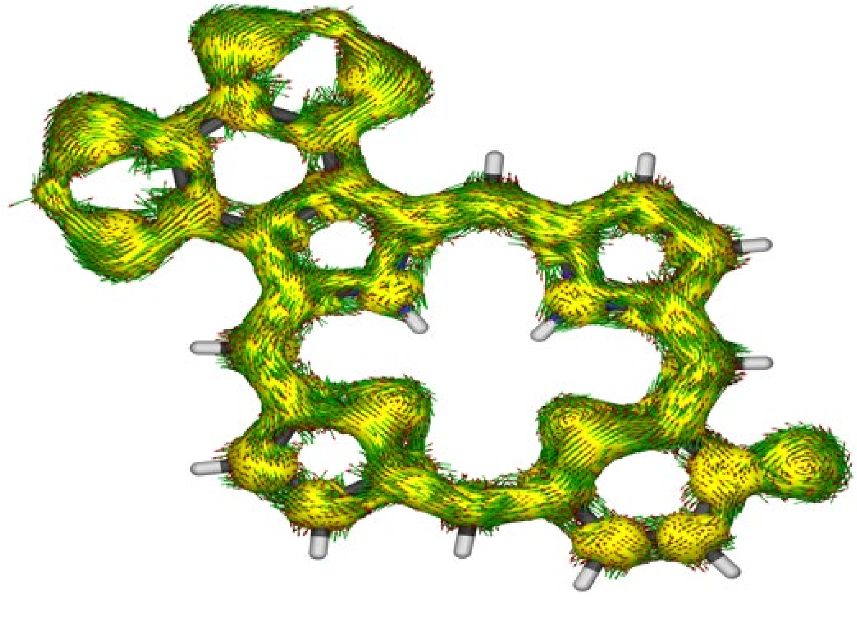

Figure S84. AICD plots for bis(thiadiazolobenzo-oxypyriporphyrin tautomer **S$_2$-OPBPc** (isovalues 0.05 and 0.07, respectively).

S_2-OPBPd

0.05

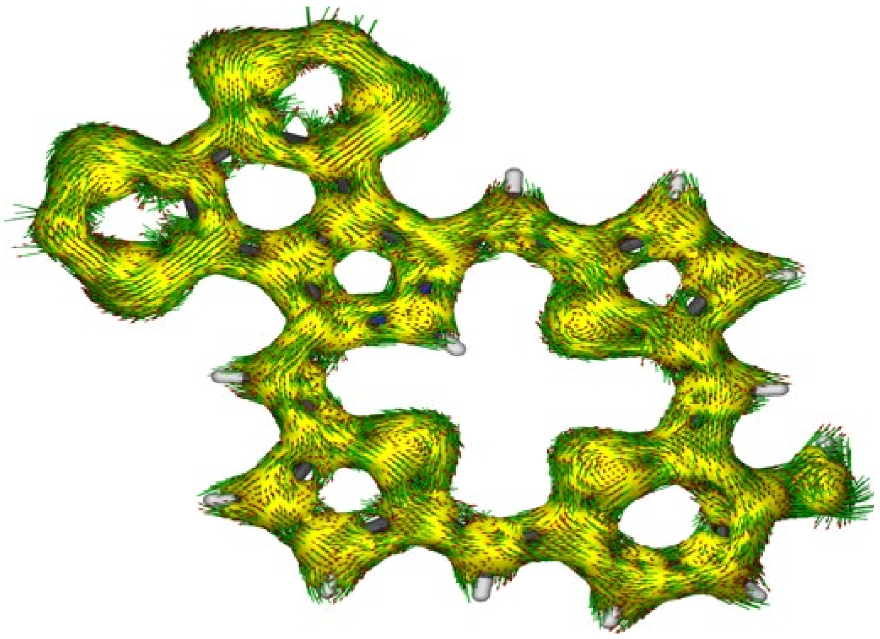

0.07

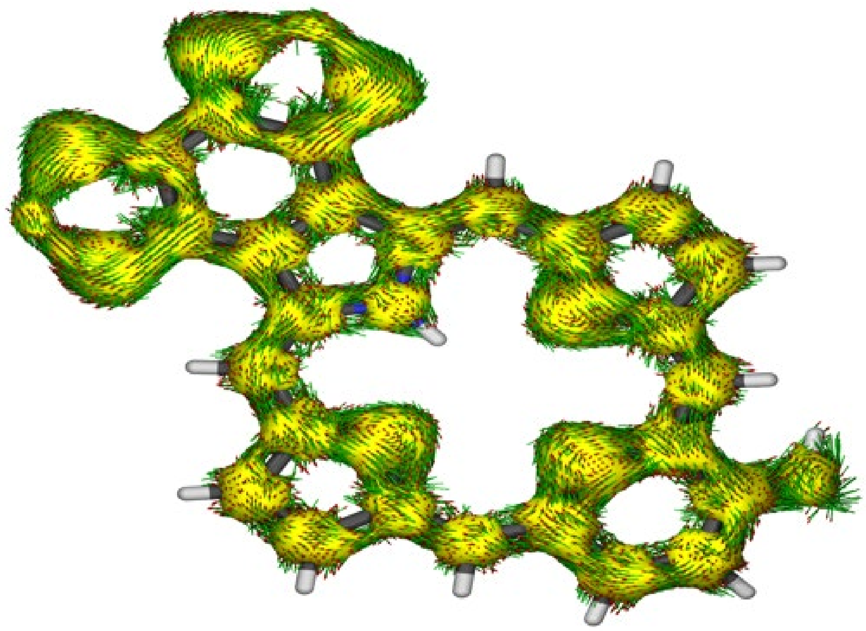

Figure S85. AICD plots for bis(thiadiazolobenzo-oxypyriporphyrin tautomer **S_2-OPBPd** (isovalues 0.05 and 0.07, respectively).

S_2-OPBPe

0.05

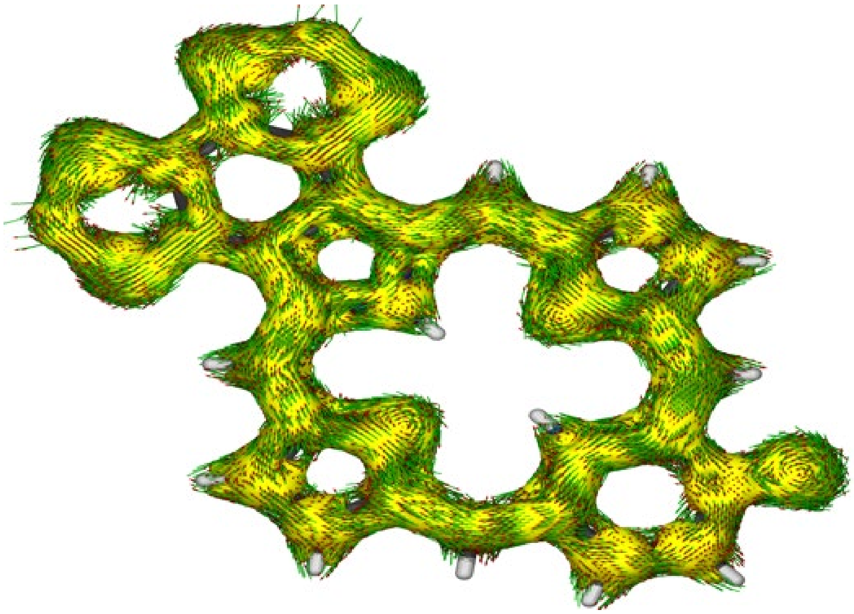

0.07

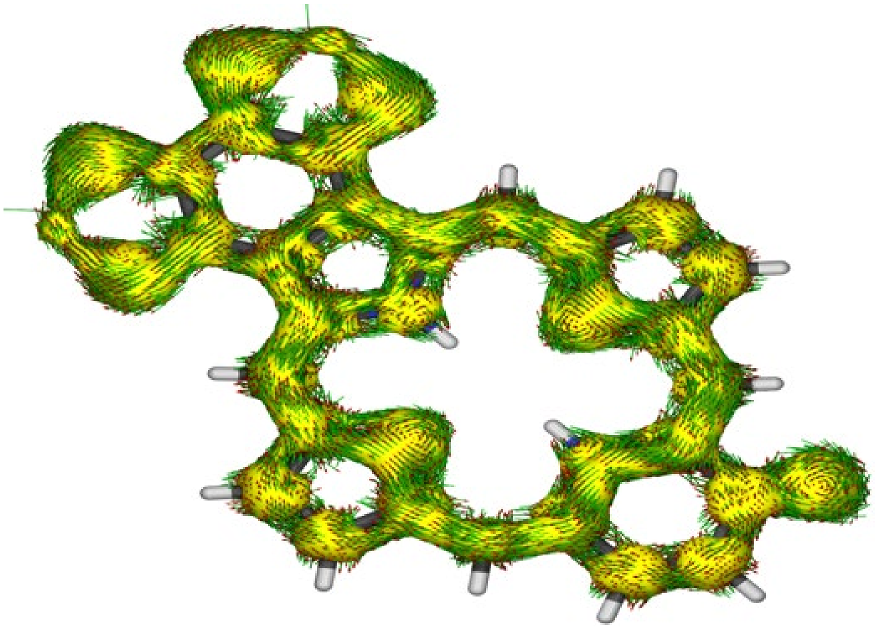

Figure S86. AICD plots for bis(thiadiazolobenzo-oxypyriporphyrin tautomer **S_2-OPBPe** (isovalues 0.05 and 0.07, respectively).

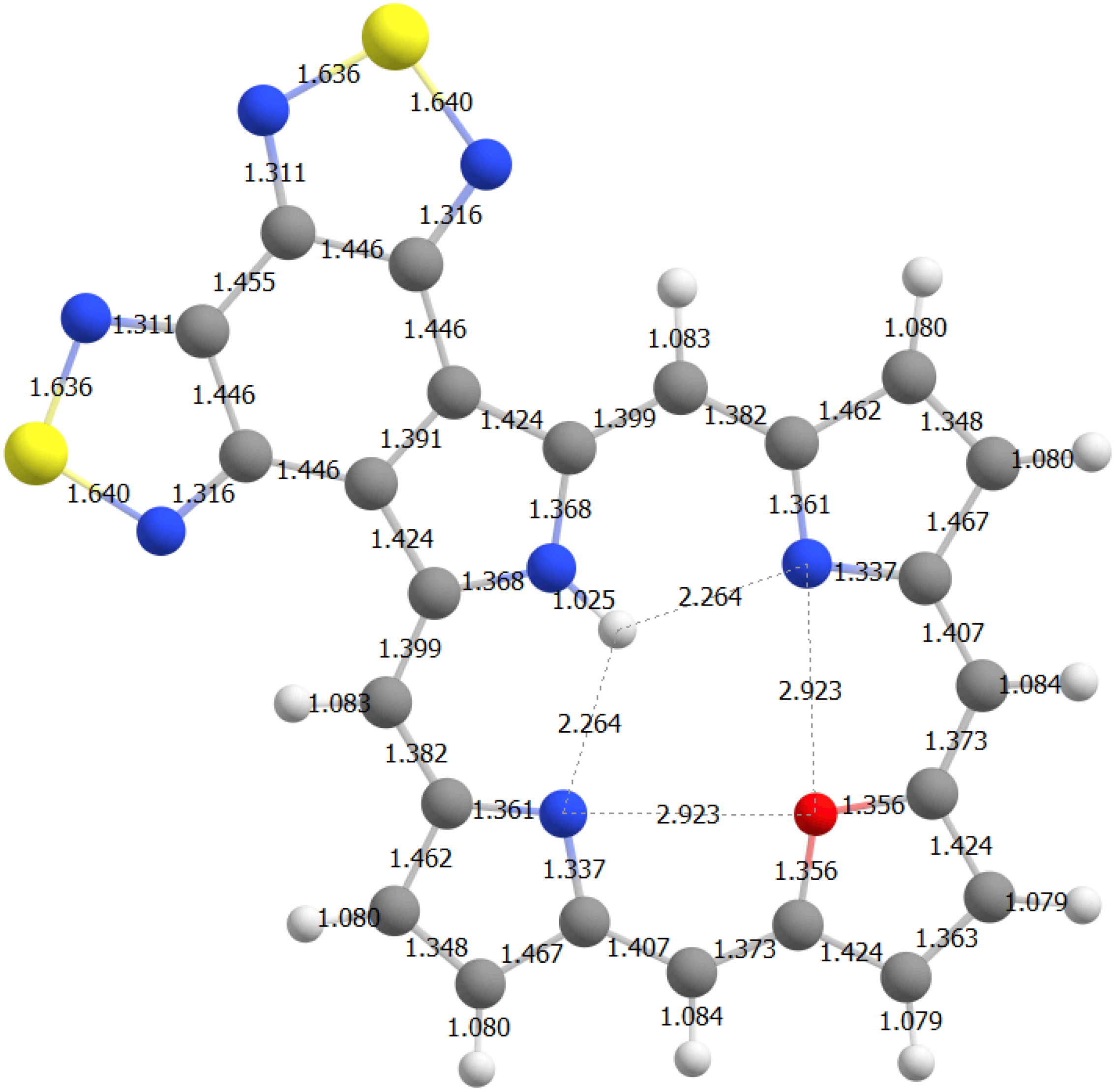

Figure S87. Calculated bond lengths for oxaporphyrin tautomer **S2-Ox-BPa**.

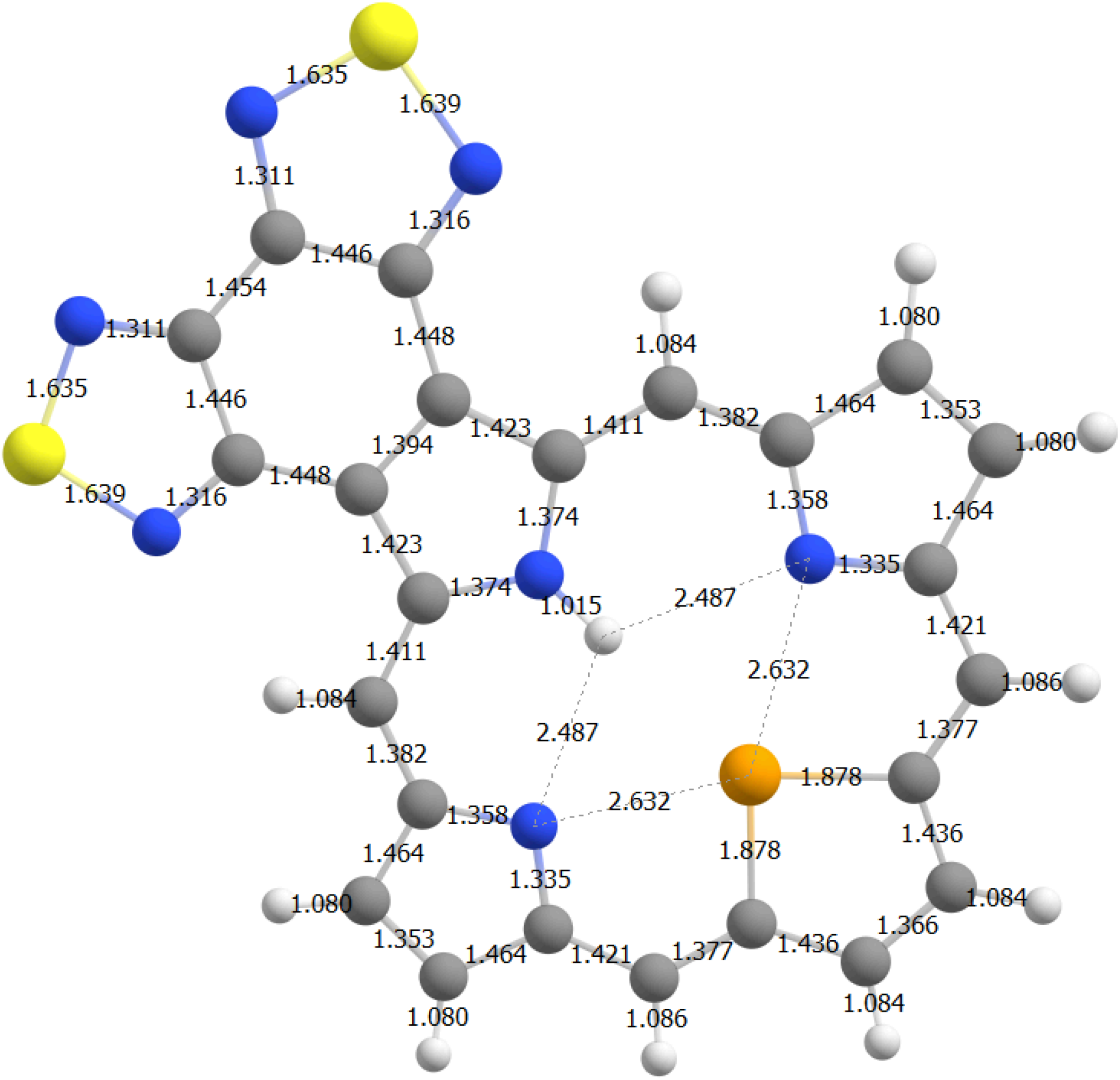

Figure S88. Calculated bond lengths for selenaporphyrin tautomer **S$_2$-BSPa**.

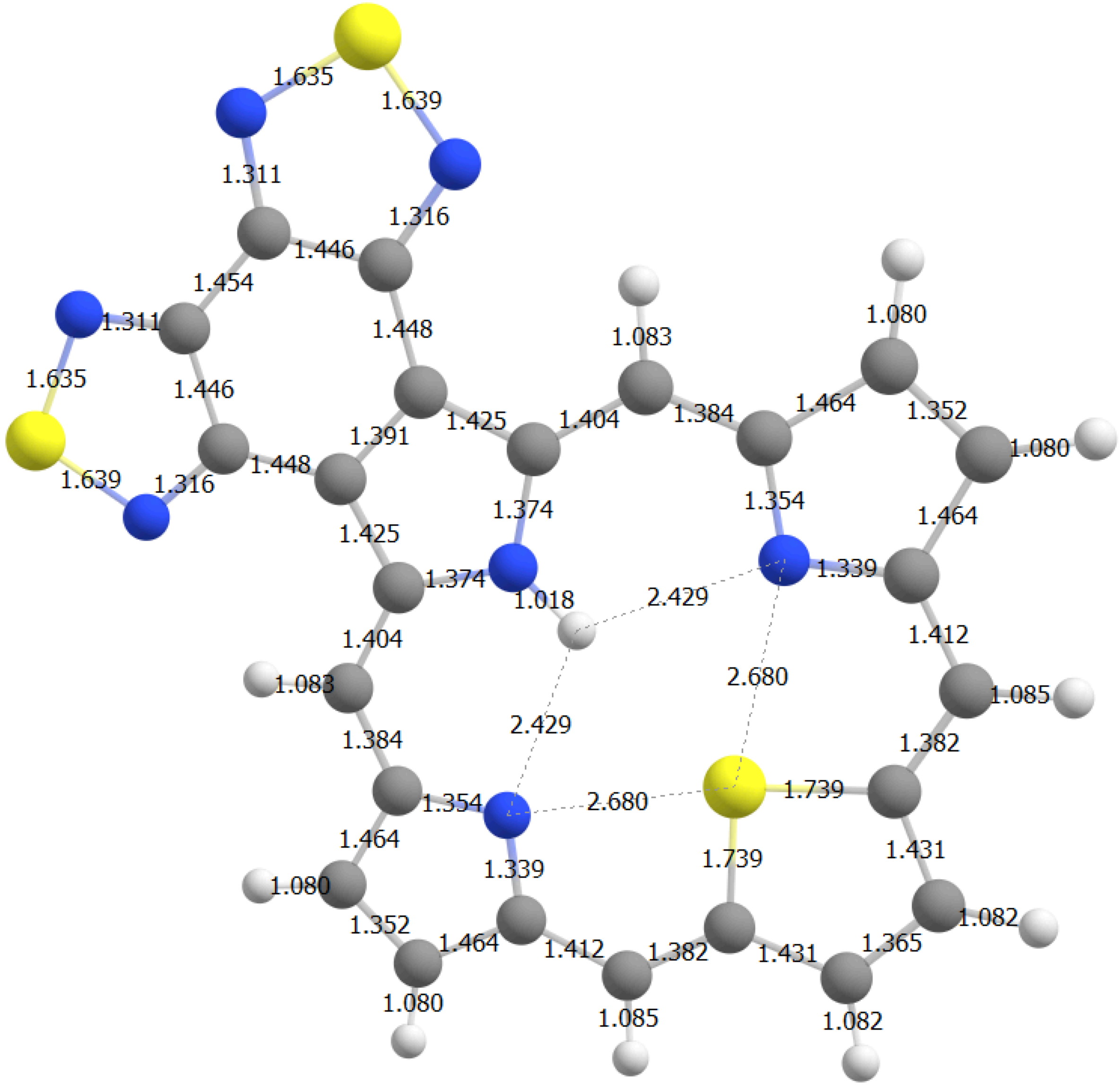

Figure S89. Calculated bond lengths for thiaporphyrin tautomer **S_2-BTPa**.

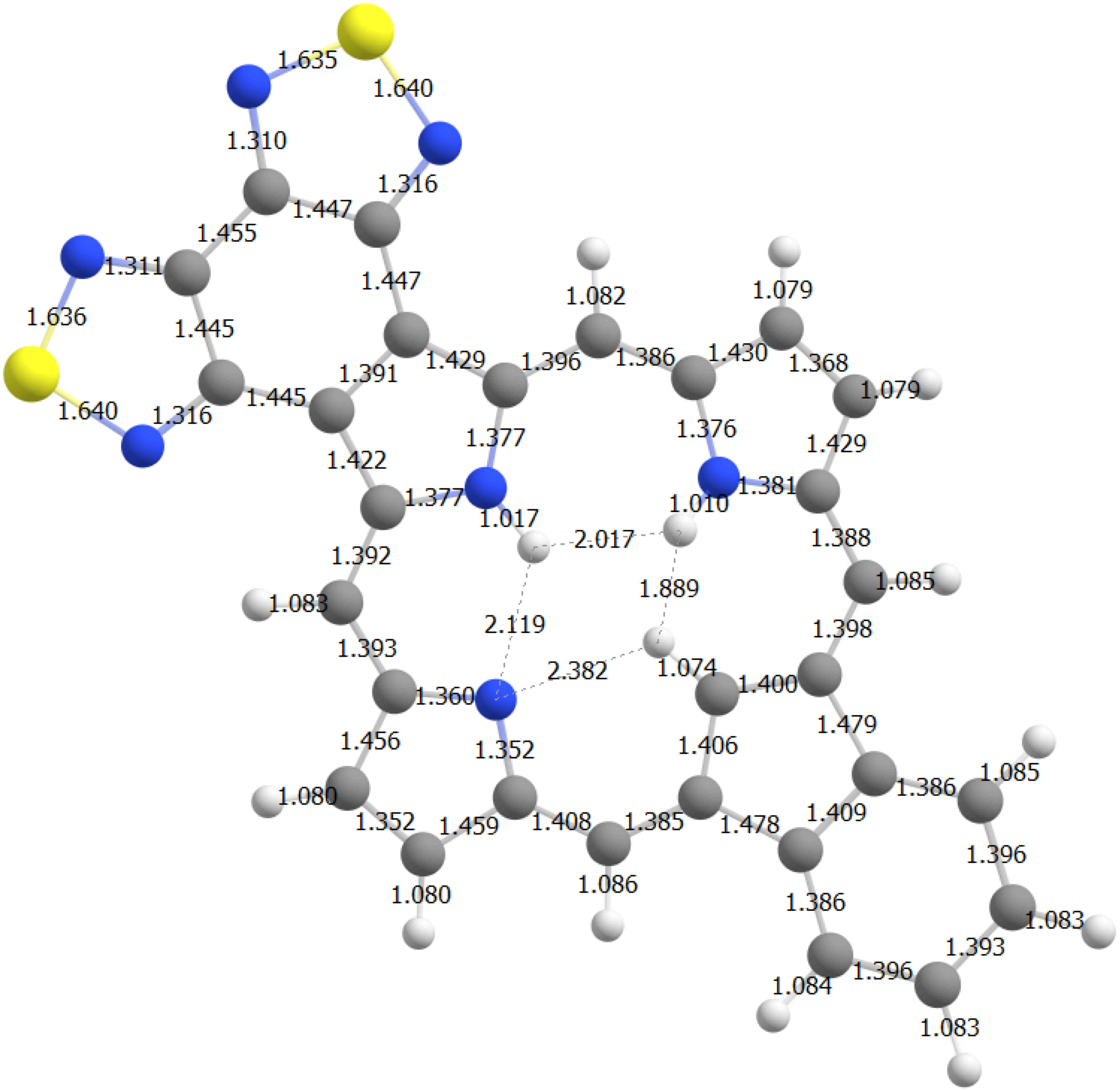

Figure S90. Calculated bond lengths for benzocarbaporphyrin tautomer **S_2-BCBPa**.

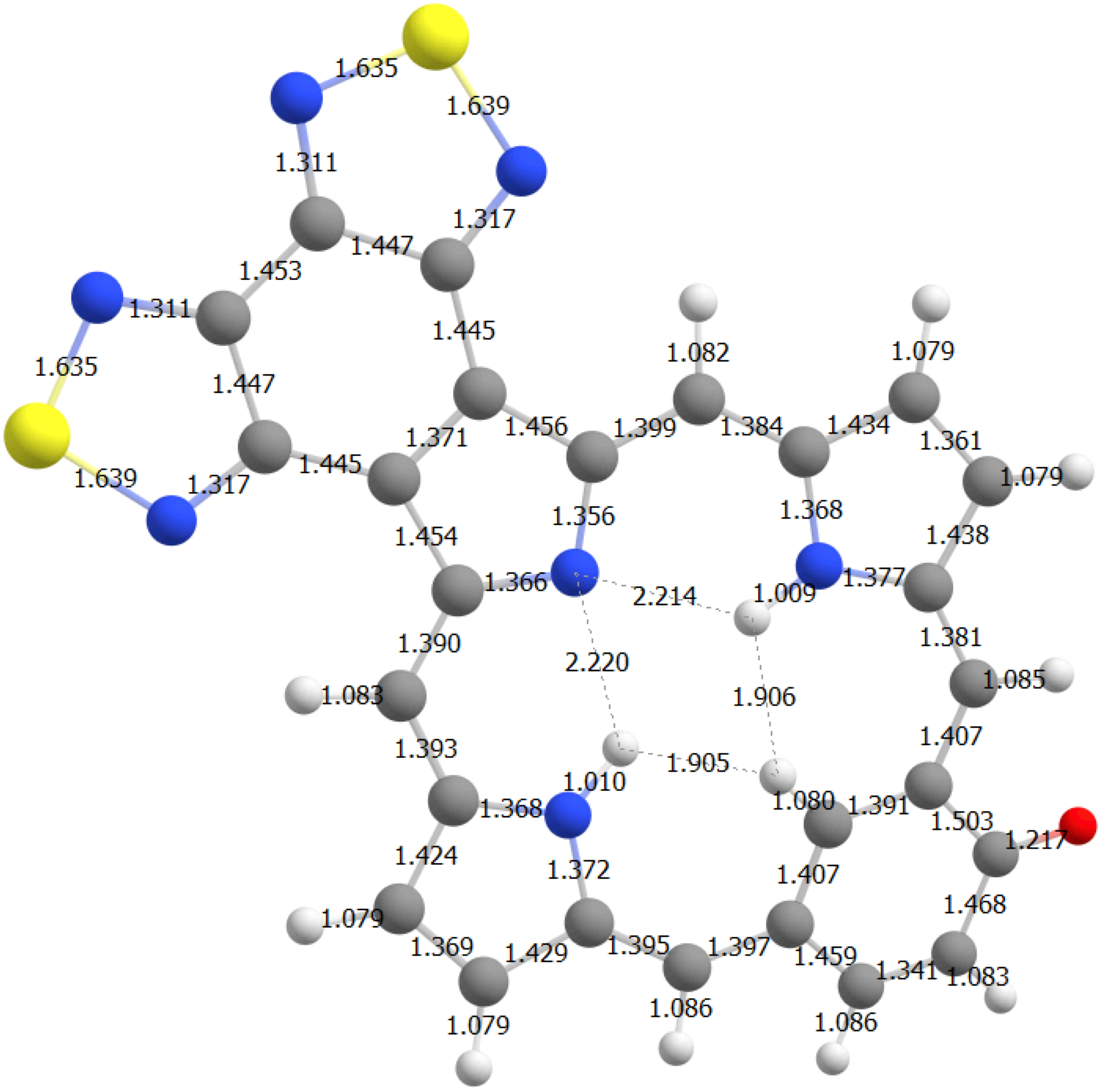

Figure S91. Calculated bond lengths for thiaporphyrin tautomer **S_2-OBBPa**.

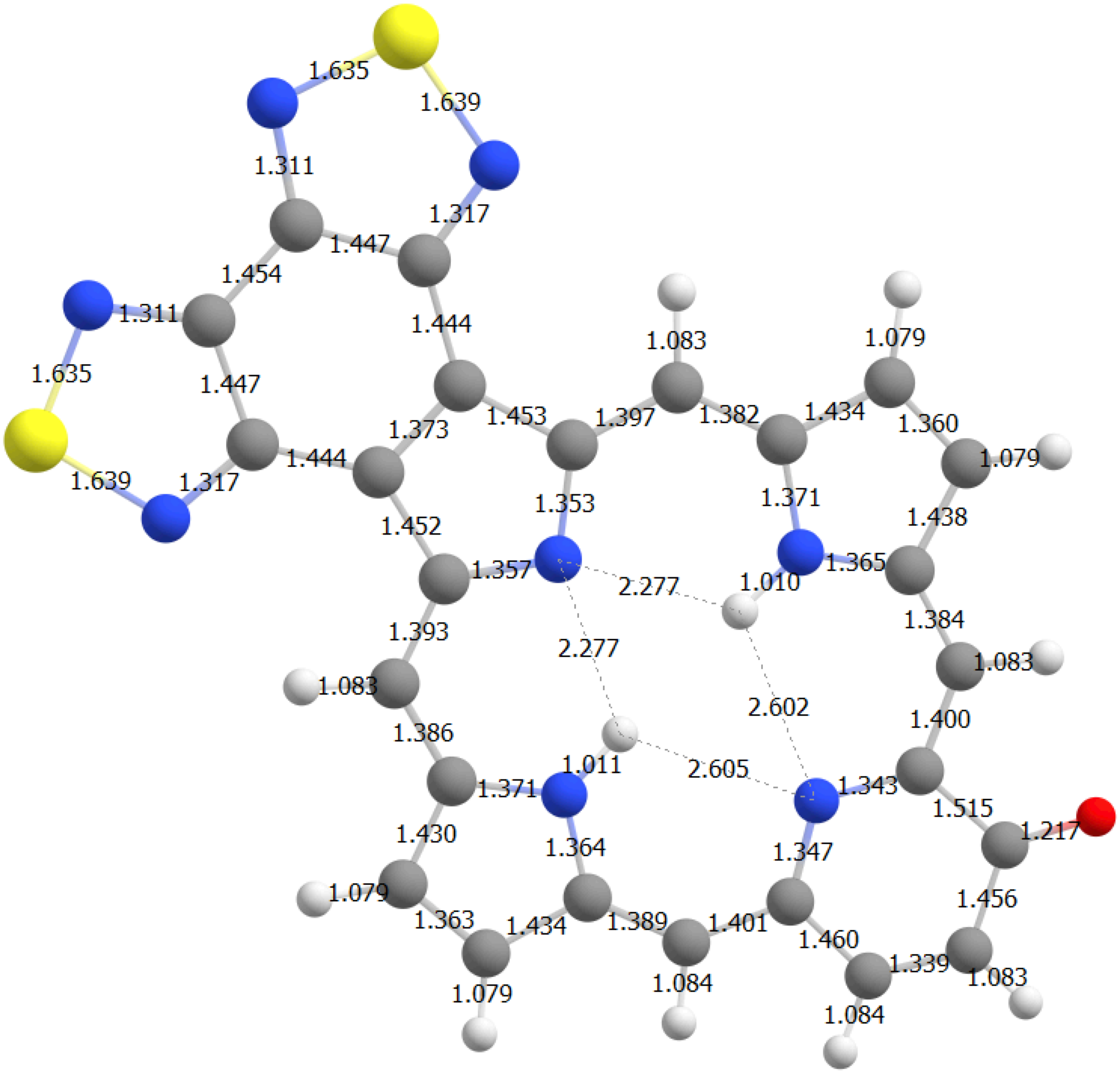

Figure S92. Calculated bond lengths for oxypyriporphyrin tautomer **S_2-OPBPa**.

Table S1. Calculated Gibb's free energies (kcal/mol) and electronic energies (kcal/mol) for Bis(thiadiazolo)benzoporphyrin tautomers and related porphyrinoid systems. Free energies were calculated using M06-2X/6-311++G(dip). Electronic energies calculated using M06-2X/cc-PVTZ.

Molecule	E (Hartrees)	G (Hartrees)	Delta E (kcal/mol)	Delta G (kcal/mol)
	M06-2X cc-PVTZ	M06-2X 6-311++G(d,p)	M06-2X cc-PVTZ	M06-2X 6-311++G(d,p)
S2-BPa	-2156.117602	-2155.680402	2.18	2.13
S2-BPb	-2156.121075	-2155.683792	0.00	0.00
S2-BPc	-2156.106827	-2155.669958	8.94	8.68
S2-BPd	-2156.103298	-2155.666584	11.16	10.80
S2-OxBPa	-2175.956253	-2175.528299	0.00	0.00
S2-OxBPb	-2175.952424	-2175.524883	2.40	2.14
S2-BTPa	-2498.931765	-2498.506468	0.00	0.00
S2-BTPb	-2498.921697	-2498.496080	6.32	6.52
S2-BSPa	-4502.350852	-4501.866640	0.00	0.00
S2-BSPb	-4502.339518	-4501.855709	7.11	6.86
S2-BCBPa	-2293.695194	-2293.186358	0.00	0.00
S2-BCBPb	-2293.682967	-2293.175156	7.67	7.03
S2-BCBPc	-2293.678525	-2293.172476	10.46	8.71
S2-BCBPd	-2293.670418	-2293.164193	15.55	13.91
S2-OBBPa	-2253.389689	-2252.922988	0.00	0.00
S2-OBBPb	-2253.379079	-2252.912513	6.66	6.57
S2-OBBPc	-2253.380513	-2252.913940	5.76	5.68
S2-OBBPd	-2253.371503	-2252.905025	11.41	11.27
S2-OPBPa	-2269.443630	-2268.989605	0.00	0.00
S2-OPBPb	-2269.428388	-2268.974162	9.56	9.69
S2-OPBPc	-2269.429873	-2268.975425	8.63	8.90
S2-OPBPd	-2269.399417	-2268.944316	27.74	28.42
S2-OPBPe	-2269.436267	-2268.980893	4.62	5.47

Table S2. Cartesian coordinates for bis(thiadiazolo)benzene-fused porphyrin tautomers and related porphyrinoid systems

S_2-BCBPa

Frequencies:

19.7127	25.3669	51.9211
67.9738	68.5019	73.8126
107.3921	108.0549	109.3521
125.0553	126.2288	151.5732
177.9663	178.1218	191.6106
209.1243	216.9928	242.7552
254.1879	261.4853	262.1475
284.3949	325.9915	330.0466
333.6566	340.4468	382.1853
382.6712	402.8916	413.5394
420.8470	455.9592	478.5540
489.9266	493.9853	501.3652
519.3760	530.7137	565.1920
573.4272	594.7377	606.9233
624.3695	636.3347	658.5824
671.9143	675.7100	684.0004
688.5816	690.5365	694.0240
697.8028	704.8956	723.6165
733.8374	744.1009	746.4633
746.8778	752.0460	770.9633
775.6837	787.5772	794.0168
802.6371	804.3229	809.0428
811.5636	838.1923	845.4082
853.2924	858.7579	867.5433
873.4989	874.7539	883.5890
891.0200	902.0946	902.8765
908.5770	943.0960	944.2633
950.6782	967.6418	1004.4681
1005.7643	1018.4059	1022.1307
1026.0985	1041.0719	1043.7570
1055.5534	1079.6798	1080.4863
1116.9337	1131.8979	1150.3366
1178.7568	1181.3994	1198.8862
1200.4190	1213.0871	1240.2551
1268.1445	1270.1869	1275.6252
1281.8946	1309.9439	1317.5168
1329.6062	1348.9694	1362.5556
1388.5022	1396.5355	1411.4870
1413.6594	1426.8057	1438.7487
1447.9468	1455.3890	1462.5335
1470.4139	1479.6407	1506.2460
1507.3995	1521.7854	1544.4307
1565.8588	1575.4536	1577.9352
1589.3979	1599.1542	1617.8495
1632.3613	1649.2755	1656.7045
1658.9297	1666.6872	1678.2459
3197.9505	3201.1877	3203.4748
3204.9328	3214.4765	3226.7843
3231.2800	3231.4211	3265.5065
3265.5519	3273.8173	3282.3803
3282.4000	3632.8868	3664.9350

Coordinates:

C	-0.759811000	-4.290260000	-0.292866000
C	-2.126261000	-4.290272000	-0.341912000
C	-2.580596000	-2.933382000	-0.273894000
N	-1.454542000	-2.157983000	-0.192260000
C	-0.325092000	-2.931566000	-0.187682000
C	2.738985000	0.687031000	0.039624000
C	2.739006000	-0.686989000	0.039682000
C	1.348003000	-1.093671000	-0.030705000
N	0.536767000	-0.000010000	-0.062118000
C	1.347978000	1.093640000	-0.030700000
C	0.965994000	-2.439489000	-0.092829000
C	-2.126322000	4.290237000	-0.341849000
C	-0.759890000	4.290240000	-0.292990000
C	-0.325134000	2.931555000	-0.187621000
N	-1.454574000	2.157968000	-0.192022000
C	-2.580636000	2.933314000	-0.273967000
C	0.965972000	2.439460000	-0.092807000
C	-5.680740000	-0.703794000	0.100214000
C	-5.680724000	0.703802000	0.100233000
C	-4.311802000	1.155754000	-0.220478000
C	-3.540200000	-0.000003000	-0.436142000
C	-4.311824000	-1.155776000	-0.220450000
C	-3.900435000	-2.484062000	-0.248828000
C	-3.900404000	2.484053000	-0.248912000
C	3.976285000	1.433097000	0.090556000
C	5.238739000	0.726642000	0.140710000
C	5.238746000	-0.726631000	0.140759000
C	3.976285000	-1.433076000	0.090674000
C	-6.835726000	-1.410927000	0.399226000
C	-8.001198000	-0.697175000	0.677438000
C	-8.001180000	0.697213000	0.677459000
C	-6.835694000	1.410949000	0.399266000
N	6.286961000	-1.513086000	0.181542000
S	5.735060000	-3.052249000	0.158475000
N	4.126433000	-2.741871000	0.096519000
N	4.126463000	2.741897000	0.096356000
S	5.735074000	3.052281000	0.158608000
N	6.286947000	1.513099000	0.181361000
H	-0.095264000	-5.139902000	-0.324234000
H	-2.784185000	-5.141753000	-0.425967000
H	-1.398506000	-1.164968000	-0.017210000
H	1.765041000	-3.170221000	-0.077806000
H	-2.784269000	5.141701000	-0.425904000
H	-0.095340000	5.139869000	-0.324547000
H	-1.398504000	1.164960000	-0.016919000
H	1.765037000	3.170181000	-0.078099000
H	-2.562997000	-0.000025000	-0.891248000
H	-4.655068000	-3.261101000	-0.191555000
H	-4.655021000	3.261104000	-0.191642000
H	-6.842499000	-2.495096000	0.418031000
H	-8.916649000	-1.231551000	0.901373000
H	-8.916621000	1.231603000	0.901405000
H	-6.842453000	2.495118000	0.418093000

S_2-BCBPb

Frequencies:

18.3912	26.8249	46.0871
63.7238	72.9847	74.3433
104.0858	109.1869	121.5148
123.7148	128.1838	149.6091
178.5551	185.0292	191.5282
205.7112	209.8655	232.7652
256.8066	262.4064	264.0861
276.4929	317.5441	334.7190
336.9360	340.2593	378.1943
380.6352	396.0617	411.3731
419.6487	448.8863	478.0451
485.7987	493.8105	501.2032
517.1725	531.2005	559.3710
572.2789	577.4835	606.9768
623.6093	635.8361	654.0973
665.1149	667.4048	674.8220
687.0762	698.9042	700.4451
702.1350	706.8512	714.5540
723.0421	725.9542	733.2390
756.8222	760.7866	771.8515
773.9368	783.7127	790.3629
803.3950	805.1092	810.6104
815.2774	832.9369	844.5402
846.6884	850.7417	858.6075
873.6720	880.4225	891.0715
894.6158	901.9974	912.0115
915.8274	943.2843	945.8248
952.9767	966.0844	982.6412
999.2556	1002.5106	1021.0530
1031.6790	1041.7292	1050.5099
1053.6945	1074.7828	1080.5189
1102.0282	1130.1670	1155.2523
1157.7702	1177.9310	1184.6693
1199.5060	1213.8551	1236.2074
1260.5029	1269.3061	1280.0159
1302.9439	1307.9225	1316.1129
1328.6992	1349.4548	1353.1183
1367.1316	1397.1911	1409.5830
1422.7496	1430.0096	1433.0747
1441.4033	1454.6425	1462.7934
1465.3858	1482.0187	1504.2914
1518.2460	1536.6103	1541.5150
1553.6184	1565.4468	1580.7357
1589.7259	1605.2107	1624.1099
1637.8294	1644.8712	1655.7874
1657.6721	1679.5641	1683.1184
3187.0504	3197.3627	3201.9800
3206.5270	3214.0991	3226.9385
3233.6617	3235.0217	3248.4088
3263.5199	3269.4691	3280.7112
3332.4521	3538.5221	3642.8156

Coordinates:

C	-0.785949000	4.229629000	0.428162000
C	-2.153005000	4.265128000	0.465306000
C	-2.644920000	2.940430000	0.255881000
N	-1.532206000	2.136100000	0.105273000
C	-0.377515000	2.879762000	0.190736000
C	2.772157000	-0.724101000	0.015804000
C	2.727835000	0.665638000	-0.012534000
C	1.360814000	1.074644000	0.070114000
N	0.628761000	-0.090090000	0.133138000
C	1.437504000	-1.204419000	0.113721000
C	0.919932000	2.398057000	0.116769000
C	-2.119958000	-4.281398000	0.255238000
C	-0.768872000	-4.313634000	0.265094000
C	-0.338455000	-2.923192000	0.247450000
N	-1.404993000	-2.079161000	0.247304000
C	-2.499757000	-2.872404000	0.242192000
C	0.993421000	-2.521433000	0.183794000
C	-5.722758000	0.678426000	-0.130360000
C	-5.699926000	-0.730557000	-0.135972000
C	-4.311059000	-1.155439000	0.136433000
C	-3.552955000	0.013828000	0.321722000
C	-4.349280000	1.152337000	0.147864000
C	-3.958636000	2.493197000	0.214462000
C	-3.843206000	-2.458460000	0.169108000
C	4.032231000	-1.429401000	-0.049186000
C	5.268451000	-0.687340000	-0.143159000
C	5.224820000	0.766339000	-0.172692000
C	3.945770000	1.440199000	-0.108056000
C	-6.898368000	1.367992000	-0.381085000
C	-8.063079000	0.636633000	-0.620657000
C	-8.041270000	-0.756467000	-0.624814000
C	-6.854765000	-1.453300000	-0.389475000
N	6.248454000	1.580532000	-0.253188000
S	5.656495000	3.104781000	-0.248832000
N	4.058134000	2.751225000	-0.143132000
S	5.830933000	-2.998421000	-0.125255000
N	6.337387000	-1.444595000	-0.192384000
H	-0.099571000	5.049249000	0.575901000
H	-2.783523000	5.120856000	0.652962000
H	-1.594268000	1.243836000	-0.363016000
H	-0.369442000	-0.230849000	0.264630000
H	1.707182000	3.140730000	0.128369000
H	-2.812585000	-5.110627000	0.253546000
H	-0.111596000	-5.170692000	0.268054000
H	1.762490000	-3.283737000	0.170321000
H	-2.537410000	-0.026676000	0.668239000
H	-4.723580000	3.262180000	0.216662000
H	-4.571902000	-3.259320000	0.082136000
H	-6.923334000	2.452141000	-0.393329000
H	-8.994083000	1.157655000	-0.809258000
H	-8.955773000	-1.304843000	-0.816684000
H	-6.846019000	-2.537508000	-0.406156000
N	4.215798000	-2.732339000	-0.031031000

S_2-BCBPc

Frequencies:

21.1423	27.3818	53.5990
63.9949	68.5026	73.0480
106.0471	108.9451	119.2457
126.5085	129.9392	134.6869
160.2208	184.6533	196.5052
197.1113	213.3922	230.7751
258.9140	259.1064	263.6149
274.5438	317.4002	331.9051
333.3001	335.1302	370.3163
381.2109	402.5863	409.5144
411.8684	451.8831	465.6782
485.8173	496.4478	504.3806
510.9379	514.9577	531.3940
559.1434	575.0249	589.5074
614.1275	657.5513	671.7905
678.0138	687.4621	695.3941
700.9960	702.8225	703.5543
724.6794	727.9456	732.8486
739.7966	760.5539	768.5364
773.9163	777.7595	779.4715
786.5994	803.3512	806.2611
809.2309	817.3760	818.5639
828.2134	842.6941	851.4723
851.8624	857.8232	888.5202
899.2929	905.9644	914.2758
930.6248	931.5560	933.4650
952.3540	952.4368	960.4050
974.5866	984.0932	1001.9169
1009.5687	1011.3338	1021.7389
1052.1971	1053.6274	1054.8655
1072.8309	1076.1129	1088.8093
1140.0535	1157.9741	1166.0216
1173.3409	1179.1344	1179.9479
1214.8420	1224.7340	1249.7015
1254.0357	1255.7396	1262.7384
1296.5602	1324.9380	1327.8576
1342.2984	1346.7349	1352.8926
1364.1488	1379.1623	1390.9533
1402.8917	1415.7296	1418.7490
1429.2512	1441.2676	1460.2198
1472.7047	1488.7151	1514.1778
1520.4394	1523.9280	1531.7893
1566.7100	1594.0005	1596.4183
1614.5198	1633.2833	1636.4997
1649.2304	1668.4275	1672.6681
1689.6972	1692.8218	1708.3324
3078.9627	3099.7163	3192.5231
3192.7124	3203.0642	3208.9402
3218.6444	3222.3541	3222.4571
3228.6926	3248.1638	3248.1708
3269.7880	3269.8061	3527.1289

Coordinates:

C	-0.760870000	4.323580000	-0.000141000
C	-2.109207000	4.318746000	-0.000329000
C	-2.513070000	2.907087000	-0.000223000
N	-1.467954000	2.097283000	-0.000022000
C	-0.356652000	2.920154000	0.000013000
C	2.723667000	-0.702595000	-0.000101000
C	2.723668000	0.702596000	0.000061000
C	1.381340000	1.128828000	0.000130000
N	0.607318000	0.000002000	0.000009000
C	1.381338000	-1.128825000	-0.000151000
C	0.933503000	2.477558000	0.000262000
C	-2.109209000	-4.318746000	0.000392000
C	-0.760872000	-4.323578000	0.000344000
C	-0.356657000	-2.920150000	-0.000056000
N	-1.467958000	-2.097283000	-0.000134000
C	-2.513074000	-2.907088000	0.000179000
C	0.933498000	-2.477555000	-0.000398000
C	-5.664546000	0.704279000	-0.000101000
C	-5.664547000	-0.704279000	0.000069000
C	-4.291293000	-1.194296000	0.000095000
C	-3.383625000	-0.000002000	-0.000059000
C	-4.291291000	1.194295000	-0.000157000
C	-3.888887000	2.484801000	-0.000272000
C	-3.888890000	-2.484803000	0.000246000
C	3.968217000	-1.441353000	-0.000169000
C	5.226990000	-0.728085000	-0.000034000
C	5.226992000	0.728082000	0.000129000
C	3.968220000	1.441352000	0.000161000
C	-6.871735000	1.411376000	-0.000143000
C	-8.059651000	0.702592000	-0.000031000
C	-8.059652000	-0.702588000	0.000130000
C	-6.871737000	-1.411374000	0.000184000
N	6.278517000	1.509435000	0.000308000
S	5.735400000	3.052948000	0.000224000
N	4.122325000	2.747656000	0.000349000
N	4.122321000	-2.747657000	-0.000312000
S	5.735395000	-3.052952000	-0.000332000
N	6.278515000	-1.509440000	-0.000060000
H	-0.086182000	5.167088000	-0.000115000
H	-2.785017000	5.161684000	-0.000479000
H	-0.412497000	0.000004000	0.000013000
H	1.727186000	3.215829000	0.000361000
H	-2.785018000	-5.161685000	0.000613000
H	-0.086183000	-5.167085000	0.000481000
H	1.727181000	-3.215826000	-0.000388000
H	-2.711163000	0.000090000	0.862775000
H	-2.711200000	-0.000095000	-0.862919000
H	-4.633057000	3.275208000	-0.000374000
H	-4.633061000	-3.275208000	0.000426000
H	-6.879448000	2.495260000	-0.000266000
H	-9.004104000	1.233538000	-0.000066000
H	-9.004106000	-1.233532000	0.000216000
H	-6.879452000	-2.495257000	0.000311000

S_2-BCBPd

Frequencies:

19.9362	26.8781	51.5987
61.9097	68.2416	74.0702
101.9456	107.3548	119.6602
124.7762	130.9908	138.0688
164.6804	183.2957	187.3053
203.1483	211.2177	232.7397
256.0061	262.3142	265.0191
277.4515	318.7001	331.7667
336.3838	337.0818	372.5057
376.9161	405.0243	413.1571
414.3269	458.4697	467.6132
486.4113	495.9488	504.4651
513.5155	520.8138	530.8156
559.3458	581.3796	588.8934
614.4234	656.2601	668.6157
670.6692	681.5438	691.1621
692.6330	695.8602	699.8532
723.2652	730.1454	731.0009
746.2223	753.0963	759.4309
763.1449	772.3745	776.3900
783.0797	786.7301	802.4418
806.8016	813.4725	815.7260
843.7614	844.3274	851.8781
857.8240	858.1316	887.4831
893.0734	904.7469	911.5263
922.2905	936.4927	939.7034
951.9007	959.8392	961.3622
983.5699	1003.3787	1011.1158
1011.6746	1021.6895	1025.2303
1042.5127	1045.8508	1054.5135
1067.4498	1073.8436	1081.7079
1140.2149	1167.3341	1176.9816
1182.7927	1193.8108	1196.3784
1213.4950	1228.0304	1244.0326
1253.7036	1263.6711	1276.4331
1301.8659	1309.9677	1327.3298
1335.1518	1345.8804	1357.0921
1365.7509	1384.7358	1396.2580
1416.9612	1420.7725	1434.5123
1440.3358	1449.8068	1459.1343
1464.9469	1478.5123	1507.2148
1518.5795	1520.4423	1533.6320
1557.6786	1571.6374	1593.7035
1597.6900	1618.7965	1634.9854
1638.1571	1655.2783	1671.9796
1675.2300	1695.0088	1708.7451
3054.7075	3080.5717	3195.5087
3197.3135	3203.4148	3209.0233
3209.2674	3215.4725	3218.9154
3229.0799	3247.4083	3265.2645
3269.5350	3281.1836	3503.2591

Coordinates:

C	-0.745691000	4.309286000	0.000004000
C	-2.124850000	4.276926000	0.000000000
C	-2.530172000	2.919105000	0.000028000
N	-1.402200000	2.161404000	0.000034000
C	-0.287886000	2.972015000	0.000018000
C	2.733709000	-0.652824000	-0.000004000
C	2.797853000	0.723938000	-0.000001000
C	1.430286000	1.180610000	0.000008000
N	0.570403000	0.110997000	0.000026000
C	1.318074000	-0.991919000	0.000012000
C	1.028691000	2.508074000	0.000005000
C	-2.098666000	-4.323740000	0.000033000
C	-0.752143000	-4.262464000	0.000038000
C	-0.409417000	-2.835529000	-0.000005000
N	-1.559034000	-2.074140000	0.000001000
C	-2.561913000	-2.927665000	0.000000000
C	0.866371000	-2.353140000	0.000014000
C	-5.677426000	0.698987000	-0.000001000
C	-5.696570000	-0.709905000	-0.000015000
C	-4.333188000	-1.223490000	-0.000015000
C	-3.407236000	-0.037974000	-0.000009000
C	-4.299589000	1.170247000	0.000006000
C	-3.886107000	2.457654000	0.000027000
C	-3.943400000	-2.518623000	-0.000012000
C	3.942353000	-1.446933000	-0.000009000
C	5.234921000	-0.790929000	-0.000010000
C	5.296429000	0.661690000	-0.000008000
C	4.063420000	1.417834000	-0.000004000
C	-6.875255000	1.423091000	-0.000004000
C	-8.072560000	0.731391000	-0.000019000
C	-8.091863000	-0.674053000	-0.000032000
C	-6.914389000	-1.399223000	-0.000031000
N	6.375249000	1.406986000	0.000002000
S	5.883392000	2.967241000	-0.000024000
N	4.261646000	2.720034000	0.000007000
N	4.042424000	-2.760512000	-0.000003000
S	5.639352000	-3.134659000	-0.000012000
N	6.251519000	-1.618167000	-0.000005000
H	-0.103069000	5.176286000	-0.000003000
H	-2.805829000	5.113642000	-0.000015000
H	-1.295809000	1.147522000	0.000050000
H	1.809644000	3.261048000	0.000002000
H	-2.732546000	-5.198738000	0.000050000
H	-0.039239000	-5.074242000	0.000063000
H	1.662134000	-3.090801000	0.000037000
H	-2.741970000	-0.076033000	0.869958000
H	-2.741979000	-0.076017000	-0.869984000
H	-4.626384000	3.251067000	0.000036000
H	-4.692803000	-3.303877000	-0.000014000
H	-6.869022000	2.507039000	0.000003000
H	-9.009395000	1.275547000	-0.000023000
H	-9.043600000	-1.191770000	-0.000046000
H	-6.936798000	-2.482833000	-0.000046000

S_2-BPa

Frequencies:

26.8413	30.7441	60.1445
69.6830	79.5589	102.2140
108.4484	125.4408	137.1522
138.5823	139.3079	184.2149
184.2779	213.6381	218.5753
235.0855	253.6958	264.8990
315.0854	315.9971	319.4988
324.6245	348.9172	366.6654
385.2836	387.3571	410.7207
411.9122	438.5158	465.1616
489.6968	498.0078	519.2721
532.2028	582.1607	630.7977
652.5115	661.7646	679.6733
679.6919	693.0163	700.4246
701.8146	708.8961	712.7683
712.7830	723.1596	725.5288
737.5078	744.6450	752.2481
764.9599	776.6341	777.5954
792.6146	797.1569	804.0651
806.9413	810.6392	820.3940
832.0185	845.5173	854.4776
858.3559	873.4386	882.7124
902.3502	906.5052	915.9759
946.3529	948.1893	950.3083
966.2493	975.4484	990.5208
1007.3509	1014.9764	1020.2866
1037.2234	1041.0962	1066.0694
1073.8024	1078.2927	1124.6153
1158.5865	1179.2170	1184.5849
1206.5568	1208.6121	1257.4001
1259.4308	1261.0275	1272.5302
1318.1486	1328.6651	1359.6832
1361.2228	1390.1146	1406.7955
1417.7232	1428.6765	1441.9724
1452.9244	1456.2801	1465.7623
1481.5496	1524.5945	1540.6464
1550.4389	1550.5130	1565.7656
1567.5817	1577.8509	1608.4800
1620.7142	1628.0893	1649.3760
1654.3372	1661.6295	1677.8894
3218.3597	3218.4916	3231.8726
3232.1726	3249.0003	3249.0130
3261.9379	3270.2245	3270.2425
3279.4441	3556.4493	3613.0197

Coordinates:

C	-1.605379000	4.241010000	0.000147000
C	-2.954008000	4.238230000	0.000106000
C	-3.354466000	2.834176000	0.000059000
N	-2.278419000	2.014351000	0.000283000
C	-1.199174000	2.839192000	0.000192000
C	1.959604000	-0.694280000	0.000126000
C	1.959604000	0.694280000	0.000102000
C	0.599830000	1.132651000	0.000192000
N	-0.166808000	0.000001000	0.000274000
C	0.599830000	-1.132651000	0.000230000
C	0.133559000	2.442861000	0.000148000
C	-2.954009000	-4.238230000	0.000026000
C	-1.605380000	-4.241009000	0.000196000
C	-1.199175000	-2.839191000	0.000161000
N	-2.278421000	-2.014350000	0.000064000
C	-3.354468000	-2.834177000	-0.000213000
C	0.133558000	-2.442860000	0.000237000
C	-6.518877000	0.681576000	-0.000189000
C	-6.518878000	-0.681575000	-0.000310000
C	-5.152662000	-1.122326000	-0.000253000
N	-4.375271000	0.000000000	-0.000103000
C	-5.152661000	1.122326000	-0.000049000
C	-4.691239000	2.428853000	0.000089000
C	-4.691241000	-2.428854000	-0.000349000
H	-1.182600000	0.000000000	0.000324000
C	3.200742000	-1.435556000	0.000078000
C	4.461468000	-0.727343000	-0.000032000
C	4.461469000	0.727342000	-0.000068000
C	3.200743000	1.435556000	0.000018000
N	3.347799000	-2.743335000	0.000041000
S	4.957639000	-3.053788000	-0.000030000
N	5.509865000	-1.514356000	-0.000143000
N	5.509866000	1.514355000	-0.000195000
S	4.957640000	3.053787000	-0.000293000
N	3.347801000	2.743335000	-0.000048000
H	-3.360673000	0.000000000	-0.000022000
H	-0.928940000	5.083137000	0.000140000
H	-3.632302000	5.078984000	0.000068000
H	0.893387000	3.214517000	0.000066000
H	-3.632302000	-5.078984000	0.000005000
H	-0.928940000	-5.083135000	0.000337000
H	0.893386000	-3.214516000	0.000315000
H	-7.369325000	1.346050000	-0.000211000
H	-7.369326000	-1.346048000	-0.000448000
H	-5.447223000	3.205301000	-0.000028000
H	-5.447224000	-3.205301000	-0.000447000

S_2-BPb

Frequencies:

26.1119	30.7822	61.6312
71.6496	80.6035	105.4173
109.0106	117.9639	122.7686
137.1429	141.9832	170.6106
208.8530	216.4802	216.5772
226.1675	252.4416	263.9826
303.4113	317.3847	331.7149
334.0489	345.3540	365.5176
383.4523	389.3172	407.9326
413.5725	451.8822	457.9110
491.6699	496.4615	522.3103
531.5224	583.1240	608.2106
653.2990	660.2111	673.0746
678.0390	687.8095	689.2262
695.7534	706.8854	714.8470
718.8260	732.9444	738.0093
743.3500	743.4102	746.5982
756.7534	757.8558	787.6600
795.5523	797.6616	802.4613
805.2331	810.1951	813.8794
845.3044	846.7415	854.5035
858.4948	873.3763	880.7545
904.5405	905.9188	907.0306
946.1105	949.2421	949.8673
963.1234	981.5383	1004.1555
1010.3530	1017.2820	1024.7491
1032.0090	1040.6213	1051.2657
1075.5977	1084.2019	1139.6351
1175.8814	1188.8310	1193.5286
1205.4531	1215.7957	1254.8412
1256.0429	1257.2248	1272.8054
1301.3356	1339.5703	1356.2268
1382.6181	1388.6289	1405.0752
1414.0950	1430.1552	1446.9815
1452.6841	1456.8737	1465.5701
1473.4303	1505.9071	1522.1871
1557.3410	1561.6898	1569.8446
1570.8556	1586.2850	1588.6307
1615.8997	1619.3289	1636.7287
1655.3243	1662.4737	1679.1278
3218.7411	3218.7808	3228.8089
3228.8788	3248.1065	3262.6915
3262.7236	3269.3727	3280.0430
3280.0522	3575.1701	3629.1604

Coordinates:

C	-1.598022000	4.238320000	-0.000094000
C	-2.962074000	4.238355000	-0.000159000
C	-3.401603000	2.873235000	-0.000115000
N	-2.281489000	2.095453000	-0.000017000
C	-1.156921000	2.873794000	0.000000000
C	1.962988000	-0.686651000	-0.000028000
C	1.962989000	0.686626000	0.000045000
C	0.566687000	1.088897000	0.000031000
N	-0.240435000	-0.000003000	0.000033000
C	0.566686000	-1.088908000	0.000005000
C	0.152318000	2.423027000	0.000090000
C	-2.962088000	-4.238345000	0.000155000
C	-1.598028000	-4.238313000	0.000136000
C	-1.156931000	-2.873803000	0.000052000
N	-2.281495000	-2.095461000	0.000026000
C	-3.401606000	-2.873241000	0.000086000
C	0.152319000	-2.423034000	0.000013000
C	-6.520172000	0.674166000	-0.000003000
C	-6.520185000	-0.674162000	0.000046000
C	-5.116505000	-1.078193000	0.000033000
N	-4.295232000	0.000005000	-0.000032000
C	-5.116493000	1.078192000	-0.000083000
C	-4.711940000	2.411314000	-0.000169000
C	-4.711947000	-2.411308000	0.000085000
C	3.200177000	-1.432666000	-0.000088000
C	4.463991000	-0.726807000	-0.000069000
C	4.463988000	0.726814000	0.000019000
C	3.200166000	1.432659000	0.000074000
N	3.348000000	-2.741655000	-0.000160000
S	4.957510000	-3.053363000	-0.000160000
N	5.511965000	-1.514848000	-0.000150000
N	5.511956000	1.514863000	0.000023000
S	4.957488000	3.053373000	0.000213000
N	3.347978000	2.741650000	0.000095000
H	-0.932292000	5.087756000	-0.000106000
H	-3.626609000	5.088680000	-0.000236000
H	-2.287208000	1.081435000	0.000031000
H	0.928097000	3.178972000	0.000102000
H	-3.626624000	-5.088670000	0.000213000
H	-0.932303000	-5.087755000	0.000176000
H	-2.287213000	-1.081441000	-0.000028000
H	0.928096000	-3.178985000	-0.000009000
H	-7.362536000	1.350471000	-0.000007000
H	-7.362564000	-1.350447000	0.000097000
H	-5.487153000	3.168507000	-0.000327000
H	-5.487152000	-3.168512000	0.000136000

S_2-BPc

Frequencies:

25.8386	28.2230	59.0597
78.4395	78.5455	98.2049
107.7890	112.2739	120.5999
148.6834	172.1158	173.6782
187.1103	208.6435	211.2669
230.0127	254.2390	264.8131
317.0048	317.9095	329.8448
334.5885	345.6789	365.8771
381.4080	392.0926	413.3958
415.8229	444.1674	462.1124
490.2234	500.7308	521.8973
530.8029	582.8538	592.0852
652.6753	654.9669	673.2029
683.4903	685.3455	688.2073
694.0506	700.0324	712.2805
716.4333	728.3603	735.6893
738.5371	744.9704	747.4798
765.3408	774.9735	783.7008
796.9161	801.5169	802.6808
807.1205	811.9482	815.1106
843.8612	850.2035	852.4107
857.9976	858.7825	876.9775
901.1601	905.4320	930.1510
943.3315	945.6410	949.8783
965.9679	981.4315	990.7397
1017.1619	1028.7298	1032.3727
1036.7613	1048.6606	1057.9360
1075.2040	1084.1812	1145.3501
1178.5354	1189.5491	1193.2638
1206.0152	1222.2070	1243.1752
1262.1566	1268.8639	1293.4140
1324.8989	1343.6175	1358.5294
1375.0819	1400.4951	1415.6413
1420.1326	1421.8110	1445.6719
1447.6047	1463.2491	1470.7236
1478.8638	1494.3180	1523.9357
1533.6016	1557.2509	1571.9735
1576.7525	1585.7690	1590.8844
1613.6112	1618.4007	1633.2721
1658.5001	1664.3746	1685.2331
3212.6931	3220.4332	3225.3247
3234.0738	3250.3805	3264.5228
3264.6111	3271.4127	3281.9405
3282.1310	3382.4539	3442.2321

Coordinates:

C	-1.635969000	4.147488000	-0.000057000
C	-2.983851000	4.227464000	-0.000090000
C	-3.459801000	2.849353000	-0.000127000
N	-2.433550000	1.972201000	-0.000092000
C	-1.306052000	2.722227000	-0.000061000
C	2.020791000	-0.718391000	-0.000011000
C	1.926471000	0.652716000	-0.000022000
C	0.500972000	0.963097000	-0.000054000
N	-0.219026000	-0.179113000	-0.000035000
C	0.656649000	-1.210324000	-0.000056000
C	0.010975000	2.273496000	-0.000044000
C	-2.903895000	-4.253124000	-0.000228000
C	-1.541743000	-4.324814000	-0.000293000
C	-1.035839000	-2.987084000	-0.000121000
N	-2.122777000	-2.146256000	0.000032000
C	-3.282002000	-2.868038000	-0.000007000
C	0.280860000	-2.558035000	-0.000098000
C	-6.560034000	0.617883000	0.000366000
C	-6.469242000	-0.742038000	0.000436000
C	-5.077205000	-1.101665000	0.000138000
N	-4.372729000	0.068792000	-0.000115000
C	-5.227451000	1.142732000	0.000012000
C	-4.806268000	2.461960000	-0.000207000
C	-4.593834000	-2.403472000	0.000122000
C	3.300061000	-1.385727000	0.000030000
C	4.516934000	-0.603688000	0.000056000
C	4.423437000	0.846945000	0.000043000
C	3.117149000	1.473486000	0.000005000
N	3.525695000	-2.683536000	0.000043000
S	5.151947000	-2.895841000	0.000086000
N	5.611451000	-1.325833000	0.000093000
N	5.420855000	1.697926000	0.000065000
S	4.772882000	3.199074000	0.000041000
N	3.186025000	2.789121000	0.000005000
H	-0.912623000	4.949962000	-0.000037000
H	-3.609486000	5.107963000	-0.000104000
H	0.770714000	3.047602000	-0.000028000
H	-3.611062000	-5.068462000	-0.000313000
H	-0.922655000	-5.208698000	-0.000439000
H	-1.954526000	-1.138165000	0.000175000
H	1.061856000	-3.308189000	-0.000099000
H	-7.453565000	1.223052000	0.000530000
H	-7.274714000	-1.460508000	0.000673000
H	-3.366902000	0.260813000	-0.000400000
H	-5.569202000	3.230919000	-0.000434000
H	-5.353386000	-3.175807000	0.000081000

S_2-BPd

Frequencies:

25.9576	28.5122	59.7497
79.5149	80.5176	97.1750
108.0809	113.0196	124.1977
149.1911	173.5129	175.8680
195.0413	201.8868	212.1685
228.2814	255.7301	266.7788
315.1287	316.4243	324.1969
338.8323	346.8536	364.7464
384.4692	389.2980	411.6582
414.2800	445.1364	459.6273
487.9533	496.5050	516.6495
531.4804	577.3331	627.8880
651.9590	662.0160	678.7112
682.1434	688.7869	693.5189
698.2686	707.3304	710.9720
719.8229	722.2326	725.5078
735.3153	750.6022	751.9095
763.8216	779.2742	783.3928
792.9168	802.9878	803.2429
804.7268	820.5917	823.3955
841.4386	845.1988	851.9128
858.6272	872.6842	888.8615
897.8602	907.4722	914.4062
942.7799	946.7526	948.1153
967.7243	975.5307	992.5260
1002.1122	1022.0165	1028.9182
1039.0918	1053.6437	1061.8476
1073.0025	1077.9884	1125.6100
1160.5294	1177.5483	1189.9903
1207.2162	1214.6916	1242.2723
1258.7386	1290.4608	1299.0099
1326.1822	1338.2468	1353.2412
1374.6561	1390.2223	1411.1856
1423.9056	1424.9344	1444.3798
1445.2776	1456.6132	1473.5627
1485.5003	1499.7402	1532.5398
1545.9298	1551.4361	1568.9182
1577.3318	1587.1594	1601.7396
1615.3101	1625.5179	1639.9013
1654.3380	1663.0821	1686.9271
3194.7724	3226.2357	3231.2572
3238.8872	3250.0806	3250.5358
3264.1233	3271.2761	3271.6806
3281.5327	3358.2450	3403.2636

Coordinates:

C	-1.542396000	4.355405000	-0.000384000
C	-2.889832000	4.277157000	-0.000499000
C	-3.221689000	2.850868000	-0.000312000
N	-2.103056000	2.099597000	-0.000156000
C	-1.067876000	2.976706000	-0.000161000
C	1.906917000	-0.653712000	-0.000095000
C	2.005281000	0.731333000	0.000009000
C	0.683365000	1.261536000	-0.000048000
N	-0.168112000	0.184110000	-0.000119000
C	0.517469000	-1.001878000	-0.000228000
C	0.270065000	2.589624000	-0.000009000
C	-3.047830000	-4.192293000	-0.000054000
C	-1.686601000	-4.129368000	-0.000271000
C	-1.298245000	-2.745160000	-0.000161000
N	-2.455631000	-2.015840000	0.000132000
C	-3.544630000	-2.848841000	0.000198000
C	0.015481000	-2.300539000	-0.000373000
C	-6.472674000	0.802407000	0.000365000
C	-6.585257000	-0.542553000	0.000571000
C	-5.217809000	-1.052651000	0.000314000
N	-4.317358000	-0.045856000	-0.000110000
C	-5.037963000	1.097546000	-0.000034000
C	-4.545551000	2.399680000	-0.000279000
C	-4.858621000	-2.404160000	0.000473000
C	3.096667000	-1.476659000	-0.000095000
C	4.402765000	-0.853588000	0.000013000
C	4.502187000	0.597627000	0.000132000
C	3.292544000	1.387439000	0.000127000
N	3.158992000	-2.791572000	-0.000207000
S	4.745452000	-3.207734000	-0.000160000
N	5.396428000	-1.708195000	-0.000021000
N	5.600626000	1.313423000	0.000195000
S	5.150807000	2.886017000	0.000493000
N	3.524151000	2.682780000	0.000189000
H	-0.914023000	5.233836000	-0.000442000
H	-3.610538000	5.082127000	-0.000656000
H	-1.174485000	0.379877000	-0.000170000
H	1.044667000	3.345908000	-0.000197000
H	-3.671874000	-5.072702000	-0.000072000
H	-0.982826000	-4.947679000	-0.000510000
H	-2.627106000	-1.005818000	0.000366000
H	0.769561000	-3.077287000	0.000049000
H	-7.259142000	1.543273000	0.000477000
H	-7.480887000	-1.146356000	0.000893000
H	-5.299556000	3.180948000	-0.000416000
H	-5.640824000	-3.153344000	0.000823000

S_2-BSPa

Frequencies:

28.2919	29.9715	34.2864
73.0640	74.5275	81.3849
105.7961	119.8574	121.9672
130.6712	138.2975	159.3882
183.2173	212.8917	218.8808
239.0983	250.2839	258.6040
268.2689	279.1298	316.6773
319.7613	322.5181	364.8900
380.4492	386.1101	409.4094
412.1283	429.9687	451.4339
483.5377	488.5331	505.2464
517.9609	519.3303	532.5648
581.0835	581.8119	622.2025
634.5052	653.9071	668.8059
682.1406	692.2128	695.4034
697.9047	702.8611	708.0703
718.1147	725.7612	738.1290
754.1032	763.8731	764.8069
774.8637	782.1841	784.0251
793.9077	803.1974	811.8053
819.6843	830.4499	847.3602
857.4126	859.9920	886.3757
900.7814	918.0153	925.2744
927.3286	947.9474	948.2230
963.8208	975.6338	990.2221
1009.1764	1017.3824	1031.4539
1041.6076	1069.8687	1071.3576
1074.2918	1105.1607	1156.4425
1162.1522	1181.2570	1204.0539
1209.3680	1241.1503	1251.7792
1266.6007	1285.0542	1306.3729
1327.3575	1335.6894	1355.5737
1360.7730	1398.8177	1413.6858
1420.7449	1433.8911	1445.5753
1449.5596	1468.7534	1488.6670
1510.4102	1518.3589	1527.4225
1558.8305	1560.7314	1571.7931
1588.2940	1602.3496	1608.1384
1624.9303	1640.4383	1647.0038
1657.5545	3192.8805	3193.1067
3208.6682	3223.4480	3223.6919
3225.8403	3250.4814	3250.4832
3270.9932	3271.0144	3590.9088

Coordinates:

C	-1.173497000	-4.435218000	0.000151000
C	-2.525367000	-4.494821000	0.000265000
C	-2.993864000	-3.108179000	-0.000169000
N	-1.960630000	-2.263123000	-0.000665000
C	-0.828414000	-3.012733000	-0.000382000
C	2.208103000	0.697212000	-0.000569000
C	2.208101000	-0.697211000	-0.000566000
C	0.855337000	-1.138434000	-0.000496000
N	0.085591000	0.000003000	-0.000255000
C	0.855340000	1.138439000	-0.000502000
C	0.450730000	-2.489691000	-0.000356000
C	-2.525362000	4.494825000	0.000243000
C	-1.173491000	4.435223000	0.000126000
C	-0.828408000	3.012739000	-0.000397000
N	-1.960623000	2.263128000	-0.000671000
C	-2.993858000	3.108182000	-0.000180000
C	0.450736000	2.489696000	-0.000370000
C	-5.945622000	-0.682900000	0.000302000
C	-5.945620000	0.682905000	0.000300000
C	-4.652216000	1.307027000	0.000305000
Se	-3.304107000	-0.000002000	0.000249000
C	-4.652220000	-1.307025000	0.000308000
C	-4.338439000	-2.648298000	0.000068000
C	-4.338433000	2.648300000	0.000059000
H	-0.928960000	0.000004000	-0.001490000
C	3.453077000	1.437363000	-0.000183000
C	4.712457000	0.727066000	0.000236000
C	4.712454000	-0.727073000	0.000235000
C	3.453073000	-1.437366000	-0.000182000
N	3.606616000	2.744521000	-0.000231000
S	5.217134000	3.051388000	0.000300000
N	5.763366000	1.510201000	0.000518000
N	5.763362000	-1.510211000	0.000523000
S	5.217124000	-3.051395000	0.000240000
N	3.606607000	-2.744524000	-0.000219000
H	-0.466467000	-5.251910000	0.000232000
H	-3.154717000	-5.372803000	0.000581000
H	1.273956000	-3.194338000	-0.000237000
H	-3.154713000	5.372807000	0.000553000
H	-0.466463000	5.251916000	0.000200000
H	1.273963000	3.194343000	-0.000256000
H	-6.853679000	-1.274706000	0.000261000
H	-6.853675000	1.274715000	0.000257000
H	-5.137677000	-3.383373000	0.000168000
H	-5.137671000	3.383375000	0.000156000

S_2-BSPb

Frequencies:

16.4850	26.3118	27.8943
60.5075	77.0620	81.2842
103.1617	108.2829	109.0406
131.6161	155.0900	161.2384
179.3539	211.1640	219.7954
238.3601	251.7359	259.9480
265.8123	284.7879	318.5374
323.2327	331.7436	364.9834
381.3596	390.7840	413.5800
414.8843	435.5475	453.4505
487.0228	490.8445	506.1518
515.5893	524.1205	531.2192
581.2099	586.5236	615.3190
629.3751	634.5028	653.3157
677.9259	687.8766	692.1347
692.8845	698.0005	704.1477
725.3314	735.8147	746.4311
747.9805	757.0168	763.1530
774.1904	784.6305	786.5305
798.0111	803.0407	809.6654
813.4521	845.9854	848.6361
854.8556	859.5321	879.0271
893.6963	907.1953	925.0005
934.9310	947.3088	955.3769
963.2398	972.4565	1001.3946
1023.5889	1027.8008	1038.2825
1043.1369	1061.1075	1071.0783
1090.1801	1111.8621	1159.7532
1164.5929	1199.7443	1206.9670
1221.7259	1238.3394	1248.4298
1267.2281	1282.4447	1316.7166
1322.2619	1343.2221	1357.0969
1369.6012	1394.6839	1406.5121
1425.6506	1437.6484	1451.2153
1460.5029	1467.5548	1479.1383
1496.0916	1515.1496	1525.6329
1544.9824	1563.9668	1571.8184
1577.0581	1583.5703	1602.1430
1615.2004	1630.9447	1646.2929
1667.7876	3185.5097	3206.2680
3206.7275	3210.4003	3222.4963
3225.5276	3252.8199	3264.0067
3273.0230	3280.8024	3456.6340

Coordinates:

C	-1.227212000	4.303649000	0.000084000
C	-2.574468000	4.447560000	-0.000162000
C	-3.119288000	3.092262000	-0.000180000
N	-2.135584000	2.194621000	0.000061000
C	-0.957243000	2.861545000	0.000191000
C	2.290160000	-0.721237000	0.000201000
C	2.181319000	0.649211000	0.000132000
C	0.751247000	0.949333000	0.000163000
N	0.040869000	-0.199228000	0.000183000
C	0.934199000	-1.231757000	0.000215000
C	0.307382000	2.297336000	0.000302000
C	-2.464527000	-4.492855000	0.000520000
C	-1.091971000	-4.511684000	0.000804000
C	-0.645002000	-3.155815000	0.000395000
N	-1.765587000	-2.375013000	-0.000148000
C	-2.901201000	-3.128298000	-0.000006000
C	0.631055000	-2.601800000	0.000424000
C	-5.991913000	0.594787000	-0.000593000
C	-5.935539000	-0.771797000	-0.000509000
C	-4.614013000	-1.326207000	-0.000223000
Se	-3.323634000	0.057115000	0.000148000
C	-4.734374000	1.285232000	-0.000371000
C	-4.464677000	2.637395000	-0.000387000
C	-4.235307000	-2.653283000	-0.000100000
C	3.577432000	-1.376688000	-0.000089000
C	4.786214000	-0.583536000	-0.000230000
C	4.679033000	0.865166000	-0.000086000
C	3.367412000	1.480812000	0.000116000
N	3.818157000	-2.672135000	-0.000288000
S	5.445996000	-2.868142000	-0.000666000
N	5.888440000	-1.293917000	-0.000574000
N	5.670715000	1.722570000	0.000039000
S	5.013320000	3.218922000	0.000271000
N	3.429967000	2.797125000	0.000349000
H	-0.473958000	5.078291000	0.000328000
H	-3.147424000	5.363096000	-0.000447000
H	1.119852000	3.016177000	0.000418000
H	-3.132102000	-5.340865000	0.000857000
H	-0.444653000	-5.374939000	0.001214000
H	-1.631935000	-1.365764000	-0.000560000
H	1.471855000	-3.285330000	0.000712000
H	-6.923916000	1.147891000	-0.000920000
H	-6.820694000	-1.397728000	-0.000629000
H	-5.280521000	3.352300000	-0.000553000
H	-5.003161000	-3.421658000	-0.000127000

S_2-BTPa

Frequencies:

27.4773	32.1180	55.6397
76.1287	78.2105	82.4066
106.0509	120.5949	122.8467
138.5926	155.0561	163.5818
181.2139	214.7814	218.1261
235.4994	259.2869	261.5714
294.8497	314.9862	321.5272
323.1431	326.9964	364.7800
383.7367	388.2292	408.7857
411.9174	433.0479	453.1184
487.9390	488.9787	519.3972
532.3385	536.5158	580.6785
581.9289	626.6095	653.7734
680.7702	681.5415	692.5285
693.7285	696.4447	702.5099
705.7042	707.2661	719.4870
725.8891	726.6373	749.2586
756.5024	760.7429	776.0502
779.6855	788.6166	788.9957
798.9042	803.3358	813.2361
819.9361	831.9209	847.5057
857.6510	859.9149	881.9446
894.4886	917.9676	922.1424
924.8955	948.1268	948.3884
959.1718	974.4283	983.0500
992.1215	1007.9623	1031.0282
1041.2877	1058.8321	1071.3876
1074.2725	1111.5983	1153.2910
1166.1943	1178.9063	1207.6961
1210.0916	1245.4904	1256.5312
1264.4816	1298.5245	1305.5434
1331.5702	1336.0057	1357.0776
1362.6969	1395.5914	1415.0361
1421.8599	1437.5012	1449.0026
1451.6250	1473.8418	1497.6288
1515.0017	1522.0795	1531.3782
1559.7796	1564.1307	1575.3949
1596.7501	1605.4303	1609.2393
1624.8471	1646.2691	1649.7788
1661.7859	3202.6104	3202.8107
3224.8809	3227.5764	3227.8382
3242.1113	3249.3417	3249.3461
3270.2796	3270.3018	3552.6510

Coordinates:

C	-1.419774000	-4.399770000	0.000140000
C	-2.770951000	-4.438612000	-0.000204000
C	-3.215458000	-3.043790000	-0.000372000
N	-2.169292000	-2.208187000	-0.000222000
C	-1.057468000	-2.981700000	0.000035000
C	1.990342000	0.695505000	-0.000103000
C	1.990342000	-0.695505000	0.000008000
C	0.634924000	-1.135928000	0.000008000
N	-0.138804000	0.000000000	-0.000138000
C	0.634925000	1.135929000	-0.000178000
C	0.232520000	-2.481052000	0.000150000
C	-2.770950000	4.438613000	-0.000017000
C	-1.419774000	4.399771000	-0.000169000
C	-1.057467000	2.981701000	-0.000043000
N	-2.169291000	2.208188000	0.000301000
C	-3.215457000	3.043790000	0.000350000
C	0.232521000	2.481053000	-0.000251000
C	-6.208274000	-0.682334000	-0.000149000
C	-6.208274000	0.682334000	0.000212000
C	-4.894858000	1.251035000	0.000368000
S	-3.687538000	0.000000000	0.000185000
C	-4.894859000	-1.251035000	-0.000316000
C	-4.551890000	-2.589595000	-0.000737000
C	-4.551890000	2.589595000	0.000671000
H	-1.157058000	0.000000000	-0.000193000
C	3.234344000	1.436804000	-0.000128000
C	4.494052000	0.727000000	-0.000065000
C	4.494052000	-0.727000000	0.000050000
C	3.234344000	-1.436804000	0.000098000
N	3.387222000	2.744098000	-0.000174000
S	4.997435000	3.051406000	-0.000213000
N	5.544704000	1.510611000	-0.000065000
N	5.544704000	-1.510612000	0.000173000
S	4.997434000	-3.051407000	0.000266000
N	3.387221000	-2.744099000	0.000259000
H	-0.723023000	-5.225209000	0.000331000
H	-3.416420000	-5.304876000	-0.000265000
H	1.048597000	-3.193488000	0.000382000
H	-3.416420000	5.304876000	-0.000050000
H	-0.723022000	5.225209000	-0.000440000
H	1.048598000	3.193489000	-0.000487000
H	-7.098240000	-1.298562000	-0.000314000
H	-7.098239000	1.298562000	0.000371000
H	-5.348727000	-3.326120000	-0.000712000
H	-5.348726000	3.326120000	0.000486000

S_2-BTPb

Frequencies:

26.2570	28.7810	47.4418
70.0520	81.1632	81.1832
106.3819	114.6864	120.0217
130.7381	157.6133	175.1917
177.6057	214.0947	214.8373
234.8626	257.3910	262.4613
298.9339	317.1882	321.5464
324.6737	331.8786	364.7952
381.2060	391.2364	413.0837
414.2473	438.1766	454.0609
488.6977	493.1513	523.3204
530.4503	532.6221	586.0650
586.8787	621.6208	653.1006
663.5235	677.1313	684.6666
688.1128	691.3877	695.3044
703.5150	708.7515	713.9299
725.9003	743.1922	746.9638
755.3748	756.5947	774.8469
777.3581	788.0893	788.8011
794.4450	802.8154	813.6717
814.5135	845.7684	849.4992
854.6415	859.3207	869.9770
889.6112	903.3540	919.0737
936.0826	947.2893	955.3006
956.1265	962.4248	989.8674
1022.5451	1027.5089	1029.4206
1039.1632	1051.5103	1070.4639
1087.0725	1113.5829	1164.0651
1171.5335	1191.8639	1206.8596
1223.2925	1241.0276	1251.8587
1267.9269	1292.5771	1319.4765
1326.1213	1341.7921	1356.8836
1369.2066	1396.0575	1406.4780
1424.6281	1440.0778	1455.2774
1461.6627	1468.7227	1480.1542
1500.7983	1518.5743	1527.9999
1553.7482	1569.1392	1575.4023
1577.9718	1592.1470	1606.1395
1615.9572	1634.0593	1652.2003
1672.3245	3205.0402	3209.9373
3211.3633	3225.9853	3226.5506
3242.9494	3251.1519	3263.5669
3271.7723	3280.6555	3446.8570

Coordinates:

C	-1.444697000	4.294153000	-0.000173000
C	-2.792046000	4.411650000	-0.000271000
C	-3.306109000	3.041860000	-0.000140000
N	-2.308916000	2.153878000	0.000135000
C	-1.154785000	2.854373000	0.000018000
C	2.073015000	-0.717979000	-0.000051000
C	1.986023000	0.652926000	0.000065000
C	0.559914000	0.973839000	0.000061000
N	-0.169289000	-0.163573000	0.000029000
C	0.706263000	-1.203973000	-0.000013000
C	0.123942000	2.316685000	0.000031000
C	-2.747596000	-4.403358000	0.000158000
C	-1.378957000	-4.428761000	0.000140000
C	-0.922813000	-3.074440000	0.000039000
N	-2.036369000	-2.280104000	0.000012000
C	-3.174892000	-3.034773000	0.000098000
C	0.368458000	-2.564054000	-0.000027000
C	-6.261884000	0.645896000	-0.000149000
C	-6.234178000	-0.721073000	-0.000083000
C	-4.907475000	-1.250520000	0.000088000
S	-3.736607000	0.032989000	0.000173000
C	-4.969148000	1.257338000	-0.000059000
C	-4.647814000	2.601757000	-0.000169000
C	-4.504594000	-2.575027000	0.000124000
C	3.348930000	-1.394060000	-0.000124000
C	4.570368000	-0.619982000	-0.000069000
C	4.485985000	0.830463000	0.000053000
C	3.183966000	1.466201000	0.000119000
N	3.568535000	-2.693212000	-0.000219000
S	5.193207000	-2.915110000	-0.000217000
N	5.661031000	-1.347970000	-0.000146000
N	5.490351000	1.673108000	0.000118000
S	4.855000000	3.179182000	0.000193000
N	3.265491000	2.781375000	0.000209000
H	-0.703798000	5.080617000	-0.000259000
H	-3.386392000	5.313601000	-0.000435000
H	0.930508000	3.042149000	-0.000056000
H	-3.422498000	-5.245580000	0.000247000
H	-0.733260000	-5.293300000	0.000192000
H	-1.910044000	-1.267259000	-0.000134000
H	1.186103000	-3.274925000	-0.000065000
H	-7.166322000	1.240705000	-0.000286000
H	-7.112785000	-1.353128000	-0.000167000
H	-5.452615000	3.328801000	-0.000368000
H	-5.269590000	-3.344005000	-0.000011000

S_2-OBBPa

Frequencies:

21.7758	24.2320	48.3404
54.7347	73.8664	85.8365
103.2196	107.6822	110.5623
116.4308	129.5181	153.0853
177.5933	195.9460	210.7669
216.6232	237.8941	245.2971
259.9102	276.1068	300.2157
303.1987	312.1363	324.9795
332.6696	357.3053	381.7741
396.6756	407.6962	414.2192
438.5349	488.2472	488.8190
495.7302	505.7658	527.5852
531.0216	532.0542	552.6970
581.8100	590.1856	594.7280
610.0305	654.6045	672.0900
677.1858	684.1424	691.5052
692.6814	697.7694	722.6956
724.1602	742.4737	744.3024
747.3898	754.8276	755.0784
772.6175	790.2142	797.4805
805.4126	808.2934	817.6276
836.9263	843.3144	846.0623
848.1255	855.5206	859.8857
872.3365	890.6737	892.2051
900.7615	909.9276	928.5912
942.4786	948.6334	957.8489
978.0933	1003.1460	1011.8316
1017.6412	1021.3316	1032.9652
1038.4655	1054.1306	1085.5876
1121.7123	1154.9175	1187.7168
1194.1775	1198.1776	1208.8842
1217.9061	1247.9323	1262.9152
1278.2532	1281.1949	1291.4017
1312.4369	1336.7226	1353.7469
1393.1326	1396.4850	1402.3939
1417.9177	1427.9204	1442.4489
1450.7146	1453.0505	1468.4137
1471.7582	1483.8453	1494.2392
1524.9258	1547.1025	1555.0151
1571.4448	1583.6499	1590.4005
1606.0343	1618.2905	1632.3956
1647.5164	1658.2860	1668.9565
1703.3267	1771.4539	3186.4536
3193.1299	3204.1473	3229.4375
3231.2521	3235.4836	3256.2914
3266.3681	3266.4686	3283.8180
3283.9482	3635.4988	3673.6078

Coordinates:

C	0.978247000	4.356693000	-0.185924000
C	2.346501000	4.398085000	-0.200253000
C	2.841879000	3.060215000	-0.125105000
N	1.737934000	2.246431000	-0.074098000
C	0.589173000	2.989416000	-0.099398000
C	-2.361610000	-0.715538000	0.025659000
C	-2.416606000	0.654123000	0.030329000
C	-1.038785000	1.117348000	0.002543000
N	-0.183338000	0.052519000	-0.006917000
C	-0.949276000	-1.066964000	-0.004973000
C	-0.699415000	2.464090000	-0.043788000
C	2.674567000	-4.081107000	-0.232505000
C	1.315161000	-4.146684000	-0.212941000
C	0.814426000	-2.806131000	-0.120018000
N	1.901215000	-1.976064000	-0.098247000
C	3.070513000	-2.700456000	-0.153391000
C	-0.505228000	-2.392388000	-0.059132000
C	4.921507000	-0.969579000	-0.014378000
C	4.839647000	1.483263000	-0.009355000
C	4.194056000	2.720474000	-0.074797000
C	4.380271000	-2.265139000	-0.104812000
C	-3.567883000	-1.511424000	0.039627000
C	-4.858273000	-0.856892000	0.058707000
C	-4.916513000	0.595109000	0.063682000
C	-3.682808000	1.350728000	0.049693000
C	4.223327000	0.226215000	-0.145319000
C	6.280342000	1.513822000	0.216118000
C	7.016752000	0.398883000	0.329944000
C	6.403713000	-0.930949000	0.232598000
O	7.066392000	-1.946322000	0.342601000
N	-3.663635000	-2.825386000	0.036688000
S	-5.258798000	-3.200725000	0.055712000
N	-5.873857000	-1.686378000	0.069157000
N	-5.995076000	1.340945000	0.079373000
S	-5.502562000	2.899835000	0.076999000
N	-3.882815000	2.652893000	0.056043000
H	0.287374000	5.184223000	-0.228951000
H	2.980644000	5.269561000	-0.259330000
H	1.687753000	1.245622000	0.050143000
H	-1.515171000	3.176180000	-0.049853000
H	3.378551000	-4.896965000	-0.295425000
H	0.689184000	-5.024492000	-0.255419000
H	1.772486000	-0.983833000	0.033298000
H	-1.263092000	-3.165142000	-0.068076000
H	4.841951000	3.591334000	-0.048120000
H	5.117177000	-3.061336000	-0.087208000
H	3.190036000	0.191552000	-0.459087000
H	6.751890000	2.488318000	0.296989000
H	8.086720000	0.423792000	0.495993000

S_2-OBBPb

Frequencies:

20.9439	23.5817	45.4413
57.9740	72.1675	88.1401
103.7399	110.0580	112.1441
129.5103	136.6777	151.5303
182.3095	194.8576	205.8281
209.6559	242.3486	244.5125
258.5112	275.9272	301.7001
303.7722	308.0501	317.8749
339.2153	357.7592	376.6762
390.3813	407.1724	412.8959
437.7093	484.5844	486.7515
492.3991	513.1639	528.1498
532.1707	552.2565	570.0625
574.0128	586.8081	600.4827
628.6826	655.6800	671.4162
671.9368	684.3400	694.4856
696.4172	705.1374	712.9043
720.2057	726.0307	729.3358
749.7286	754.9638	761.2293
773.8761	791.1286	801.7437
807.5875	808.4990	820.3541
837.9264	838.8287	846.1355
847.4635	853.4379	859.5303
877.5516	896.2787	899.1613
909.1533	916.0625	939.2874
942.1222	948.4964	969.9017
973.3548	997.9022	1002.0565
1012.4229	1019.5575	1037.6771
1044.2060	1056.7515	1079.2612
1113.1030	1155.0881	1159.3392
1179.2428	1203.4240	1210.7780
1222.1278	1244.8708	1262.2544
1278.7804	1300.9665	1307.5499
1319.9616	1328.7077	1357.0168
1367.5978	1392.1543	1413.0202
1423.6046	1431.4286	1435.0318
1445.9340	1454.8510	1464.4962
1479.1124	1484.5312	1502.8029
1529.8834	1538.6744	1560.3600
1563.8334	1577.8093	1588.9301
1617.3573	1625.6576	1640.7345
1646.2977	1660.1998	1687.9489
1704.5302	1772.1431	3184.0731
3190.0609	3194.4645	3228.2819
3236.0106	3237.0794	3253.2087
3260.1464	3265.5958	3274.1085
3282.8807	3526.7573	3651.8093

Coordinates:

C	-1.024730000	4.282418000	0.386583000
C	-2.395471000	4.356197000	0.365332000
C	-2.911564000	3.057381000	0.115738000
N	-1.819363000	2.217494000	0.002649000
C	-0.652778000	2.933649000	0.146124000
C	2.389901000	-0.747024000	0.027955000
C	2.403596000	0.635062000	0.004885000
C	1.044945000	1.104075000	0.066687000
N	0.263563000	-0.028906000	0.109066000
C	1.025234000	-1.175916000	0.102888000
C	0.648711000	2.426193000	0.102948000
C	-2.671867000	-4.074025000	0.237552000
C	-1.325657000	-4.152482000	0.259769000
C	-0.848602000	-2.778429000	0.209940000
N	-1.867538000	-1.893246000	0.171910000
C	-3.005229000	-2.654338000	0.174991000
C	0.520021000	-2.454862000	0.169054000
C	-4.917090000	-0.980748000	-0.051338000
C	-4.878847000	1.467361000	-0.075325000
C	-4.259159000	2.715417000	0.022685000
C	-4.329858000	-2.254581000	0.070569000
C	3.618843000	-1.504827000	-0.020584000
C	4.886680000	-0.816534000	-0.091478000
C	4.903600000	0.637071000	-0.115595000
C	3.652154000	1.360490000	-0.068966000
C	-4.233280000	0.222178000	0.050121000
C	-6.322711000	1.469792000	-0.287201000
C	-7.038290000	0.340688000	-0.392758000
C	-6.397946000	-0.977791000	-0.291550000
O	-7.042891000	-2.005687000	-0.391972000
N	3.744418000	-2.814937000	-0.004747000
S	5.346544000	-3.149686000	-0.073578000
N	5.922225000	-1.620410000	-0.126336000
N	5.959328000	1.412052000	-0.175630000
S	5.425959000	2.957105000	-0.174258000
N	3.814685000	2.666682000	-0.096396000
H	-0.321019000	5.078388000	0.574211000
H	-3.010736000	5.226500000	0.536018000
H	-1.874025000	1.323666000	-0.463274000
H	-0.736425000	-0.125914000	0.266744000
H	1.448070000	3.155019000	0.131982000
H	-3.397344000	-4.874299000	0.250651000
H	-0.695376000	-5.028722000	0.295016000
H	1.239734000	-3.263391000	0.177114000
H	-4.918930000	3.578008000	0.030015000
H	-5.046910000	-3.069581000	0.038567000
H	-3.185105000	0.157719000	0.303590000
H	-6.814109000	2.434913000	-0.364968000
H	-8.109603000	0.344900000	-0.552290000

S_2-OBBPc

Frequencies:

20.8333	24.8112	47.8632
57.5524	71.5487	82.8805
106.2862	109.3097	111.8829
130.3370	139.5884	150.7370
182.0282	198.4503	206.1822
209.9557	242.4287	244.7659
260.9585	273.2840	298.5724
301.2490	316.7822	323.6408
334.1684	359.4940	377.2226
391.0400	409.4355	412.6503
434.3094	481.2955	487.1963
493.4463	511.7278	524.2128
531.7321	547.3374	564.4352
576.2700	589.0963	590.9038
643.6029	660.5555	669.3357
675.4731	686.6759	697.6753
699.1693	705.9361	716.1329
723.0387	725.5857	727.6507
755.1862	759.4673	763.4623
779.2822	792.4281	794.6806
805.2528	810.9017	819.5668
834.8740	841.6268	844.9345
846.0408	851.6930	858.8548
876.5115	902.5557	909.0540
913.9005	924.3325	928.4325
946.3586	955.4088	965.9696
983.0333	992.4056	1006.8159
1014.0585	1018.0985	1037.2191
1043.5518	1063.8889	1079.4127
1111.6993	1149.8985	1164.7416
1180.2010	1192.1311	1201.7358
1222.6534	1259.5192	1272.4371
1278.2137	1287.5389	1309.3365
1326.6018	1330.4747	1350.5243
1364.1442	1393.1451	1411.6062
1419.2901	1430.2214	1436.1216
1444.0807	1449.9846	1468.5229
1473.4210	1488.5960	1496.2380
1526.5208	1546.2141	1556.0776
1568.9011	1575.8615	1599.7668
1608.2034	1626.1167	1638.4401
1651.7834	1657.1521	1685.8405
1700.1953	1769.4122	3175.3025
3190.9362	3204.2325	3229.3059
3231.9087	3236.5144	3249.5892
3263.3531	3264.0992	3271.4214
3281.0335	3536.7907	3648.8052

Coordinates:

C	0.989698000	4.367509000	0.224132000
C	2.340998000	4.391723000	0.192352000
C	2.777234000	3.003351000	0.143233000
N	1.717921000	2.159968000	0.154658000
C	0.619691000	2.965686000	0.192111000
C	-2.350659000	-0.699657000	0.017905000
C	-2.442060000	0.686312000	0.032127000
C	-1.121918000	1.211795000	0.107186000
N	-0.275142000	0.124977000	0.126809000
C	-0.967140000	-1.058423000	0.087390000
C	-0.707507000	2.531924000	0.159865000
C	2.711879000	-4.040745000	0.403515000
C	1.349897000	-4.073309000	0.422617000
C	0.866599000	-2.747431000	0.179978000
N	1.974825000	-1.940573000	0.041014000
C	3.134371000	-2.695790000	0.150816000
C	-0.461095000	-2.355528000	0.138819000
C	4.949828000	-0.953344000	-0.072875000
C	4.830198000	1.491146000	-0.061337000
C	4.146704000	2.700879000	0.037057000
C	4.436875000	-2.261461000	0.056290000
C	-3.539588000	-1.518466000	-0.056548000
C	-4.841976000	-0.891851000	-0.116155000
C	-4.936478000	0.559728000	-0.101970000
C	-3.726715000	1.345861000	-0.028436000
C	4.225362000	0.225140000	0.045525000
C	6.270556000	1.553549000	-0.279540000
C	7.026627000	0.451759000	-0.395379000
C	6.432917000	-0.887657000	-0.308193000
O	7.110738000	-1.893536000	-0.418387000
N	-3.603318000	-2.833073000	-0.075224000
S	-5.188615000	-3.245122000	-0.161103000
N	-5.835835000	-1.743999000	-0.177145000
N	-6.031160000	1.279952000	-0.148503000
S	-5.577934000	2.850419000	-0.103272000
N	-3.954361000	2.641916000	-0.022414000
H	0.296124000	5.194691000	0.256030000
H	2.999478000	5.248198000	0.193172000
H	0.715999000	0.292445000	0.279213000
H	-1.490387000	3.280469000	0.162890000
H	3.396107000	-4.858209000	0.573451000
H	0.707816000	-4.920163000	0.609703000
H	1.961256000	-1.063409000	-0.458823000
H	-1.200824000	-3.144024000	0.181556000
H	4.774232000	3.587445000	-0.006989000
H	5.186394000	-3.045841000	0.077130000
H	3.174813000	0.220877000	0.296309000
H	6.724628000	2.537368000	-0.348113000
H	8.096949000	0.493312000	-0.555708000

S_2-OBBPd

Frequencies:

19.1055	22.3610	42.9592
65.5961	71.9956	81.1376
107.3927	108.6288	119.8991
127.1780	138.6843	150.6193
180.6475	185.9628	198.4745
211.1015	238.9789	245.3630
260.1762	271.2396	302.3092
306.8742	313.2863	324.9120
335.1200	360.1898	369.1322
380.8878	400.1077	415.0085
418.8231	451.7084	485.6855
490.9221	493.2244	505.3110
516.4882	532.4464	562.4164
585.7119	603.6439	626.3453
649.2858	667.1483	679.9189
685.8878	689.9436	700.9059
708.5487	710.6655	727.0243
728.0604	730.2595	731.9360
740.0003	747.6680	779.3731
786.3456	795.6430	802.6536
803.4293	819.1821	828.6436
832.3381	839.2767	849.5980
849.8800	857.5544	860.8644
869.8332	882.6279	890.1046
916.0507	918.3136	947.2520
949.5930	953.2597	960.1915
970.9828	992.0144	1006.8793
1012.3758	1013.9301	1032.4652
1035.0920	1076.0978	1077.9923
1133.5375	1147.5979	1170.8011
1179.2915	1183.2907	1212.6337
1216.6571	1248.8369	1261.2474
1281.4347	1292.4317	1295.2828
1302.9962	1334.1645	1338.0766
1357.6073	1363.7376	1368.8434
1393.9295	1416.0192	1423.2045
1431.9746	1465.0957	1474.6688
1483.4805	1485.9742	1508.8596
1522.5579	1534.0901	1535.1920
1582.0913	1606.2613	1614.0950
1623.1958	1633.4635	1656.4992
1664.7318	1669.4051	1687.1861
1697.9772	1709.5414	3167.5691
3174.5239	3202.9214	3235.6343
3240.0740	3243.1232	3248.2582
3249.6490	3271.2087	3271.5649
3271.7657	3541.4919	3903.0176

Coordinates:

C	-0.954380000	4.386643000	-0.176773000
C	-2.297872000	4.457593000	-0.208607000
C	-2.784957000	3.078114000	-0.152455000
N	-1.714300000	2.196367000	-0.097457000
C	-0.633111000	2.958806000	-0.105666000
C	2.365917000	-0.720659000	0.028584000
C	2.431021000	0.647108000	0.029890000
C	1.073981000	1.166853000	-0.004307000
N	0.248341000	0.063166000	-0.009685000
C	0.964119000	-1.108806000	-0.006510000
C	0.710439000	2.477878000	-0.046707000
C	-2.690538000	-4.064044000	-0.350827000
C	-1.349967000	-4.119323000	-0.297062000
C	-0.898185000	-2.730082000	-0.134053000
N	-1.907296000	-1.874888000	-0.080625000
C	-3.052158000	-2.648125000	-0.199761000
C	0.480376000	-2.383708000	-0.060280000
C	-4.921168000	-0.923135000	0.015475000
C	-4.797559000	1.511590000	-0.018233000
C	-4.112534000	2.779550000	-0.118032000
C	-4.349942000	-2.242296000	-0.152186000
C	3.566756000	-1.524226000	0.049259000
C	4.861345000	-0.881974000	0.071942000
C	4.930787000	0.568891000	0.073036000
C	3.703690000	1.331839000	0.051821000
C	-4.179880000	0.261224000	-0.099545000
C	-6.195506000	1.563607000	0.169323000
C	-6.944256000	0.419395000	0.322787000
C	-6.311889000	-0.821247000	0.273104000
O	-7.107571000	-1.893213000	0.481100000
N	3.647121000	-2.838888000	0.049384000
S	5.236868000	-3.229254000	0.076944000
N	5.868476000	-1.722132000	0.088406000
N	6.013581000	1.309192000	0.090609000
S	5.528177000	2.869913000	0.080985000
N	3.909052000	2.632791000	0.054691000
H	-0.231655000	5.189190000	-0.194129000
H	-2.927188000	5.334542000	-0.258099000
H	-0.768246000	0.113153000	-0.028520000
H	1.510886000	3.205882000	-0.042794000
H	-3.395972000	-4.874406000	-0.467338000
H	-0.702454000	-4.981527000	-0.360774000
H	1.208922000	-3.183082000	-0.070410000
H	-4.769366000	3.645450000	-0.144734000
H	-5.050320000	-3.060152000	-0.302350000
H	-3.115878000	0.196746000	-0.273953000
H	-6.685917000	2.530136000	0.214323000
H	-8.011226000	0.449782000	0.501541000
H	-6.583735000	-2.678591000	0.659020000

S_2-OPBPa

Frequencies:

20.3270	24.5905	44.2640
52.6737	65.6356	87.5951
94.2098	102.0942	108.4906
115.7101	121.5479	131.3533
174.9195	210.3707	216.0545
216.6187	234.2121	240.7396
262.6951	279.4744	298.4531
302.7707	313.8559	329.1380
335.6589	354.7287	381.3109
398.8519	401.3039	413.7982
445.5244	489.5696	494.0143
498.1866	519.9277	524.5163
529.3214	531.9780	571.3937
585.6594	589.2886	615.1244
656.1113	662.5754	681.9760
684.5672	688.0608	689.6565
695.5581	698.2298	721.8921
733.6541	743.4110	748.0751
748.3912	755.8385	778.8534
791.2340	796.2844	797.1873
806.8061	811.8607	814.9882
839.4954	846.1745	847.3054
853.5722	855.7395	859.0224
890.5767	892.4864	904.1679
908.8134	931.2540	942.3800
948.9722	955.9840	996.3801
1006.4774	1011.4333	1013.9082
1014.6527	1031.4564	1036.0287
1046.1252	1088.7386	1122.6565
1164.7231	1189.6764	1192.0648
1194.3836	1215.8256	1219.5979
1235.6742	1262.5384	1268.8587
1275.3532	1290.9822	1302.6166
1352.2053	1380.9275	1389.5522
1399.6022	1412.6720	1419.8279
1434.6741	1453.9737	1458.0152
1472.2085	1474.2130	1487.8769
1504.4305	1515.4356	1552.6778
1563.5634	1570.2980	1584.4103
1596.7282	1603.1708	1619.8979
1621.0839	1658.0096	1660.1905
1676.9871	1705.5061	1778.4957
3196.6687	3209.6858	3219.5388
3229.0666	3229.9087	3230.2450
3263.9109	3265.7663	3281.4646
3283.2558	3630.3237	3684.3210

Coordinates:

C	1.007758000	4.307082000	0.000026000
C	2.370726000	4.345116000	0.000046000
C	2.853233000	2.995047000	0.000074000
N	1.756925000	2.184214000	0.000060000
C	0.608642000	2.933905000	0.000035000
C	-2.390290000	-0.716426000	0.000090000
C	-2.443988000	0.655406000	0.000049000
C	-1.065293000	1.111249000	0.000086000
N	-0.215559000	0.053467000	0.000182000
C	-0.979351000	-1.063235000	0.000166000
C	-0.691093000	2.452858000	0.000017000
C	2.692002000	-4.028414000	-0.000140000
C	1.333379000	-4.095087000	-0.000007000
C	0.827673000	-2.752862000	0.000083000
N	1.914988000	-1.917581000	-0.000011000
C	3.072111000	-2.641628000	-0.000151000
C	-0.502346000	-2.376206000	0.000220000
C	4.923934000	-0.904558000	-0.000162000
C	4.842235000	1.400694000	0.000004000
C	4.196933000	2.644753000	0.000102000
C	4.381699000	-2.195096000	-0.000283000
C	-3.596912000	-1.510258000	0.000068000
C	-4.886842000	-0.854175000	-0.000002000
C	-4.943595000	0.598520000	-0.000054000
C	-3.708699000	1.352883000	-0.000024000
N	4.191311000	0.221318000	-0.000137000
C	6.299732000	1.488105000	0.000120000
C	7.074333000	0.396195000	0.000084000
C	6.438506000	-0.913974000	-0.000090000
O	7.082223000	-1.947076000	-0.000041000
N	-3.692368000	-2.824040000	0.000104000
S	-5.288409000	-3.198344000	0.000080000
N	-5.902699000	-1.683376000	-0.000015000
N	-6.020891000	1.346200000	-0.000111000
S	-5.525983000	2.904662000	-0.000201000
N	-3.905860000	2.655307000	-0.000061000
H	0.316974000	5.136114000	0.000003000
H	3.011712000	5.213386000	0.000047000
H	1.766974000	1.173541000	0.000067000
H	-1.484678000	3.190109000	-0.000064000
H	3.401123000	-4.841916000	-0.000232000
H	0.707017000	-4.973891000	0.000030000
H	1.847576000	-0.909521000	0.000018000
H	-1.237143000	-3.171866000	-0.000051000
H	4.854638000	3.506329000	0.000245000
H	5.123120000	-2.985162000	-0.000447000
H	6.740445000	2.478996000	0.000249000
H	8.156871000	0.431471000	0.000180000

S_2-OPBPb

Frequencies:

19.2290	24.1563	42.8319
54.1680	73.9865	90.9910
98.9383	108.9675	112.2174
125.6052	141.2551	151.7292
178.1047	189.5146	211.0390
211.6536	236.2810	242.2899
261.1119	284.4830	300.5169
306.2281	319.9597	321.2079
345.4659	355.0475	378.7880
397.1065	402.0266	412.6629
440.4454	486.6226	499.0377
512.4011	518.8560	531.7087
532.5421	562.6300	572.2206
587.5302	589.0864	609.8246
654.5110	665.9564	684.7621
688.7154	693.3963	697.6984
703.1459	717.1321	717.8533
726.2308	731.9641	744.1738
761.9661	772.8139	780.8351
793.1275	793.7606	798.7249
803.9905	812.5908	821.9872
836.6286	846.2648	846.2879
853.3341	854.7972	859.0818
883.5208	893.0917	898.7900
912.9236	937.4437	941.7091
944.7550	966.1791	985.4711
997.7151	1005.8464	1011.8777
1021.6231	1034.5436	1043.4898
1061.3063	1081.1924	1108.3652
1155.2823	1162.7574	1174.9719
1198.5671	1218.7674	1225.9471
1242.3661	1265.1421	1286.8051
1302.6214	1304.8177	1318.3533
1347.0029	1364.6761	1385.1957
1410.2566	1416.0647	1430.0449
1433.3090	1446.4984	1459.2383
1465.7909	1481.1260	1497.0395
1512.3068	1538.8841	1542.3429
1550.0487	1568.5177	1580.6680
1598.4546	1614.5609	1627.3657
1643.8123	1652.6252	1663.2903
1684.1299	1705.8910	1778.1424
3197.5196	3204.5963	3212.9022
3229.2720	3231.0814	3237.3481
3254.9057	3267.2590	3275.9989
3284.6043	3352.4746	3537.0429

Coordinates:

C	1.090681000	4.186554000	0.000087000
C	2.446841000	4.305158000	0.000181000
C	3.000180000	2.986869000	0.000130000
N	1.950490000	2.103473000	-0.000024000
C	0.759201000	2.792379000	-0.000037000
C	-2.439200000	-0.767367000	0.000021000
C	-2.397880000	0.620041000	-0.000055000
C	-1.022766000	1.022897000	-0.000170000
N	-0.291683000	-0.131186000	-0.000146000
C	-1.094325000	-1.240622000	-0.000055000
C	-0.548368000	2.326702000	-0.000151000
C	2.648609000	-4.030366000	0.000023000
C	1.309904000	-4.175960000	0.000063000
C	0.767895000	-2.824061000	-0.000016000
N	1.757090000	-1.889586000	-0.000180000
C	2.914430000	-2.587691000	-0.000113000
C	-0.596303000	-2.535940000	-0.000002000
C	4.864957000	-0.896237000	-0.000098000
C	4.955791000	1.410378000	0.000148000
C	4.348865000	2.675633000	0.000197000
C	4.237888000	-2.147551000	-0.000121000
C	-3.697625000	-1.475452000	0.000062000
C	-4.938639000	-0.736371000	0.000028000
C	-4.898939000	0.717914000	-0.000044000
C	-3.619481000	1.393352000	-0.000084000
N	4.227207000	0.283777000	0.000050000
C	6.413132000	1.409801000	0.000195000
C	7.104901000	0.263812000	0.000084000
C	6.378507000	-1.002912000	-0.000113000
O	6.961175000	-2.071371000	-0.000215000
N	-3.873700000	-2.779442000	0.000188000
S	-5.490214000	-3.050272000	0.000158000
N	-6.005879000	-1.498208000	0.000124000
N	-5.925426000	1.533191000	-0.000028000
S	-5.334139000	3.057101000	-0.000115000
N	-3.732631000	2.704715000	-0.000098000
H	0.351923000	4.973257000	0.000078000
H	3.036838000	5.208724000	0.000272000
H	2.141044000	1.103833000	0.000094000
H	0.721384000	-0.289673000	-0.000349000
H	-1.312871000	3.093346000	-0.000217000
H	3.412857000	-4.793768000	0.000088000
H	0.722419000	-5.082106000	0.000163000
H	-1.311821000	-3.348114000	0.000103000
H	5.009177000	3.534990000	0.000287000
H	4.956249000	-2.960359000	-0.000087000
H	6.918059000	2.369947000	0.000322000
H	8.187165000	0.218740000	0.000116000

S_2-OPBPc

Frequencies:

19.7228	22.9471	46.4044
50.1862	74.1381	92.4110
97.6162	110.5451	112.8380
124.4925	142.6593	152.1705
177.1220	190.2320	211.8431
211.9027	237.5301	241.8257
264.2510	280.4322	300.9266
303.7234	322.1049	325.5994
337.4453	356.8446	378.3290
403.3768	404.5910	412.6569
440.0598	486.6371	497.9143
501.4762	519.7847	531.9410
532.2787	561.6998	570.3752
584.1398	590.8244	615.9901
654.6536	666.1723	685.2277
688.5090	694.0758	699.5027
704.9174	717.6421	719.3002
726.1952	732.6649	745.8815
765.2068	773.9576	783.6154
793.9427	798.2671	806.6504
807.2354	814.9883	824.6114
835.5650	845.0156	847.1020
851.1049	854.3936	858.3687
891.1358	899.7627	912.6517
915.1301	923.3826	949.4714
955.9485	958.0060	986.7072
1002.0870	1008.3815	1014.5304
1018.6750	1036.2665	1042.3481
1066.7493	1083.2728	1116.5124
1161.5151	1177.5302	1178.3505
1191.9871	1205.8514	1224.9013
1255.7515	1269.5204	1277.7974
1298.1181	1308.0326	1322.1138
1345.3470	1365.7829	1389.2532
1404.7348	1415.6049	1430.5083
1437.1647	1446.5205	1455.3866
1464.1076	1477.6103	1494.5943
1509.4856	1537.9337	1546.7188
1550.4448	1567.1452	1585.6546
1607.3579	1610.7991	1627.5918
1648.3436	1654.0010	1660.7996
1683.5294	1697.4813	1777.5609
3187.2270	3204.2519	3228.9224
3229.4802	3230.2314	3238.9977
3249.9564	3264.1875	3271.4440
3281.9025	3371.3422	3541.8086

Coordinates:

C	-0.975876000	4.387758000	0.000057000
C	-2.323460000	4.344595000	0.000167000
C	-2.696239000	2.928273000	0.000061000
N	-1.600080000	2.144567000	-0.000102000
C	-0.538769000	3.001048000	-0.000158000
C	2.354089000	-0.677262000	-0.000011000
C	2.500370000	0.704695000	-0.000102000
C	1.197045000	1.276890000	-0.000202000
N	0.312413000	0.230072000	-0.000157000
C	0.954052000	-0.974011000	-0.000045000
C	0.796931000	2.606913000	-0.000325000
C	-2.751883000	-3.988904000	-0.000427000
C	-1.393813000	-3.972708000	-0.000405000
C	-0.956279000	-2.603538000	-0.000074000
N	-2.091838000	-1.827413000	0.000082000
C	-3.207222000	-2.629198000	-0.000119000
C	0.380067000	-2.241184000	0.000027000
C	-5.023073000	-0.909193000	0.000036000
C	-4.774282000	1.382829000	0.000118000
C	-4.053218000	2.583696000	0.000150000
C	-4.524359000	-2.220821000	-0.000041000
C	3.513223000	-1.541125000	0.000101000
C	4.840106000	-0.964782000	0.000124000
C	4.990351000	0.482456000	0.000030000
C	3.809464000	1.314412000	-0.000087000
N	-4.216761000	0.157227000	0.000048000
C	-6.225960000	1.560215000	0.000162000
C	-7.073938000	0.522112000	0.000160000
C	-6.529709000	-0.828339000	0.000136000
O	-7.230296000	-1.824033000	0.000050000
N	3.526674000	-2.857269000	0.000213000
S	5.097201000	-3.330105000	0.000244000
N	5.801963000	-1.855235000	0.000253000
N	6.112524000	1.160648000	0.000044000
S	5.717334000	2.747518000	-0.000101000
N	4.084616000	2.601054000	-0.000162000
H	-0.322541000	5.247614000	0.000090000
H	-3.023256000	5.167712000	0.000315000
H	-0.685791000	0.460736000	-0.000227000
H	1.573261000	3.361459000	-0.000204000
H	-3.411312000	-4.843031000	-0.000638000
H	-0.715163000	-4.811893000	-0.000594000
H	-2.207008000	-0.816572000	0.000347000
H	1.084435000	-3.063284000	0.000057000
H	-4.690162000	3.462551000	0.000288000
H	-5.269428000	-3.006843000	-0.000029000
H	-6.604220000	2.576537000	0.000195000
H	-8.151306000	0.634151000	0.000185000

S_2-OPBPd

Frequencies:

18.6072	22.0836	42.2567
62.1979	73.8190	80.3381
105.9705	108.7229	119.3331
123.5332	132.2803	150.3189
178.6392	191.6159	194.6322
212.1499	235.0354	244.0449
261.3200	273.8887	304.6325
310.9016	313.6106	328.2611
337.1622	359.9488	377.6210
393.0237	405.7173	414.9529
421.0927	448.2242	484.8740
487.0809	493.2161	509.6134
518.9256	532.1807	580.3646
586.7934	622.9039	645.2358
650.4510	669.1126	680.3612
686.9157	697.6288	703.3235
709.4698	714.6930	726.9703
731.0977	734.9896	738.0462
776.0401	787.2211	787.8689
795.1706	802.2752	804.8875
817.2509	818.5774	829.2394
834.9637	848.4223	849.4773
856.6163	857.0162	861.1929
869.3311	880.7340	900.9976
915.3763	915.8414	950.5590
951.6576	959.8897	965.6595
991.8847	1011.1968	1015.7771
1018.1561	1030.3768	1038.8419
1075.7843	1077.1241	1131.7879
1146.8401	1169.1530	1178.1995
1191.6794	1209.6700	1220.8409
1246.8317	1253.9198	1277.5330
1294.7162	1301.2261	1324.3060
1334.1207	1340.2203	1357.3883
1365.2474	1394.9518	1412.4871
1423.6282	1430.7512	1467.3484
1470.6357	1478.2164	1487.8566
1509.7891	1513.7950	1528.3398
1535.7800	1583.7324	1609.5063
1616.4669	1623.7727	1628.6071
1651.4163	1663.2463	1669.2617
1682.8917	1712.1539	1718.1248
3167.7622	3183.4809	3217.3999
3234.9498	3241.7188	3244.5104
3248.1524	3249.2915	3271.4039
3271.4636	3441.3465	3890.4401

Coordinates:

C	-0.990068000	4.357806000	-0.326907000
C	-2.331040000	4.394887000	-0.387493000
C	-2.789362000	3.001302000	-0.269007000
N	-1.702030000	2.151638000	-0.155500000
C	-0.641689000	2.936878000	-0.180391000
C	2.362238000	-0.718233000	0.031950000
C	2.423891000	0.649304000	0.044845000
C	1.065525000	1.163240000	-0.014725000
N	0.240386000	0.060857000	-0.037385000
C	0.961910000	-1.106626000	-0.033634000
C	0.703698000	2.475512000	-0.077452000
C	-2.704213000	-3.997173000	-0.554422000
C	-1.368193000	-4.085783000	-0.486975000
C	-0.892551000	-2.713461000	-0.233288000
N	-1.881665000	-1.842502000	-0.140439000
C	-3.037105000	-2.578901000	-0.316411000
C	0.485210000	-2.384032000	-0.122719000
C	-4.899918000	-0.857844000	0.033605000
C	-4.809437000	1.438444000	-0.026766000
C	-4.112135000	2.698127000	-0.213600000
C	-4.328956000	-2.167254000	-0.227633000
C	3.564351000	-1.519373000	0.066959000
C	4.856724000	-0.874119000	0.117031000
C	4.922437000	0.576894000	0.130891000
C	3.693995000	1.337261000	0.094292000
N	-4.198715000	0.261023000	-0.169954000
C	-6.177388000	1.538748000	0.305508000
C	-6.901471000	0.400290000	0.575249000
C	-6.250831000	-0.821148000	0.470524000
O	-6.962111000	-1.924297000	0.790839000
N	3.648207000	-2.833747000	0.057626000
S	5.238334000	-3.220420000	0.107758000
N	5.865700000	-1.711832000	0.143841000
N	6.003068000	1.319351000	0.172912000
S	5.514349000	2.879037000	0.166694000
N	3.896248000	2.638615000	0.110819000
H	-0.284508000	5.174604000	-0.370965000
H	-2.980038000	5.252515000	-0.492064000
H	-0.782052000	0.108294000	-0.081236000
H	1.501134000	3.206571000	-0.066623000
H	-3.426268000	-4.781723000	-0.730805000
H	-0.737613000	-4.955909000	-0.597482000
H	1.214068000	-3.182842000	-0.141672000
H	-4.772155000	3.559127000	-0.271996000
H	-5.048825000	-2.961023000	-0.411897000
H	-6.644520000	2.513915000	0.371996000
H	-7.941076000	0.425061000	0.876871000
H	-6.376286000	-2.674388000	0.927882000

S_2-OPBPe

Frequencies:

19.5757	24.5099	46.3217
52.7581	65.0100	96.3504
103.3438	108.1318	118.3234
124.8178	133.7008	140.0788
182.9608	186.5193	214.1447
214.6820	239.2878	241.9573
261.6906	280.5098	291.5290
302.1854	314.6762	317.7946
338.7820	358.8079	374.6259
400.4829	403.6558	412.0864
432.2354	487.8426	498.6699
505.9700	516.1135	532.0711
537.0729	563.6635	578.1419
584.3924	610.5255	656.7443
678.6703	692.1873	695.1670
696.8036	699.8242	707.9787
725.6496	725.8962	727.4358
733.0168	743.2224	759.4282
764.9799	769.8814	781.4356
794.0224	798.3958	803.3998
807.1251	821.6088	832.6519
833.8855	844.2666	845.3265
847.4451	851.5529	858.5827
881.7644	913.5280	917.0384
918.7833	928.6475	950.1162
953.0056	966.7550	980.4947
992.8158	1009.7861	1018.6290
1023.9604	1036.6658	1044.6234
1078.1259	1082.3516	1148.8954
1154.3315	1160.5067	1186.9189
1198.0905	1212.1988	1232.2315
1258.2817	1268.5132	1270.0955
1296.1124	1313.8297	1324.9678
1351.9004	1362.4052	1372.7641
1391.1729	1403.9303	1428.4397
1429.3028	1459.6631	1462.0321
1475.2234	1486.1574	1507.1599
1529.0218	1546.5140	1557.6696
1561.3377	1568.5576	1585.5494
1623.7045	1625.9895	1630.8319
1650.2123	1652.6871	1674.8493
1689.4899	1705.4780	1788.8554
3209.8893	3219.1067	3221.5555
3227.0926	3229.0461	3235.8402
3250.7827	3253.4411	3272.7258
3274.8357	3468.2047	3521.2658

Coordinates:

C	1.002525000	4.320825000	-0.000348000
C	2.348425000	4.362809000	-0.000119000
C	2.794470000	2.969640000	0.000209000
N	1.767418000	2.113400000	0.000135000
C	0.645101000	2.908225000	-0.000189000
C	-2.375216000	-0.729063000	-0.000161000
C	-2.429437000	0.665471000	-0.000161000
C	-1.094714000	1.146194000	-0.000315000
N	-0.281958000	0.050414000	-0.000422000
C	-1.007012000	-1.104221000	-0.000314000
C	-0.662891000	2.478126000	-0.000389000
C	2.676861000	-4.041460000	-0.000061000
C	1.332668000	-4.106276000	-0.000259000
C	0.864661000	-2.725123000	-0.000239000
N	1.922156000	-1.843665000	-0.000107000
C	3.012448000	-2.616626000	0.000069000
C	-0.471839000	-2.399544000	-0.000354000
C	4.926137000	-0.959198000	0.000214000
C	4.831759000	1.453111000	0.000334000
C	4.169884000	2.662936000	0.000438000
C	4.362871000	-2.206966000	0.000267000
C	-3.587344000	-1.517035000	-0.000014000
C	-4.873409000	-0.856019000	0.000142000
C	-4.929923000	0.598606000	0.000137000
C	-3.698875000	1.357164000	-0.000019000
N	4.234635000	0.225720000	0.000331000
C	6.278941000	1.498113000	0.000158000
C	7.043242000	0.396624000	0.000006000
C	6.429118000	-0.926637000	0.000034000
O	7.088725000	-1.946382000	-0.000041000
N	-3.684678000	-2.828976000	0.000029000
S	-5.282691000	-3.199936000	0.000165000
N	-5.891053000	-1.682026000	0.000302000
N	-6.008496000	1.343267000	0.000300000
S	-5.519255000	2.904062000	0.000109000
N	-3.897471000	2.657691000	0.000027000
H	0.296069000	5.137785000	-0.000614000
H	2.999828000	5.224467000	-0.000156000
H	0.737029000	0.088604000	-0.000559000
H	-1.445962000	3.226821000	-0.000659000
H	3.397459000	-4.845905000	0.000009000
H	0.691994000	-4.975768000	-0.000386000
H	-1.193945000	-3.207191000	-0.000485000
H	4.822325000	3.527659000	-0.000429000
H	5.096455000	-3.004190000	0.000239000
H	3.212916000	0.183805000	0.000421000
H	6.727181000	2.484229000	0.000134000
H	8.125392000	0.435906000	-0.000135000

S_2-OxBPa

Frequencies:

26.6585	30.8551	58.4834
74.7512	77.2760	103.8592
108.1482	118.7169	127.2024
140.7198	145.7985	177.3891
189.0392	210.6129	212.3383
229.1577	253.8884	264.0399
305.8473	309.7614	318.3559
327.9221	344.9034	364.5847
386.6192	389.4547	408.8791
411.4939	436.4980	453.8700
489.6160	495.4639	517.3413
532.3538	580.8760	654.8862
666.3564	677.6973	678.8116
680.9706	694.9680	696.7985
700.0943	710.9402	712.8294
725.4524	728.5046	734.6653
746.4147	755.1762	774.7665
782.4539	792.9592	793.0255
802.9109	816.1189	817.4865
820.2114	838.0073	845.1145
854.3919	858.3460	863.5113
876.3000	886.4136	913.7312
915.2843	916.5606	929.6900
947.3513	948.8642	950.8283
968.5824	984.7958	1002.1660
1014.6164	1021.4348	1034.7661
1045.3855	1058.5222	1075.1951
1075.9693	1117.3867	1158.8498
1182.4393	1185.0870	1198.2332
1206.5175	1254.5092	1259.9248
1272.5424	1313.5911	1326.3364
1356.6315	1364.3820	1372.0143
1394.7603	1399.5508	1422.7137
1430.7939	1436.4324	1451.0792
1454.1995	1474.2816	1507.7871
1529.7754	1544.1624	1557.8050
1565.6016	1576.4171	1577.9048
1616.3884	1627.3457	1627.6454
1654.1552	1654.4245	1677.2338
1688.8610	3214.6449	3214.7852
3230.5218	3230.7460	3247.9672
3247.9776	3268.2205	3269.6735
3269.6946	3285.2621	3432.3313

Coordinates:

C	1.605958000	-4.258291000	0.000035000
C	2.953232000	-4.223359000	0.000076000
C	3.319947000	-2.802606000	0.000055000
N	2.240037000	-2.014319000	0.000004000
C	1.174934000	-2.861086000	0.000043000
C	-1.949377000	0.695545000	0.000062000
C	-1.949377000	-0.695545000	0.000053000
C	-0.592595000	-1.128372000	0.000048000
N	0.180739000	0.000000000	0.000051000
C	-0.592595000	1.128372000	0.000062000
C	-0.145522000	-2.454345000	0.000056000
C	2.953232000	4.223359000	0.000039000
C	1.605958000	4.258291000	0.000073000
C	1.174934000	2.861086000	0.000055000
N	2.240037000	2.014319000	-0.000005000
C	3.319947000	2.802606000	-0.000007000
C	-0.145522000	2.454345000	0.000083000
C	6.514244000	-0.681301000	-0.000120000
C	6.514244000	0.681301000	-0.000148000
C	5.152379000	1.098722000	-0.000077000
O	4.358463000	0.000000000	0.000007000
C	5.152380000	-1.098722000	-0.000013000
C	4.662054000	-2.380958000	0.000046000
C	4.662054000	2.380958000	-0.000087000
H	1.206102000	0.000000000	0.000063000
C	-3.191247000	1.437056000	0.000026000
C	-4.451513000	0.727444000	-0.000041000
C	-4.451513000	-0.727444000	-0.000050000
C	-3.191247000	-1.437056000	0.000008000
N	-3.341035000	2.744435000	0.000012000
S	-4.951626000	3.053141000	-0.000006000
N	-5.501081000	1.512545000	-0.000090000
N	-5.501081000	-1.512545000	-0.000109000
S	-4.951626000	-3.053141000	-0.000043000
N	-3.341035000	-2.744435000	-0.000023000
H	0.949260000	-5.115873000	0.000027000
H	3.650161000	-5.049010000	0.000098000
H	-0.920506000	-3.211128000	0.000082000
H	3.650161000	5.049010000	0.000031000
H	0.949260000	5.115873000	0.000104000
H	-0.920506000	3.211128000	0.000133000
H	7.358535000	-1.352944000	-0.000166000
H	7.358534000	1.352944000	-0.000223000
H	5.418661000	-3.157269000	0.000040000
H	5.418661000	3.157269000	-0.000025000

S_2-OxBPb

Frequencies:

26.6619	28.0101	58.3584
73.8133	78.2543	103.0902
109.1402	113.4890	134.9285
136.6703	147.7845	175.1658
191.9659	201.9737	209.5563
231.1177	249.4667	263.0208
303.9050	312.3077	325.6058
327.3532	347.4761	364.9761
379.4859	391.6848	409.0109
413.1605	442.3154	452.2346
490.5392	498.8604	520.9975
530.9712	584.0766	650.4227
659.7843	670.7965	677.4226
687.6208	691.0620	694.3360
695.3134	698.8690	708.1237
729.5465	734.6508	735.9054
746.8151	751.3224	774.2582
779.0182	779.3417	796.6932
800.6163	807.7846	810.5631
821.4279	832.5343	843.5070
850.9401	852.3200	857.4944
862.6692	886.2398	896.7038
914.5357	935.1956	935.5724
945.2267	946.7463	953.8004
974.9217	983.0546	1011.4210
1018.7369	1027.2187	1029.2450
1037.8890	1054.6488	1072.8301
1081.2673	1131.8635	1178.6865
1184.3902	1196.2316	1203.2285
1216.4378	1246.1397	1265.5858
1270.1921	1305.6060	1324.3432
1354.9241	1366.5236	1372.7066
1398.7284	1404.9316	1420.6901
1428.5550	1446.9914	1455.6928
1464.4910	1474.5537	1479.3131
1520.0697	1535.3480	1540.6709
1572.6331	1580.6901	1586.3381
1596.5254	1619.3408	1621.3424
1633.3527	1659.6311	1676.0825
1700.4446	3211.3075	3213.7423
3224.3004	3226.6443	3245.7549
3263.3936	3267.7728	3269.1591
3280.7386	3285.9628	3443.2555

Coordinates:

C	-1.601741000	4.181092000	0.000053000
C	-2.949060000	4.204229000	0.000043000
C	-3.364019000	2.796885000	-0.000022000
N	-2.321319000	1.964174000	0.000066000
C	-1.222914000	2.763549000	0.000061000
C	2.030150000	-0.711742000	0.000078000
C	1.984406000	0.662466000	0.000017000
C	0.568789000	1.019072000	0.000104000
N	-0.189269000	-0.088473000	0.000045000
C	0.646165000	-1.148302000	0.000054000
C	0.096563000	2.342463000	0.000084000
C	-2.978460000	-4.155795000	-0.000095000
C	-1.614452000	-4.196501000	-0.000064000
C	-1.136975000	-2.851699000	-0.000007000
N	-2.240808000	-2.026011000	-0.000005000
C	-3.376233000	-2.782026000	-0.000067000
C	0.196445000	-2.466941000	0.000029000
C	-6.565902000	0.698083000	0.000050000
C	-6.576780000	-0.666420000	0.000034000
C	-5.220572000	-1.088846000	-0.000050000
O	-4.416438000	0.003185000	-0.000073000
C	-5.206692000	1.114911000	-0.000008000
C	-4.712621000	2.394759000	-0.000009000
C	-4.712737000	-2.364671000	-0.000104000
C	3.285024000	-1.422431000	0.000084000
C	4.528872000	-0.683136000	0.000020000
C	4.486139000	0.770176000	-0.000050000
C	3.201787000	1.440985000	-0.000054000
N	3.464561000	-2.727417000	0.000115000
S	5.082657000	-2.996675000	0.000042000
N	5.597173000	-1.443538000	0.000032000
N	5.511636000	1.587131000	-0.000127000
S	4.914255000	3.109644000	-0.000092000
N	3.314191000	2.753512000	-0.000098000
H	-0.910531000	5.011487000	0.000071000
H	-3.611727000	5.057608000	0.000049000
H	0.857588000	3.115723000	0.000094000
H	-3.670133000	-4.984071000	-0.000145000
H	-0.973304000	-5.064663000	-0.000082000
H	-2.162126000	-1.004170000	0.000033000
H	0.936356000	-3.258932000	0.000028000
H	-7.407698000	1.372976000	0.000104000
H	-7.426136000	-1.331253000	0.000068000
H	-5.466409000	3.174087000	-0.000004000
H	-5.449221000	-3.158394000	-0.000105000
